# ARNTL2 promotes metastatic phenotypes through SERPINE1 transcription in KRAS G12D pancreatic cancer

**DOI:** 10.1016/j.isci.2026.117479

**Published:** 2026-09-08

**Authors:** Xiaohan Shi, Xiaoyi Yin, Suizhi Gao, Xinqian Wu, Huan Wang, Beilei Wang, Xiongfei Xu, Kailian Zheng, Wei Jing, Gang Li, Shiwei Guo, Gang Jin

**Affiliations:** 1 Department of Hepatobiliary Pancreatic Surgery, Changhai Hospital, Naval Medical University, Shanghai 200433, China

**Keywords:** pancreatic ductal adenocarcinoma, metastasis, KRAS G12D mutation, ARNTL2, SERPINE1, epithelial-mesenchymal transition

## Abstract

Despite the prevalence of the KRAS G12D mutation in approximately 40% of pancreatic ductal adenocarcinoma (PDAC) cases and its association with the poorest prognosis among KRAS variants, the downstream transcriptional effectors driving its aggressive metastatic phenotype remain poorly defined. Through integrated bioinformatics and experimental validation, we identified ARNTL2 as a factor associated with KRAS G12D-mutant PDAC. ARNTL2 directly bound to the SERPINE1 promoter to activate its expression, establishing an ARNTL2-SERPINE1 axis that orchestrated epithelial-mesenchymal transition and drove metastasis *in vitro* and *in vivo*. Rescue experiments confirmed SERPINE1 as the indispensable downstream mediator. Clinical validation demonstrated that ARNTL2 and SERPINE1 protein levels correlated positively and stratified patients into distinct prognostic subgroups. Furthermore, this axis altered sensitivity to selective KRAS G12D inhibition *in vitro*. These findings identify an experimentally supported ARNTL2-SERPINE1 transcriptional axis in KRAS G12D-mutant PDAC that warrants further validation in genotype-faithful and clinically relevant models.

## Highlights

• ARNTL2 is upregulated in KRAS G12D-mutant PDAC and predicts poor prognosis
• ARNTL2 transcriptionally activates SERPINE1 via direct promoter binding
• The ARNTL2-SERPINE1 axis drives EMT and metastasis *in vitro* and *in vivo*
• The ARNTL2-SERPINE1 axis alters sensitivity to KRAS G12D inhibition *in vitro*

## Graphical abstract

**Figure:**
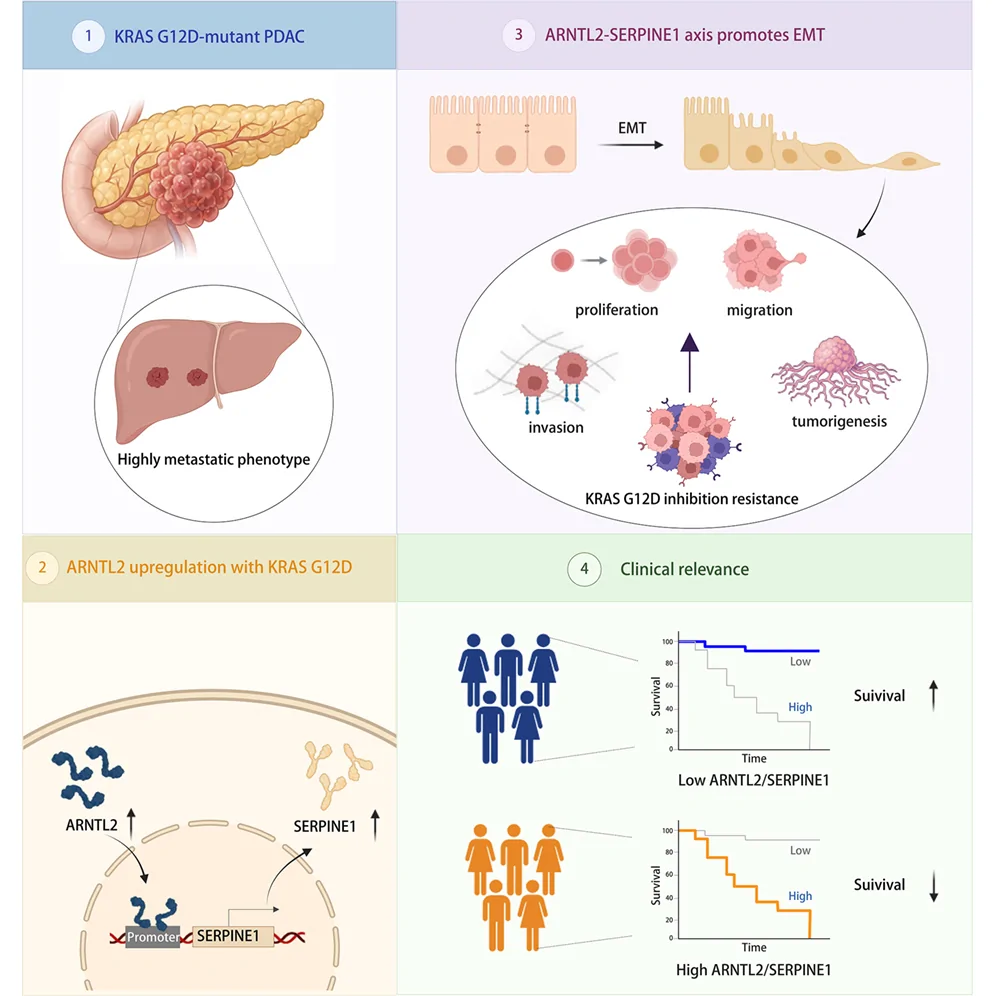

## Teaser

Molecular interaction; Molecular network; Cancer

## Introduction

Pancreatic ductal adenocarcinoma (PDAC) is a highly lethal malignancy with a dismal 5-year survival rate, largely attributable to its insidious onset, profound tumor heterogeneity, and aggressive metastatic behavior.^1^^,^^2^ The advancements in molecular profiling have gradually dissected this complexity, revealing distinct molecular subtypes and subtype-specific drivers of disease progression.^3^^,^^4^ Oncogenic KRAS mutations are the foundational genetic event in over 90% of PDAC cases.^5^ However, the consequences of KRAS mutations are not uniform, where different point mutations within codon 12 (G12D, G12V, and G12R) cf. unique biological properties and clinical outcomes.^6^

Among these, the KRAS G12D mutation is the most prevalent allele, accounting for approximately 40% of PDAC cases.^7^^,^^8^ Accumulating clinical evidence from the real-world patient cohorts has established a strong association between KRAS G12D status and a significantly more aggressive disease course, characterized by poorer overall survival (OS) compared to other KRAS variants.^9^ Consistent with our previous reports,^10^^,^^11^ many studies suggested that KRAS G12D uniquely orchestrated a more metastatic phenotype,^12^^,^^13^ with potentially distinct downstream transcriptional programs, metabolic rewiring, or induction of epithelial-to-mesenchymal transition (EMT),^13^^,^^14^^,^^15^ while detailed molecular mechanisms remained to be further elucidated.

The advent of selective, potent KRAS G12D inhibitors marks a transformative milestone in the targeted therapy of this high-risk patient subset, demonstrating significant preclinical and early clinical anti-tumor efficacy.^16^^,^^17^^,^^18^ However, the inevitable emergence of primary and acquired resistance remains a major clinical challenge, as observed with KRAS G12C inhibitors.^19^ The sobering precedent underscores an unmet need to delineate the unique downstream effectors and dependencies specific to KRAS G12D-mutant PDAC. Identifying such cooperative vulnerabilities is crucial for developing rational combination therapies that could enhance the efficacy of direct KRAS inhibition, delay or overcome resistance, and expand the therapeutic selection for this prognostically unfavorable subtype.

In this context, we aimed to elucidate novel molecular mediators that specifically contribute to the aggressive metastasis of KRAS G12D-mutant PDAC. Through an integrated analysis of public datasets and a series of *in vitro* and *in vivo* functional studies, we identified ARNTL2 (aryl hydrocarbon receptor nuclear translocator-like 2) as a key transcriptional regulator specifically upregulated in KRAS G12D tumors and associated with poor prognosis. We further unveiled a previously unrecognized oncogenic axis wherein ARNTL2 transcriptionally activates SERPINE1 (serpin family E member 1, also known as PAI-1) to drive metastatic progression by promoting EMT. Moreover, the ARNTL2-SERPINE1 axis promoted resistance to selective KRAS G12D inhibition. Our findings not only revealed a novel mechanistic link between the KRAS G12D mutation and enhanced metastatic potential but also nominated the ARNTL2-SERPINE1 signaling axis as a promising therapeutic target for combination strategies to improve outcomes in patients with KRAS G12D-mutant PDAC.

## Results

### Elevated ARNTL2 expression correlated with KRAS G12D mutation and predicted poor prognosis in PDAC

**Figure 1:**
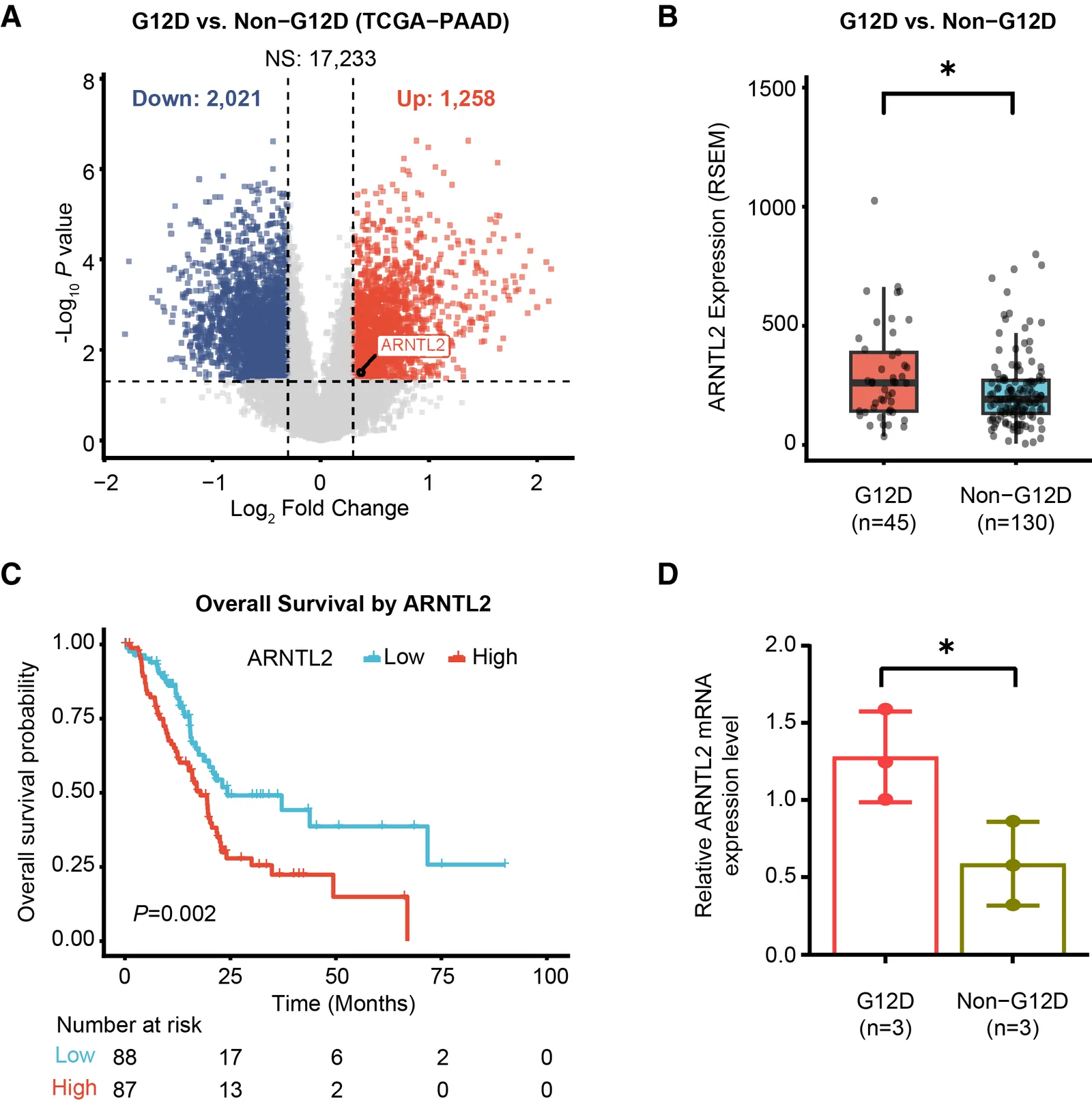
ARNTL2 expression in relation to KRAS G12D mutation status, patient prognosis, and its validation across pancreatic cancer cell lines (A) Differential gene-expression analysis comparing KRAS G12D-mutant (*n* = 45) versus non-G12D (*n* = 130) tumor samples in the TCGA-PAAD cohort (PanCancer Atlas). Volcano plot shows log_2_ fold change (*x* axis) versus −log_10_*p* value (*y* axis). Significantly, 1,258 upregulated genes are shown in red and 2,021 downregulated genes in blue, while 17,233 non-significant genes are shown in gray. Dashed vertical and horizontal lines indicate the prespecified fold change and significance cutoffs used for calling differentially expressed genes. ARNTL2 is annotated among the upregulated candidates. *n* represents the number of biologically independent tumor samples from distinct patients. (B) ARNTL2 mRNA expression (RNA-Seq by expectation-maximization [RSEM]) in TCGA-PAAD tumors (PanCancer Atlas) stratified by KRAS status (G12D, *n* = 45; non-G12D, *n* = 130). Individual data points are overlaid to show sample distribution. Boxplots show the median (center line), interquartile range (box), and 1.5× interquartile range (IQR) (whiskers). *n* represents the number of biologically independent tumor samples from distinct patients. *p* values were calculated using the two-sided Wilcoxon rank-sum test. ∗*p* < 0.05. (C) Kaplan-Meier curves for overall survival in TCGA-PAAD (PanCancer Atlas, *n* = 175), stratified by median ARNTL2 expression into ARNTL2-high (*n* = 87) and ARNTL2-low (*n* = 88). *n* represents the number of distinct patients. *p* values were calculated using the log-rank test. The numbers of patients at risk at each time point are listed below the curves. (D) qPCR validation of ARNTL2 mRNA levels in pancreatic cancer cell lines grouped by KRAS genotype (G12D: SW1990, PANC-1, AsPC-1, *n* = 3; non-G12D: BxPC-3, Capan-1, and MIAPaCa-2, *n* = 3). Relative expression was quantified after normalization to GAPDH and is presented as mean ± SD with individual data points shown. *n* represents the number of biologically independent cell lines. *p* values were calculated using the two-tailed Student’s *t* test. ∗*p* < 0.05.

To investigate the potential mechanisms underlying the aggressive metastasis of KRAS G12D-mutant PDAC, we performed a differential expression analysis comparing the G12D group (*n* = 45) against the non-G12D group (*n* = 130) within The Cancer Genome Atlas (TCGA)-pancreatic adenocarcinoma (PAAD) cohort (PanCancer Atlas). A total of 1,258 upregulated genes in the G12D group and 2,021 upregulated genes in the non-G12D group were identified (Figure 1A), among which ARNTL2 was significantly upregulated in the G12D cohort (*p* = 0.044) (Figure 1B). While ARNTL2 was not the top-ranked gene by fold-change, its implication in promoting metastasis in other solid tumors, including estrogen receptor-negative breast cancer, lung adenocarcinoma, and colon cancer,^20^^,^^21^^,^^22^ and its unexplored role in PDAC, particularly in the context of the KRAS G12D mutation, prompted us to prioritize it for functional investigation.

We subsequently evaluated the impact of ARNTL2 expression on patient prognosis using Kaplan-Meier survival analysis. Patients were stratified into low (*n* = 88) and high (*n* = 87) expression groups based on the median ARNTL2 expression value, and the results demonstrated that high ARNTL2 expression is significantly associated with poorer OS (*p* = 0.002) (Figure 1C), while no significant correlation was observed between ARNTL2 expression and American Joint Committee on Cancer (AJCC) stage (Figure S1A). To determine if the clinical relevance of ARNTL2 expression was independent of other factors, we performed a multivariate Cox regression analysis, which demonstrated an elevated ARNTL2 level as a significant predictor of poor clinical outcomes in PDAC patients (Figure S1B).

To further confirm the observed association between ARNTL2 expression and KRAS G12D mutation, we examined ARNTL2 mRNA expression levels *in vitro* using a panel of pancreatic cell lines. Quantitative analysis revealed differential ARNTL2 expression across cell lines, with higher expression observed in lines carrying the G12D mutation (SW1990, PANC-1, and AsPC-1) compared to controls (BxPC-3 for KRAS wild type (WT), Capan-1 for KRAS G12V, and MIAPaCa-2 for KRAS G12C) (*p* = 0.015) (Figure 1D), which supported our findings in the public database. These results suggested that elevated ARNTL2 expression was closely associated with poor patient prognosis and might represent a critical mechanism for the highly metastatic phenotype of KRAS G12D-mutant PDAC.

### ARNTL2 conferred malignant phenotypes of KRAS G12D-mutant PDAC by enhancing proliferation and metastatic potential

To explore the biological function of ARNTL2 in KRAS G12D-mutant PDAC, we first constructed cellular models with modulated ARNTL2 expression. We established stable knockdown models in KRAS G12D-mutant lines (SW1990 and PANC-1) and an overexpression model in the KRAS WT line (BxPC-3). Quantitative PCR (qPCR) verified significantly reduced ARNTL2 mRNA levels in the knockdown groups (Figures S2A and S2B) and elevated levels in the BxPC-3 overexpression group (Figure S2C), with corresponding changes observed at the protein level as confirmed by western blotting analysis (Figures S2D, S2E, and S2F).

**Figure 2:**
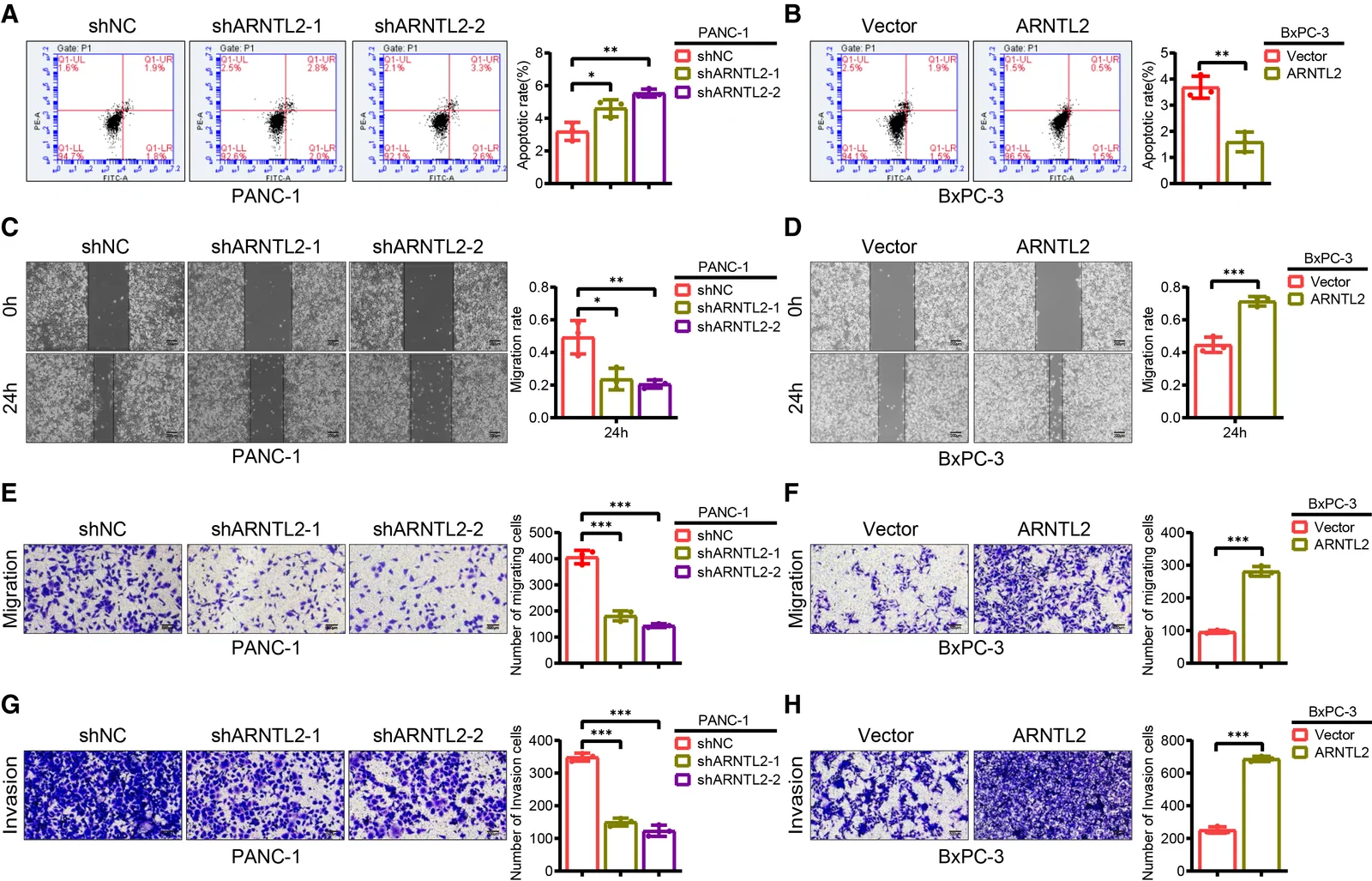
ARNTL2 conferred malignant phenotypes of KRAS G12D-mutant PDAC by suppressing apoptosis and enhancing metastatic potential (A and B) Flow cytometric analysis of cellular apoptosis using Annexin V-FITC/Phycoerythrin (PE) staining. (A) PANC-1 cells were transfected with negative control shRNA (shNC) or two independent shRNAs targeting ARNTL2 (shARNTL2-1 and shARNTL2-2). (B) BxPC-3 cells were transfected with an empty vector or an ARNTL2 overexpression plasmid. Representative scatterplots (left) display the distribution of cell populations, and the bar graphs (right) quantify the total percentage of apoptotic cells (sum of Q1-UR and Q1-LR quadrants). (C and D) Wound-healing assays assessing lateral cell motility. Representative images of the wound area were captured at 0 h and 24 h post-scratching for (C) PANC-1 cells with ARNTL2 knockdown and (D) BxPC-3 cells with ARNTL2 overexpression. Histograms quantify the migration rate calculated as the percentage of wound closure relative to the 0 h baseline. Scale bars represent 200 μm. (E and F) Transwell migration assays. Cells were seeded into the upper chamber without Matrigel and allowed to migrate for 24 h. Representative micrographs of crystal violet-stained cells on the lower surface of the membrane are shown for (E) PANC-1 and (F) BxPC-3 cells under the indicated transfection conditions. Histograms display the average number of migrated cells counted in five random fields. Scale bars represent 500 μm. (G and H) Transwell invasion assays using Matrigel-coated chambers. The invasive capacity of (G) PANC-1 and (H) BxPC-3 cells was evaluated following the same staining and quantification protocols as the migration assay. Scale bars represent 500 μm. Statistical analysis: representative images derive from three biologically independent experiments. Data are presented as mean ± SD from three biologically independent experiments with individual data points shown. Statistical significance between groups was determined using one-way ANOVA followed by Tukey’s post hoc test (for PANC-1 groups) or the two-tailed Student’s *t* test (for BxPC-3 groups). ∗*p* < 0.05, ∗∗*p* < 0.01, and ∗∗∗*p* < 0.001.

We next assessed whether ARNTL2 contributes to tumor growth. CCK-8 proliferation assays demonstrated that silencing ARNTL2 significantly suppressed the growth rate of SW1990 and PANC-1 cells (Figures S2G and S2H), whereas overexpression in BxPC-3 cells markedly promoted viability (Figure S2I). To determine if this growth alteration was driven by regulation of cell death pathways, we performed flow cytometry analysis. The results indicated that ARNTL2 knockdown significantly increased the apoptotic rate in the mutant cells (Figures 2A and S2J), while overexpression attenuated apoptosis in the WT cells (Figure 2B).

We further evaluated the functional role of ARNTL2 in regulating tumor metastatic capacity. Scratch wound-healing assays revealed that ARNTL2 depletion impaired the lateral migration capability of SW1990 and PANC-1 cells, resulting in delayed wound closure compared to controls (Figures 2C and S2K). Conversely, ARNTL2 overexpression accelerated wound healing in BxPC-3 cells (Figure 2D). To validate the findings using a more rigorous model and to assess invasive potential, we performed Transwell assays. Consistent with the wound-healing data, ARNTL2 knockdown significantly reduced the number of vertically migrating cells (Figures 2E and S2L), while upregulation enhanced migration (Figure 2F). Furthermore, Matrigel invasion assays demonstrated that ARNTL2 silencing significantly attenuated the ability of cells to penetrate the matrix (Figures 2G and S2M), whereas overexpression potentiated the invasive capacity of BxPC-3 cells (Figure 2H). Collectively, these *in vitro* data provided preliminary evidence that ARNTL2 acted as an oncogene, promoting both the proliferation and the metastatic behavior of KRAS G12D-mutant PDAC.

### ARNTL2 facilitated tumorigenesis and distant metastasis of KRAS G12D-mutant PDAC in xenograft models

**Figure 3:**
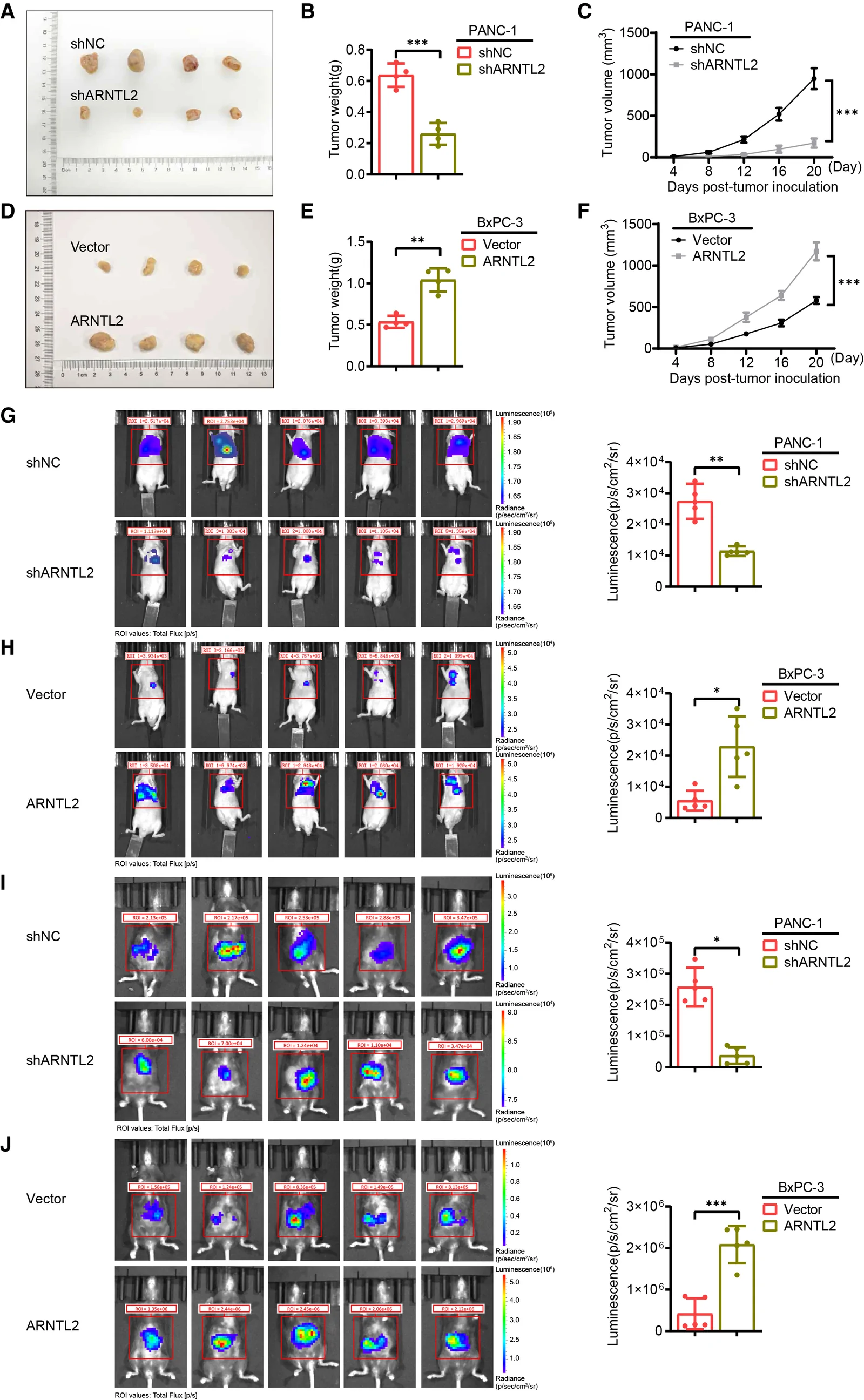
ARNTL2 facilitated tumorigenesis and distant metastasis of KRAS G12D-mutant PDAC in xenograft models (A–F) Assessment of tumor growth in subcutaneous xenograft models. BALB/c nude mice were inoculated subcutaneously in the flank with indicated pancreatic cancer cells (*n* = 4 per group). *n* represents the number of biological replicates per group. (A–C) Evaluation of ARNTL2 knockdown in PANC-1 cells. (A) Photographs of excised tumors from the shNC (control) and shARNTL2 groups harvested at day 20. (B) Comparison of terminal tumor weights. (C) Longitudinal tumor growth curves measuring tumor volume (V = 0.5×length×width^2^) every 4 days post-inoculation. (D–F) Evaluation of ARNTL2 overexpression in BxPC-3 cells. (D) Images of dissected tumors from the vector and ARNTL2-overexpressing groups. (E) Quantification of tumor weights at the endpoint. (F) Tumor volume growth kinetics over the 20-day observation period. (G–J) Assessment of metastatic colonization using bioluminescence imaging. Luciferase-labeled cancer cells were injected into nude mice (*n* = 5 per group), and metastatic burden was monitored via the in vivo imaging system (IVIS) spectrum system. *n* represents the number of biological replicates per group. The heatmaps represent the bioluminescence intensity ranging from blue (low) to red (high). (G and H) Lung metastasis model established via tail-vein injection. IVIS images (left) and quantification of photon flux (right) in the pulmonary region for (G) PANC-1 cells (shNC vs. shARNTL2) and (H) BxPC-3 cells (Vector vs. ARNTL2) at day 21 post-injection. (I and J) Liver metastasis model established via intrasplenic injection. IVIS images (left) and quantification of photon flux (right) indicating hepatic tumor burden for (I) PANC-1 cells with ARNTL2 knockdown and (J) BxPC-3 cells with ARNTL2 overexpression at day 21 post-injection. Statistical analysis: data are presented as mean ± SD from four or five biologically independent experiments with individual data points shown. Statistical comparisons were performed using the two-tailed Student’s *t* test. ∗*p* < 0.05, ∗∗*p* < 0.01, and ∗∗∗*p* < 0.001.

To substantiate the oncogenic role of ARNTL2 observed *in vitro*, we first established subcutaneous xenograft models to assess tumor growth dynamics *in vivo*. PANC-1 cells with stable ARNTL2 knockdown and BxPC-3 cells with ARNTL2 overexpression were implanted into nude mice. Longitudinal monitoring revealed that silencing ARNTL2 significantly abrogated tumorigenic capacity, resulting in markedly reduced tumor weight and volume compared to controls (Figures 3A–3C). Conversely, the overexpression of ARNTL2 in BxPC-3 cells significantly accelerated tumor proliferation, leading to a substantial increase in tumor burden (Figures 3D–3F). Immunohistochemical analysis on the excised tumor tissues confirmed that ARNTL2 protein levels remained suppressed in the knockdown group (Figure S3A) and elevated in the overexpression group throughout the experimental period (Figure S3B).

We then assessed the potential of ARNTL2 to drive metastatic colonization with a tail vein injection model. Bioluminescence imaging of the lungs revealed that ARNTL2 knockdown (PANC-1) significantly inhibited the formation of lung metastases compared to the control group (Figure 3G), while ARNTL2 overexpression (BxPC-3) resulted in a strong photon flux, indicating extensive metastatic colonization (Figure 3H). An intrasplenic injection model was also used to recapitulate hepatic metastasis. Consistent with above findings, silencing ARNTL2 (PANC-1) effectively prevented hepatic colonization (Figure 3I), whereas mice injected with ARNTL2-overexpressing cells (BxPC-3) exhibited profound bioluminescent signals in the liver (Figure 3J), indicative of robust metastatic burden. These results provided definitive evidence that ARNTL2 not only fueled primary tumor growth but also critically potentiated metastatic progression.

### ARNTL2 promoted KRAS G12D-mutant PDAC metastasis via the upregulation of SERPINE1

To elucidate the molecular mechanism by which ARNTL2 mediated the metastatic phenotype, we screened for potential downstream targets according to public databases. Considering the transcriptional regulatory role of ARNTL2, we identified genes associated with ARNTL2 expression levels by analyzing the TCGA PAAD cohort (PanCancer Atlas) (Figure S4A), which revealed a significant positive correlation between ARNTL2 and SERPINE1 expression (*R* = 0.21, *p* = 0.005) (Figure S4B). SERPINE1 has been previously reported to be closely associated with tumor metastasis in various cancers.^23^^,^^24^^,^^25^ However, its pro-metastatic function and underlying mechanisms in PDAC remained largely unknown. Kaplan-Meier analysis confirmed that high SERPINE1 expression is significantly associated with poor OS (*p* = 0.008) (Figure S4C).

**Figure 4:**
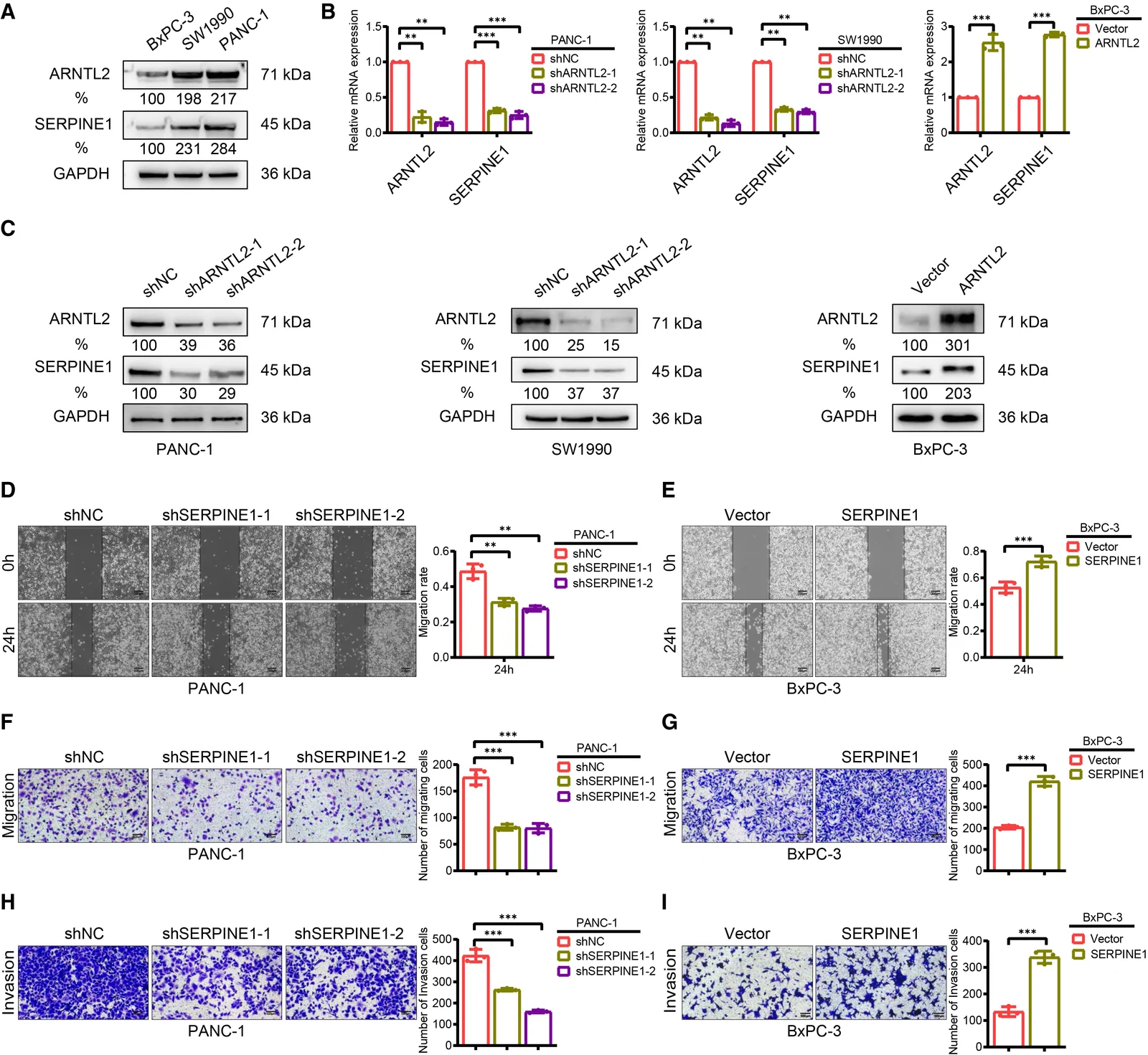
ARNTL2 positively regulates SERPINE1 expression and SERPINE1 mediates metastatic behaviors in KRAS G12D-mutant PDAC (A) Western blot analysis of baseline endogenous ARNTL2 and SERPINE1 protein levels across three pancreatic cancer cell lines (BxPC-3, SW1990, and PANC-1). GAPDH served as the loading control. Molecular weights (kDa) are indicated on the right. Numbers underneath the blots indicate relative band intensities after normalization against loading controls with BxPC-3 set as 100%. (B and C) Validation of SERPINE1 regulation by ARNTL2 at the mRNA and protein levels. (B) Relative mRNA expression of ARNTL2 and SERPINE1 measured by RT-qPCR. (C) Immunoblotting of ARNTL2 and SERPINE1 proteins. The left and middle images show PANC-1 and SW1990 cells transfected with shNC or shARNTL2 (shARNTL2-1/2), while the right image shows BxPC-3 cells transfected with an empty Vector or ARNTL2 overexpression plasmid. GAPDH served as the loading control. Molecular weights (kDa) are indicated on the right. Numbers underneath the blots indicate relative band intensities after normalization against loading controls with shNC or vector set as 100%. (D and E) Wound-healing assays assessing the effect of SERPINE1 modulation on cell motility. Representative images of wound closure were captured at 0 h and 24 h. (D) PANC-1 cells transfected with shNC or shSERPINE1 (shSERPINE1-1/2). (E) BxPC-3 cells transfected with vector or SERPINE1 expression plasmid. Histograms quantify the migration rate calculated as the percentage of wound closure relative to the 0 h baseline. Scale bars represent 200 μm. (F and G) Transwell migration assays. Cells were seeded in the upper chamber without Matrigel and allowed to migrate for 24 h. Representative micrographs of crystal violet-stained cells on the lower surface of the membrane are shown for (F) PANC-1 cells with SERPINE1 knockdown and (G) BxPC-3 cells with SERPINE1 overexpression. Histograms display the average number of migrated cells counted in five random fields. Scale bars represent 500 μm. (H and I) Transwell invasion assays using Matrigel-coated chambers. The invasive capacity of (H) PANC-1 cells (shNC vs. shSERPINE1) and (I) BxPC-3 cells (vector vs. SERPINE1) was evaluated following the same staining and quantification protocols as the migration assay. Scale bars represent 500 μm. Statistical analysis: representative images derive from three biologically independent experiments. Data are expressed as mean ± SD from three biologically independent experiments with individual data points shown. Statistical significance was determined using one-way ANOVA followed by Tukey’s post-hoc test for multiple comparisons (PANC-1 and SW1990 groups) or the two-tailed Student’s *t* test for two-group comparisons (BxPC-3 groups). ∗*p* < 0.05, ∗∗*p* < 0.01, and ∗∗∗*p* < 0.001.

To translate these bioinformatics associations into biological evidence, we examined protein expression in our cell models. Western blot analysis showed that basal SERPINE1 expression mirrored ARNTL2 levels, being notably higher in KRAS G12D-mutant cell lines compared to KRAS WT cells (Figure 4A). Crucially, genetic manipulation supported that ARNTL2 acted as a positive regulator of SERPINE1: knockdown of ARNTL2 suppressed SERPINE1 mRNA and protein expression (Figures 4B and 4C), while ARNTL2 overexpression upregulated its expression (Figures 4B and 4C).

Before establishing a causal axis, we sought to verify that SERPINE1 functioned as an independent driver of metastasis in this context. We established stable knockdown models in KRAS G12D-mutant lines (SW1990 and PANC-1) and an overexpression model in the KRAS WT line (BxPC-3), which were confirmed by qPCR (Figures S4D–S4F) and western blot analysis (Figures S4G–S4I). Scratch wound-healing assays demonstrated that SERPINE1 knockdown significantly impaired lateral migration in SW1990 and PANC-1 cells (Figures 4D and S4L), while overexpression enhanced it in BxPC-3 cells (Figure 4E). Consistent with this, Transwell assays revealed that SERPINE1 silencing reduced both vertical migration (Figures 4F, 4G, and S4M) and invasion (Figures 4H, 4I, and S4N). These findings indicated that SERPINE1 was functionally capable of recapitulating the pro-metastatic phenotypes observed with ARNTL2.

**Figure 5:**
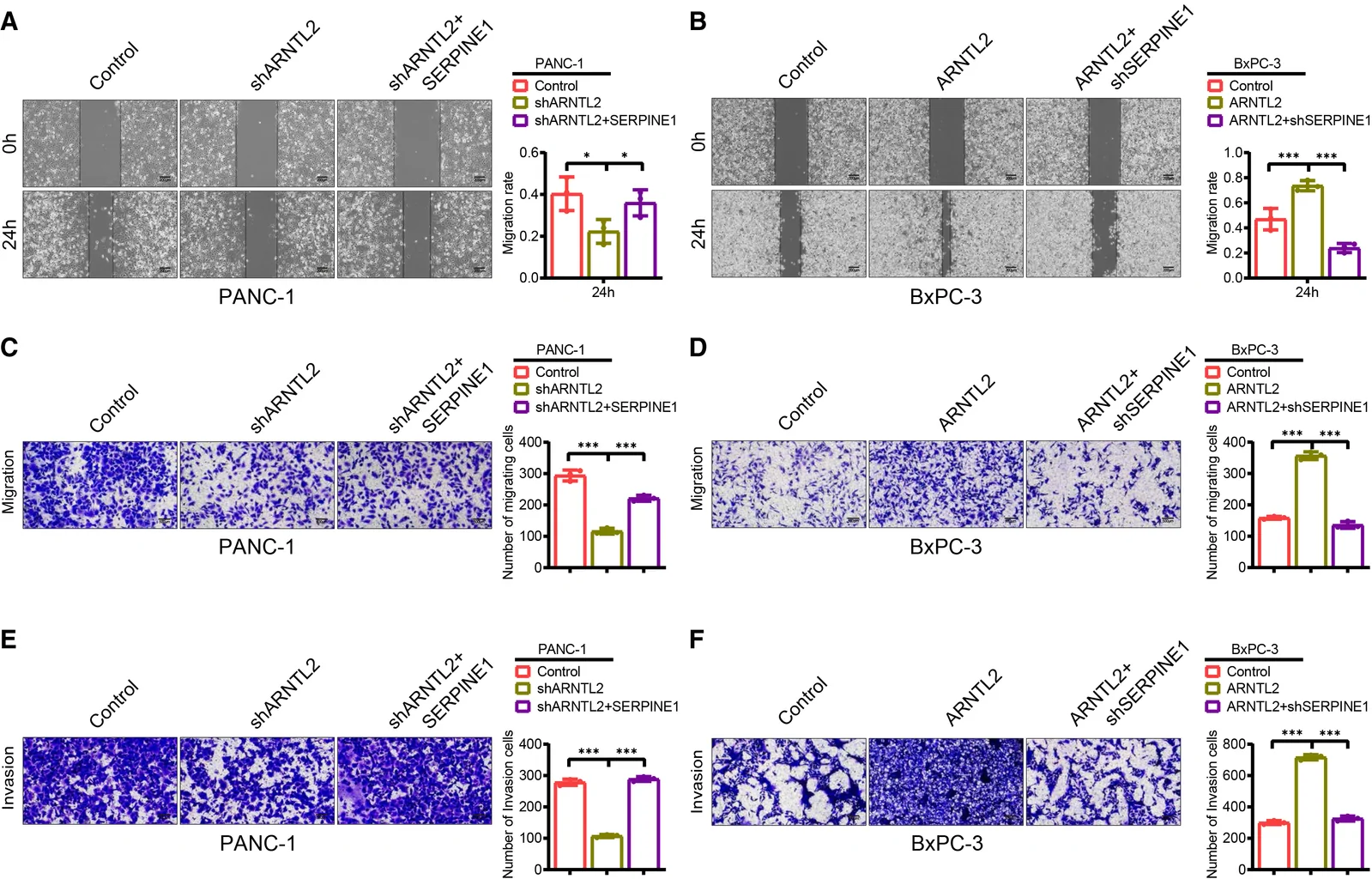
SERPINE1 mediates ARNTL2-induced migration and invasion in KRAS G12D-mutant PDAC (A and B) Wound-healing assays assessing lateral cell motility. (A) PANC-1 cells were assigned to three groups: control (shNC), ARNTL2 knockdown (shARNTL2), and rescue group (co-transfected with shARNTL2 and a SERPINE1 overexpression plasmid). (B) BxPC-3 cells were assigned to three groups: control (vector), ARNTL2 overexpression (ARNTL2), and reversal group (co-transfected with ARNTL2 plasmid and shSERPINE1). Representative images of the wound area were captured at 0 h and 24 h post-scratching (left). The bar graphs (right) quantify the migration rate calculated as the percentage of wound closure relative to the 0 h baseline. Scale bars represent 200 μm. (C and D) Transwell migration assays. Cells were seeded into the upper chamber without Matrigel and allowed to migrate for 24 h. Representative micrographs of crystal violet-stained cells on the lower surface of the membrane are shown for (C) PANC-1 cells and (D) BxPC-3 cells under the specified rescue conditions. Histograms display the average number of migrated cells counted in five random fields. Scale bars represent 500 μm. (E and F) Transwell invasion assays using Matrigel-coated chambers. The invasive capability of (E) PANC-1 and (F) BxPC-3 cells was evaluated under the specified rescue conditions following the same staining and quantification protocols as the migration assay. Scale bars represent 500 μm. Statistical analysis: representative images derive from three biologically independent experiments. Data are presented as mean ± SD from three biologically independent experiments with individual data points shown. Statistical significance for multiple group comparisons was determined using one-way ANOVA followed by Tukey’s post hoc test. ∗*p* < 0.05, ∗∗*p* < 0.01, and ∗∗∗*p* < 0.001.

Finally, to definitively prove that SERPINE1 was the critical effector mediating ARNTL2-induced metastasis, we performed rescue experiments. In ARNTL2-silenced PANC-1 cells, the ectopic re-introduction of SERPINE1 successfully restored the impaired migratory potential (Figure 5A). Conversely, in ARNTL2-overexpressing BxPC-3 cells, the concomitant knockdown of SERPINE1 abolished the enhanced migratory phenotype (Figure 5B). This rescue effect was corroborated across multiple independent Transwell assays (Figures 5C–5F), where SERPINE1 modulation consistently reversed the migration defects or enhancements caused by ARNTL2 manipulation. Collectively, these results demonstrated that the pro-metastatic function of ARNTL2 in KRAS G12D-mutant PDAC is dependent on the upregulation of SERPINE1.

### ARNTL2 transcriptionally activated SERPINE1 to orchestrate the metastasis-associated EMT program

**Figure 6:**
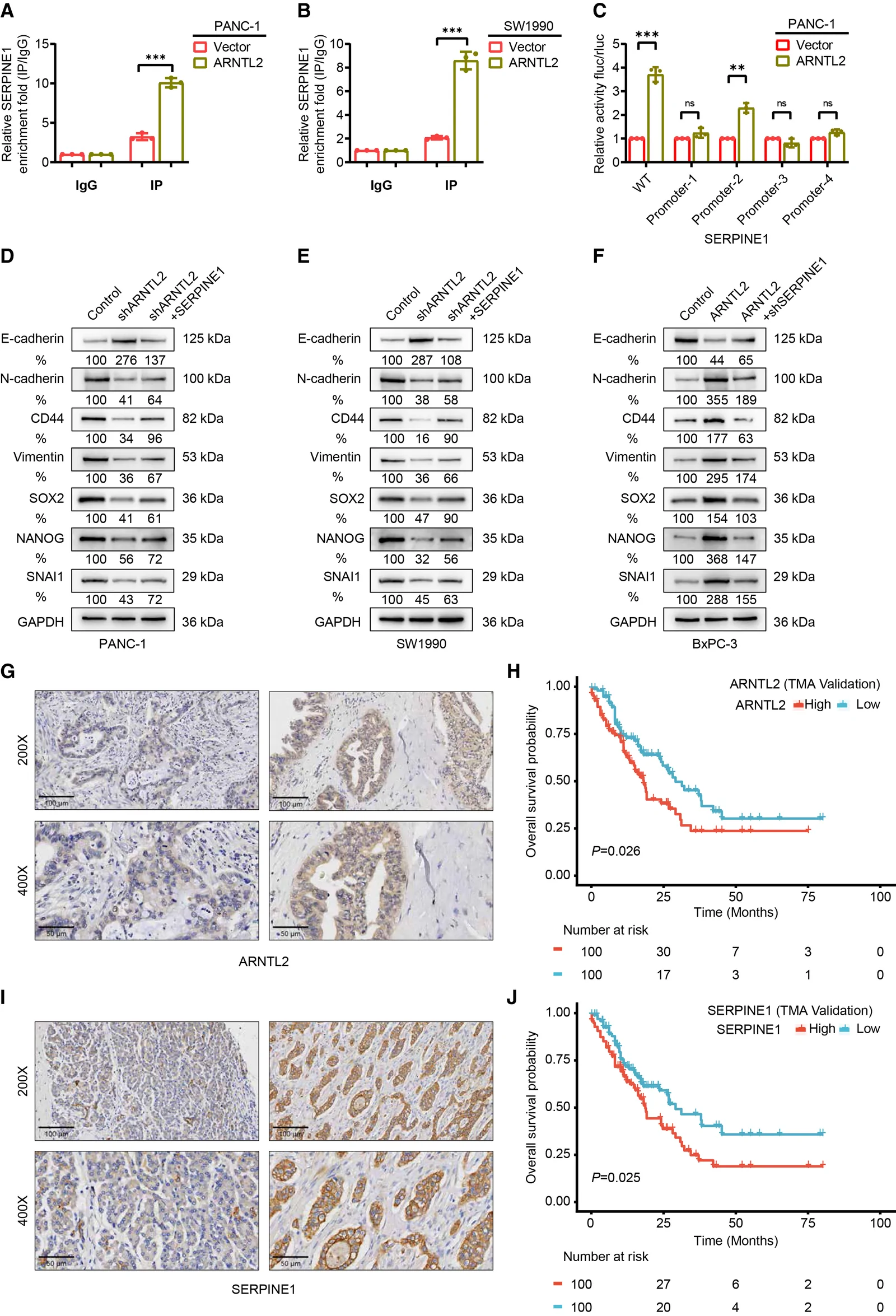
ARNTL2 transcriptionally activates SERPINE1 to drive EMT and cancer stemness, with clinical prognostic validation in PDAC patients (A and B) Chromatin immunoprecipitation (ChIP) assays coupled with qPCR analysis in PANC-1 (A) and SW1990 (B) cells. Cells were transfected with either an empty vector or an ARNTL2 expression vector. The enrichment of ARNTL2 on the SERPINE1 promoter region was quantified using specific primers. Data are presented as fold enrichment relative to the IgG negative control (mean ± SD) from three biologically independent experiments with individual data points shown. *p* values were calculated using the two-tailed Student’s *t* test; ∗∗∗*p* < 0.001. (C) Dual-luciferase reporter assays in PANC-1 cells. Cells were co-transfected with an ARNTL2 expression plasmid (or vector) and luciferase reporter constructs containing either the SERPINE1 wild type (WT) promoter or truncated promoter fragments (promoter-1 to promoter-4). Relative luciferase activity (Renilla/Firefly) was measured 48 h post-transfection. Data are presented as mean ± SD from three biologically independent experiments with individual data points shown. *p* values were calculated using the two-tailed Student’s *t* test. ∗∗*p* < 0.01; ns, not significant. (D–F) Western blot analysis of EMT-related proteins (E-cadherin, N-cadherin, Vimentin, and SNAI1) and cancer stemness markers (CD44, SOX2, and NANOG). (D) PANC-1 cells were subjected to ARNTL2 knockdown (shARNTL2) or concurrent ARNTL2 knockdown and SERPINE1 overexpression (shARNTL2 + SERPINE1). (E) SW1990 cells were treated as in (D). (F) Cells were subjected to ARNTL2 overexpression or concurrent ARNTL2 overexpression and SERPINE1 knockdown (ARNTL2 + shSERPINE1). GAPDH was used as the loading control. Molecular weights (kDa) are indicated. Numbers underneath the blots indicate relative band intensities after normalization against loading controls with control set as 100%. (G–J) Representative immunohistochemistry micrographs of pancreatic cancer tissue microarray (TMA) derived from 200 biologically independent tumor samples from distinct patients (G and I). (G) Staining for ARNTL2 protein. (I) Staining for SERPINE1 protein. Images are displayed at 200× (scale bars: 100 μm) and 400× (scale bars: 50 μm) magnifications to visualize cytoplasmic and nuclear distribution. (H and J) Kaplan-Meier survival analysis based on the TMA cohort (*n* = 200). (H) Overall survival probability stratified by ARNTL2 expression levels (high vs. low). (J) Overall survival probability stratified by SERPINE1 expression levels (high vs. low). *p* values were calculated using the log rank test. The numbers of patients at risk at each time point are listed below the respective curves. *n* represents number of distinct patients.

Having established that ARNTL2 regulated SERPINE1 protein levels, we sought to define the precise molecular mechanism by examining whether ARNTL2 could directly bind to the SERPINE1 promoter. We first performed chromatin immunoprecipitation (ChIP) assays in SW1990 and PANC-1 cells, which confirmed significant enrichment of ARNTL2 on the SERPINE1 promoter region compared to IgG controls (Figures 6A and 6B). To further verify the functional consequence of this binding, dual-luciferase reporter assays were conducted. Co-transfection of ARNTL2 significantly amplified the luciferase activity driven by the WT SERPINE1 promoter; crucially, this activation was abolished when the specific binding sites were mutated (Figures 6C and S5). These data definitively established ARNTL2 as a direct transcriptional activator of SERPINE1.

To exclude the possibility that ARNTL2 regulates SERPINE1 protein stability, we performed cycloheximide (CHX) chase experiments. ARNTL2 knockdown in PANC-1 and SW1990 cells, as well as ARNTL2 overexpression in BxPC-3 cells, did not alter SERPINE1 protein degradation rates (Figure S6), confirming that ARNTL2 regulates SERPINE1 exclusively at the transcriptional level.

To understand the biological process by which this ARNTL2-SERPINE1 axis facilitated metastasis, we utilized gene set enrichment analysis (GSEA). The analysis revealed that SERPINE1 expression is highly correlated with “EMT pathway” and “metastasis-associated pathways” signatures (Figure S7A). This bioinformatics evidence suggests that ARNTL2 may drive metastasis by triggering the EMT, a critical step in cancer dissemination (Figure S7B).

We experimentally validated this hypothesis by analyzing key EMT markers via western blot. In PANC-1 and SW1990 cells, ARNTL2 knockdown reversed the EMT phenotype, resulting in the upregulation of the epithelial marker E-cadherin and the downregulation of mesenchymal markers (N-cadherin, Vimentin, and SNAI1) (Figures 6D and 6E). Conversely, ARNTL2 overexpression in BxPC-3 cells induced a robust “cadherin switch,” characterized by the loss of E-cadherin and the gain of N-cadherin (Figure 6F). To prove that this EMT induction was specifically dependent on SERPINE1, we performed rescue experiments. In ARNTL2-silenced cells (PANC-1 and SW1990), the ectopic re-expression of SERPINE1 restored the mesenchymal phenotype, increasing N-cadherin, Vimentin, and SNAI1 levels while suppressing E-cadherin (Figures 6D and 6E). Reciprocally, in ARNTL2-overexpressing BxPC-3 cells, the concomitant knockdown of SERPINE1 successfully blocked the EMT process, restoring E-cadherin expression (Figure 6F). These results demonstrated that ARNTL2 expression was sufficient to induce the molecular hallmarks of EMT with SERPINE1 as the indispensable downstream effector.

Given the well-established functional interplay between EMT and cancer stemness in driving metastasis, we further examined whether the ARNTL2-SERPINE1 axis concurrently regulates stemness markers. Western blot analysis revealed that ARNTL2 knockdown in PANC-1 and SW1990 cells significantly downregulated the expression of established cancer stemness markers, including CD44, SOX2, and NANOG, and this effect was fully rescued by ectopic SERPINE1 re-expression (Figures 6D and 6E). Conversely, ARNTL2 overexpression in BxPC-3 cells upregulated these stemness markers, which was abolished by concomitant SERPINE1 silencing (Figure 6F).

To further validate the stemness-promoting function of the ARNTL2-SERPINE1 axis in a more physiologically relevant context, we performed 3D spheroid formation assays, which serve as a functional readout for cancer stemness. In BxPC-3 cells, ARNTL2 overexpression significantly enhanced both spheroid number and diameter, phenocopying SERPINE1 overexpression; this effect was abolished by concurrent SERPINE1 knockdown (Figures S8A and S8B). Conversely, in PANC-1 and SW1990 cells, ARNTL2 knockdown markedly reduced spheroid formation and size, and these defects were rescued by ectopic SERPINE1 expression (Figures S8A and S8B). These 3D-culture data corroborate our stemness marker findings.

Finally, to corroborate these mechanistic findings *in vivo*, we performed immunohistochemistry (IHC) staining on xenograft tumor tissues. Consistent with our *in vitro* models, tumors from the ARNTL2-overexpression group exhibited strong staining for SERPINE1 and mesenchymal markers (N-cadherin, Vimentin, and Snail) (Figure S3A), whereas the ARNTL2-knockdown group displayed a marked reduction in these proteins (Figure S3B). Moreover, immunohistochemical staining of xenograft tumor tissues confirmed that ARNTL2 overexpression enhanced the expression of cancer stemness markers CD44, SOX2, and NANOG, whereas ARNTL2 knockdown reduced their levels (Figures S3A and S3B), corroborating the *in vitro* findings in an *in vivo* context. Collectively, these findings delineated a complete signaling axis where ARNTL2 directly transactivated SERPINE1 to initiate the EMT program and cancer stemness, thereby fueling pancreatic cancer metastasis.

### Clinical validation of prognostic value of the ARNTL2-SERPINE1 axis in PDAC

To establish the clinical relevance of our findings, we analyzed a tissue microarray (TMA) comprised of PDAC patient samples. Kaplan-Meier survival analysis revealed that patients with high ARNTL2 protein expression had significantly shorter OS compared to those with low expression (median OS: 18.0 vs. 29.0 months, hazard ratio (HR) 1.589, 95% confidence interval [CI]: 1.058–2.385, *p* = 0.026), confirming its prognostic value (Figures 6G and 6H). Parallel analysis of SERPINE1 expression demonstrated a similar trend, where elevated SERPINE1 levels were associated with poor clinical outcomes (median OS: 18.5 vs. 29.0 months, HR 1.594, 95% CI: 1.059–2.397, *p* = 0.025) (Figures 6I and 6J). Furthermore, correlation analysis of the TMA data revealed a significant positive linear correlation between ARNTL2 and SERPINE1 protein expression in human tissues (*R* = 0.25, *p* < 0.001) (Figure S9). Collectively, these clinical data validate our experimental findings, identifying the ARNTL2-SERPINE1 axis as a clinically significant driver of pancreatic cancer progression and a potent predictor of patient prognosis.

### The ARNTL2-SERPINE1 axis confers resistance to KRAS G12D-targeted therapy

Given the clinical emergence of selective KRAS G12D inhibitors and the inevitable challenge of therapeutic resistance, we investigated whether the ARNTL2-SERPINE1 axis modulates sensitivity to zoldonrasib (RMC-9805), a potent non-covalent KRAS G12D inhibitor currently under clinical evaluation. Dose-response assays in PANC-1 and SW1990 cells revealed that ARNTL2 overexpression significantly increased the inhibitory concentration (IC50) of zoldonrasib, indicating reduced drug sensitivity (Figure S10). Notably, SERPINE1 overexpression alone phenocopied this resistance, while concurrent SERPINE1 knockdown in ARNTL2-overexpressing cells restored zoldonrasib sensitivity to near-baseline levels (Figure S10). These data establish the ARNTL2-SERPINE1 axis as a functional driver of therapeutic resistance to KRAS G12D inhibition and nominate SERPINE1 as a potential combinatorial target to enhance the effectiveness of KRAS-directed therapies.

## Discussion

This study established ARNTL2 as a novel driver of metastasis in KRAS G12D-mutant PDAC. We delineated the following key advances: (1) ARNTL2 expression was preferentially elevated in KRAS G12D-mutant PDAC and served as an independent prognostic factor; (2) ARNTL2 functionally promoted malignant behaviors of KRAS G12D-mutant PDAC cells *in vitro* and fueled tumor growth and metastasis *in vivo*; (3) mechanistically, ARNTL2 bound to and transcriptionally activated SERPINE1, inducing an EMT program; and (4) the protein levels of both ARNTL2 and SERPINE1 correlated positively with each other and individually predicted inferior survival in patient tissues. Our findings illuminated a clinically relevant transcriptional axis linking the most common PDAC KRAS genotype to heightened metastatic propensity.

Our identification of ARNTL2 enrichment in KRAS G12D-mutant PDAC elucidated an underappreciated facet of KRAS oncogenic complexity. KRAS mutations, though ubiquitous, were not functionally monolithic.^26^ Congruent with prior clinical data indicating KRAS G12D as the poorest prognostic subtype among KRAS alleles,^7^^,^^8^ our TCGA mining revealed KRAS G12D as the predominant subtype, within which ARNTL2 exhibited significant upregulation. Unlike broader studies linking circadian clock disruptions to PDAC pathogenesis, none has specifically connected ARNTL2 to a particular KRAS mutant allele.^27^ ARNTL2 has been implicated in enhancing glycolysis and conferring sensitivity to erlotinib in PDAC,^28^ and its overexpression correlated with aggressive phenotypes in other cancers.^20^^,^^21^^,^^22^^,^^29^ However, our work is the first to establish ARNTL2 as a KRAS G12D-associated modulator, offering a discrete molecular correlation for the heightened malignancy of this subtype and underscoring the necessity of stratified approaches to KRAS-targeted oncobiology.^30^

The mechanistic dissection of ARNTL2-mediated metastasis converged on SERPINE1, its direct transcriptional target. SERPINE1 is a well-established promoter of tumor invasion and metastasis across malignancies like gastric cancer, triple-negative breast cancer, and colorectal cancer.^23^^,^^24^^,^^31^ In PDAC, SERPINE1 overexpression correlated with tumor progression and poor prognosis.^32^^,^^33^ While its pleiotropic oncogenic functions were recognized, its upstream regulation remained less defined. Our discovery that ARNTL2 directly bound and transactivated the SERPINE1 promoter established a novel regulatory link. This ARNTL2-SERPINE1 axis was functionally critical: SERPINE1 knockdown phenocopied ARNTL2 loss, and conversely, its forced expression rescued the migratory defect induced by ARNTL2 silencing, confirming its indispensable role as a downstream effector.

Beyond transcriptional regulation, ARNTL2-SERPINE1 signaling orchestrated a pro-metastatic EMT program. GSEA linked SERPINE1 to EMT and metastatic pathways, a connection validated by ARNTL2/SERPINE1-dependent modulation of canonical EMT markers and rescue experiments. This aligned with the established role of EMT in driving PDAC progression and dissemination.^34^^,^^35^^,^^36^ Recent studies have revealed enhanced EMT in KRAS G12D-mutant PDAC,^13^ while the potential mechanism was still unclear. Our findings showed that ARNTL2 might be a transcriptional activator implicated in amplifying pro-oncogenic signals downstream of the KRAS G12D oncogene, which offered a novel perspective on the regulatory network contributing to this aggressive phenotype. The clinical validation of correlative ARNTL2 and SERPINE1 protein expression in patient tissues solidified the translational relevance of the axis beyond cell line models, suggesting that these factors could serve as combinable prognostic biomarkers.

From a therapeutic perspective, while KRAS G12D-selective inhibitors have achieved promising preclinical and early clinical results,^16^^,^^17^^,^^18^ mechanisms of adaptive and acquired resistance were anticipated.^37^^,^^38^^,^^39^ Our work positioned the ARNTL2-SERPINE1 axis as a novel mediator of therapeutic resistance to direct KRAS G12D inhibition. We demonstrated that ARNTL2 or SERPINE1 overexpression significantly reduces zoldonrasib sensitivity in KRAS G12D-mutant PDAC cells, and that this resistance was reversible upon SERPINE1 knockdown. These findings nominate the ARNTL2-SERPINE1 axis as a parallel vulnerability for combination therapy: pharmacologically targeting this transcriptional node in conjunction with direct KRAS G12D inhibition might yield synergistic effects, forestall resistance, and expand therapeutic options for this prognostically unfavorable subtype.

In conclusion, our study unveiled a novel, clinically pertinent link that the circadian transcription factor ARNTL2 was elevated in KRAS G12D-mutant PDAC, which transcriptionally activated SERPINE1 to drive EMT and metastasis. This ARNTL2-SERPINE1 axis provided a mechanistic basis for the aggressively metastatic phenotype associated with KRAS G12D. Furthermore, the ARNTL2-SERPINE1 axis promoted resistance to selective KRAS G12D inhibition, positioning it as a promising target for combination therapeutic strategies.

### Limitations of the study

Several limitations of our study merited acknowledgment. First, the clinical conclusions regarding ARNTL2 and SERPINE1 should be interpreted as prognostic associations rather than predictive biomarkers. Although compelling within our retrospective tissue analysis, these findings ultimately require prospective validation in larger, multi-center clinical cohorts to establish their predictive utility. Second, the KRAS G12D specificity of the ARNTL2-SERPINE1 axis was inferred from comparative bioinformatic analyses and cell line correlations rather than causally demonstrated. We did not provide direct evidence that KRAS G12D itself mechanistically drives ARNTL2 upregulation, and the upstream signaling link between this oncogenic mutation and ARNTL2 transcription remains to be fully elucidated. Third, our metastasis models (tail-vein and intrasplenic injection assays) supported a role for ARNTL2 in survival and colonization at distant organ sites, but these approaches bypassed earlier stages of the metastatic cascade, including local invasion, intravasation, and circulation. Consequently, they were not sufficient to claim that ARNTL2 controls the complete metastatic process. Fourth, subcutaneous xenografts, while informative for tumor growth kinetics, did not fully recapitulate the pancreatic tumor microenvironment, including its characteristic desmoplastic stroma, immune infiltrate, and organ-specific paracrine signaling networks. As such, employing *in situ* models such as patient-derived organoids or genetically engineered mouse models with conditional ARNTL2 manipulation in a KRAS G12D-driven background would more faithfully recapitulate the native tumor microenvironment and allow for investigation of the role of ARNTL2 in earlier stages of tumorigenesis. Fifth, the mechanistic depth was bounded by our experimental design: we demonstrated ARNTL2 occupancy at the SERPINE1 promoter, mapped promoter activity via luciferase reporters, quantified expression changes, and performed genetic rescue experiments, yet these approaches did not capture higher-order regulatory mechanisms such as chromatin remodeling dynamics. Sixth, conclusions regarding EMT and cancer stemness were based largely on the expression of canonical protein markers and on spheroid size or number as functional readouts. While these data supported EMT and stemness-associated phenotypes, they did not establish lineage plasticity, mesenchymal-epithelial interconversion or tumor-initiating capacity. Finally, the therapeutic resistance findings were exploratory. We did not pharmacologically inhibit the ARNTL2-SERPINE1 axis, perform formal combination analyses, derive resistant cell line models, employ orthogonal viability readouts, or conduct *in vivo* treatment experiments. Therefore, the translational scope of these drug sensitivity data remained limited, and future studies incorporating pharmacologic targeting and clinically relevant treatment models will be essential to validate combinatorial therapeutic strategies.

## Resource availability

### Lead contact

Requests for further information and resources should be directed to and will be fulfilled by the lead contact, Gang Jin (jingang@smmu.edu.cn).

### Materials availability

This study did not generate new unique reagents.

### Data and code availability

• This paper analyzes existing, publicly available data, accessible at The Cancer Genome Atlas (TCGA) database (https://portal.gdc.cancer.gov/) (TCGA-PAAD, PanCancer Atlas).
• This paper does not report original code.
• Any additional information required to reanalyze the data reported in this paper is available from the lead contact upon request.

## Acknowledgments

This study was supported by Noncommunicable Chronic Diseases-10.13039/501100018537National Science and Technology Major Project (2025ZD0552300), the 10.13039/501100001809National Natural Science Foundation of China (82303496), the Shanghai Science and Technology Innovation Action Plan (23Y41900200), the 10.13039/100017950Shanghai Municipal Health Commission (20244Y0211), the Shanghai “Rising Stars of Medical Talents” Youth Development Program (SHWSRS(2026)_071), the Yangpu District Health Commission of Shanghai Municipality (YPQ202528), and the Navy Military Medical University (25TPSL0008).

## Author contributions

Conceptualization, X.S., S. Guo, and G.J.; methodology, X.S. and S. Guo; investigation, X.S., X.Y., S. Gao, X.W., H.W., B.W., X.X., K.Z., W.J., and G.L.; formal analysis, X.S., X.Y., and S. Gao; writing – original draft, X.S., X.Y., and S. Gao; writing – review and editing, X.S., X.Y., S. Gao, S. Guo, and G.J.; funding acquisition, X.S. and G.J.; project administration, S. Guo and G.J.; supervision, S. Guo and G.J.

## Declaration of interests

The authors declare no competing interests.

## STAR★Methods

### Key resources table

**Table:** 

REAGENT or RESOURCE	SOURCE	IDENTIFIER
**Antibodies**		

Anti-ARNTL2	Bioss	Cat# bs-11446 R; N/A
Anti-SERPINE1 (PAI-1)	Proteintech	Cat# 13801-1-AP; RRID: AB_2186881
Anti-E-cadherin	Cell Signaling Technology (CST)	Cat# 3195; RRID: AB_2291471
Anti-N-cadherin	Cell Signaling Technology (CST)	Cat# 13116; RRID: AB_2687616
Anti-Vimentin	Cell Signaling Technology (CST)	Cat# 5741; RRID: AB_10695459
Anti-Snail (SNAI1)	Cell Signaling Technology (CST)	Cat# 3879; RRID: AB_2255011
Anti-GAPDH	Proteintech	Cat# 60004-1-Ig; RRID: AB_2107436
HRP-conjugated secondary antibodies	Proteintech	N/A; N/A
Anti-CD44	Proteintech	Cat#60224-1-Ig; RRID: AB_11042767
Anti-SOX2	Proteintech	Cat#11064-1-AP; RRID: AB_2195801
Anti-NANOG	Proteintech	Cat#67255-1-Ig; RRID: AB_2882529

**Biological samples**		

Human pancreatic cancer tissue microarray	Shanghai Outdo Biotech Co., Ltd.	TMA
Human pancreatic cancer transcriptomic dataset	TCGA-PAAD	PanCancer Atlas
Xenograft tumor tissues	This study	N/A

**Chemicals, peptides, and recombinant proteins**		

RPMI-1640 medium	Gibco	Cat#11875093
DMEM, high glucose	Gibco	Cat#11965092
Fetal bovine serum	Gibco	Cat#10099141C
Penicillin-streptomycin	Gibco	Cat#15140122
Lipofectamine 3000	Invitrogen	Cat#L3000015
Puromycin	Sigma-Aldrich	Cat# P9620
TRIzol Reagent	Invitrogen	Cat#15596018
RIPA Lysis Buffer	Beyotime	Cat#P0013B
Protease and phosphatase inhibitors	Sigma-Aldrich	Cat# P8340
PVDF membranes	Millipore	N/A
Matrigel	BD Biosciences	Cat#356234
zoldonrasib	MedChemExpress	Cat#HY-156819

**Critical commercial assays**		

PrimeScript RT Reagent Kit	Takara	Cat# RR037A
TB Green Premix Ex Taq II	Takara	Cat# RR820A
BCA Protein Assay Kit	Thermo Fisher Scientific	Cat#23225
ECL Detection Kit	Thermo Fisher Scientific	Cat#32109
Cell Counting Kit-8 (CCK-8)	Dojindo Laboratories	Cat#CK04
Annexin V-FITC/PI Apoptosis Detection Kit	BD Biosciences	Cat# 556547
Magna ChIP HiSens Kit	Millipore	Cat# 17-10460
Dual-Luciferase Reporter Assay System	Promega	Cat#E1910

**Deposited data**		

TCGA-PAAD cohort, PanCancer Atlas	The Cancer Genome Atlas/GDC	https://portal.gdc.cancer.gov/

**Experimental models: Cell lines**		

Human: SW1990	ATCC	CRL-2172
Human: PANC-1	ATCC	CRL-1469
Human: AsPC-1	ATCC	CRL-1682
Human: BxPC-3	ATCC	CRL-1687
Human: Capan-1	ATCC	HTB-79
Human: MIAPaCa-2	ATCC	CRM-CRL-1420

**Experimental models: Organisms/strains**		

BALB/c nude mice, 4–6 weeks old	GemPharmatech Co., Ltd.	N/A

**Oligonucleotides**		

qPCR primers for ARNTL2	This study	Table S1
qPCR primers for SERPINE1	This study	Table S1
qPCR primers for CD44	This study	Table S1
qPCR primers for SOX2	This study	Table S1
qPCR primers for NANOG	This study	Table S1
ChIP-qPCR primers	This study	Table S3
shARNTL2 sequences	This study	Table S2
shSERPINE1 sequences	This study	Table S2
SERPINE1 promoter (ChIP-qPCR)	This study	Table S1

**Recombinant DNA**		

Lentiviral vectors targeting ARNTL2 and SERPINE1	GeneChem Co., Ltd.	N/A
ARNTL2 and SERPINE1 overexpression plasmids	GeneChem Co., Ltd.	N/A
pGL3-Basic vector	Promega	N/A
SERPINE1 promoter wild type and mutant reporter plasmids	This paper	N/A

**Software and algorithms**		

limma R package	Bioconductor	N/A
clusterProfiler R package	Bioconductor	N/A
GraphPad Prism 8.0	GraphPad Software	Version 8.0
QuPath software	QuPath	Version 0.4.x
ImageJ software	NIH	Version 1.48v
clusterProfiler R package (for GSEA)	Bioconductor	N/A

### Experimental model and study participant details

#### Human subjects and tissue specimens

Transcriptomic data and corresponding clinical information of pancreatic cancer patients were retrieved from The Cancer Genome Atlas (TCGA) database (https://portal.gdc.cancer.gov/) (TCGA-PAAD, PanCancer Atlas). Patients lacking RNA-sequencing data (*n* = 7) or missing OS information (*n* = 2) were excluded. Consequently, a total of 175 eligible patients were included in the final analysis. The cohort (95 males and 80 females, aged 35–88 years) was stratified into KRAS G12D mutant (*n* = 45) and non-G12D (*n* = 130) groups, which comprised predominantly patients of European ancestry (*n* = 149), African ancestry (*n* = 5), East Asian ancestry (*n* = 11), Admixed American ancestry (*n* = 4) and other/unknown (*n* = 6).

A pancreatic cancer TMA containing paired tumor and adjacent normal tissues was purchased from Shanghai Outdo Biotech Co., Ltd. (Shanghai, China). All tissues were collected under ethical standards with donors being completely informed and providing written consent. The cohort comprised 103 males and 97 females, aged 33–80 years, and all individuals were of East Asian ancestry. This study was approved by the Ethics Committee of Changhai Hospital (CHEC2018-112).

#### Cell lines

The human pancreatic cancer cell lines (SW1990, PANC-1, AsPC-1, BxPC-3, Capan-1, MIAPaCa-2) were obtained from the American Type Culture Collection (Manassas, VA, USA). All human cell lines have been authenticated using STR profiling within the last three years and were routinely tested for mycoplasma contamination. Cells were cultured in DMEM or RPMI-1640 medium (Gibco, Grand Island, NY, USA) supplemented with 10% fetal bovine serum (Gibco) and 1% penicillin-streptomycin (Gibco) at 37°C in a humidified incubator containing 5% CO_2_. Information regarding the sex of the original donors of the established cell lines was not considered in the experimental design.

#### Animals

Male BALB/c nude mice (4–6 weeks old) were purchased from GemPharmatech Co., Ltd. (Shanghai, China). Mice were maintained under specific pathogen-free (SPF) conditions at 22 ± 2°C with a 12-h light/12-h dark cycle and had free access to food and water. Animals were acclimatized for at least one week prior to experimentation. Mice were randomly assigned to experimental groups. All animal experiments were approved by the Ethics Committee of Changhai Hospital (CHEC2018-112) and were performed in accordance with institutional guidelines for the care and use of laboratory animals.

Male mice were specifically chosen to eliminate potential sex-related hormonal influences on tumor growth, as previous studies have shown that estrogen can affect pancreatic cancer progression. Sex as a biological variable was not considered a confounding factor in this study.

### Method details

#### Bioinformatics analysis

Transcriptomic data and corresponding clinical information from the TCGA-PAAD cohort were downloaded from the TCGA database. Differential gene expression analysis between KRAS G12D-mutant and non-G12D pancreatic cancer samples was performed using the “limma” R package. Gene Set Enrichment Analysis (GSEA) was conducted using the “clusterProfiler” R package to identify biological pathways associated with ARNTL2 expression and KRAS G12D-related transcriptional signatures.

#### Lentiviral transfection and cell line construction

To generate stable knockdown or overexpression models, lentiviral vectors targeting ARNTL2 (shARNTL2) and SERPINE1 (shSERPINE1), as well as overexpression constructs (ARNTL2 and SERPINE1) and their corresponding controls (shNC and Vector), were synthesized by GeneChem Co., Ltd. (Shanghai, China). For overexpression studies, the full-length human ARNTL2 or SERPINE1 coding sequence was cloned into the pCDH lentiviral expression vector. For knockdown studies, shRNA sequences targeting human ARNTL2 or SERPINE1 were cloned into pLKO.1-based lentiviral vectors. Recombinant lentiviruses were packaged and supplied by GeneChem Co., Ltd.

Cells were infected with lentiviral particles according to the manufacturer’s instructions. Stable cell lines were established by selection with puromycin (2 μg/mL; Sigma-Aldrich, Cat#P9620) for two weeks. The efficiency of gene knockdown or overexpression was confirmed by quantitative real-time PCR (qPCR) and western blot analysis. The sequences of the qPCR primers and shRNAs used in this study are provided in Tables S1 and S2, respectively.

#### RNA extraction and qPCR

Total RNA was extracted using the TRIzol Reagent (Invitrogen). cDNA was synthesized using the PrimeScript™ RT Reagent Kit (Cat# RR037A, Takara, Shiga, Japan). qPCR was performed using TB Green® Premix Ex Taq™ II (Cat# RR820A, Takara) on an ABI 7500 Real-Time PCR System (Applied Biosystems). Relative gene expression was calculated using the 2−ΔΔCt method, normalized to GAPDH. Primer sequences are listed in Table S1.

#### Western blot analysis

Total protein was extracted using RIPA Lysis Buffer (Beyotime, Shanghai, China) containing protease and phosphatase inhibitors (Sigma-Aldrich). Protein concentration was determined using a BCA Protein Assay Kit (Thermo Fisher Scientific). Proteins were separated by SDS-PAGE and transferred onto PVDF membranes (Millipore). After blocking with 5% non-fat milk, membranes were incubated with primary antibodies overnight at 4°C, followed by HRP-conjugated secondary antibodies (1:5000) (Proteintech). Bands were visualized using an ECL Detection Kit (Thermo Fisher Scientific).

The primary antibodies used in this study were as follows:

Anti-ARNTL2: 1:1000 dilution (bs-11446 R, Bioss);

Anti-SERPINE1 (PAI-1): 1:1000 dilution (Cat No. 13801-1-AP, Proteintech);

Anti-E-cadherin: 1:1000 dilution (Cat# 3195, Cell Signaling Technology [CST]);

Anti-N-cadherin: 1:1000 dilution (Cat# 13116, CST);

Anti-Vimentin: 1:1000 dilution (Cat# 5741, CST);

Anti-Snail (SNAI1): 1:1000 dilution (Cat# 3879, CST);

Anti-CD44: 1:5000 dilution (Cat# 60224-1-Ig, Proteintech);

Anti-SOX2: 1:500 dilution (Cat# 11064-1-AP, Proteintech);

Anti-NANOG: 1:5000 dilution (Cat# 67255-1-Ig, Proteintech);

Anti-GAPDH: 1:5000 dilution (Cat# 60004-1-Ig, Proteintech).

#### Cell proliferation and apoptosis assays

Cell proliferation was assessed using the Cell Counting Kit-8 (CCK-8) (Dojindo Laboratories, Kumamoto, Japan). Cells were seeded into 96-well plates (2×10^3^ cells/well), and absorbance at 450 nm was measured at indicated time points using a microplate reader.

For apoptosis analysis, cells were stained using the Annexin V-FITC/PI Apoptosis Detection Kit (Cat# 556547, BD Biosciences, San Jose, CA, USA) and analyzed by flow cytometry (BD FACSCanto II).

#### Wound healing and transwell assays

For wound healing assays, a scratch was created in a confluent cell monolayer using a 200 μL pipette tip. Images were captured at 0 h and 24 h.

Transwell assays were performed using 8-μm pore chambers (Corning, NY, USA). For migration, cells were seeded in the upper chamber without Matrigel. For invasion, the upper chamber was pre-coated with Matrigel (BD Biosciences). Medium containing 10% FBS was added to the lower chamber as a chemoattractant. After 24 h, migrated/invaded cells were stained with 0.1% crystal violet and counted under a microscope.

#### 3D tumor spheroid formation assay

Three-dimensional (3D) tumor spheroid formation assays were performed to evaluate spheroid-forming capacity and stemness-associated characteristics of pancreatic cancer cells. Briefly, PANC-1, BxPC-3 and SW1990 cells with the indicated genetic modifications were dissociated into single-cell suspensions and seeded into ultra-low-attachment culture plates. Cells were cultured in complete growth medium supplemented with 2.5% Matrigel (Corning, Cat#356234) under non-adherent conditions.

For knockdown and rescue experiments, cells were assigned to shNC, shARNTL2, shSERPINE1, and shARNTL2+SERPINE1 groups. For overexpression and reversal experiments, cells were assigned to Vector, ARNTL2, SERPINE1, and ARNTL2+shSERPINE1 groups.

Cells were maintained at 37°C in a humidified incubator containing 5% CO_2_. Fresh medium was replenished every 2–3 days. After 7 days of culture, spheroid formation was examined under an inverted microscope. The number and diameter of tumor spheroids were quantified from randomly selected fields using ImageJ software (NIH). Each experiment was performed in triplicate.

#### Cycloheximide chase assay

To assess SERPINE1 protein stability, cycloheximide (CHX) chase assays were performed. PANC-1 and SW1990 cells with stable ARNTL2 knockdown and BxPC-3 cells with ARNTL2 overexpression, together with their corresponding control cells, were seeded into 6-well plates and cultured until approximately 70–80% confluence. Cells were treated with cycloheximide (CHX; Sigma-Aldrich, Cat#C7698) at a final concentration of 100 μg/mL to block *de novo* protein synthesis and harvested at the indicated time points (0, 4, 8, and 12 h). Total protein was extracted using RIPA lysis buffer supplemented with protease inhibitors, and SERPINE1 protein levels were examined by western blotting. GAPDH was used as the loading control. SERPINE1 protein abundance at each time point was normalized to GAPDH and quantified relative to the corresponding 0 h sample.

#### Chromatin immunoprecipitation (ChIP) assay

ChIP assays were performed using the Magna ChIP™ HiSens Kit (Cat# 17-10460, Millipore) according to the manufacturer’s instructions. Chromatin was immunoprecipitated with the anti-ARNTL2 antibody or IgG control. The enrichment of specific DNA fragments (SERPINE1 promoter region) was analyzed by qPCR.

#### Dual-luciferase reporter assay

To determine the ARNTL2-responsive region within the human SERPINE1 promoter, a full-length SERPINE1 promoter fragment and four truncated promoter fragments were amplified and cloned into the pGL3-Basic luciferase reporter vector. The full-length SERPINE1 promoter reporter construct contained an approximately 2.0-kb upstream regulatory region, whereas Promoter-1, Promoter-2, Promoter-3, and Promoter-4 represented four consecutive truncated fragments of the SERPINE1 promoter, each approximately 500 bp in length. These constructs were used to map the promoter region responsible for ARNTL2-mediated transcriptional activation. Detailed sequences of all promoter constructs are provided in Table S3.

For luciferase reporter assays, PANC-1 and SW1990 cells were seeded into 24-well plates and co-transfected with the indicated SERPINE1 promoter reporter plasmids together with either an ARNTL2 overexpression plasmid or the corresponding empty vector control. The Renilla luciferase plasmid pRL-TK was co-transfected as an internal normalization control. Forty-eight hours after transfection, cells were lysed, and firefly and Renilla luciferase activities were measured using the Dual-Luciferase Reporter Assay System according to the manufacturer’s instructions. Relative luciferase activity was calculated by normalizing firefly luciferase activity to Renilla luciferase activity. Each experiment was performed in triplicate.

#### Drug sensitivity assay

To determine whether the ARNTL2/SERPINE1 axis influences the response of pancreatic cancer cells to KRAS G12D inhibition, cell sensitivity to zoldonrasib (RMC-9805; MedChemExpress, Cat#HY-156819) was assessed using the Cell Counting Kit-8 (CCK-8) assay. PANC-1 and SW1990 cells with the indicated genetic modifications were seeded into 96-well plates at a density of 2 × 10^3^ cells per well and allowed to adhere overnight. Cells were subsequently treated with increasing concentrations (ranging from 0.1 nmol/L to 100 nmol/L, with three technical replicates per concentration) of zoldonrasib for 72 h.

Following drug treatment, 10 μL of CCK-8 reagent (Dojindo, Cat#CK04) was added to each well and incubated at 37°C for 2 h. Absorbance at 450 nm was measured using a microplate reader. Cell viability was normalized to untreated control cells. Dose-response curves were generated using GraphPad Prism 8.0 software, and the half-maximal inhibitory concentration (IC50) values were calculated by nonlinear regression analysis. All experiments were performed independently in triplicate.

#### Animal studies

Subcutaneous Xenograft: Mice were injected subcutaneously with 5×10^6^ transfected cells. Tumor volume (V = 0.5×Length×Width^2^) was monitored every 4 days.

Lung/Liver Metastasis: Cells (2×10^6^) were injected into the tail vein (lung metastasis) or spleen capsule (liver metastasis). Metastatic burden was monitored using the IVIS Spectrum Imaging System (PerkinElmer) following luciferin injection. Bioluminescence imaging was initiated 7 days after cell injection and subsequently performed weekly for 6 weeks. Images at day 21 post-injection are shown in Figure 3.

#### Immunohistochemistry (IHC) and tissue microarray (TMA)

Tumor tissues from xenografts and TMA slides were deparaffinized, rehydrated, and subjected to antigen retrieval. Slides were incubated with primary antibodies against ARNTL2 (1:100), SERPINE1 (1:100), and Ki-67 (1:200) overnight at 4°C, followed by secondary antibody incubation and DAB staining.

The stained slides were digitized, and protein expression was quantitatively analyzed using QuPath software. The expression levels were evaluated based on the H-score or the percentage of positive cells.

### Quantification and statistical analysis

Data are presented as mean ± standard deviation. Statistical analyses were performed using GraphPad Prism 8.0 software. Differences between two groups were analyzed using Student’s *t* test or Wilcoxon rank-sum test. Multiple groups were compared using One-way ANOVA. Survival curves were generated using the Kaplan-Meier method and compared via the Log rank test. A *p*-value <0.05 was considered statistically significant.

## Supplemental information

Document S1. Figures S1–S10 and Tables S1–S3

Data S1. Original images of western-blot experiments

Data S2. Unedited microscopy images

Data S3. Sequence (5'→3′)
