# Supplementary material for: ARNTL2 promotes metastatic phenotypes through SERPINE1 transcription in KRAS G12D pancreatic cancer
Source: iScience. 2026 Sep 8;29(9):117479. doi: 10.1016/j.isci.2026.117479 (PMC13573702; doi:10.1016/j.isci.2026.117479)
Supplement: Supplementary file 1 [file mmc1.pdf]

iScience, Volume *29*

Supplemental information

ARNTL2 promotes metastatic phenotypes through SERPINE1 transcription in KRAS G12D pancreatic cancer

Xiaohan Shi, Xiaoyi Yin, Suizhi Gao, Xinqian Wu, Huan Wang, Beilei Wang, Xiongfei Xu, Kailian Zheng, Wei Jing, Gang Li, Shiwei Guo, and Gang Jin

Supplemental Figure

Figure S1

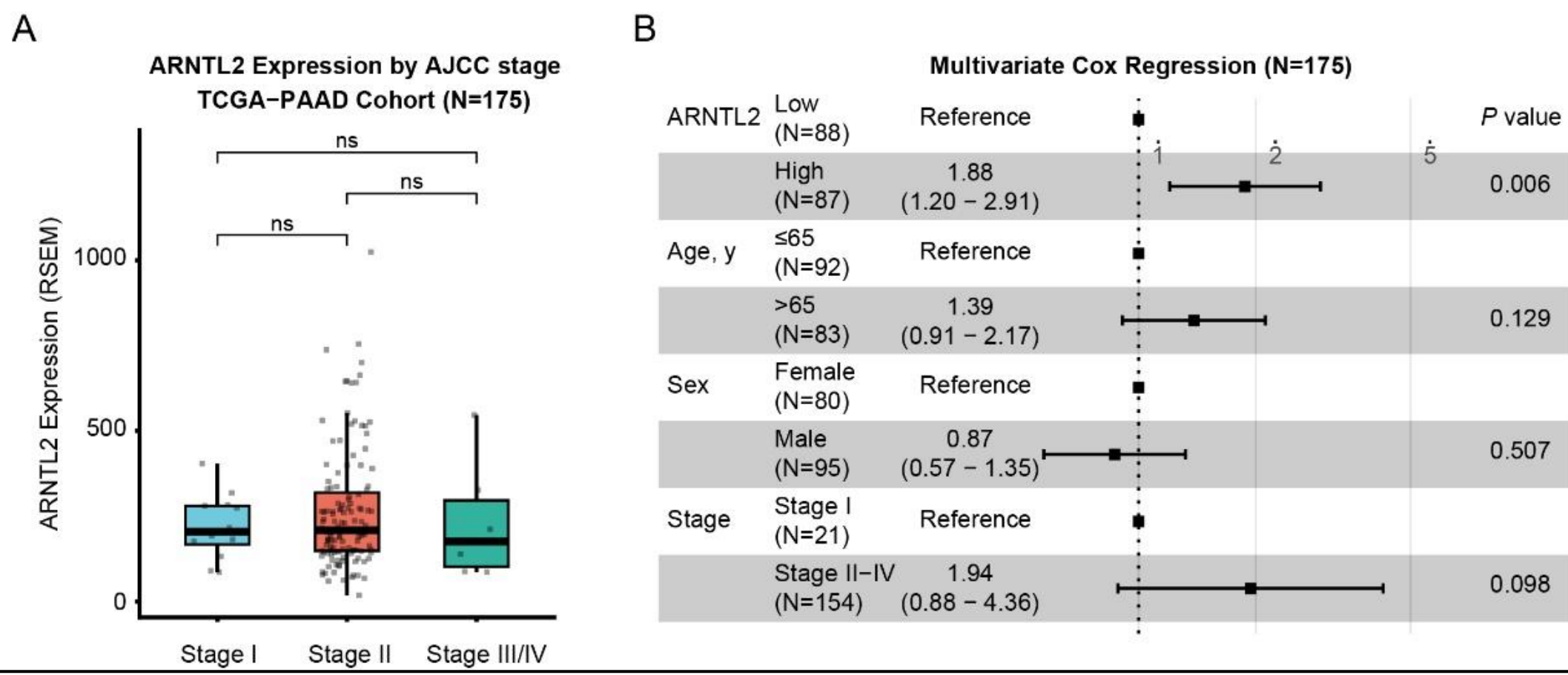

Figure S1. ARNTL2 expression across clinical stages and its independent prognostic value in the TCGA-PAAD cohort.

(A) Boxplots illustrating the mRNA expression levels of ARNTL2 stratified by American Joint Committee on Cancer (AJCC) clinical stages (Stage I, Stage II, and Stage III/IV). Data were retrieved from the TCGA-PAAD cohort (n=175). The expression values are presented as RSEM normalized counts. Box plots show the median (center line), interquartile range (box), and 1.5×IQR (whiskers). Individual data points are overlaid to show sample distribution. n represents number of biologically independent tumor samples from distinct patients.

(B) Forest plot summarizing the results of a multivariate Cox proportional hazards regression analysis (n=175). The analysis evaluated the independent prognostic significance of ARNTL2 expression (High vs Low) for overall survival, adjusting for clinicopathological covariates including age (≤65 vs >65 years), sex (Female vs Male), and tumor stage (Stage I vs Stage II–IV). The square markers represent the hazard ratio (HR), and the horizontal lines indicate the 95% confidence interval (CI). The reference group for each variable is indicated on the left. n represents number of distinct patients.

Statistical analysis: In (A), comparisons among the three stage groups were performed using one-way ANOVA followed by Tukey's post-hoc test. In (B), P values, HRs, and 95% CIs were derived from the multivariate Cox regression model. Exact P values are displayed in the figure panel. ns for not significant.

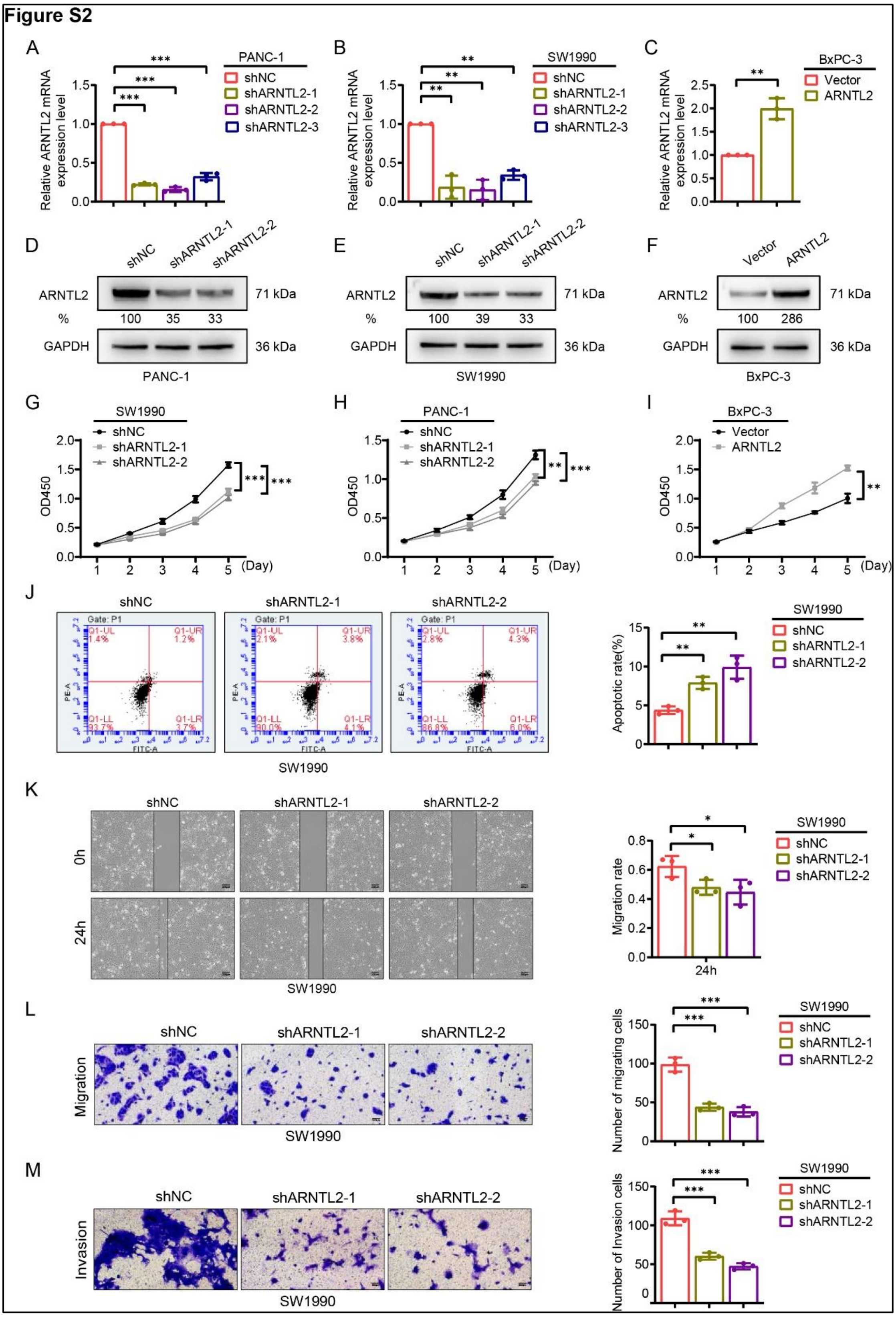

Figure S2. Validation of ARNTL2 transfection efficiency and its effects on proliferation, apoptosis, and motility in pancreatic cancer cells.

(A-C) Quantitative real-time PCR analysis of ARNTL2 mRNA expression. (A) PANC-1 and (B) SW1990 cells were transfected with three independent short hairpin RNAs

targeting ARNTL2 (shARNTL2-1, -2, -3) or a negative control (shNC). (C) BxPC-3 cells were transfected with an ARNTL2 overexpression vector or an empty vector. Expression levels were normalized to GAPDH.

(D-F) Western blot analysis confirming ARNTL2 protein abundance. (D) PANC-1 and (E) SW1990 cells transfected with shNC, shARNTL2-1, or shARNTL2-2. (F) BxPC-3 cells transfected with Vector or ARNTL2 plasmid. GAPDH was used as the loading control. Molecular weights (kDa) are indicated. Numbers underneath the blots indicate relative band intensities after normalization against loading controls with shNC or Vector set as 100%.

(G-I) Cell proliferation assessment using the CCK-8 assay. Viability curves of (G) SW1990 and (H) PANC-1 cells with ARNTL2 knockdown, and (I) BxPC-3 cells with ARNTL2 overexpression were generated by measuring absorbance at 450 nm (OD450) daily for 5 days.

(J) Flow cytometric analysis of apoptosis in SW1990 cells. Cells transfected with shNC, shARNTL2-1, or shARNTL2-2 were stained with Annexin V-FITC and PI (PE channel). Representative quadrant dot plots are shown on the left, and the percentage of total apoptotic cells (sum of Q1-UR and Q1-LR quadrants) is quantified in the bar graph on the right.

(K) Wound healing assay examining the lateral motility of SW1990 cells. Representative images of the scratch area were captured at 0 h and 24 h (left). Histograms quantify the migration rate calculated as the percentage of wound closure relative to the 0 h baseline (right). Scale bars represent 200 μm

(L and M) Transwell assays evaluating cell motility. (L) Migration assay and (M) Matrigel invasion assay were performed on SW1990 cells following ARNTL2 knockdown. Cells were seeded into the upper chamber without Matrigel and allowed to migrate for 24 h. Representative micrographs of crystal violet-stained cells on the lower surface of the membrane are displayed (left). Histograms display the average number of migrated cells counted in five random fields (right). Scale bars represent 50 μm.

Statistical analysis: Representative images derive from three biologically independent experiments. Data are presented as mean ± SD from three biologically independent experiments with individual data points shown. Proliferation curves (G–I) were analyzed using two-way ANOVA. Statistical significance was determined using one-way ANOVA followed by Tukey's post-hoc test for multiple comparisons (A, B, D, E, J–M) or the two-tailed Student's t-test for two-group comparisons (C, F). * $P<0.05$, ** $P<0.01$, *** $P<0.001$.

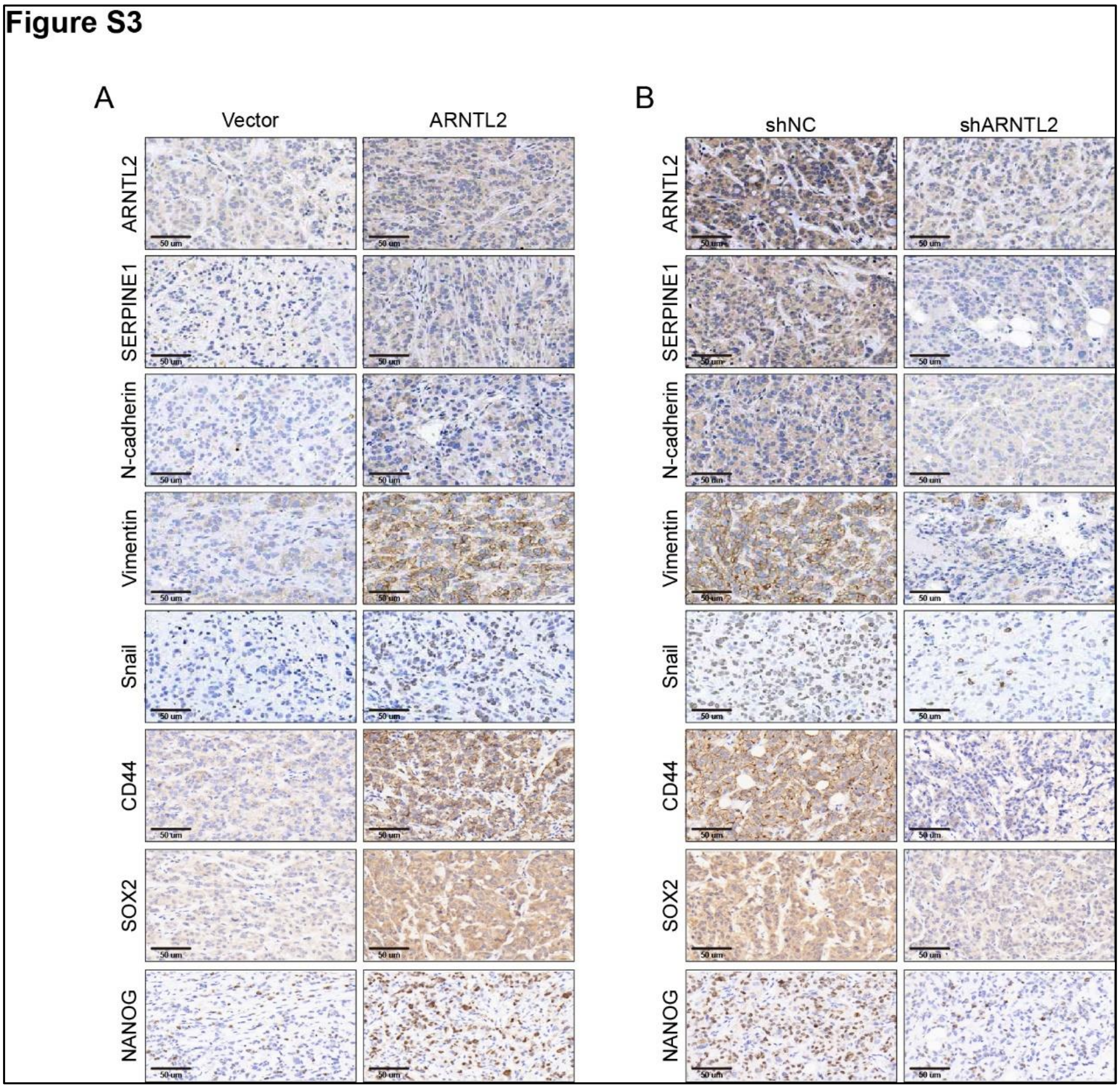

Figure S3. Immunohistochemical evaluation of ARNTL2, SERPINE1, EMT markers and cancer stemness markers in pancreatic cancer xenograft tissues.

Representative immunohistochemistry (IHC) micrographs showing protein expression patterns in subcutaneous xenograft tumors derived from nude mice. Serial sections were stained for ARNTL2, SERPINE1, epithelial-mesenchymal transition (EMT) markers (N-cadherin, Vimentin and Snail) and cancer stemness markers (CD44, SOX2 and NANOG).

(A) IHC staining of tumor tissues derived from BxPC-3 cells. Mice were injected with cells stably transfected with either an empty vector (Vector) or an ARNTL2 overexpression plasmid (ARNTL2). Compared to the Vector group, the ARNTL2 group exhibits elevated staining intensity for ARNTL2, SERPINE1, the EMT markers and the cancer stemness markers.

(B) IHC staining of tumor tissues derived from PANC-1 cells. Mice were injected with cells transfected with a negative control shRNA (shNC) or an shRNA targeting ARNTL2 (shARNTL2). The shARNTL2 group displays reduced immunoreactivity for ARNTL2,

SERPINE1, the EMT markers and cancer stemness markers compared to the shNC group.

Scale bars represent 50 μm. Representative images derive from four biologically independent experiments.

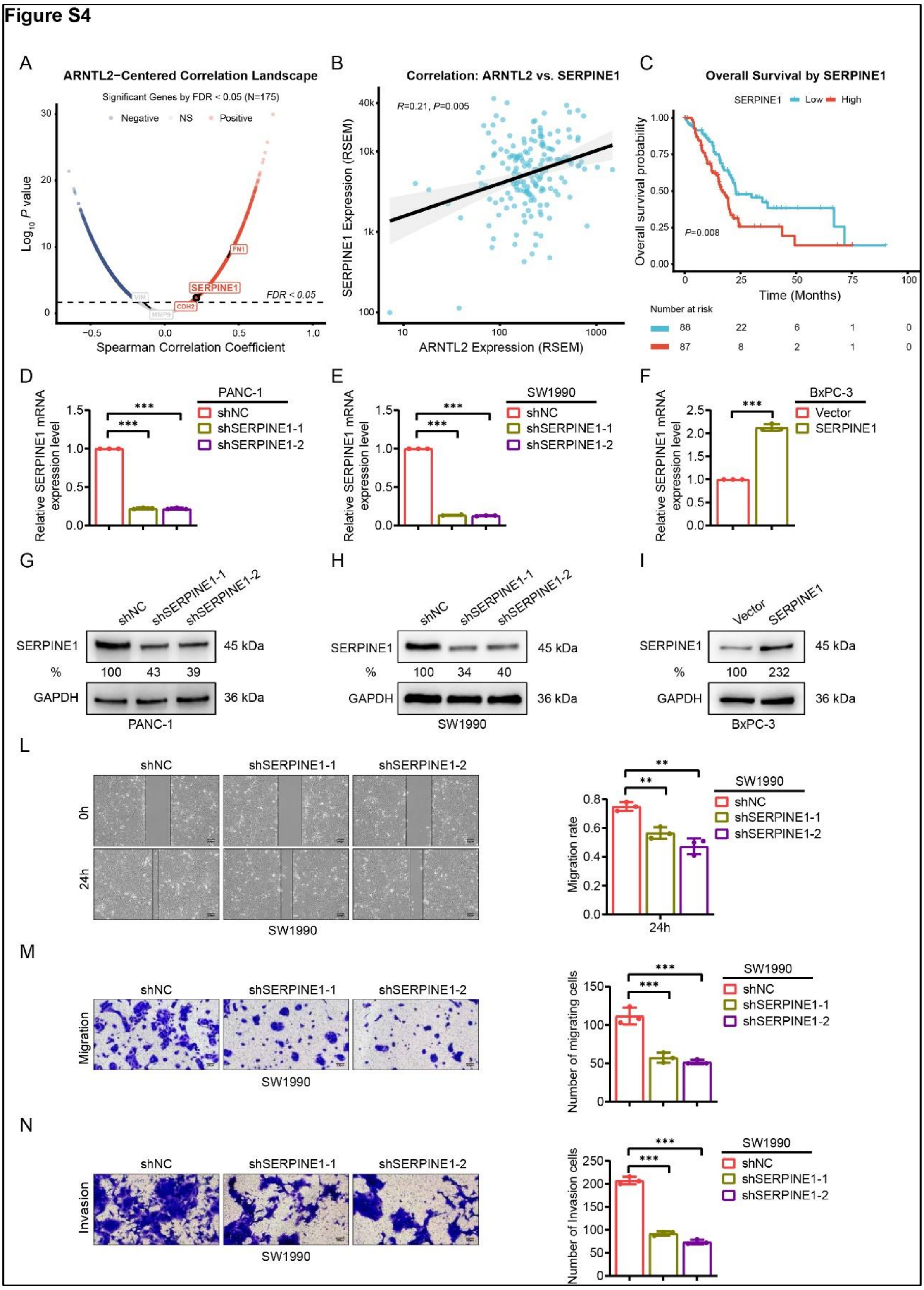

Figure S4. Correlation of ARNTL2 with SERPINE1, verification of transfection efficiency, and functional analysis of SERPINE1 knockdown in pancreatic cancer cells.

(A–C) Bioinformatics analysis based on the TCGA-PAAD cohort (PanCancer Atlas, n=175). n represents number of biologically independent tumor samples from distinct patients or number of distinct patients. (A) Correlation landscape visualizing genes co-

expressed with ARNTL2. The x-axis represents the Spearman correlation coefficient, and the y-axis represents statistical significance (-Log10 P value). SERPINE1 is highlighted as the positively correlated gene (red). (B) Scatter plot showing the positive correlation between ARNTL2 and SERPINE1 mRNA expression. The linear regression line, Spearman's correlation coefficient (R), and P value are indicated. (C) Kaplan-Meier survival analysis comparing overall survival between patients with high and low SERPINE1 expression. The numbers of patients at risk at each time point are listed below the curves.

(D–I) Validation of SERPINE1 knockdown and overexpression efficiency. (D and F) Quantitative real-time PCR (qPCR) analysis of SERPINE1 mRNA levels in (D) PANC-1 and (F) SW1990 cells transfected with shNC or two independent shRNAs targeting SERPINE1 (shSERPINE1-1, -2). (H) qPCR analysis of SERPINE1 mRNA levels in BxPC-3 cells transfected with an empty vector or a SERPINE1 overexpression plasmid. (E, G, and I) Western blot analysis confirming SERPINE1 protein abundance in (E) PANC-1, (G) SW1990, and (I) BxPC-3 cells corresponding to the treatments described above. GAPDH was used as the loading control. Numbers underneath the blots indicate relative band intensities after normalization against loading controls with shNC or Vector set as 100%.

(L–N) Evaluation of cell motility following SERPINE1 knockdown in SW1990 cells. (L) Wound healing assay. Representative images of the scratch area were captured at 0 h and 24 h (left). Histograms quantify the migration rate calculated as the percentage of wound closure relative to the 0 h baseline (right). Scale bars represent 200 μm. (M) Transwell migration assay. Cells were seeded into the upper chamber without Matrigel and allowed to migrate for 24 h. Representative micrographs of crystal violet-stained cells on the lower surface of the membrane are shown under the indicated transfection conditions (left). Histograms display the average number of migrated cells counted in five random fields (right). Scale bars represent 500 μm. (N) Matrigel invasion assay. The invasive capacity was evaluated following the same staining and quantification protocols as the migration assay. Scale bars represent 500 μm.

Statistical analysis: Representative images derive from three biologically independent experiments. Data for functional assays and expression validation are presented as mean ± SD from three biologically independent experiments with individual data points shown. Comparisons involving two groups (H) were analyzed using the two-tailed Student's t-test. Comparisons among multiple groups (D, F, L–N) were performed using one-way ANOVA followed by Tukey's post-hoc test. Survival significance (C) was determined by the log-rank test. Correlation (A, B) was assessed using Spearman's rank correlation. ** P<0.01, *** P<0.001.

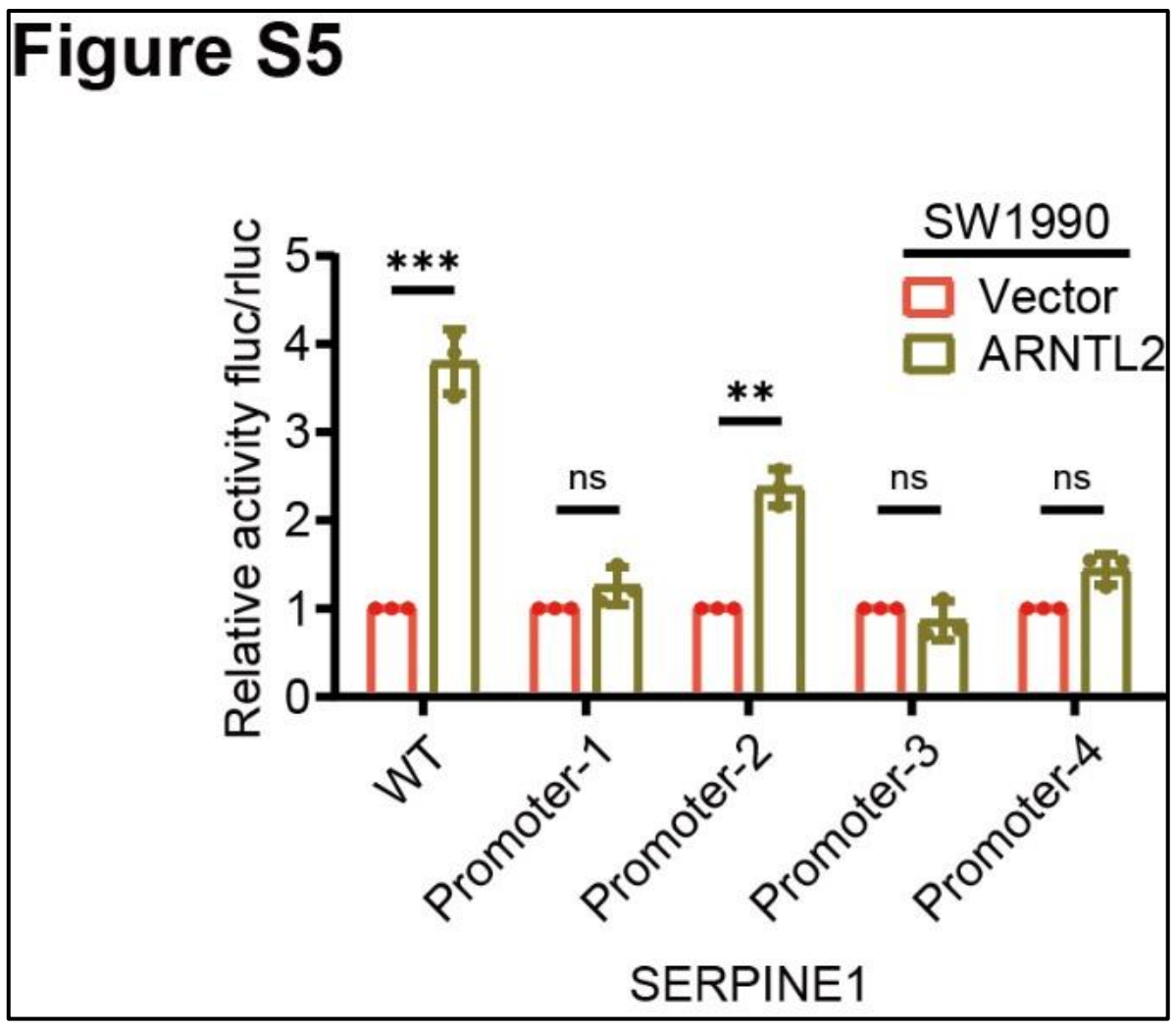

Figure S5. Dual-luciferase reporter assays of ARNTL2 transcriptionally regulating SERPINE1 expression in SW1990 cells.

Cells were co-transfected with an ARNTL2 expression plasmid (or vector) and luciferase reporter constructs containing either the SERPINE1 wild type (WT) promoter or truncated promoter fragments (Promoter-1 to Promoter-4). Relative luciferase activity (Renilla/Firefly) was measured 48 hours post-transfection. Data are presented as mean ± SD from three biologically independent experiments with individual data points shown. P values were calculated using the two-tailed Student's t-test. ** P<0.01, *** P<0.001, ns for not significant.

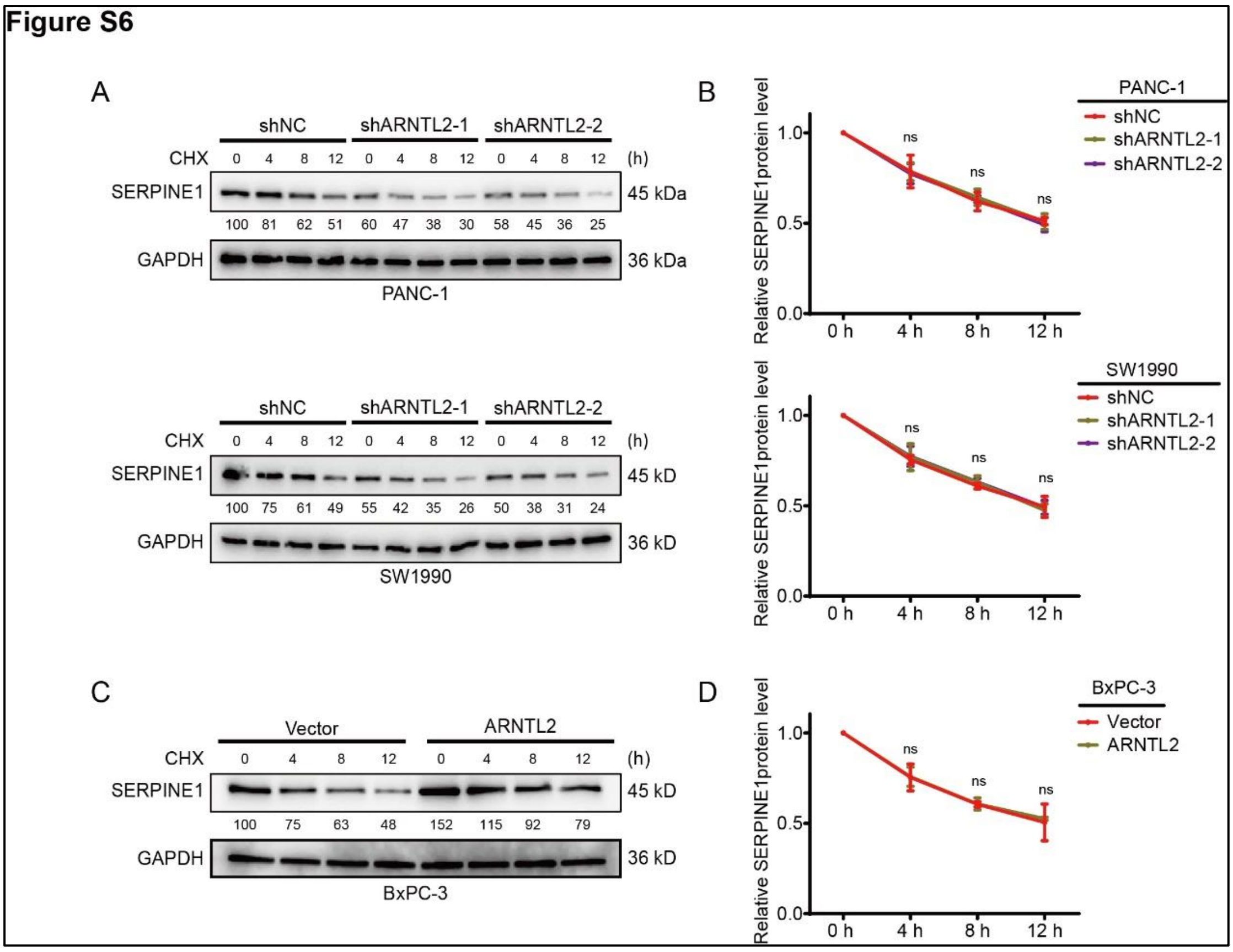

Figure S6. Cycloheximide chase assays demonstrating that ARNTL2 did not alter SERPINE1 protein stability.

(A) PANC-1 and SW1990 cells were transfected with negative control shRNA (shNC) or two independent shRNAs targeting ARNTL2 (shARNTL2-1 and shARNTL2-2), treated with cycloheximide (CHX, 100 μg/mL) to block de novo protein synthesis, and harvested at the indicated time points (0, 4, 8, and 12 h). SERPINE1 protein levels were assessed by western blotting, and GAPDH served as the loading control. Numbers underneath the blots indicate relative band intensities after normalization against GAPDH with the corresponding 0 h sample set as 100%; (B) Quantification of relative SERPINE1 protein levels over time in PANC-1 and SW1990 cells with ARNTL2 knockdown. (C) BxPC-3 cells were transfected with an empty vector (Vector) or an ARNTL2 overexpression plasmid (ARNTL2), subjected to CHX treatment, and analyzed as in (A). (D) Quantification of relative SERPINE1 protein levels in BxPC-3 cells with ARNTL2 overexpression. Data are presented as mean ± SD from three biologically independent experiments with individual data points shown. Statistical significance was determined using two-way ANOVA followed by Tukey's post-hoc test for multiple comparisons (B) or the two-tailed Student's t-test (D). ns for not significant.

Figure S7

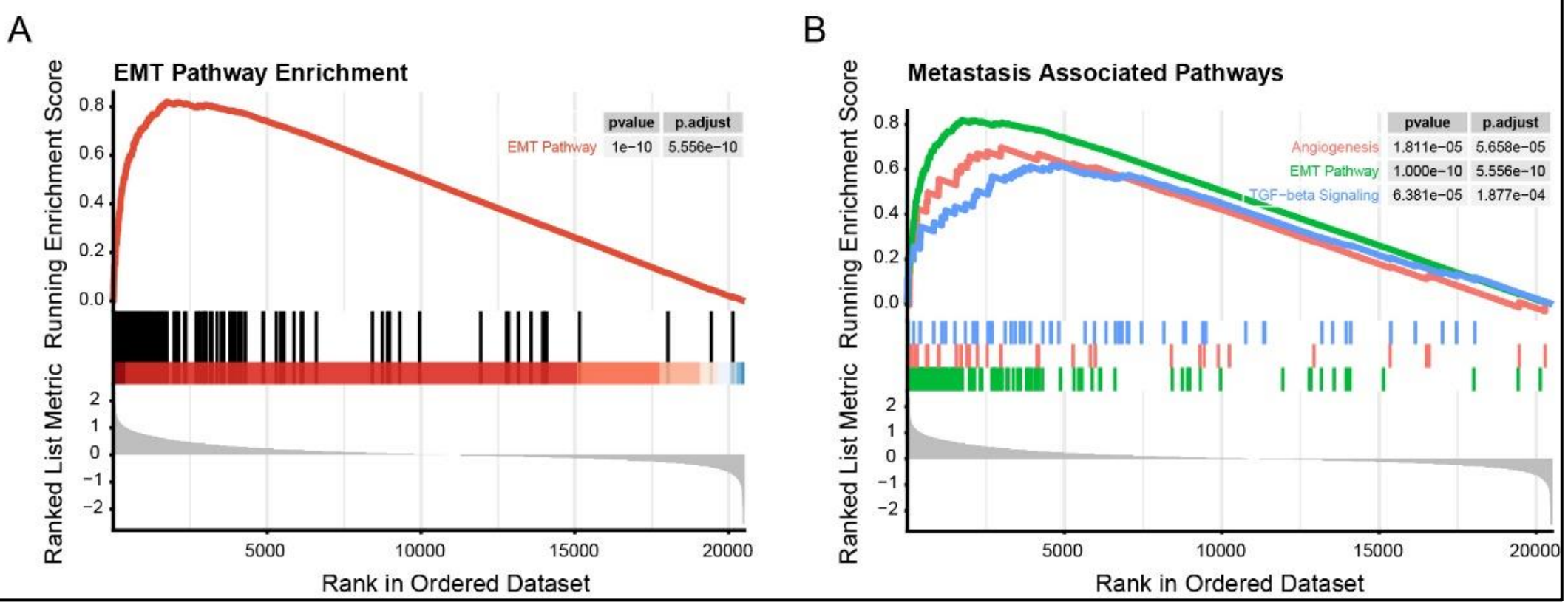

Figure S7. Gene Set Enrichment Analysis plots identifying biological pathways enriched in the ARNTL2-high expression phenotype within the TCGA-PAAD cohort.

(A) Enrichment plot for the “EMT Pathway” gene set. The upper curve (green) represents the running enrichment score, while the vertical black bars indicate the position of gene set members within the ranked list of differentially expressed genes. (B) Multi-pathway GSEA plot summarizing the enrichment of metastasis-associated signatures. The curves depict the enrichment scores for Angiogenesis (red), EMT Pathway (green), and TGF-beta Signaling (blue). The associated nominal P values and adjusted P values are provided in the inset tables.

Statistical analysis: Statistical significance was determined using the permutation test embedded in the GSEA software; P values and false discovery rate are displayed.

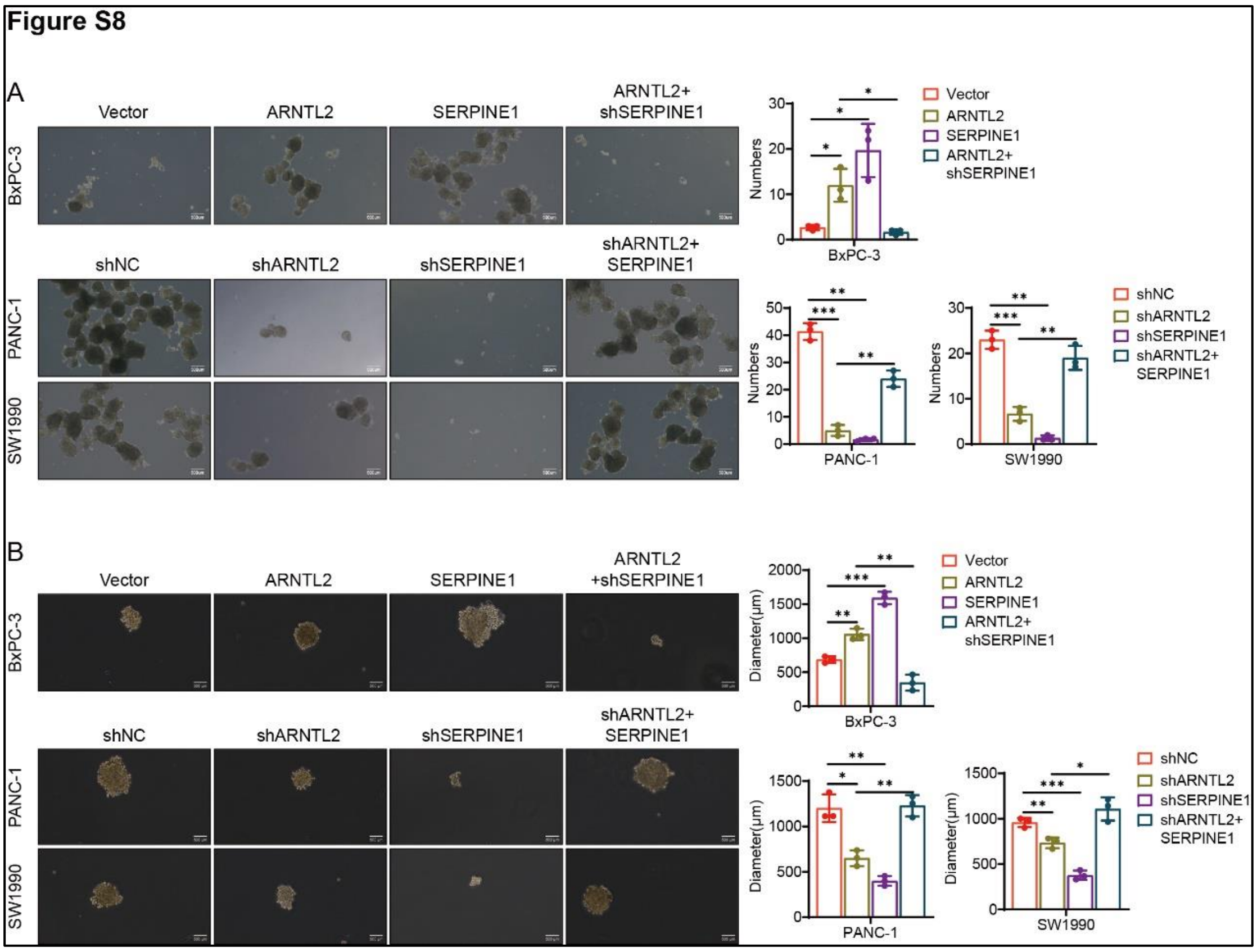

Figure S8. Tumor spheroid formation assays evaluating the cancer stemness-promoting function of the ARNTL2-SERPINE1 axis.

Representative images of spheroids are shown on the left, and quantifications are shown on the right. (A) Quantification of spheroid number. (B) Quantification of spheroid diameter. BxPC-3 cells were assigned to Vector, ARNTL2, SERPINE1 and ARNTL2+shSERPINE1 groups. PANC-1 and SW1990 cells were assigned to shNC, shARNTL2, shSERPINE1 and shARNTL2+SERPINE1 groups. Scale bars represent 500 μm.

Statistical analysis: Representative images derive from three biologically independent experiments. Data are presented as mean ± SD from three biologically independent experiments with individual data points shown. Statistical significance between groups was determined using one-way ANOVA followed by Tukey's post-hoc test. * $P<0.05$, ** $P<0.01$, *** $P<0.001$.

Figure S9

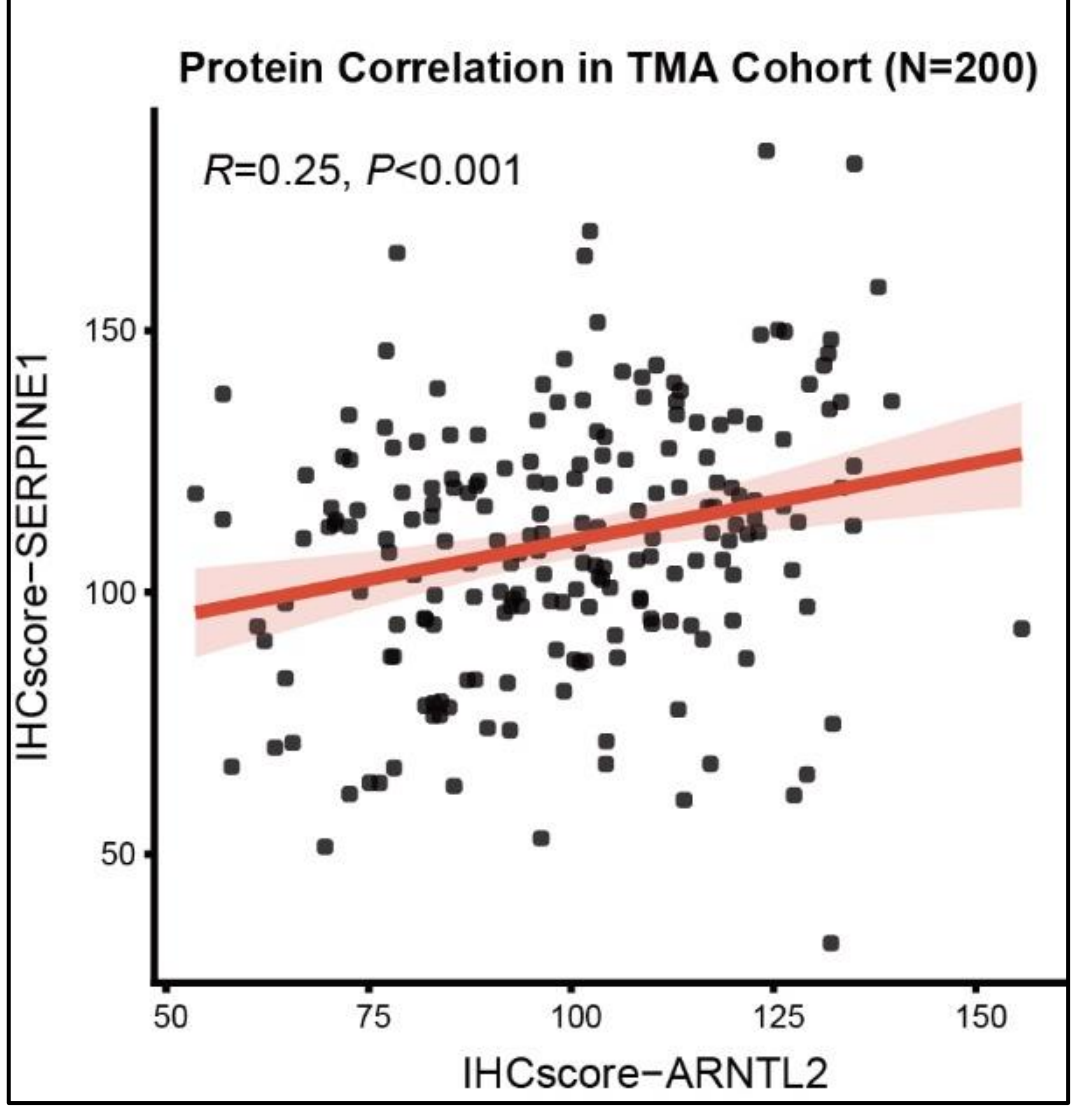

Figure S9. Protein level correlation of ARNTL2 and SERPINE1 in TMA cohort of pancreatic cancer.

Scatter plot analysis of protein expression correlation in the tissue microarray validation cohort (n=200). The graph illustrates the relationship between the immunohistochemical staining scores of ARNTL2 (x-axis) and SERPINE1 (y-axis) in tumor tissues. The red solid line represents the linear regression fit, and the shaded red area indicates the 95% confidence interval. n represents number of biologically independent tumor samples from distinct patients.

Statistical analysis: The correlation coefficient (R) and P value were calculated using Spearman's rank correlation analysis.

Figure S10

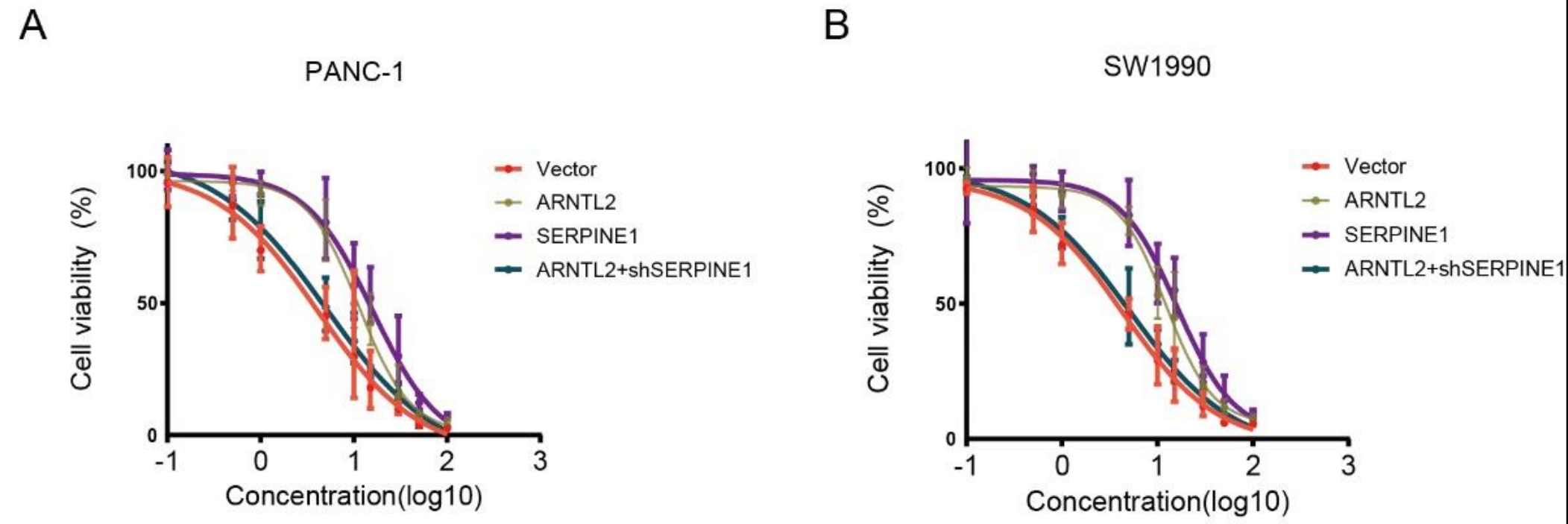

	Vector	ARNTL2	SERPINE1	ARNTL2+shSERPINE1
IC50 (nM)	4.13	11.34	15.95	5.07

	Vector	ARNTL2	SERPINE1	ARNTL2+shSERPINE1
IC50 (nM)	4.30	12.25	16.05	5.08

Figure S10. Dose-response analysis of the selective KRAS G12D inhibitor zoldonrasib (RMC-9805) in PANC-1 and SW1990 cells with modulated ARNTL2 and SERPINE1 expression.

PANC-1 (A) and SW1990 (B) cells with the indicated genetic modifications were treated with increasing concentrations (0.1 to 100 nmol/L) of zoldonrasib (RMC-9805), and cell viability was assessed using the CCK-8 assay. Data are presented as mean ± SD from three biologically independent experiments with three technical replicates per concentration. Dose-response curves were generated using nonlinear regression analysis, and IC50 values were calculated and summarized in the tables below each panel.

Supplemental Table

Table S1. Primer sequences used for qRT-PCR and ChIP-qPCR analyses.

Species	Gene	Forward Primer (5′→3′)	Reverse Primer (5′→3′)
Human	ARNTL2	GTGGGAAGTAATTATAGACC	CTGACTTAGAAACGAAGAG
Human	SERPINE1	ATCAATGACTGGGTGAAGAC	TTGAAGTAGAGGGCATTCAC
Human	CD44	ATCTTGGCATCCCTCTTGGC	AGCTCCATTGCCACTGTTGA
Human	SOX2	TACAGCATGTCCTACTCGCAG	GAGGAAGAGGTAACCACAGGG
Human	NANOG	TTTGTGGGCCTGAAGAAAACT	AGGGCTGTCCTGAATAAGCAG
Human	GAPDH	TCCATGACAACTTTGGTATC	CAGGGATGATGTTCTGGA
Human	SERPINE1 promoter (ChIP-qPCR)	GGAGTCAGCCGTGTATCATC	GCCACGTGATTGTCTAGGTT

Table S2. shRNA sequences used in this study.

Target	Sequence (5′→3′)
shARNTL2-1	GCGCGTAAACTGGACAAACTT
shARNTL2-2	GCCTCCAAATATTGTTGGAAT
shARNTL2-3	TTGACTGGACAAAGCTTATTT
shSERPINE1-1	TCTCTGCCCTCACCAACATTC
shSERPINE1-2	AGACCAACAAGTTCAACTATA

Table S3. Sequences of SERPINE1 promoter constructs used for luciferase reporter assays.

**Construct**	**Position**	**Length (bp)**
WT	Full promoter	2012
Promoter-1	Region 1	512

Promoter-2	Region 2	512
Promoter-3	Region 3	512
Promoter-4	Region 4	512
