# Supplementary material for: ARNTL2 promotes metastatic phenotypes through SERPINE1 transcription in KRAS G12D pancreatic cancer
Source: iScience. 2026 Sep 8;29(9):117479. doi: 10.1016/j.isci.2026.117479 (PMC13573702; doi:10.1016/j.isci.2026.117479)

Original images of western-blotting experiments

- Original, uncropped western-blotting images from two independent repeats for each condition are provided.
- The images used in the published figures are indicated by red boxes.

- Molecular weight standards used in this study.

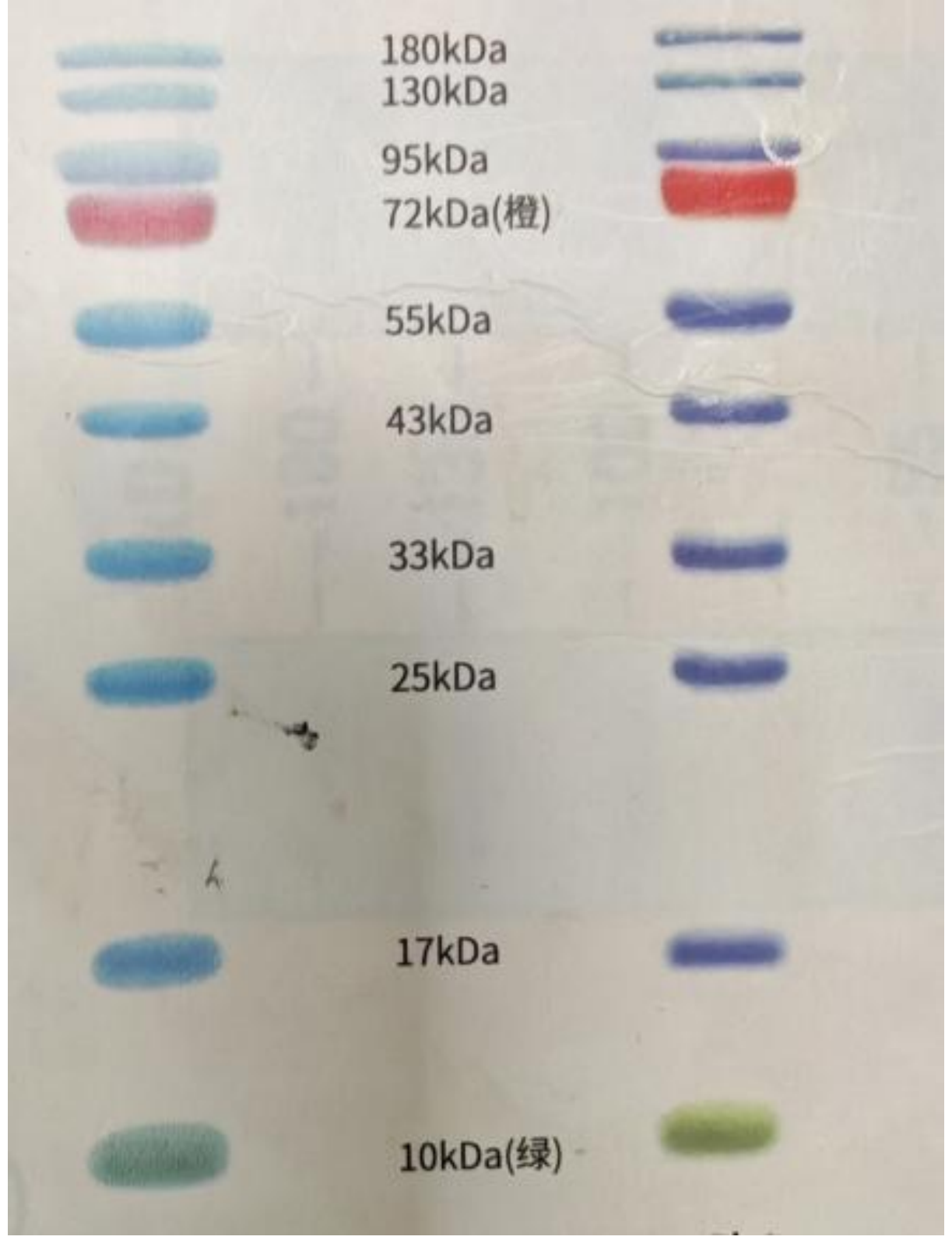

Figure 4A: ARNTL2 (three repeats)

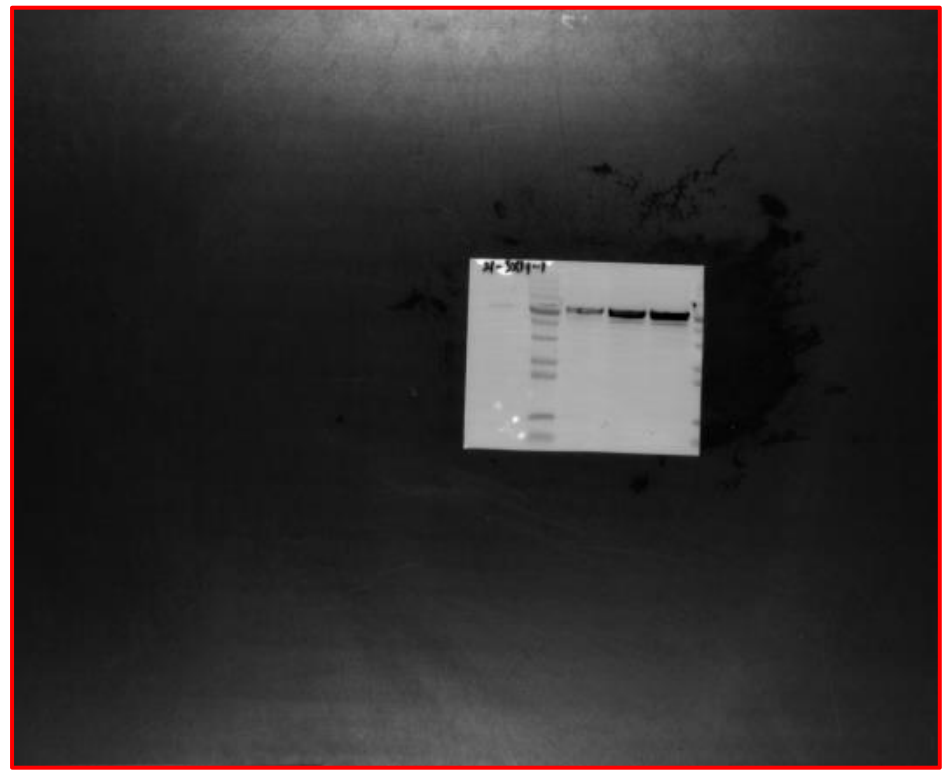
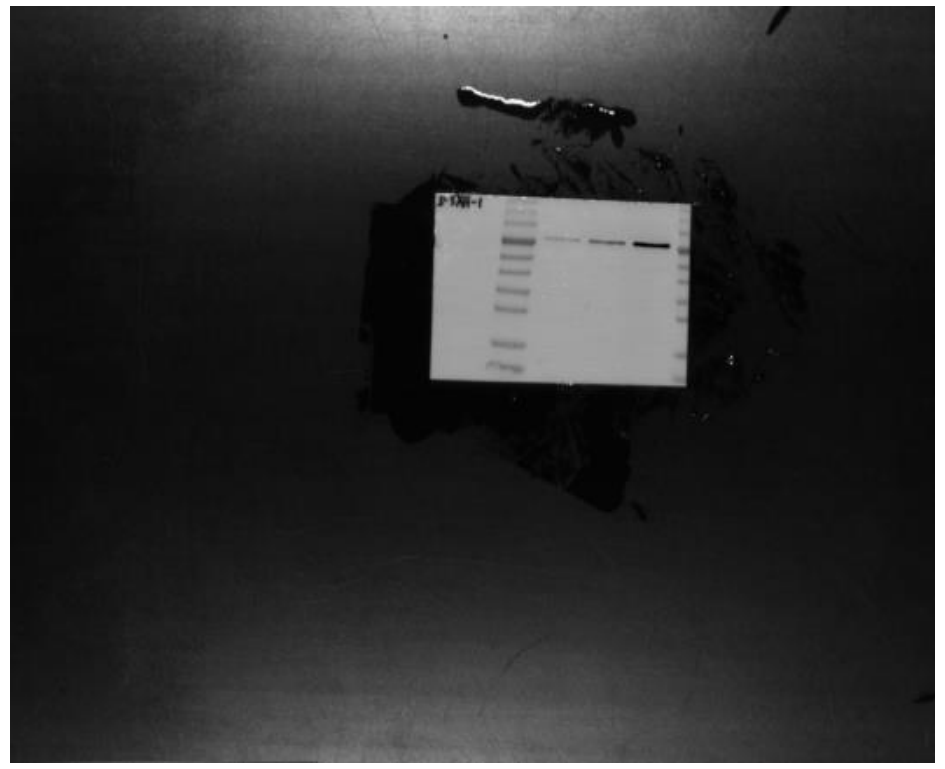

Figure 4C PANC-1: ARNTL2 (three repeats)

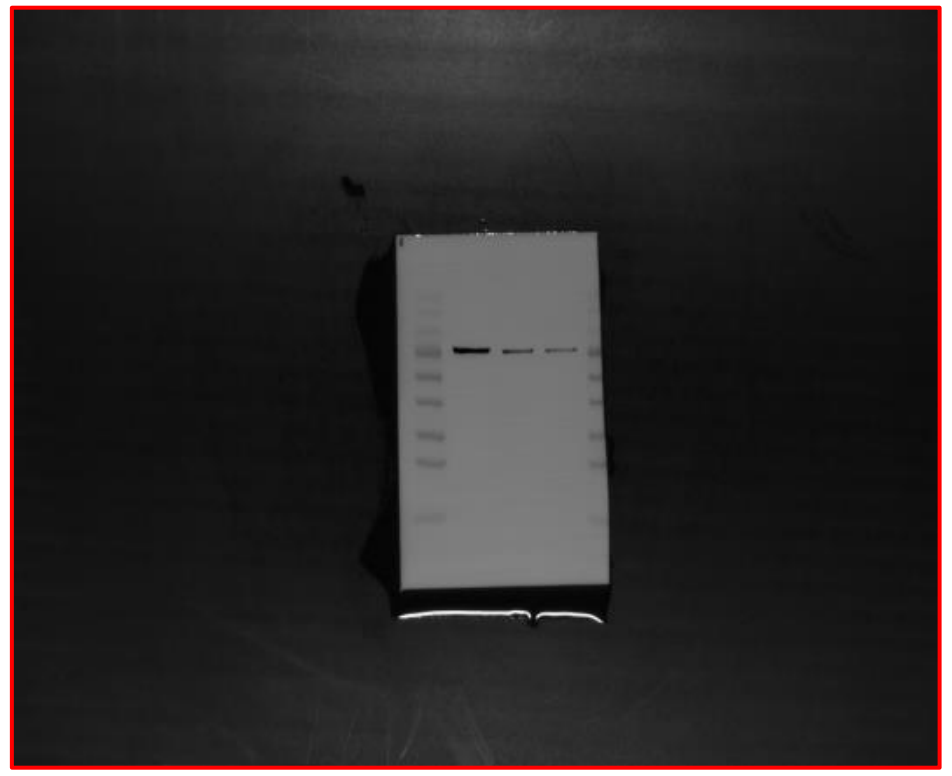
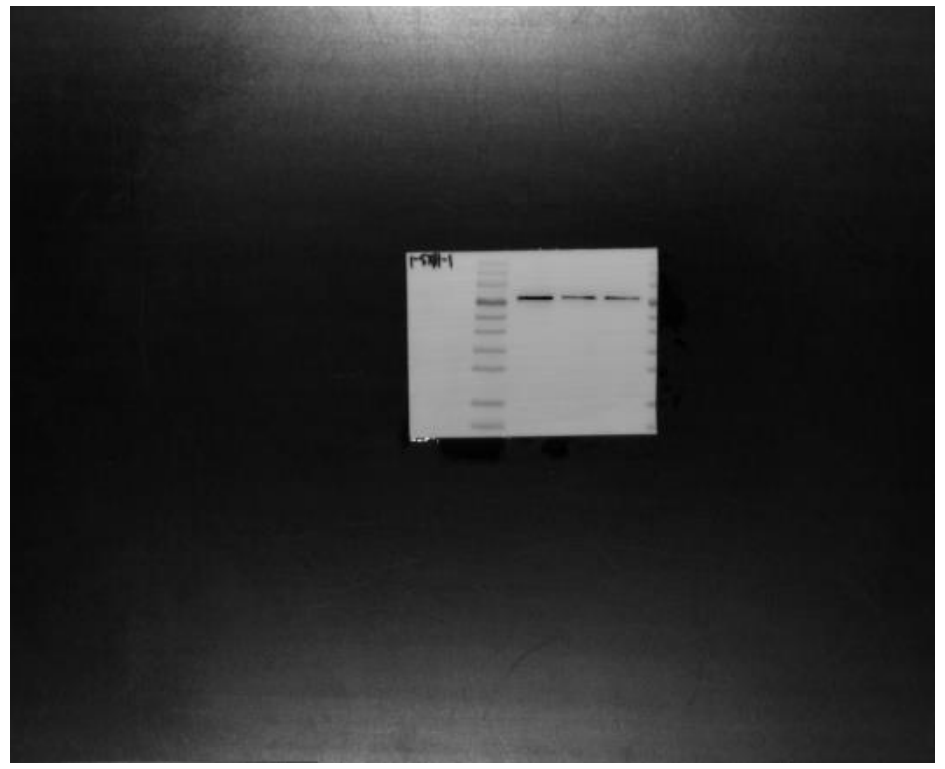

Figure 4A: SERPINE1 (three repeats)

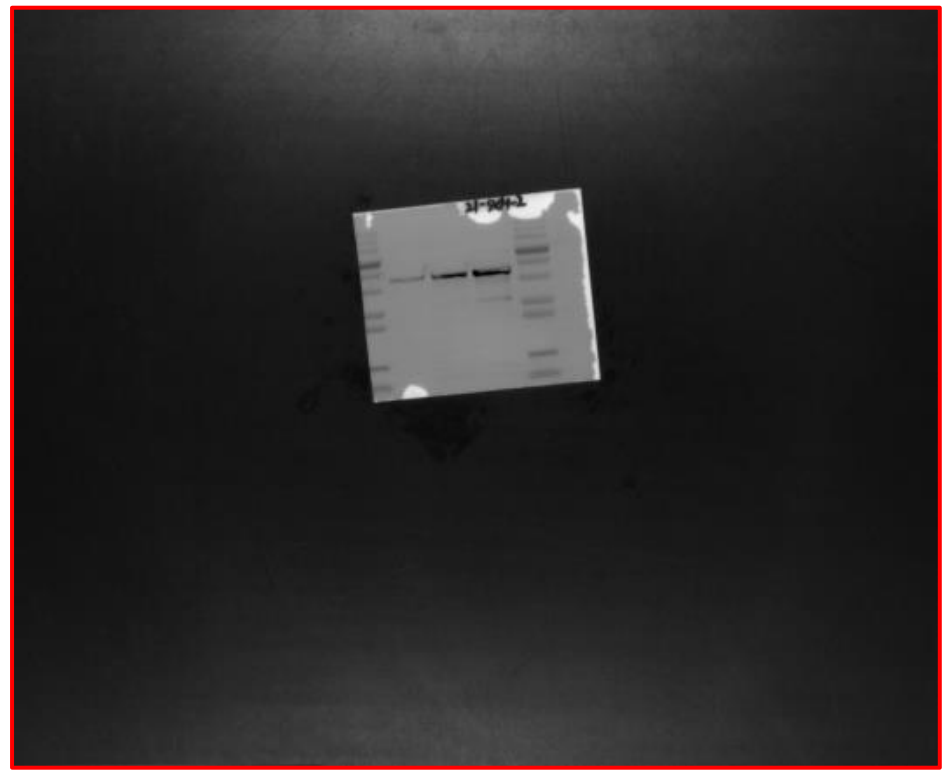
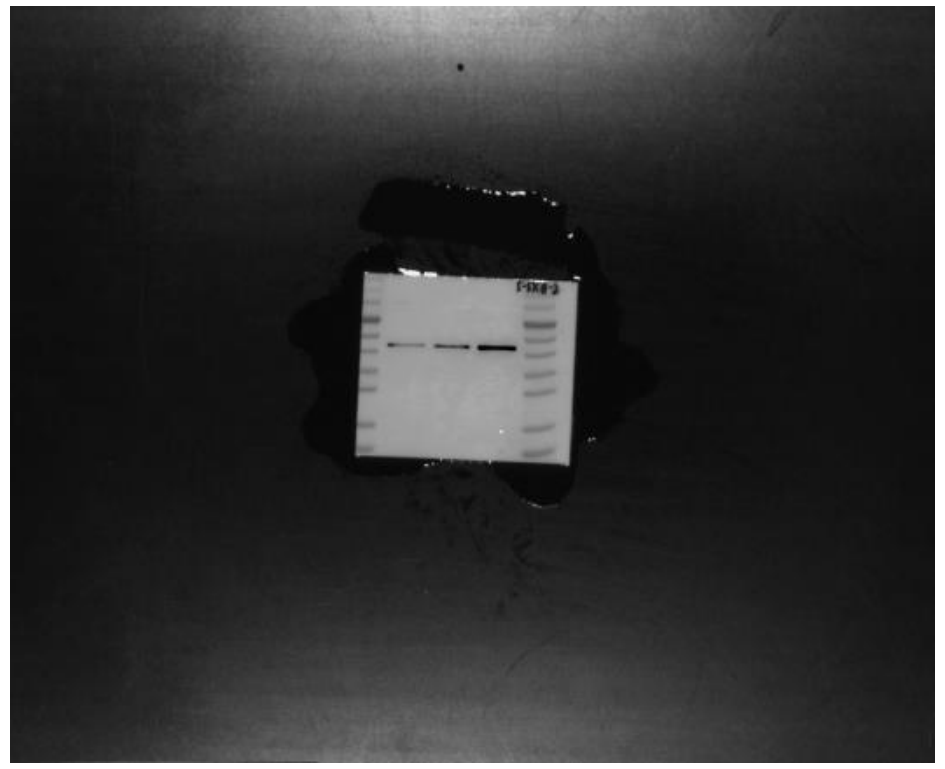

Figure 4C PANC-1: SERPINE1 (three repeats)

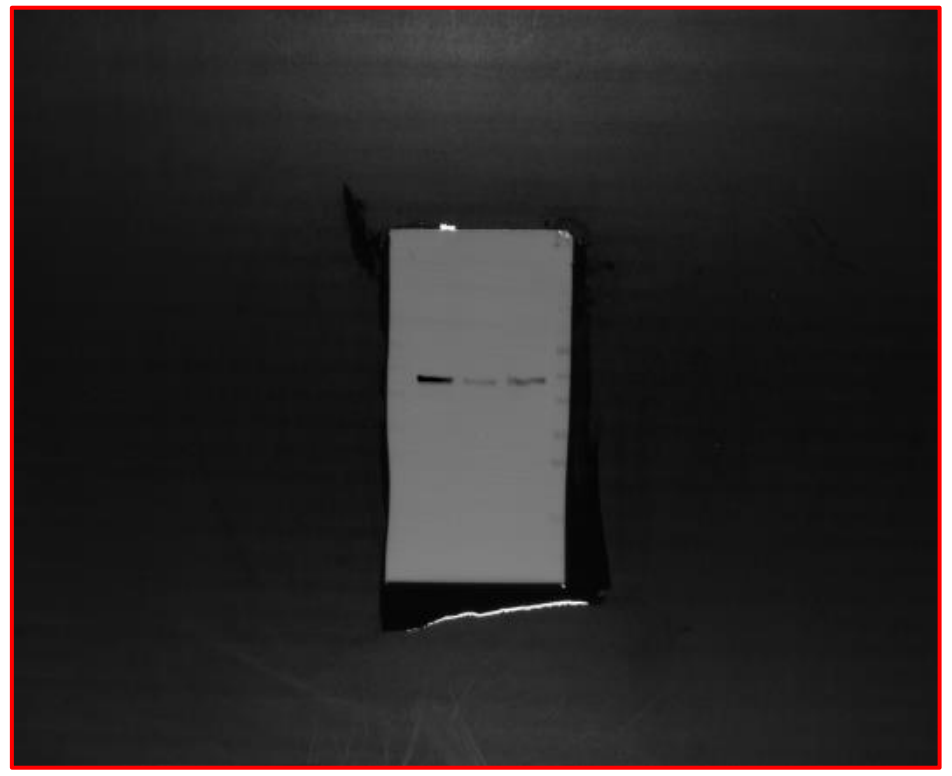
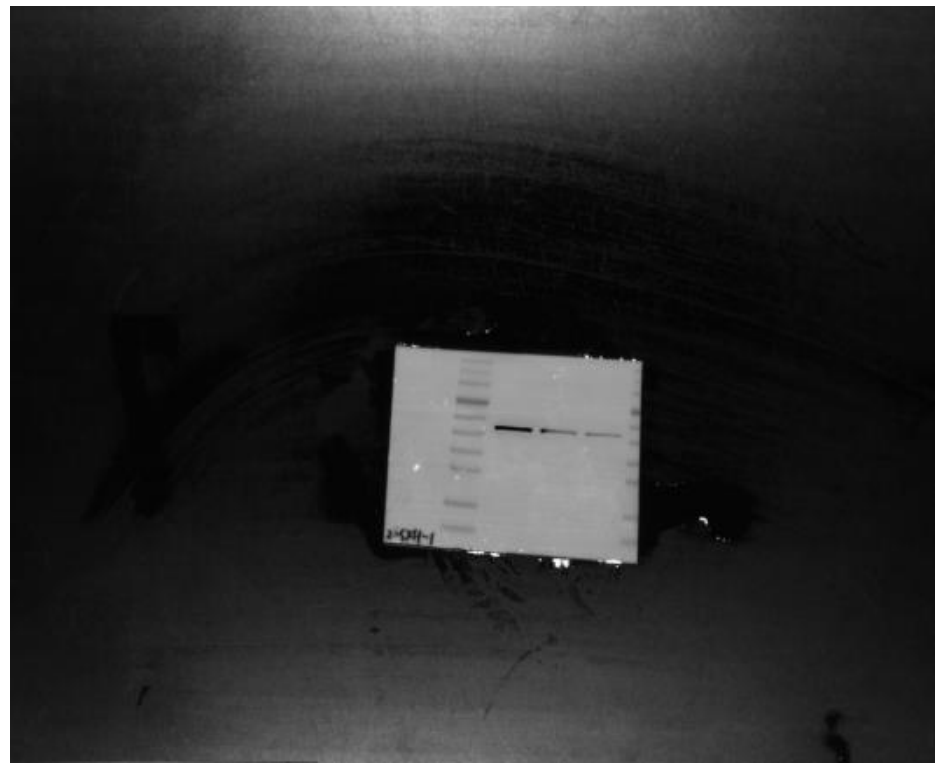

Figure 4A: GAPDH (three repeats)

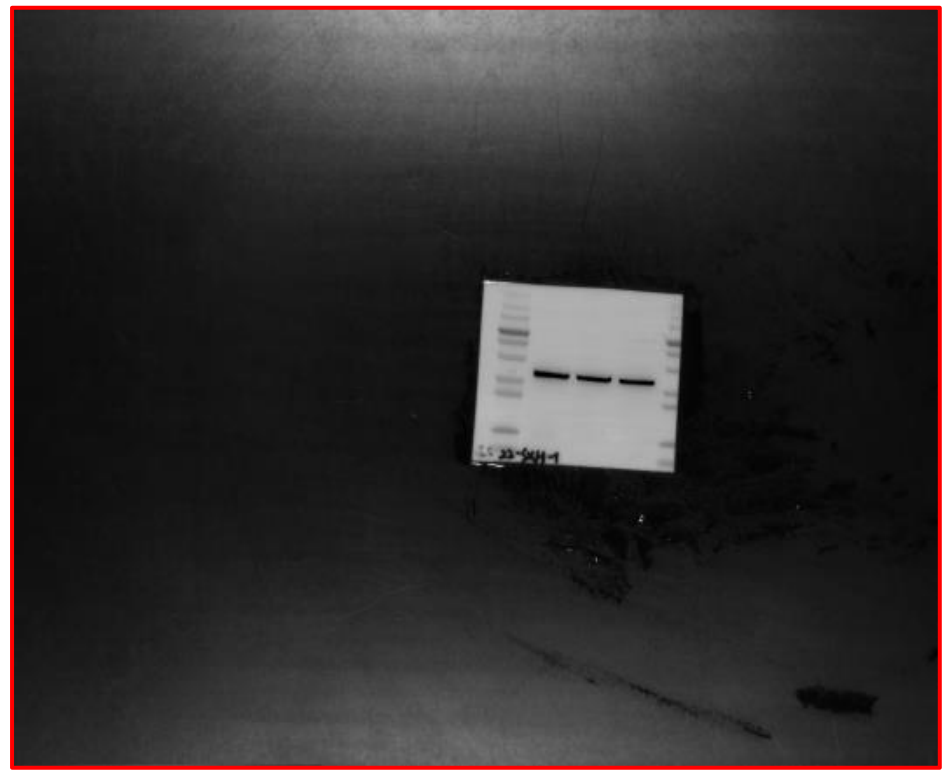
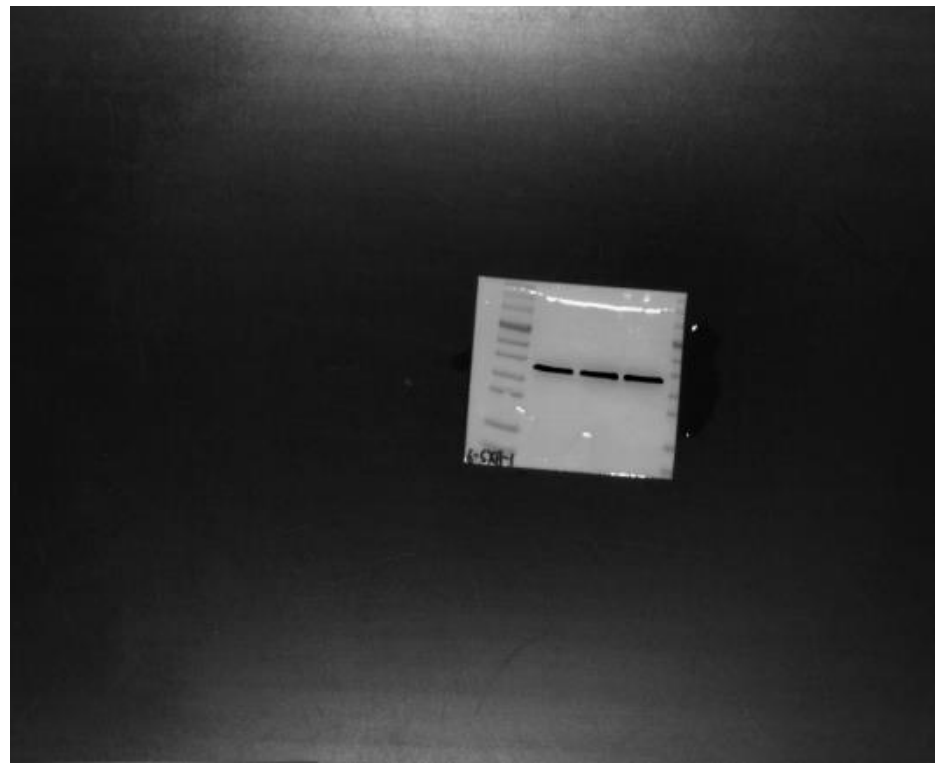

Figure 4C PANC-1: GAPDH (three repeats)

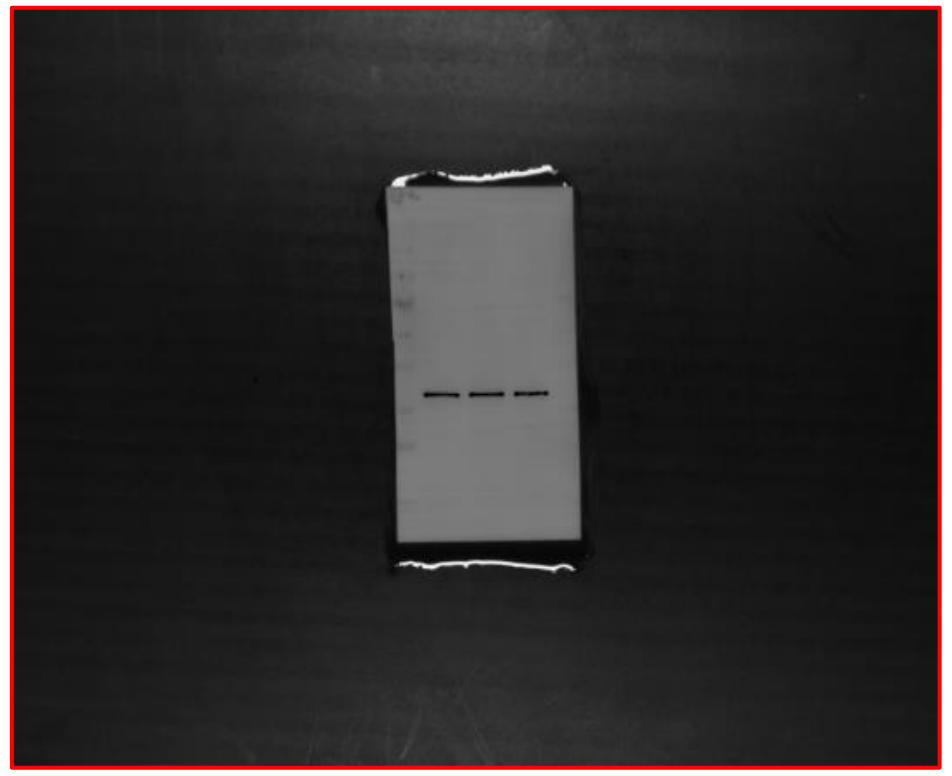
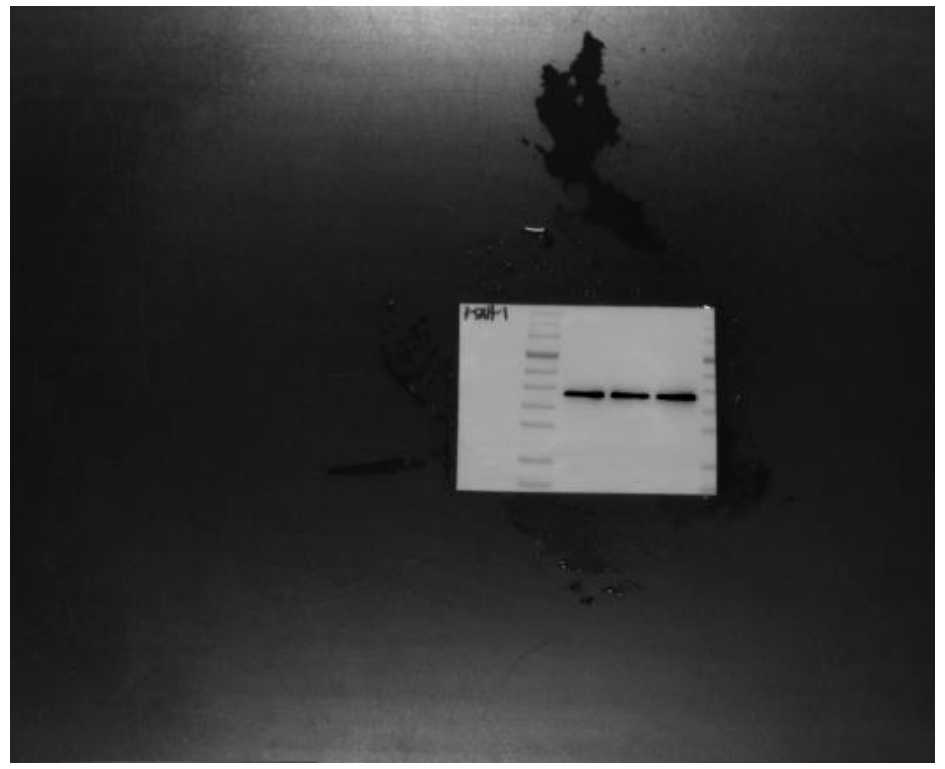

Figure 4C SW1990: ARNTL2 (three repeats)

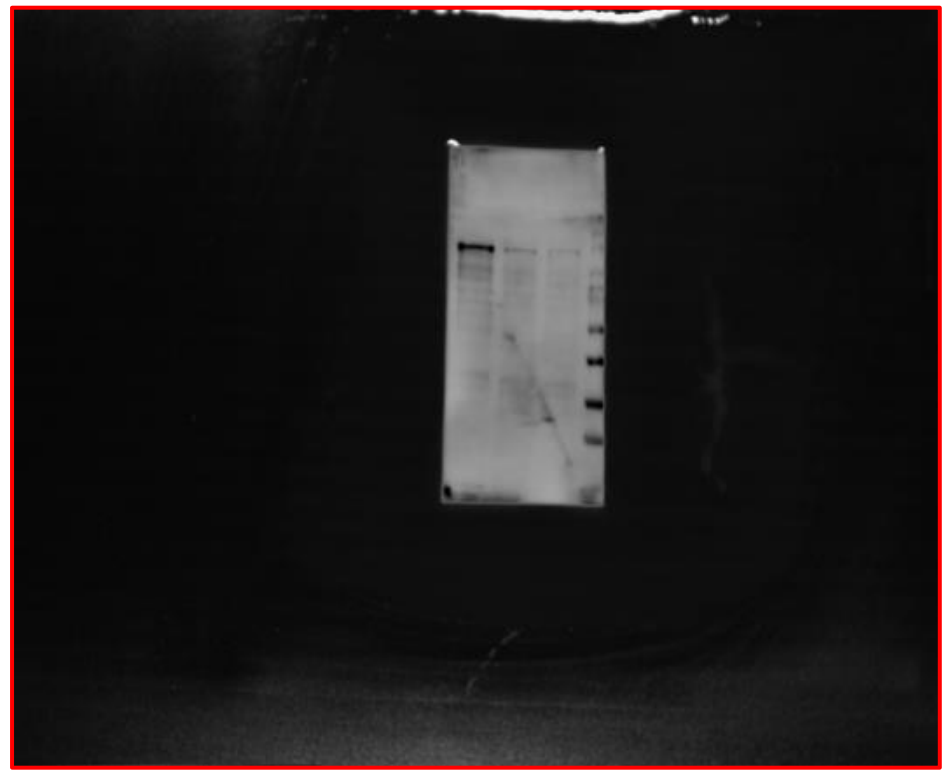

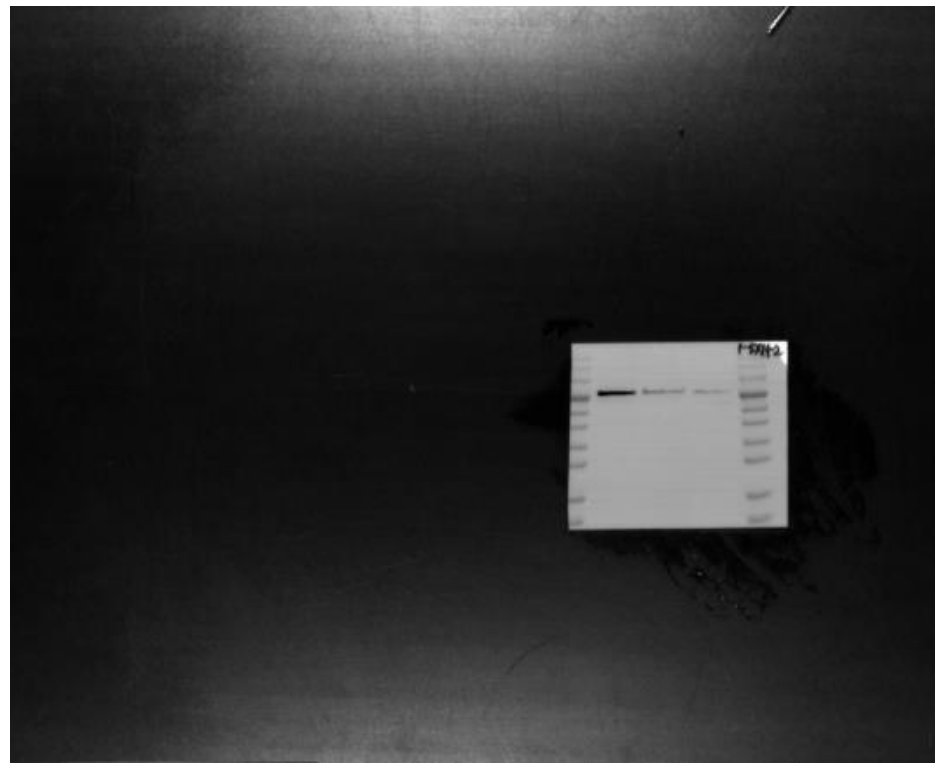

Figure 4C SW1990 : SERPINE1 (three repeats)

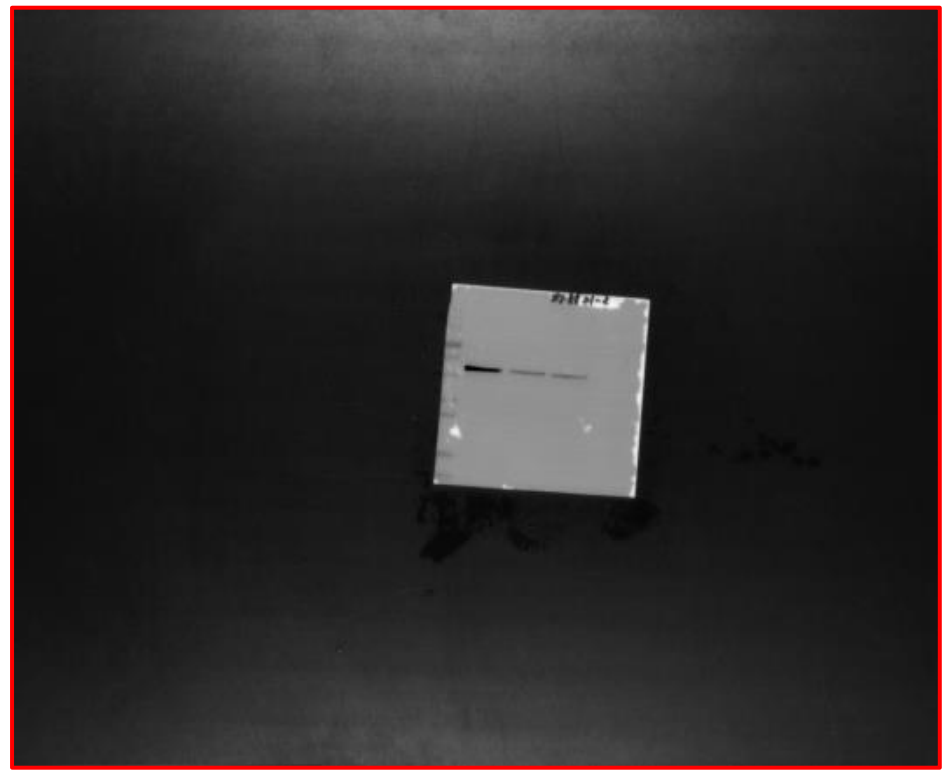

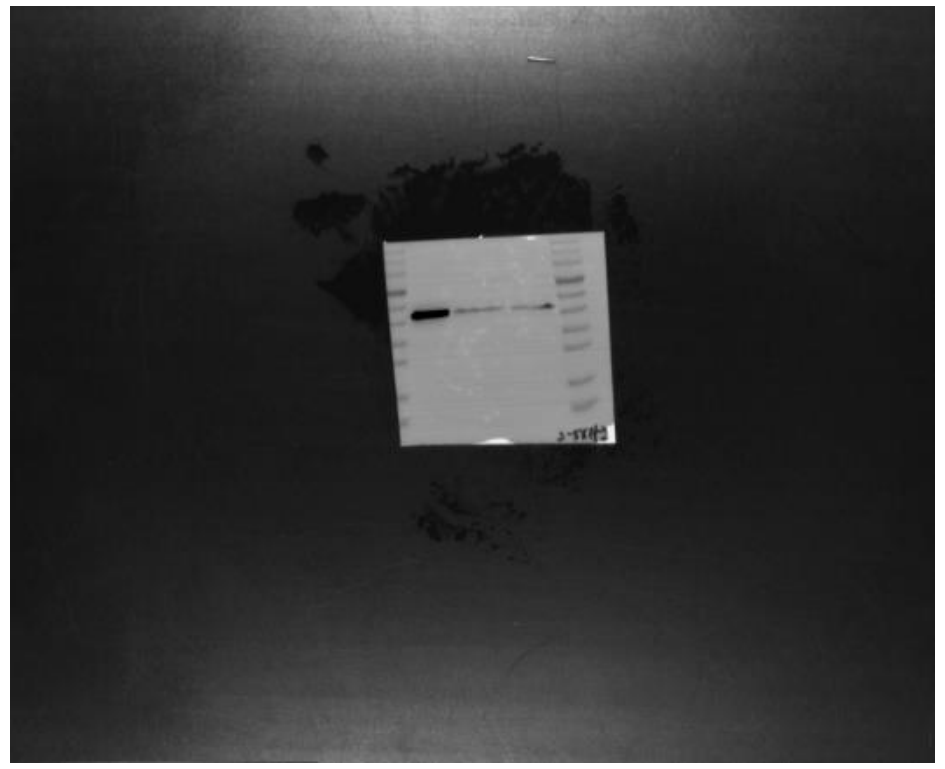

Figure 4C SW1990 : GAPDH (three repeats)

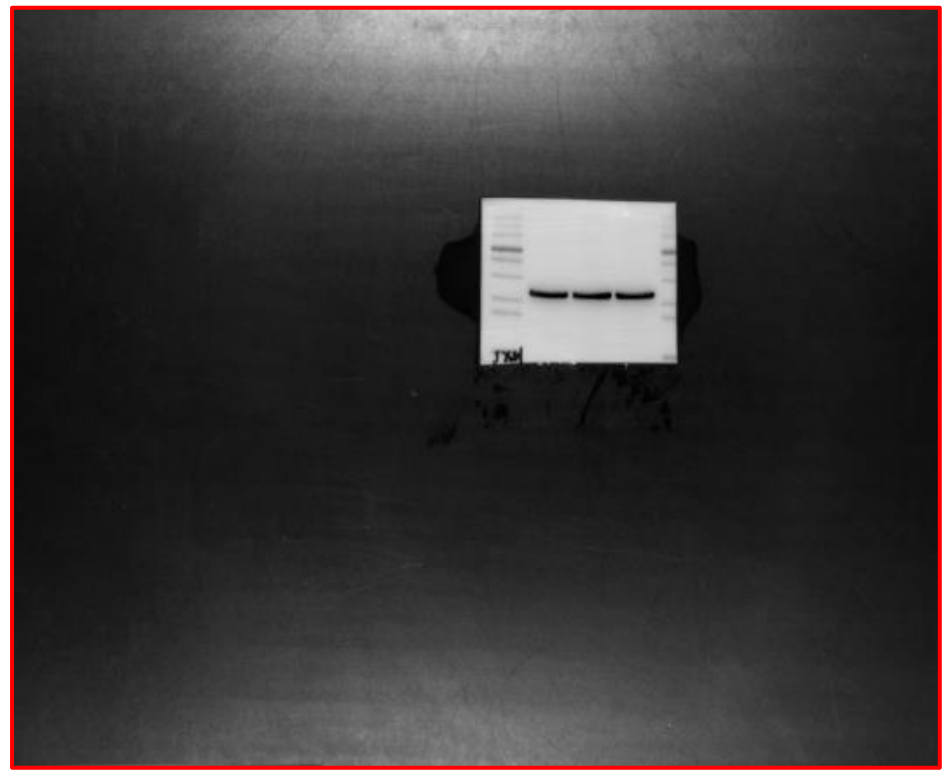

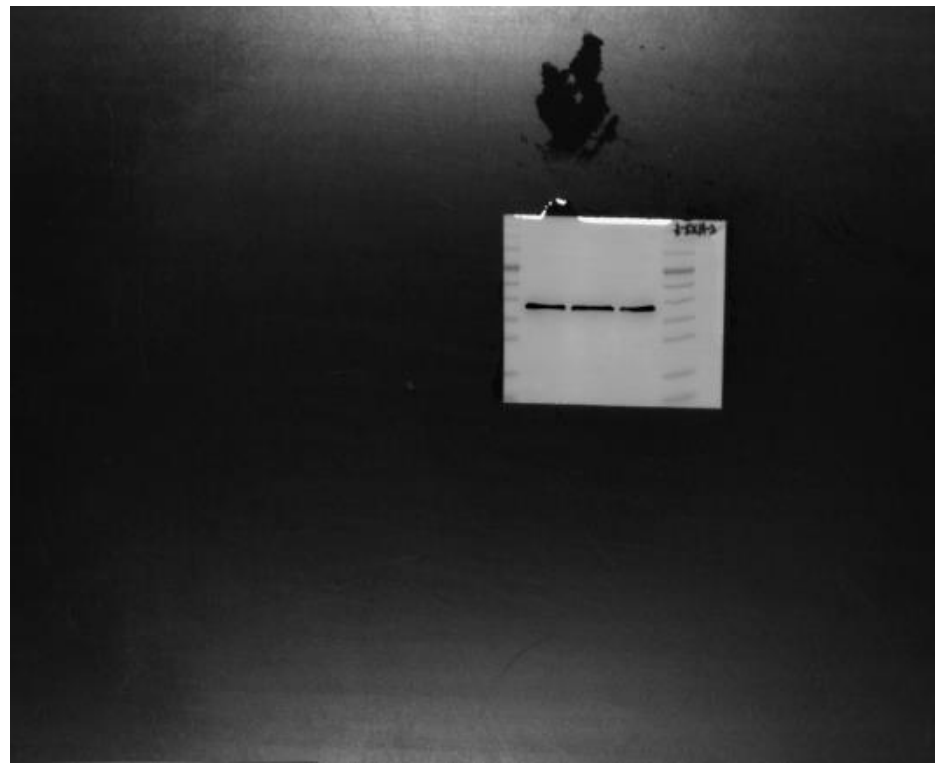

Figure 4C BxPC-3: ARNTL2 (three repeats)

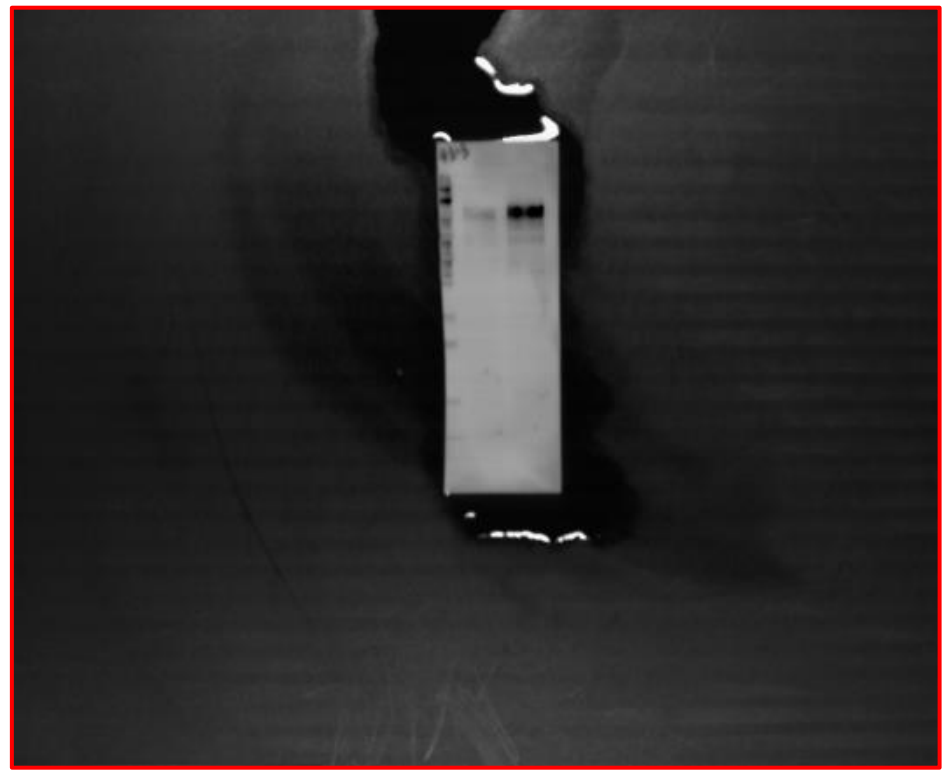

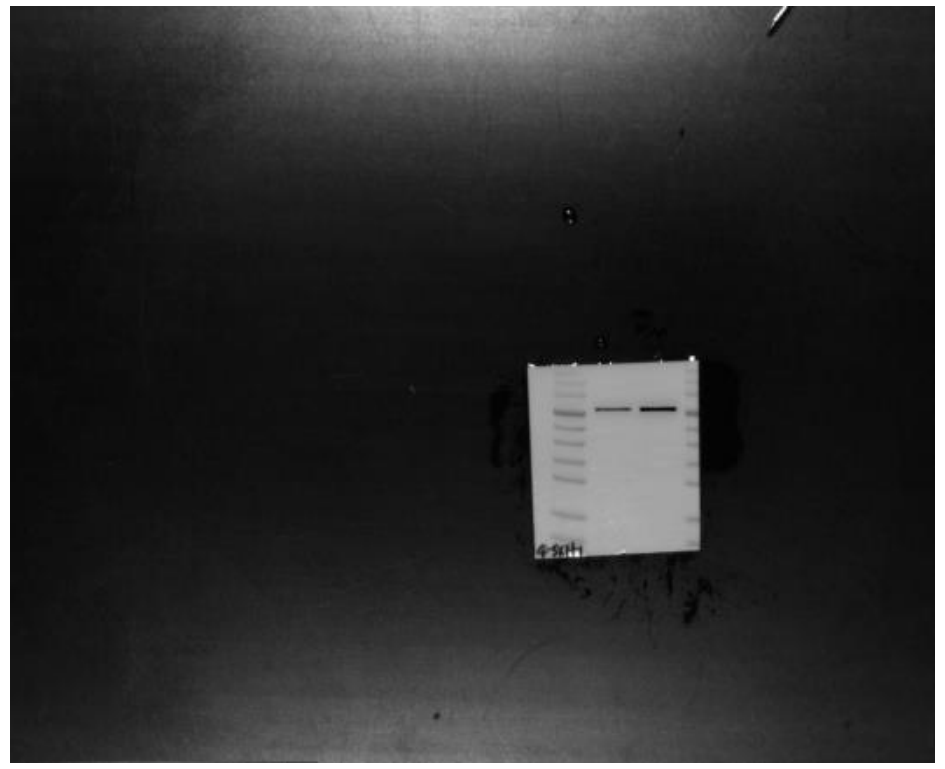

Figure 4C BxPC-3: SERPINE1 (three repeats)

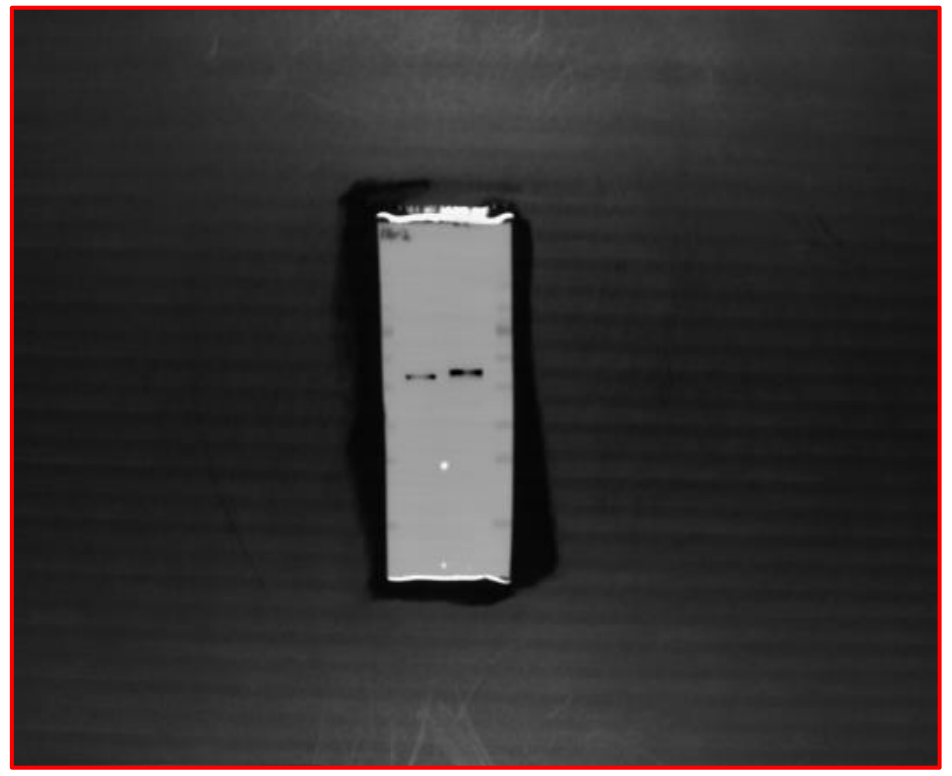

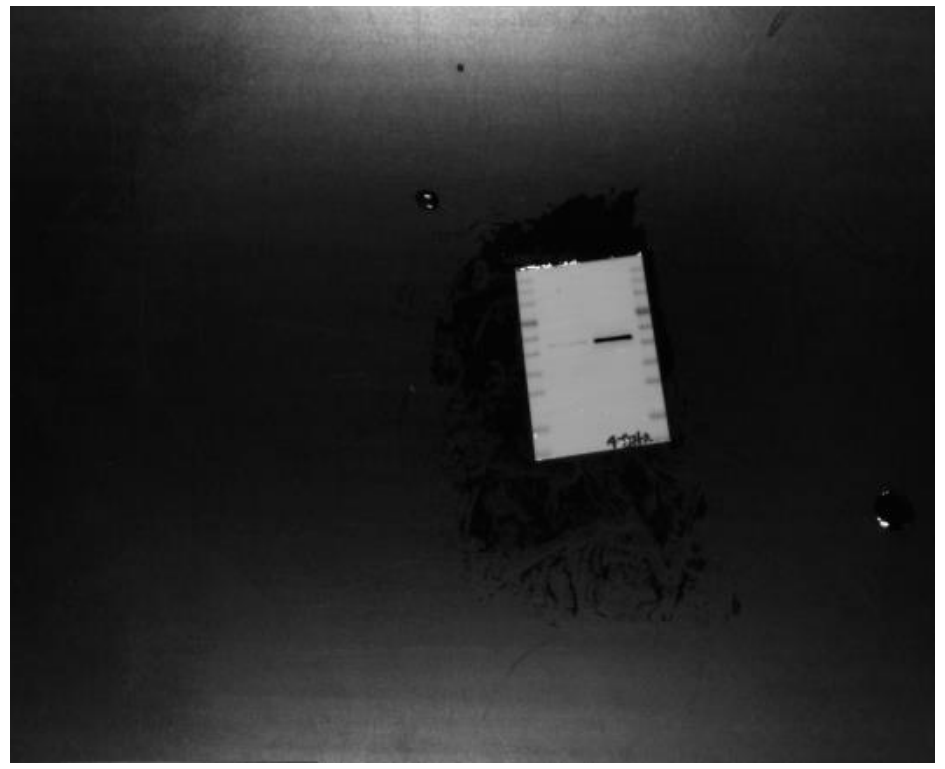

Figure 4C BxPC-3: GAPDH (three repeats)

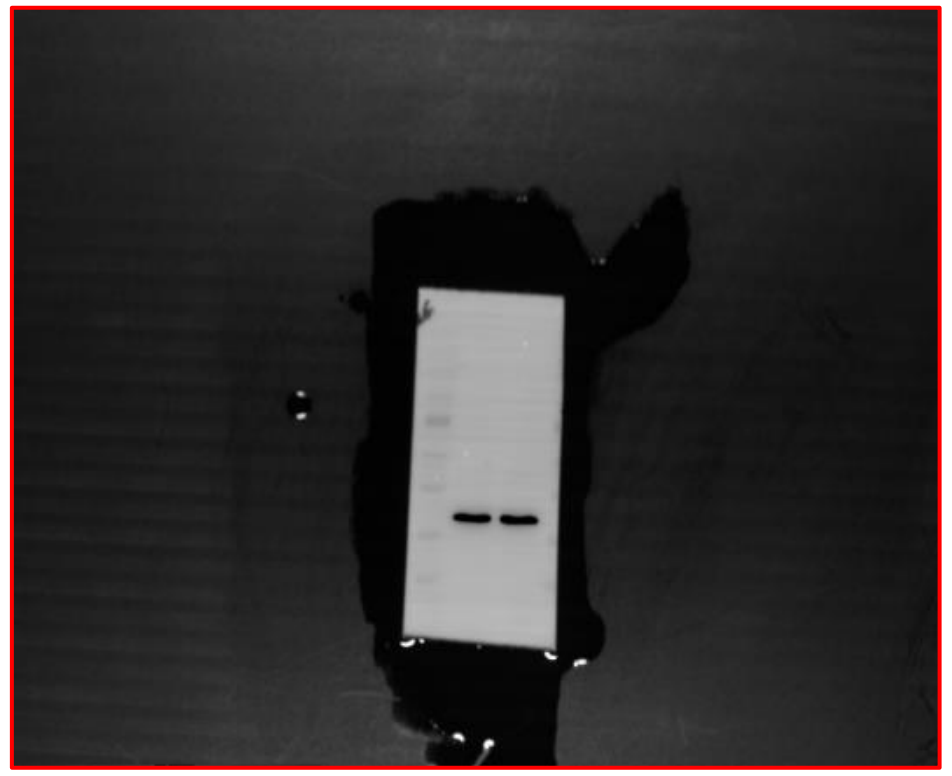

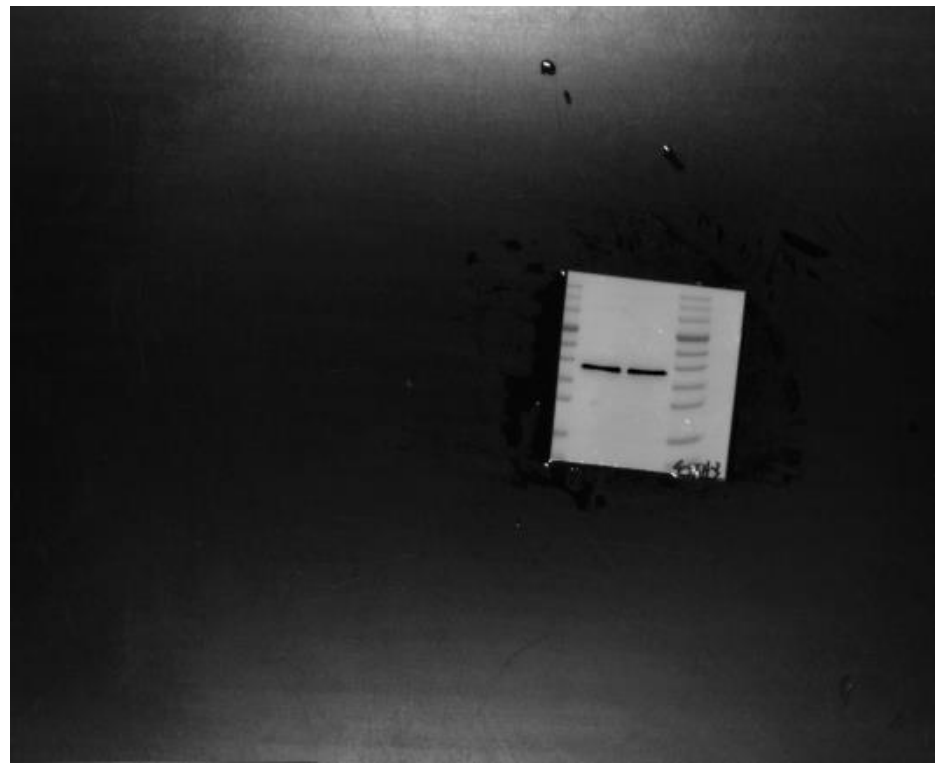

Figure 6D: E-cadherin (three repeats)

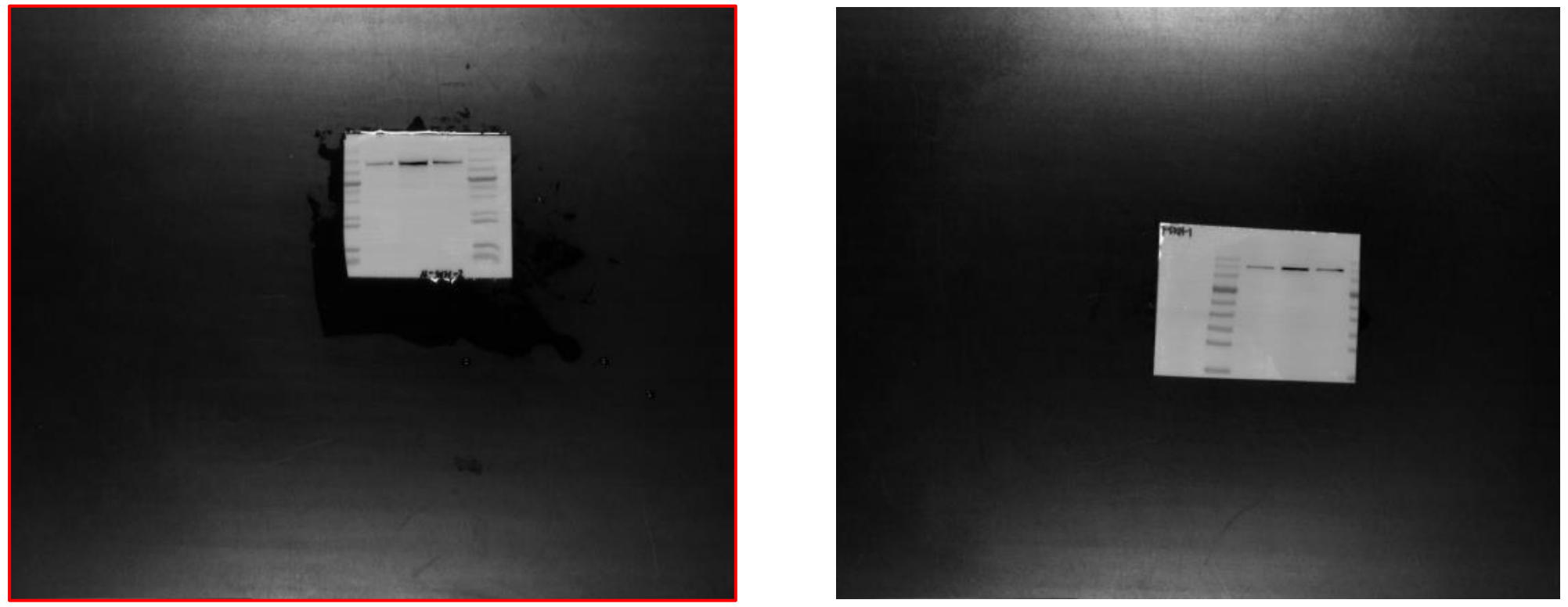

Figure 6D: Vimentin (three repeats)

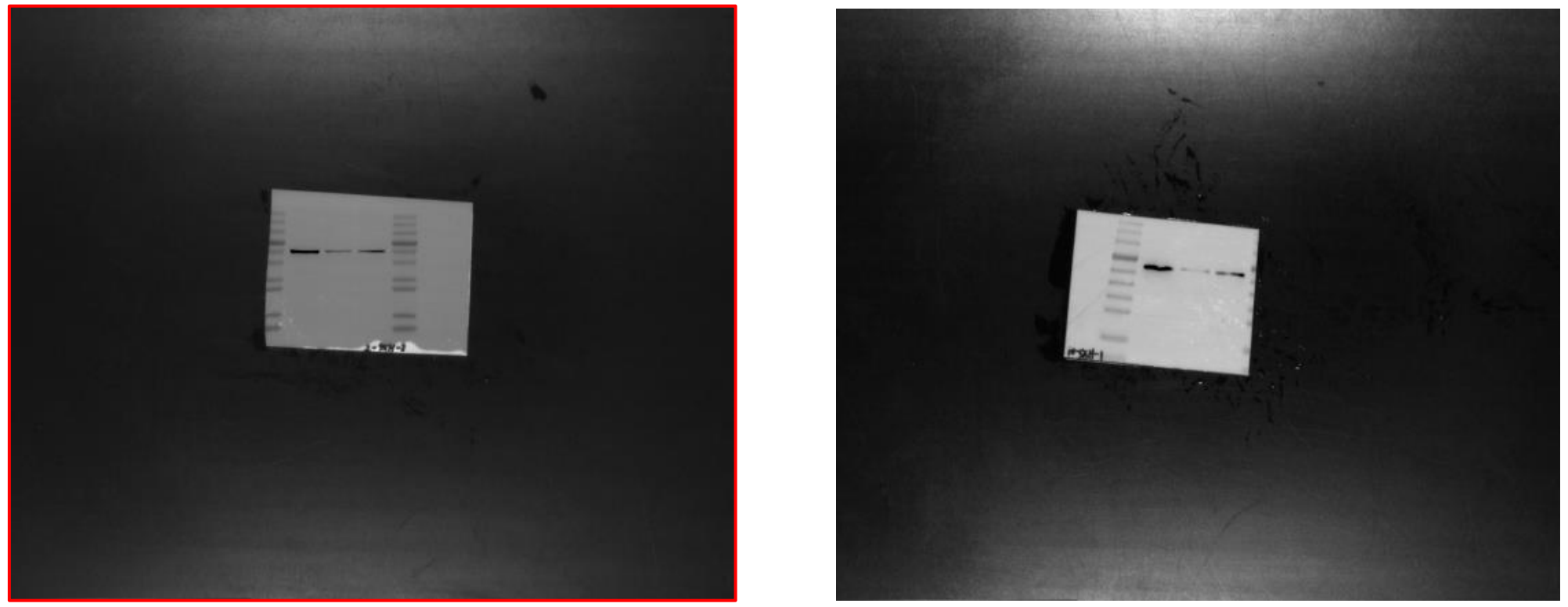

Figure 6D: N-cadherin (three repeats)

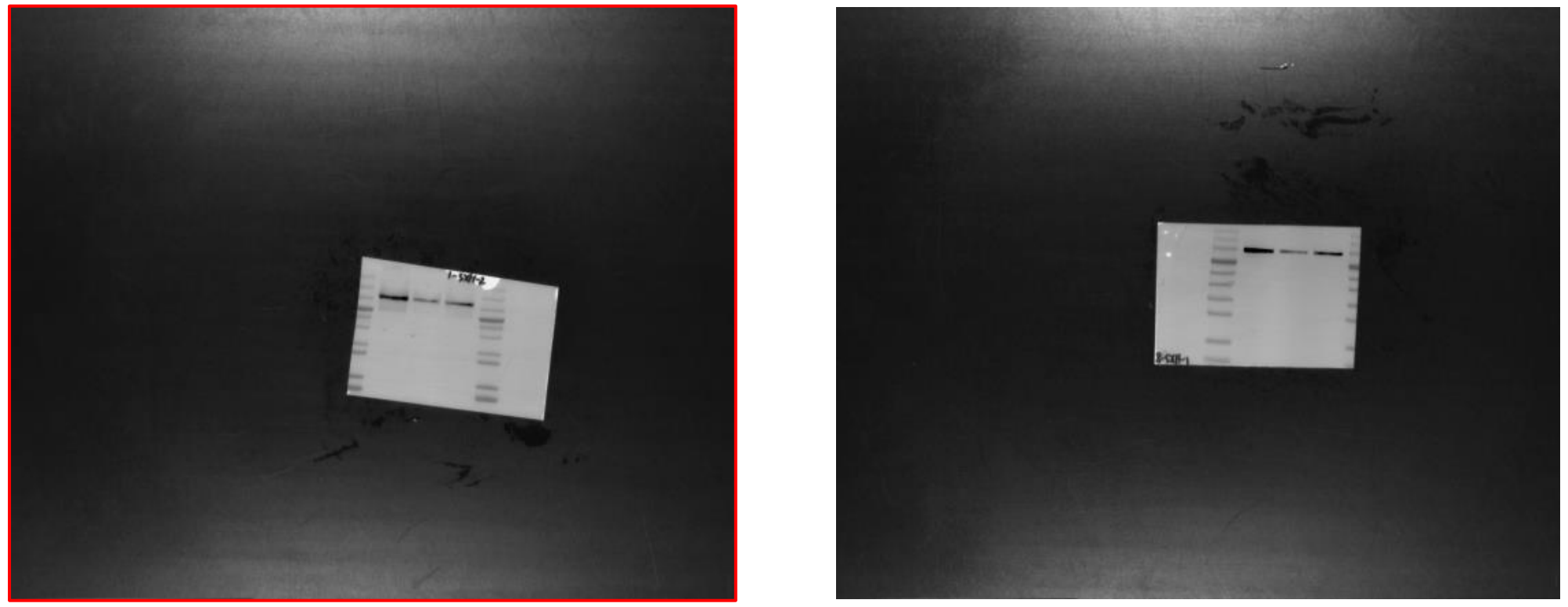

Figure 6D: SOX2 (three repeats)

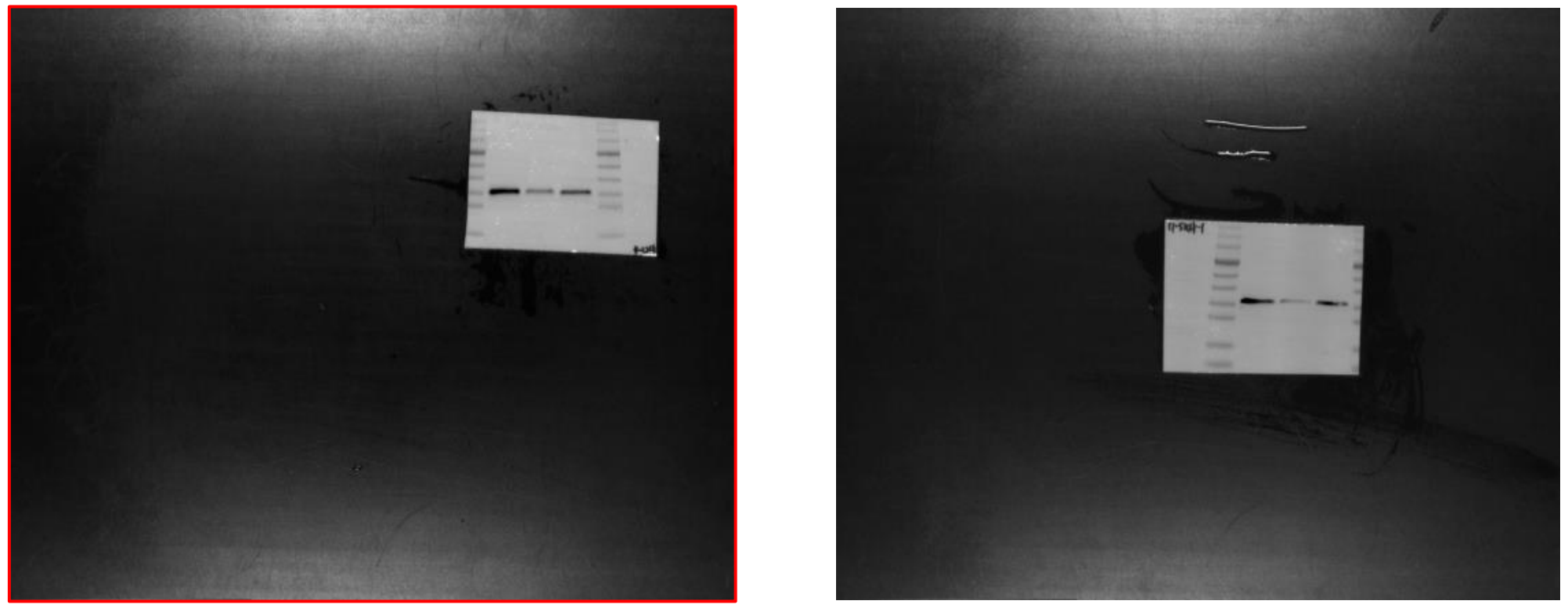

Figure 6D: CD44 (three repeats)

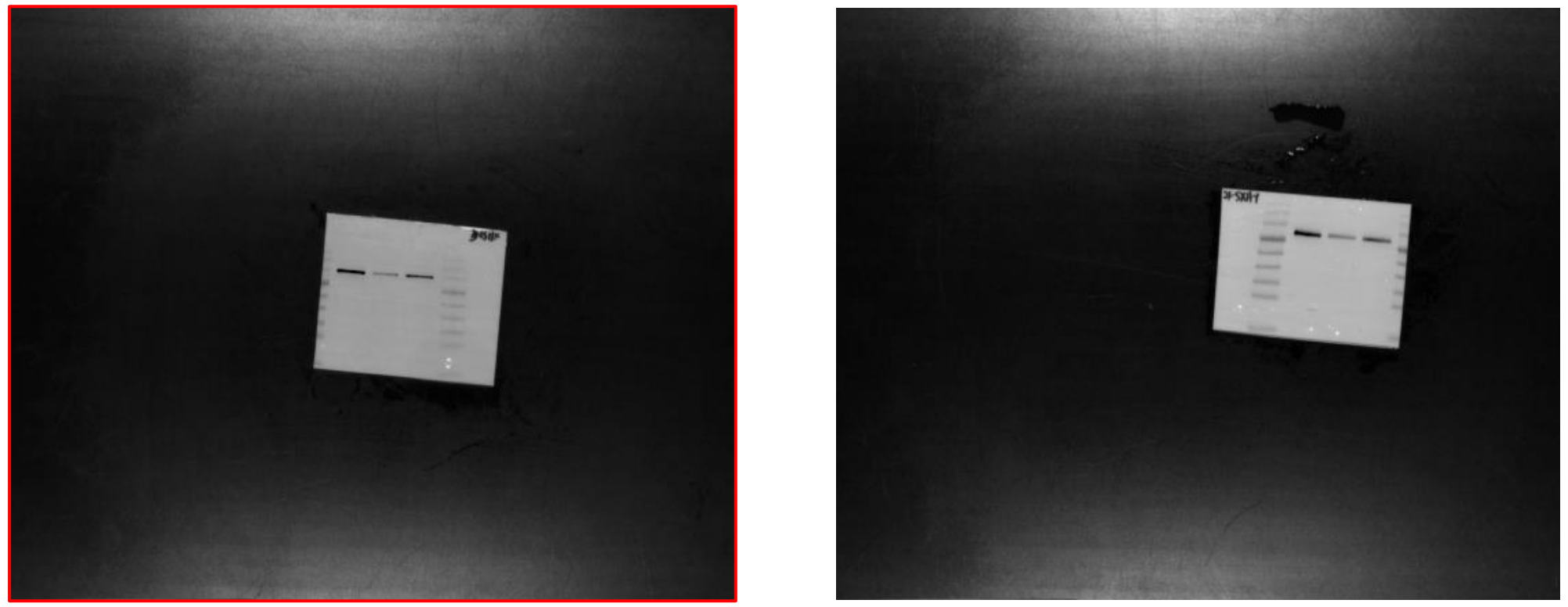

Figure 6D: NANOG (three repeats)

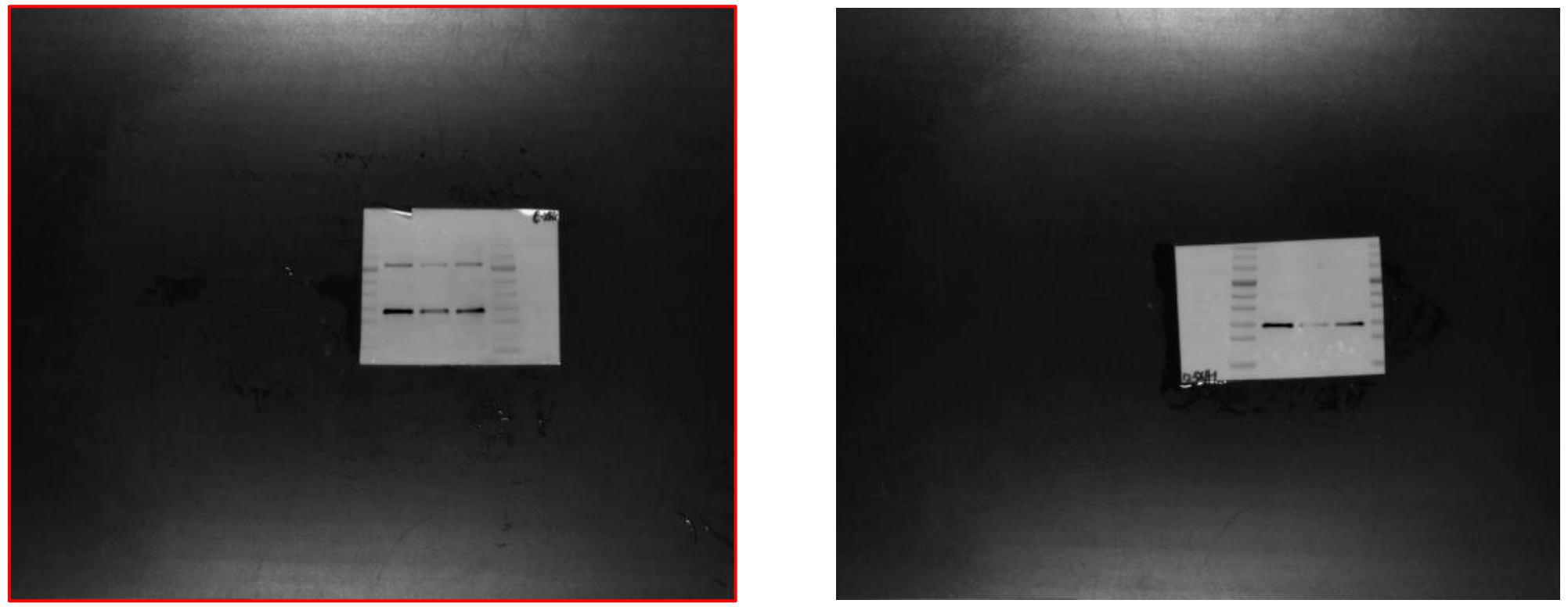

Figure 6D: SNAI1 (three repeats)

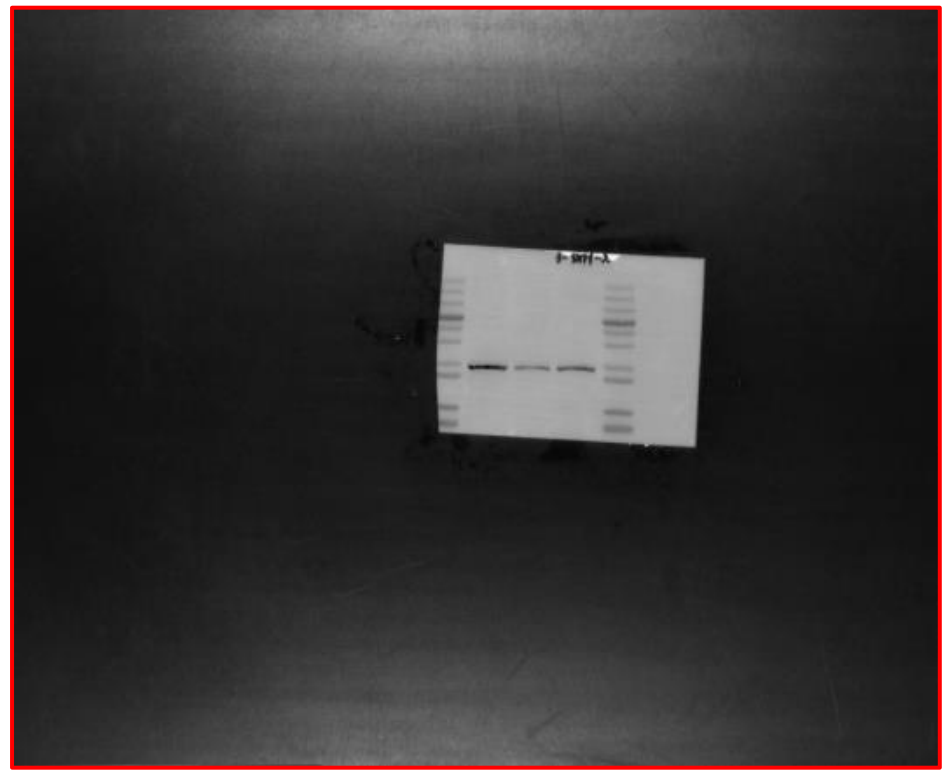

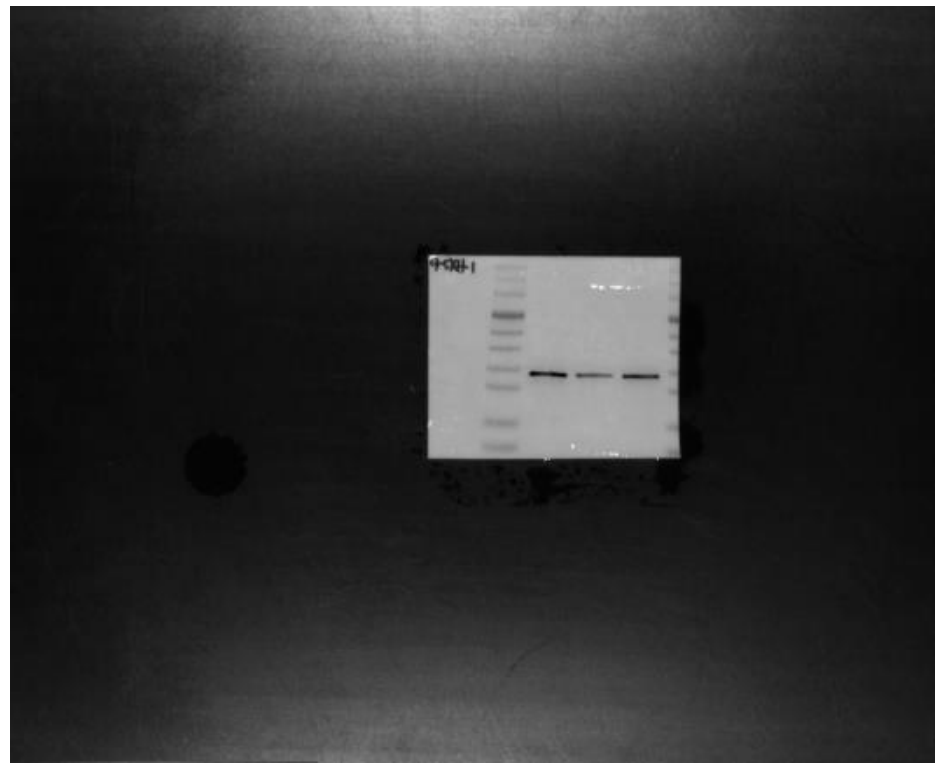

Figure 6D: GAPDH (three repeats)

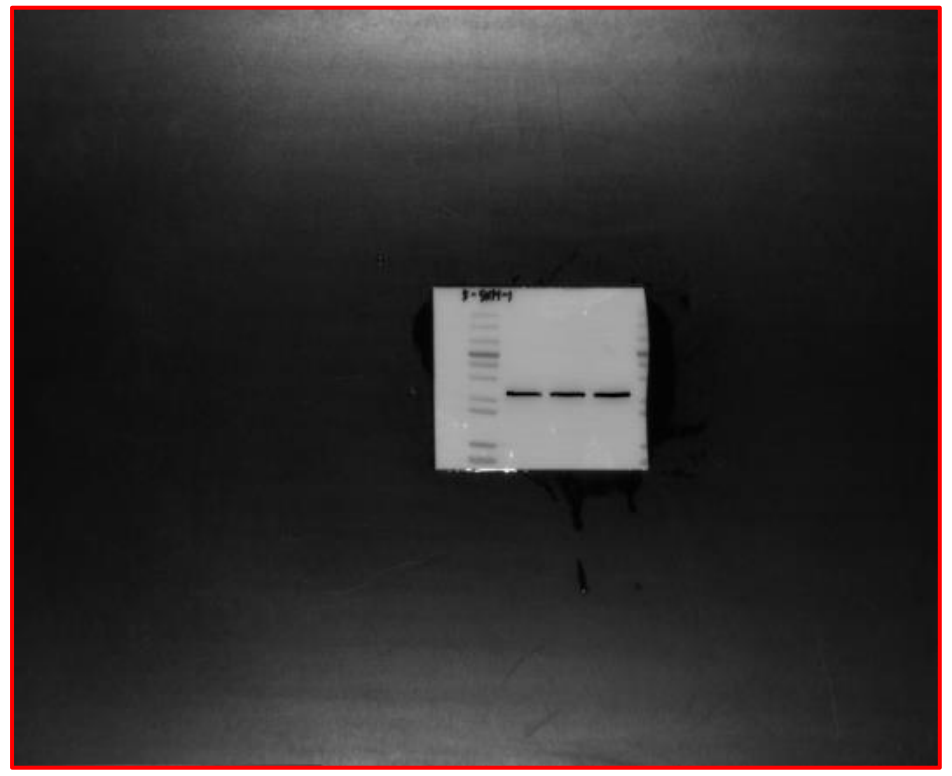

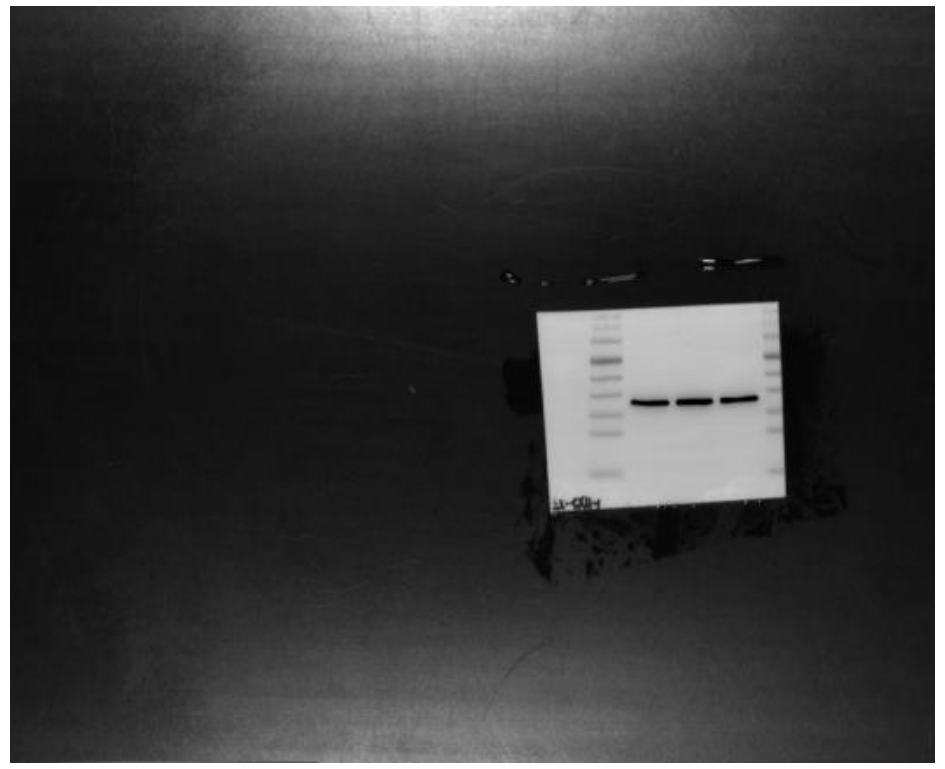

Figure 6E: E-cadherin (three repeats)

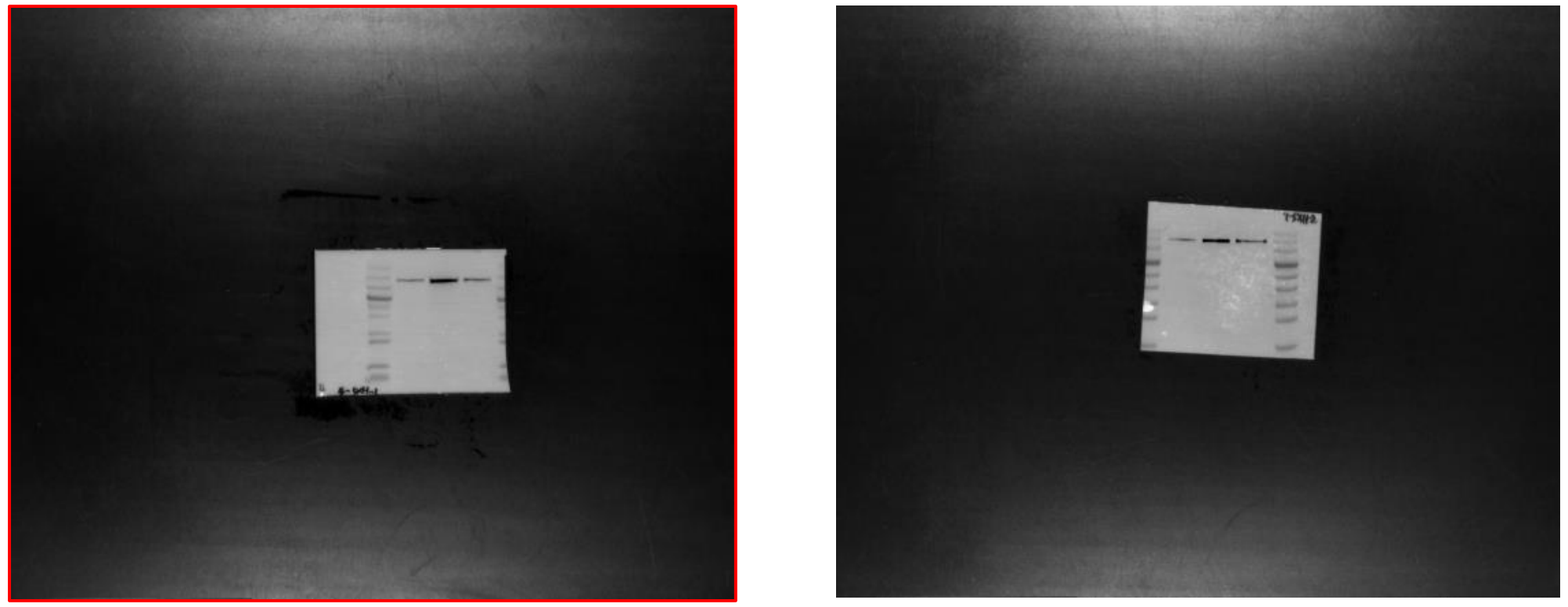

Figure 6E: N-cadherin (three repeats)

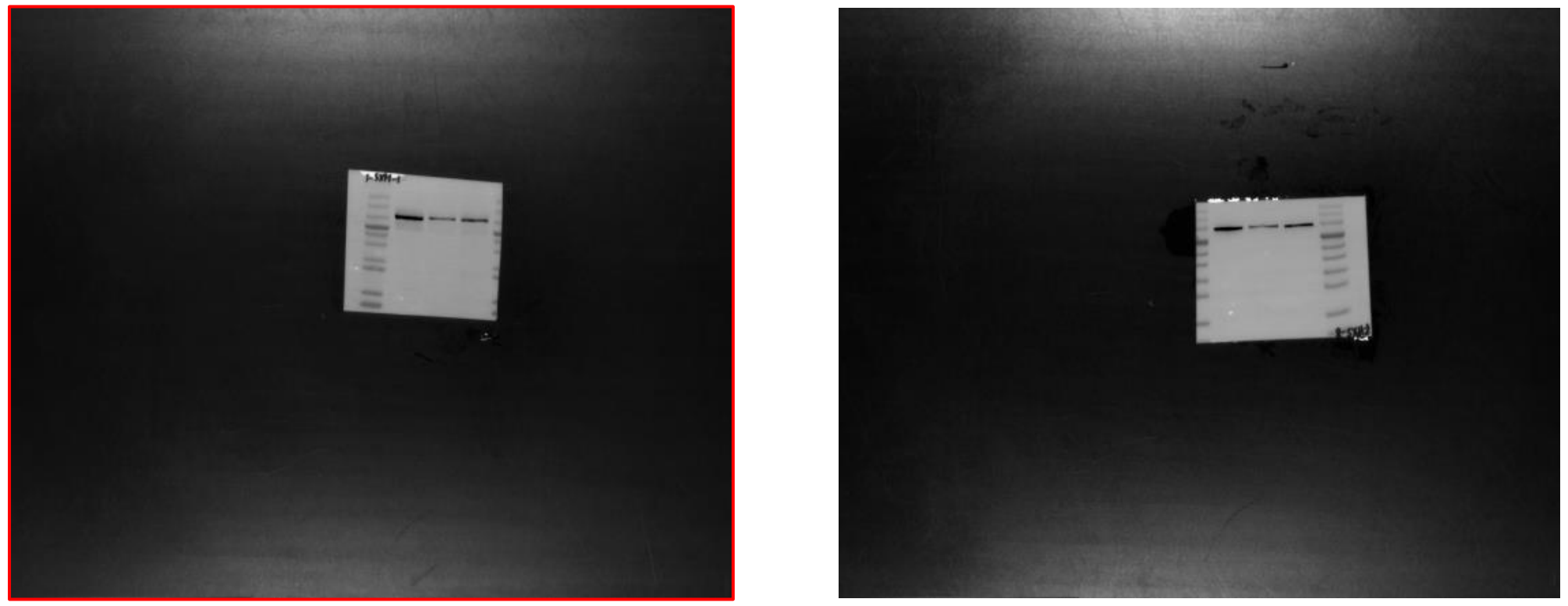

Figure 6E: CD44 (three repeats)

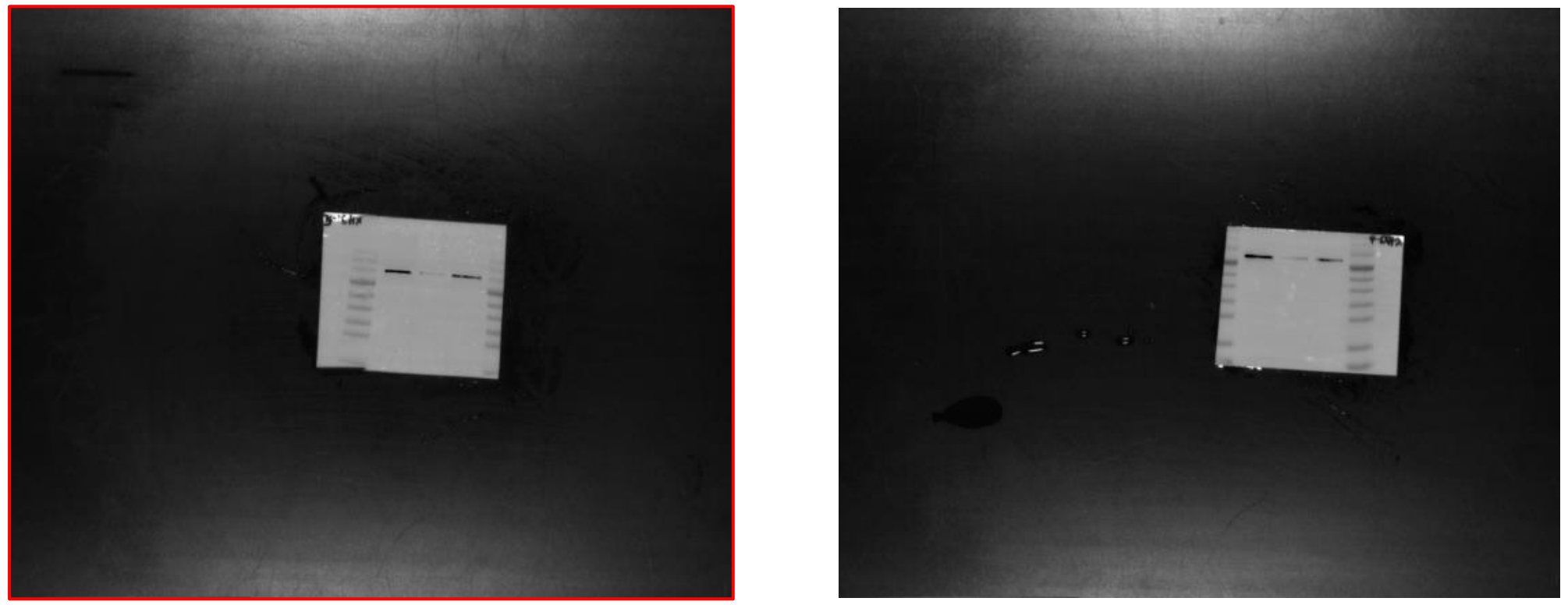

Figure 6E: Vimentin (three repeats)

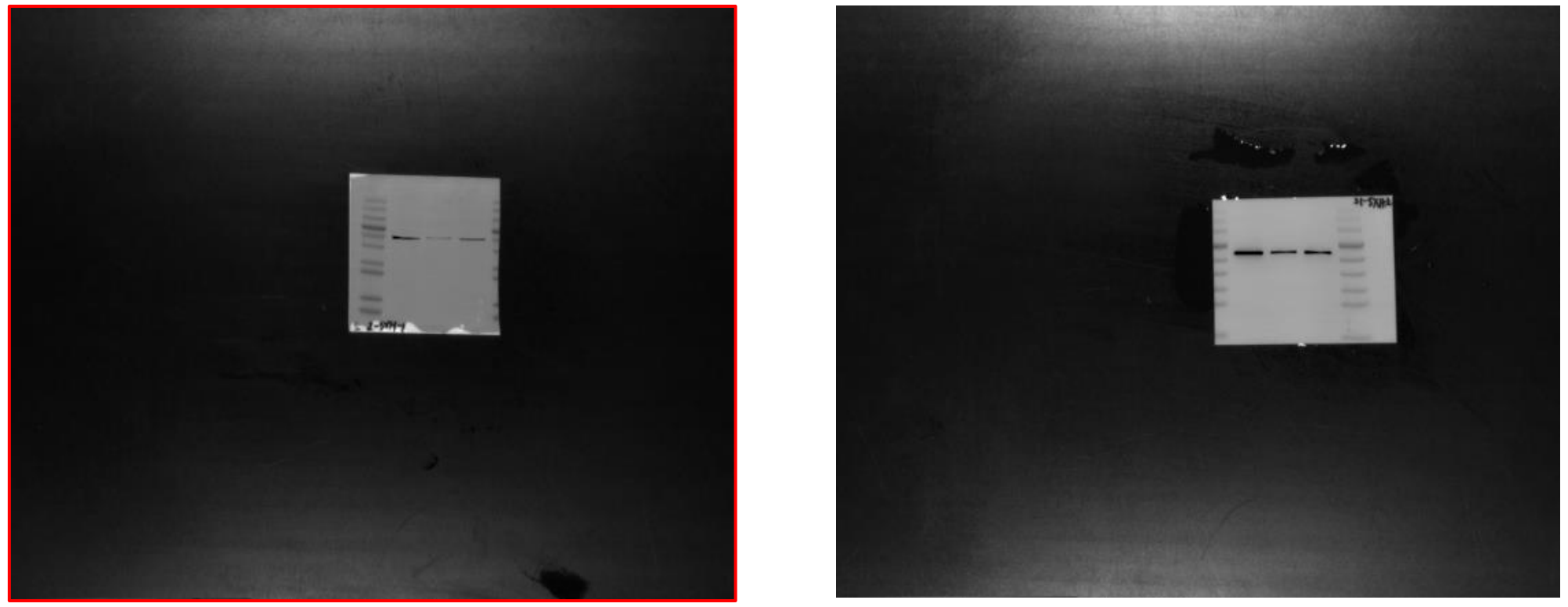

Figure 6E: SOX2 (three repeats)

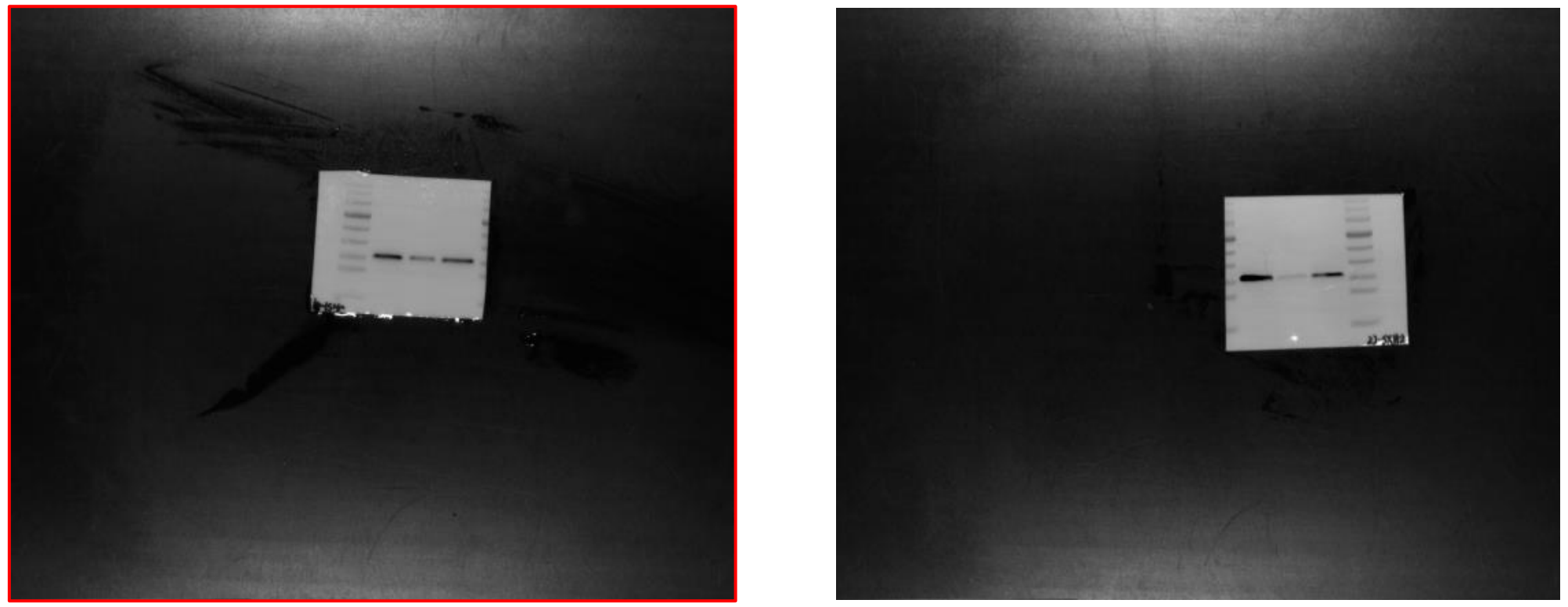

Figure 6E: NANOG (three repeats)

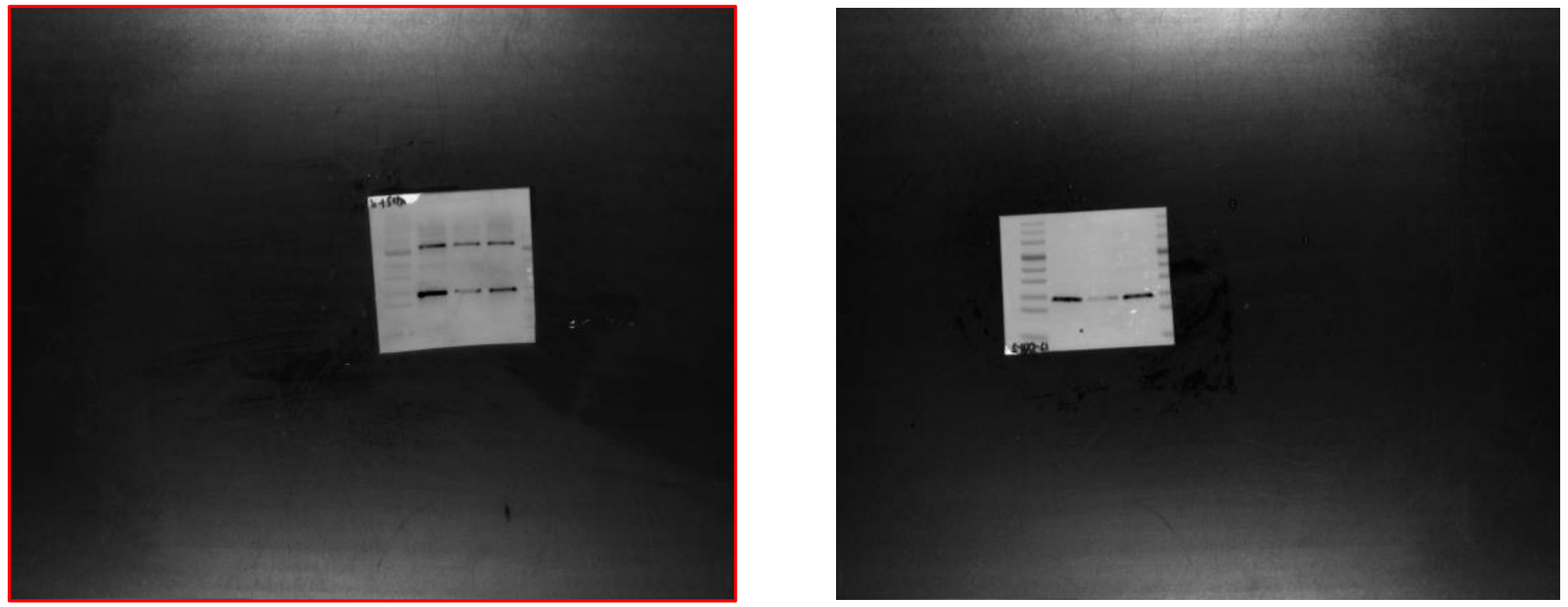

Figure 6E: SNAI1 (three repeats)

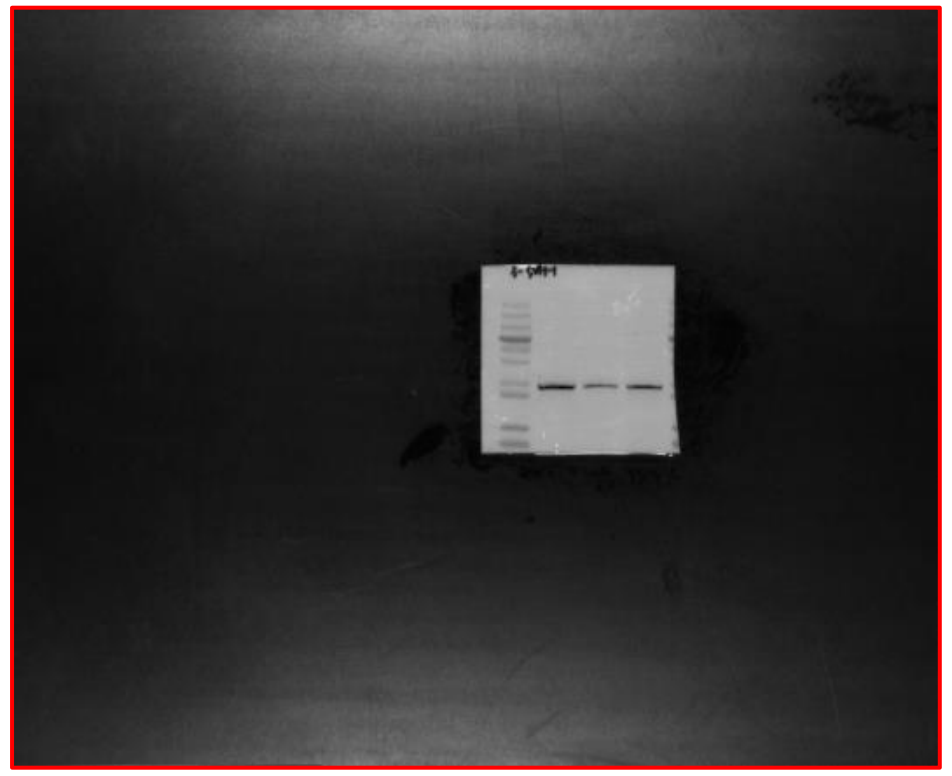

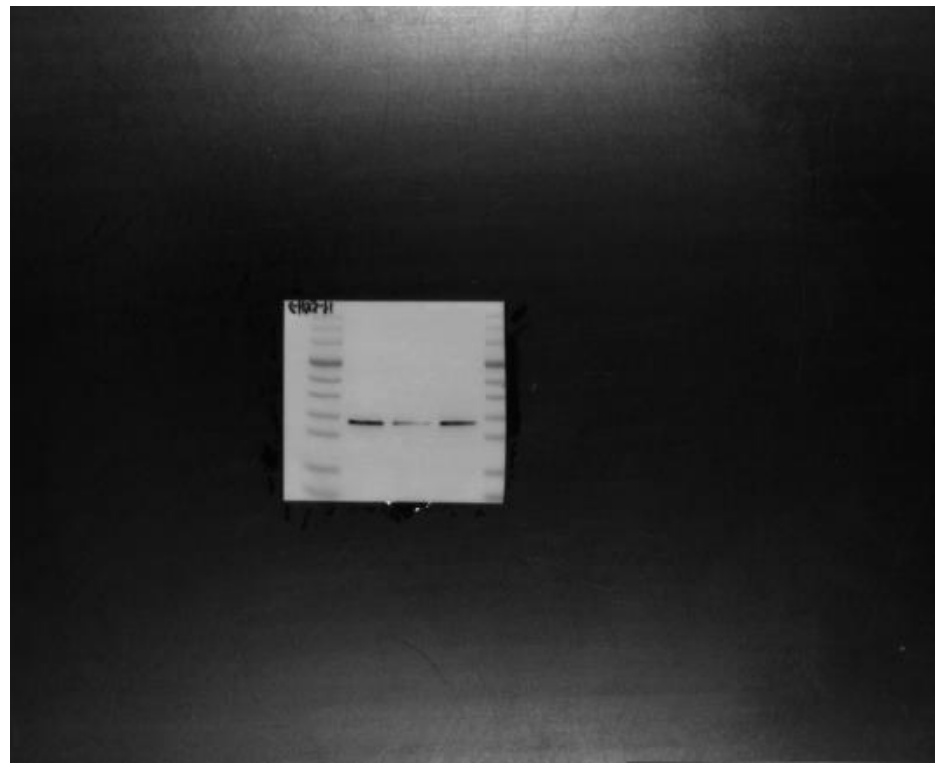

Figure 6E: GAPDH (three repeats)

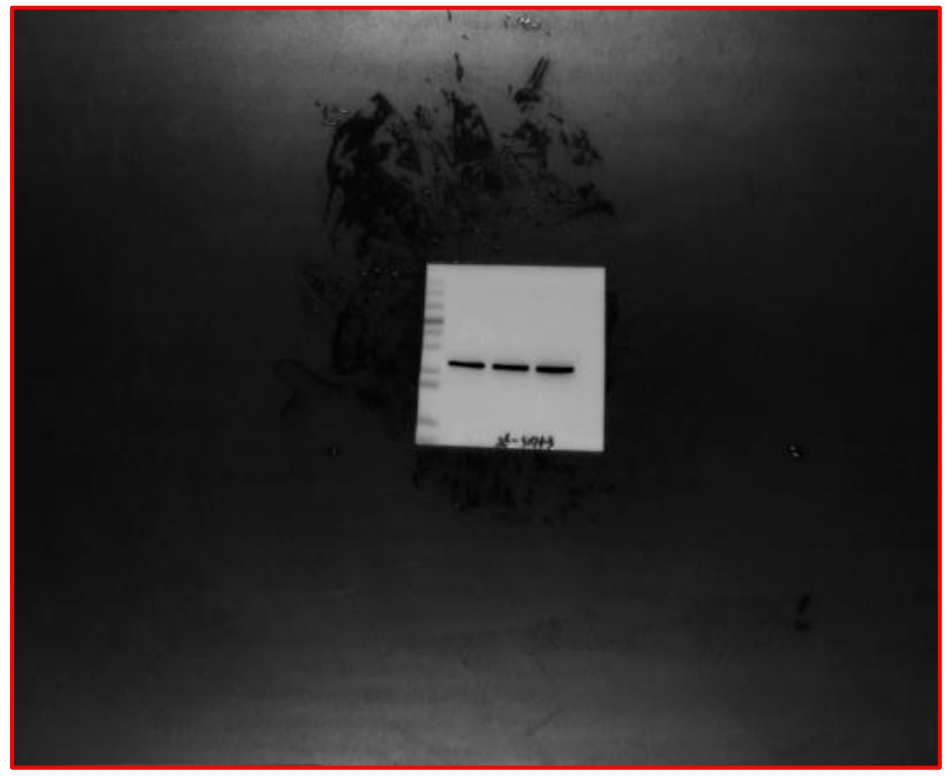

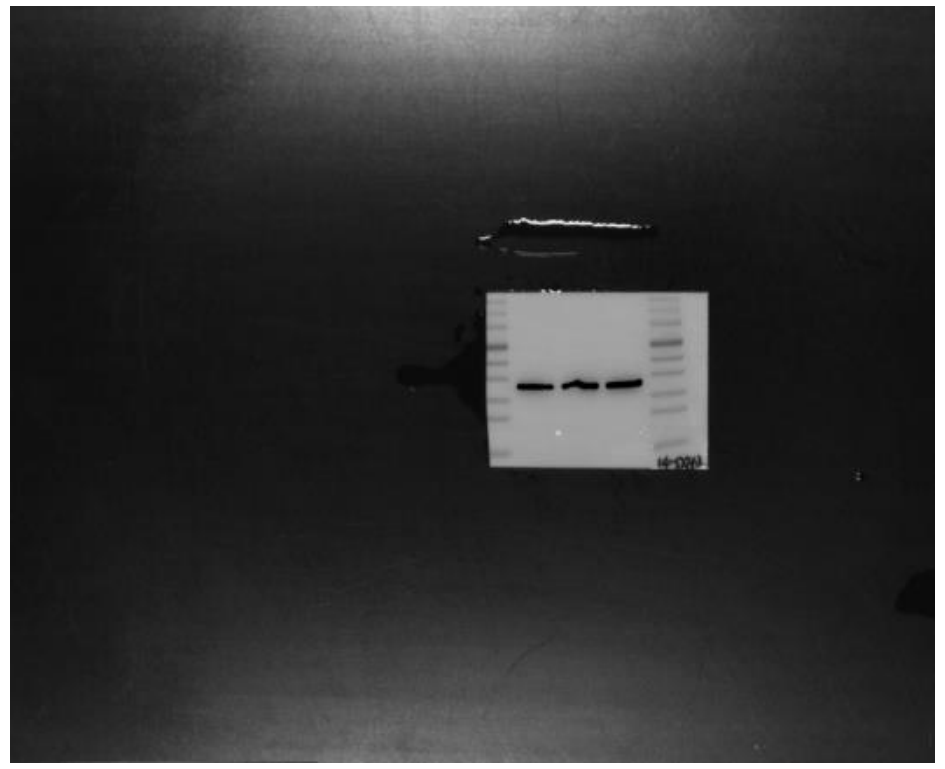

Figure 6F: E-cadherin (three repeats)

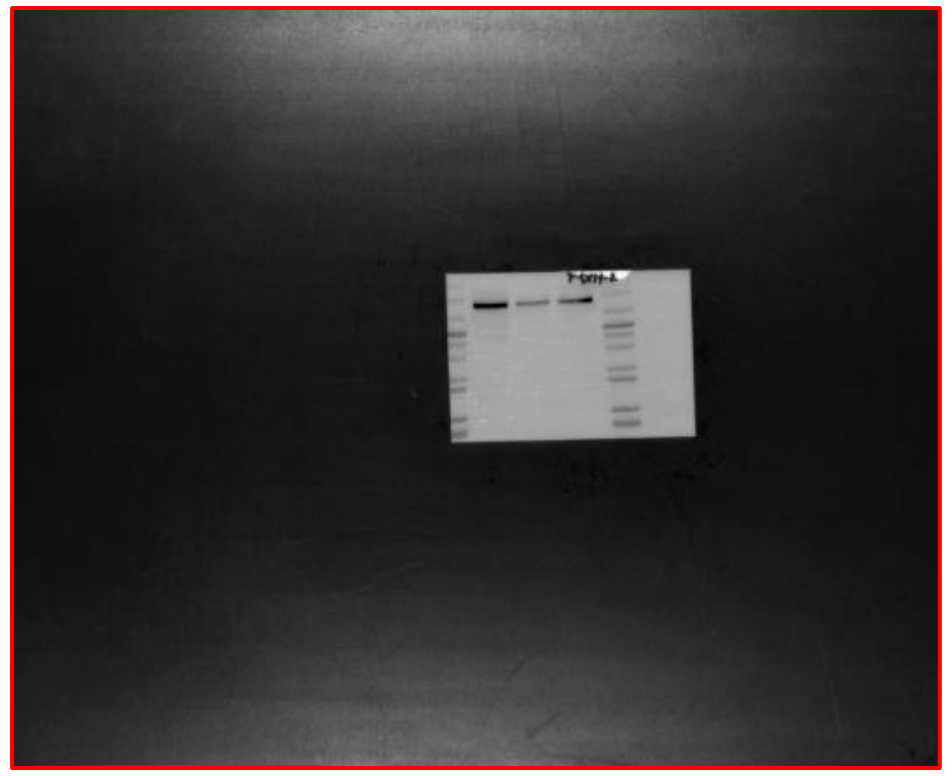

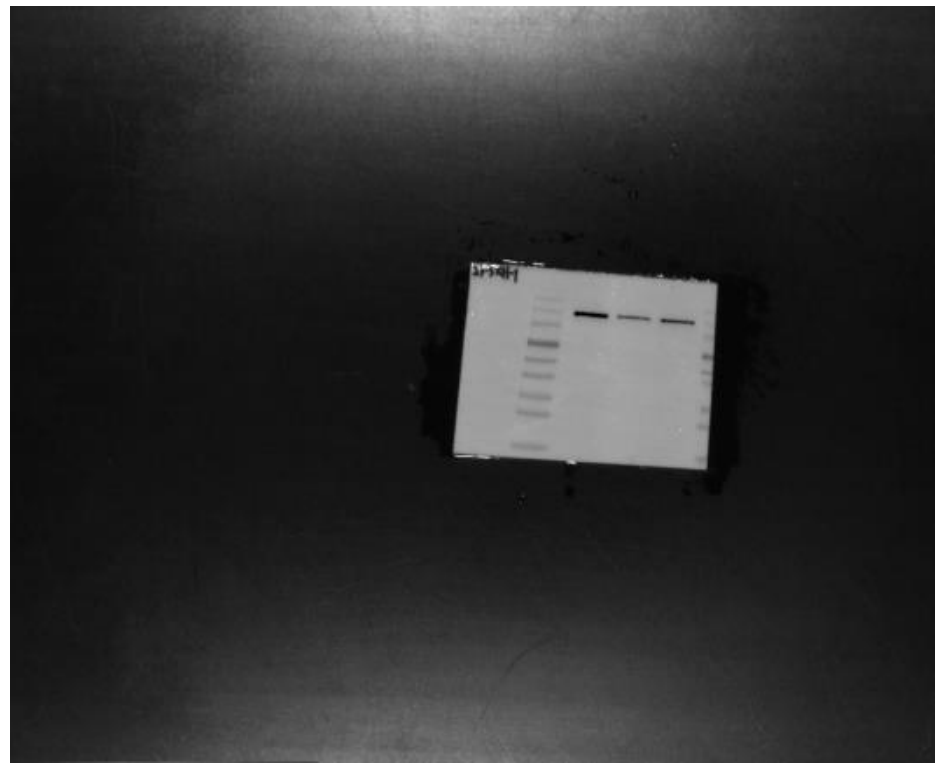

Figure 6F: N-cadherin (three repeats)

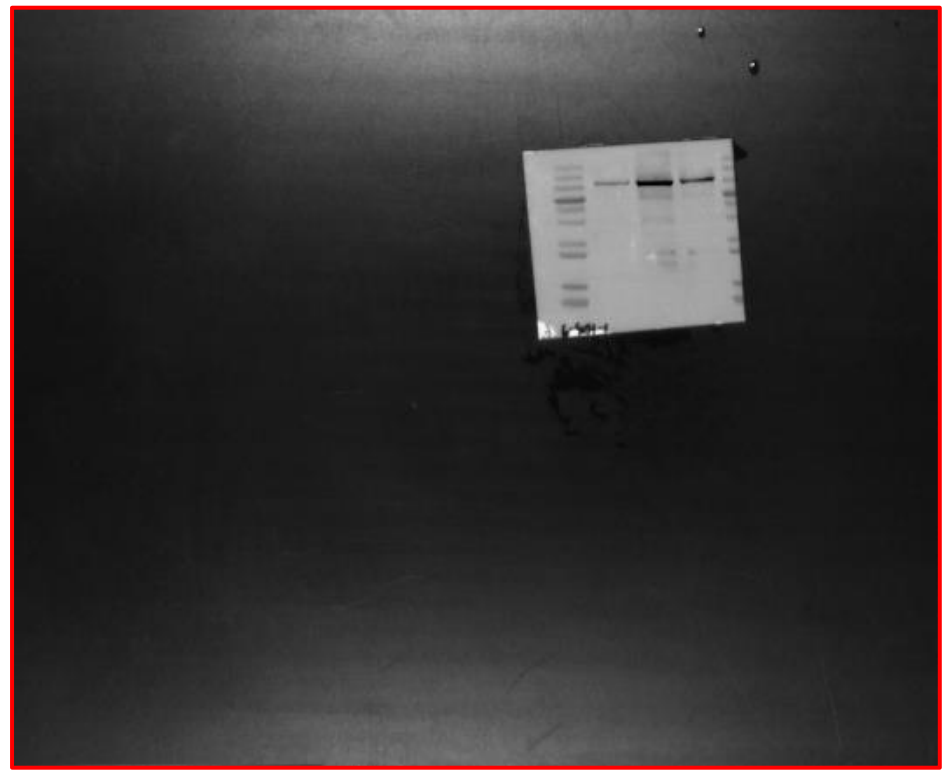

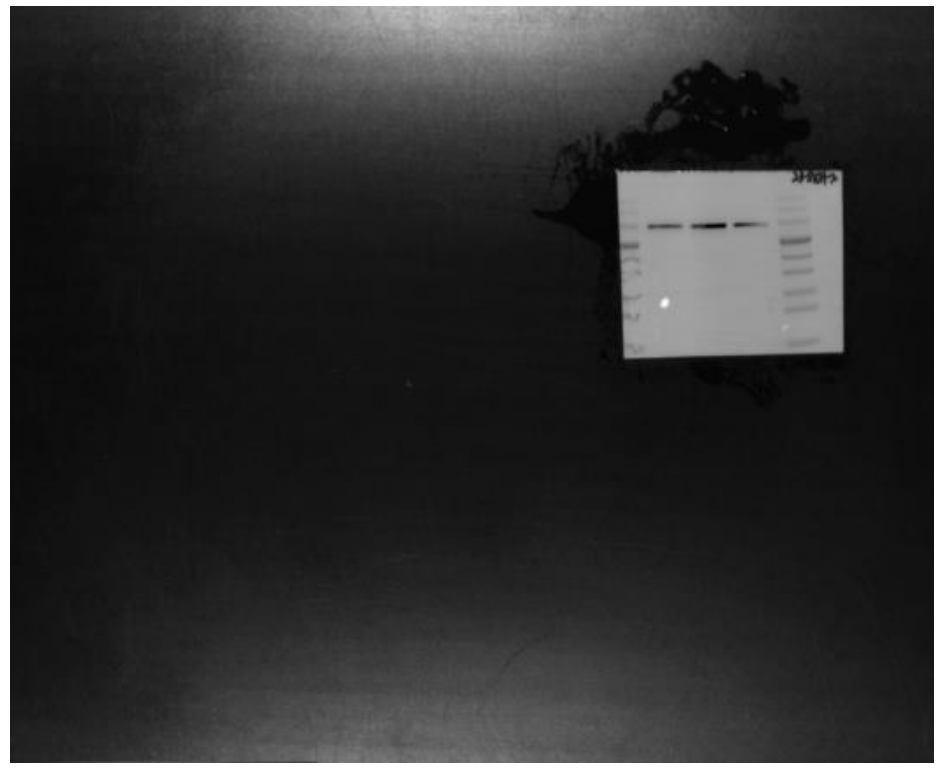

Figure 6F: CD44 (three repeats)

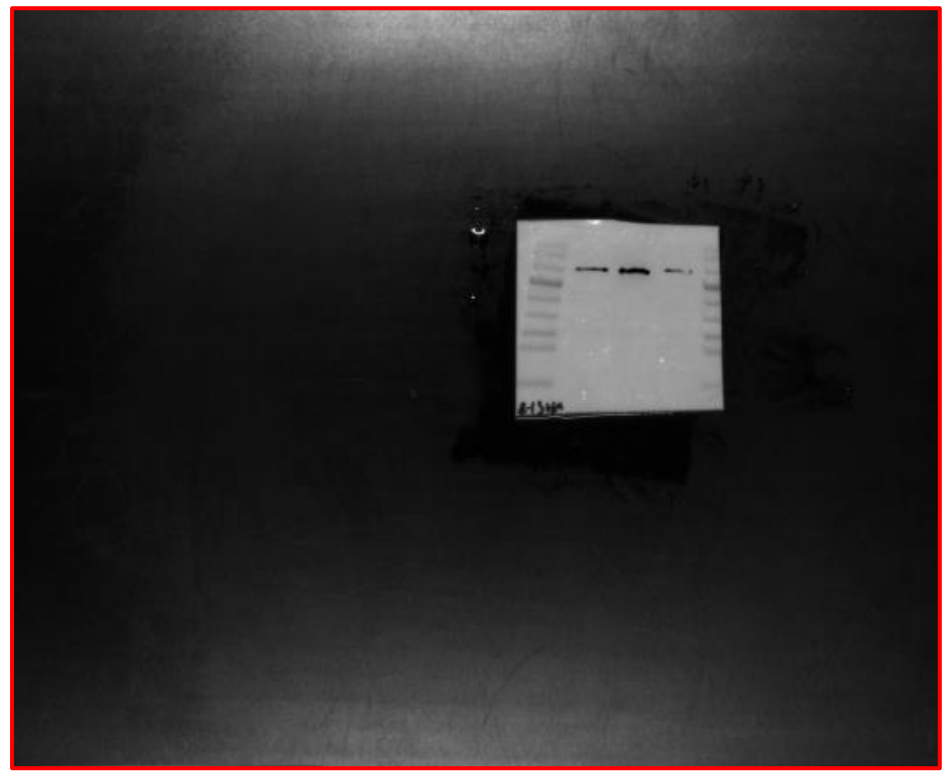

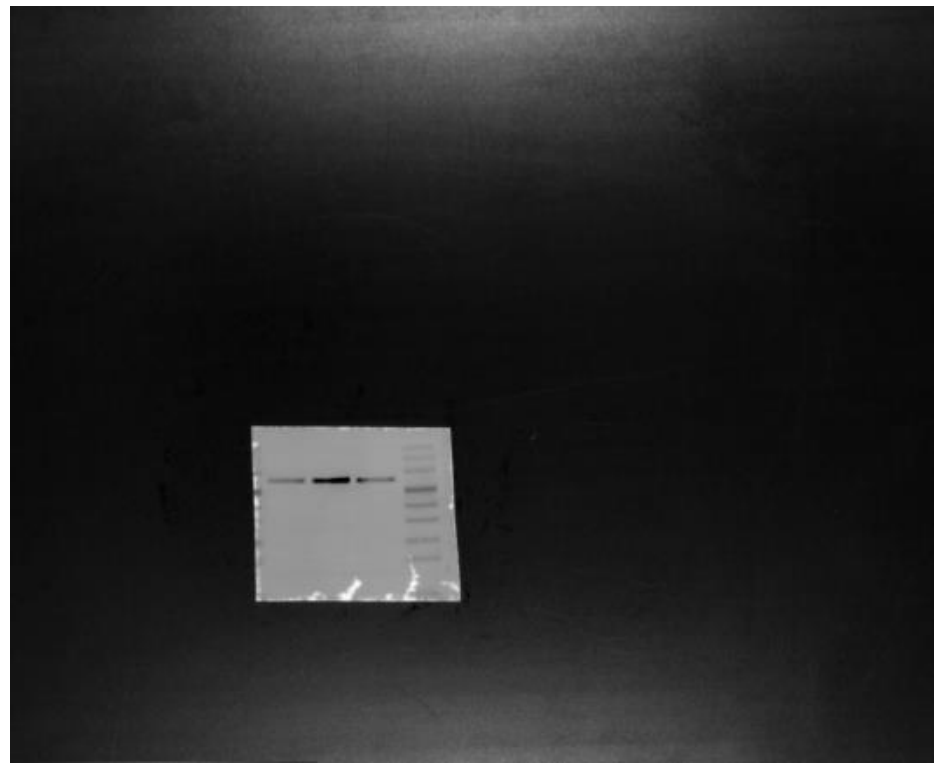

Figure 6F: Vimentin (three repeats)

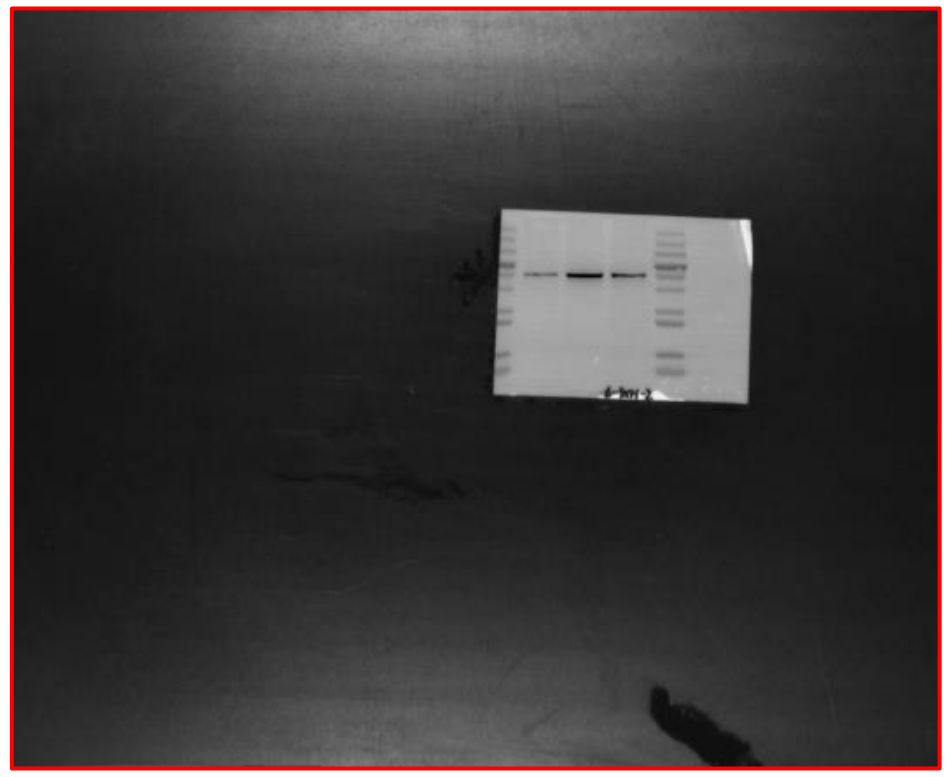

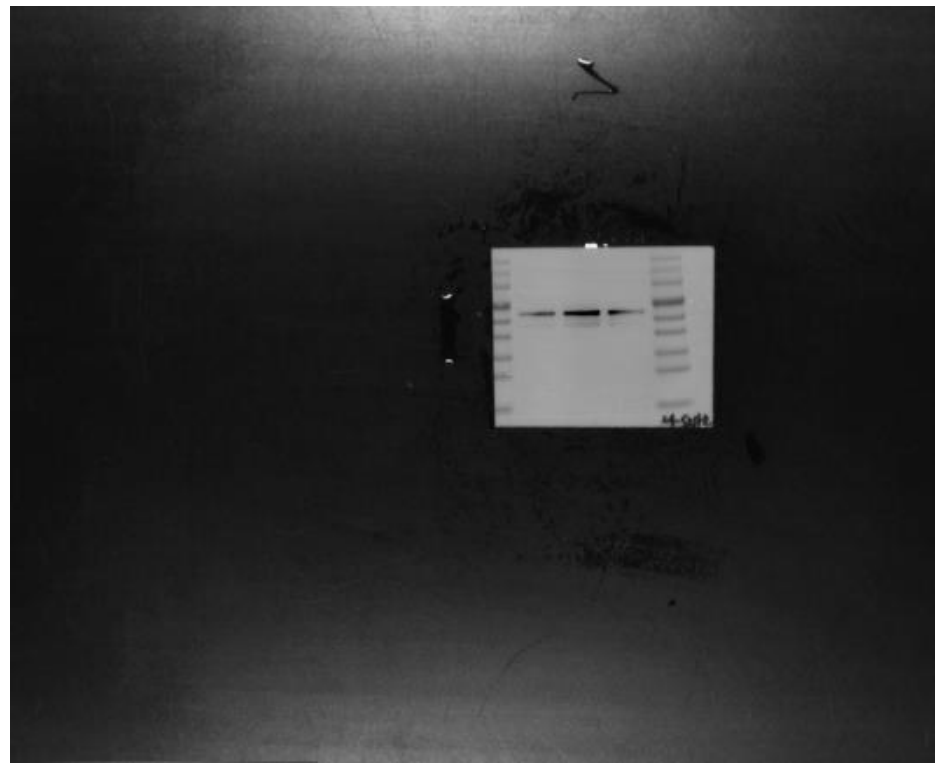

Figure 6F: SOX2 (three repeats)

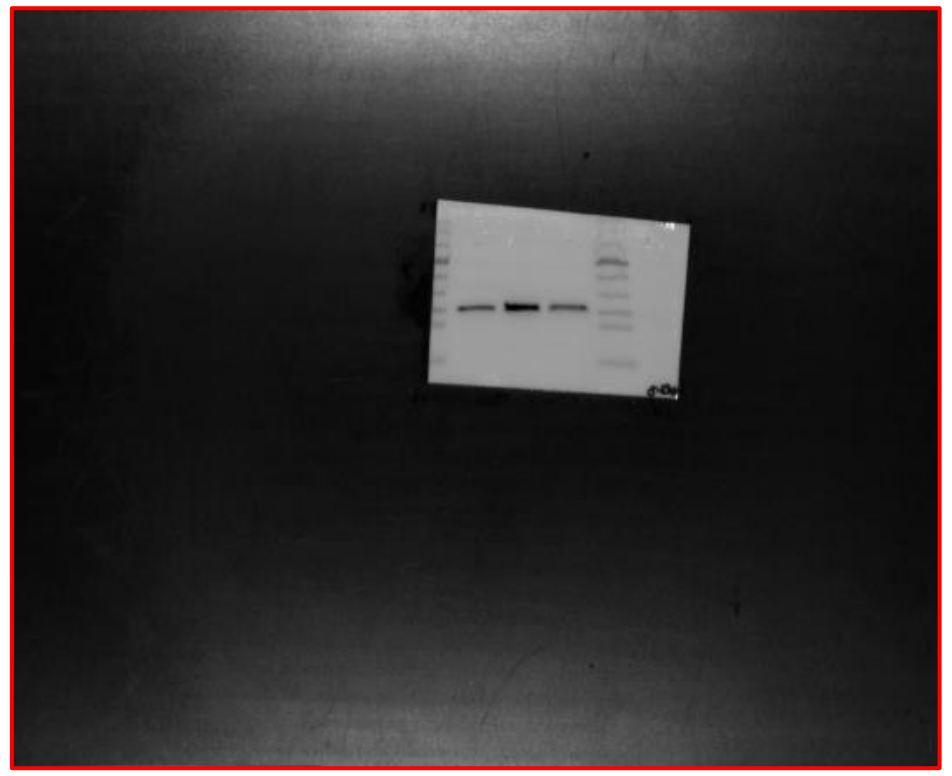

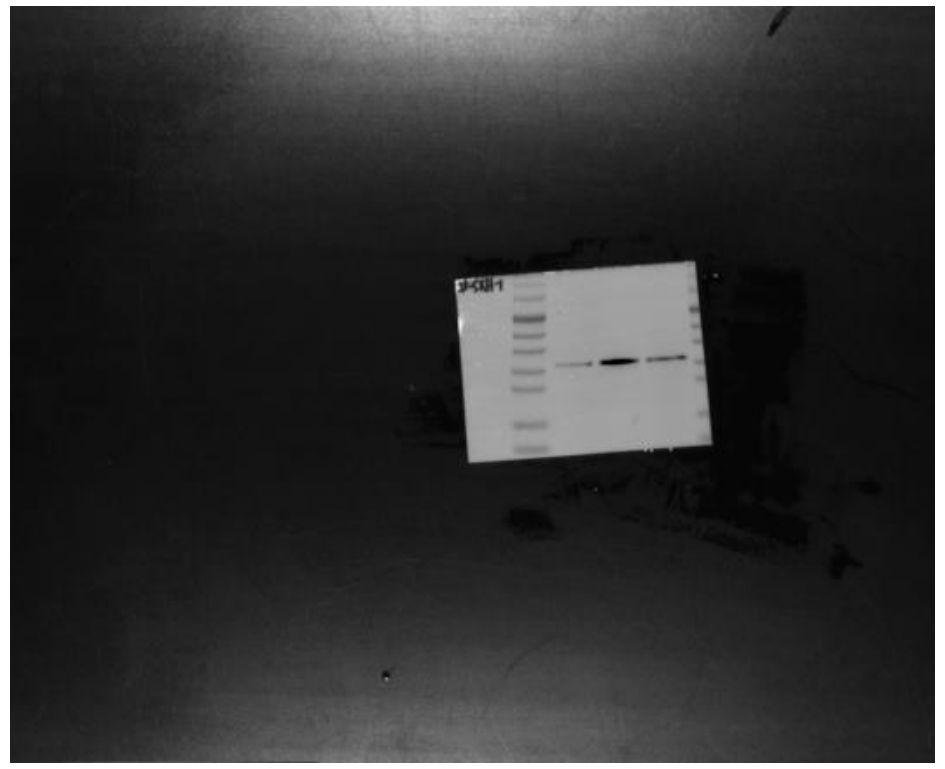

Figure 6F: NANOG (three repeats)

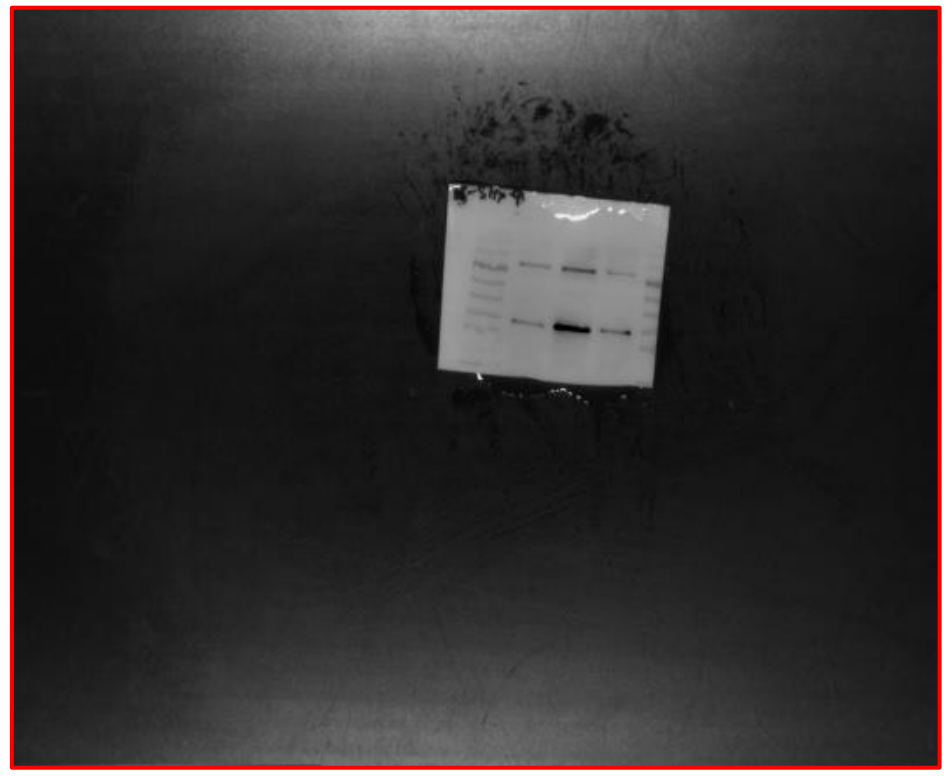

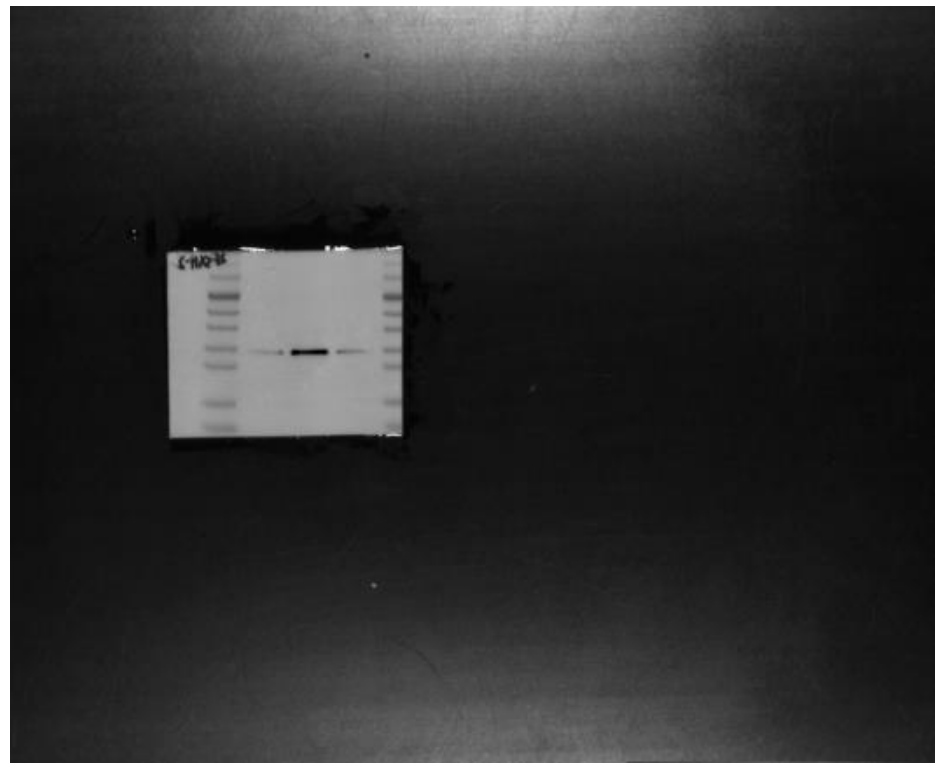

Figure 6F: SNAI1 (three repeats)

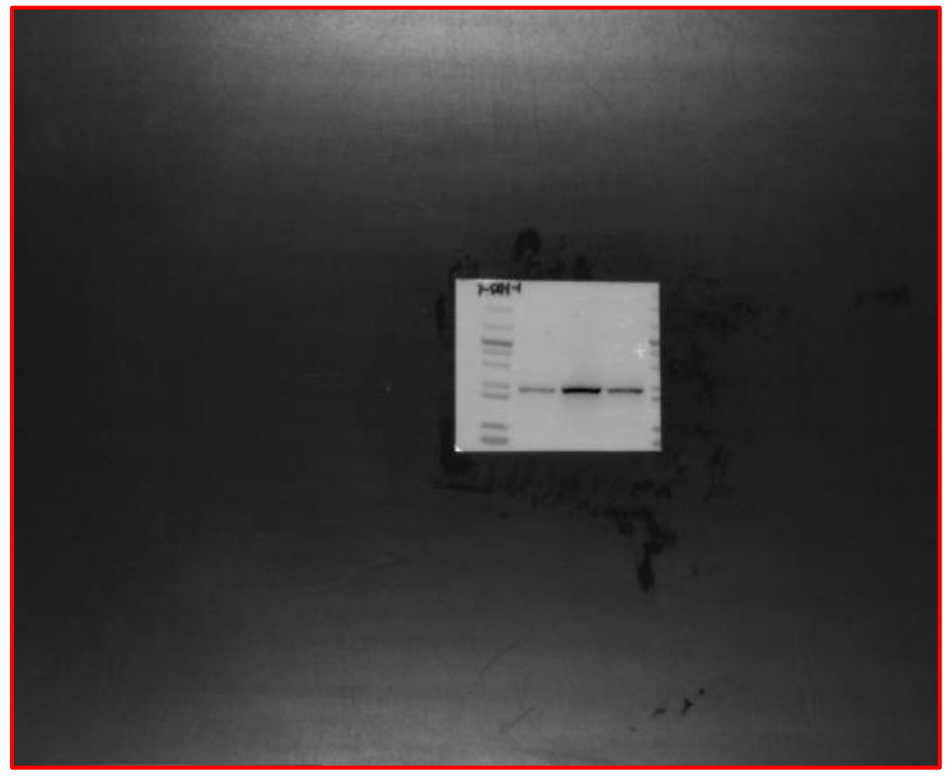

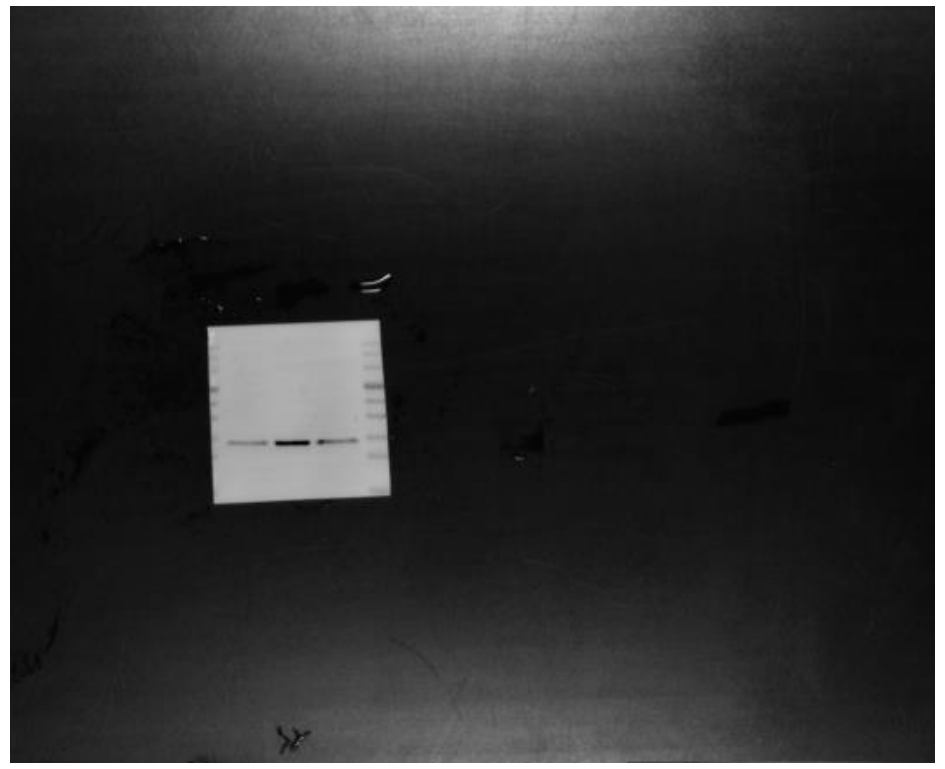

Figure 6F: GAPDH (three repeats)

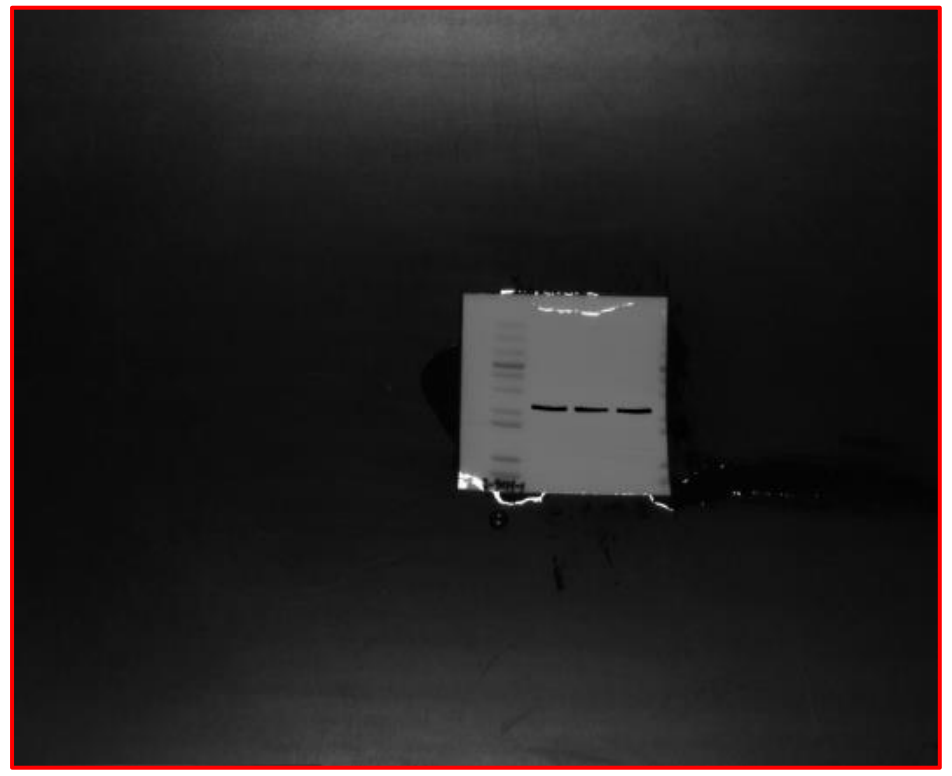

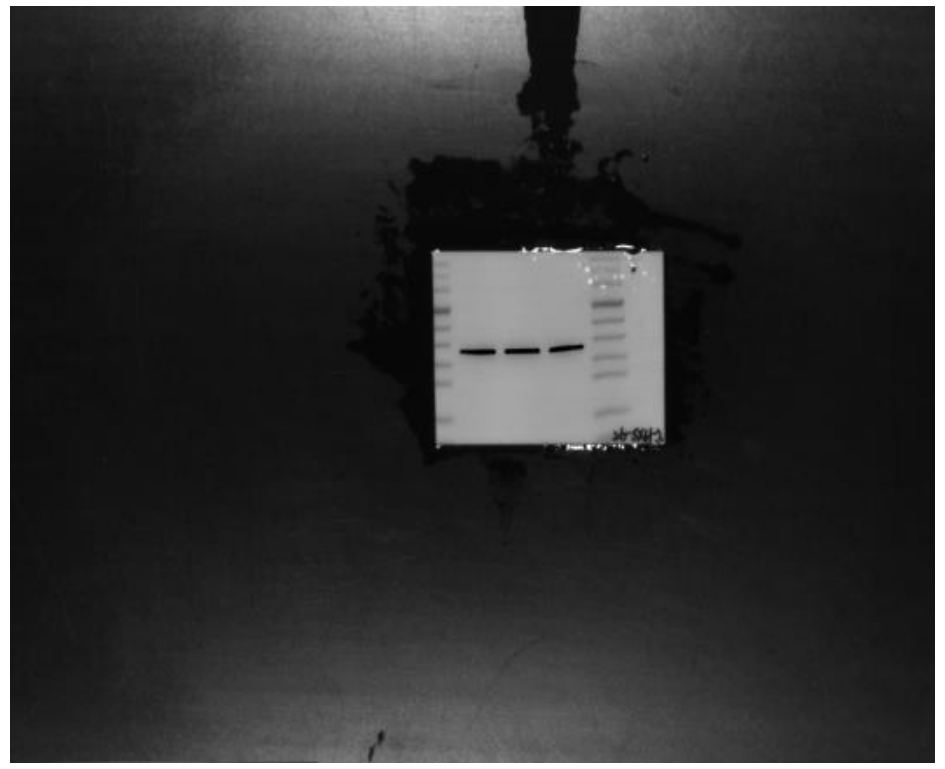

Figure S2D: ARNTL2 (three repeats)

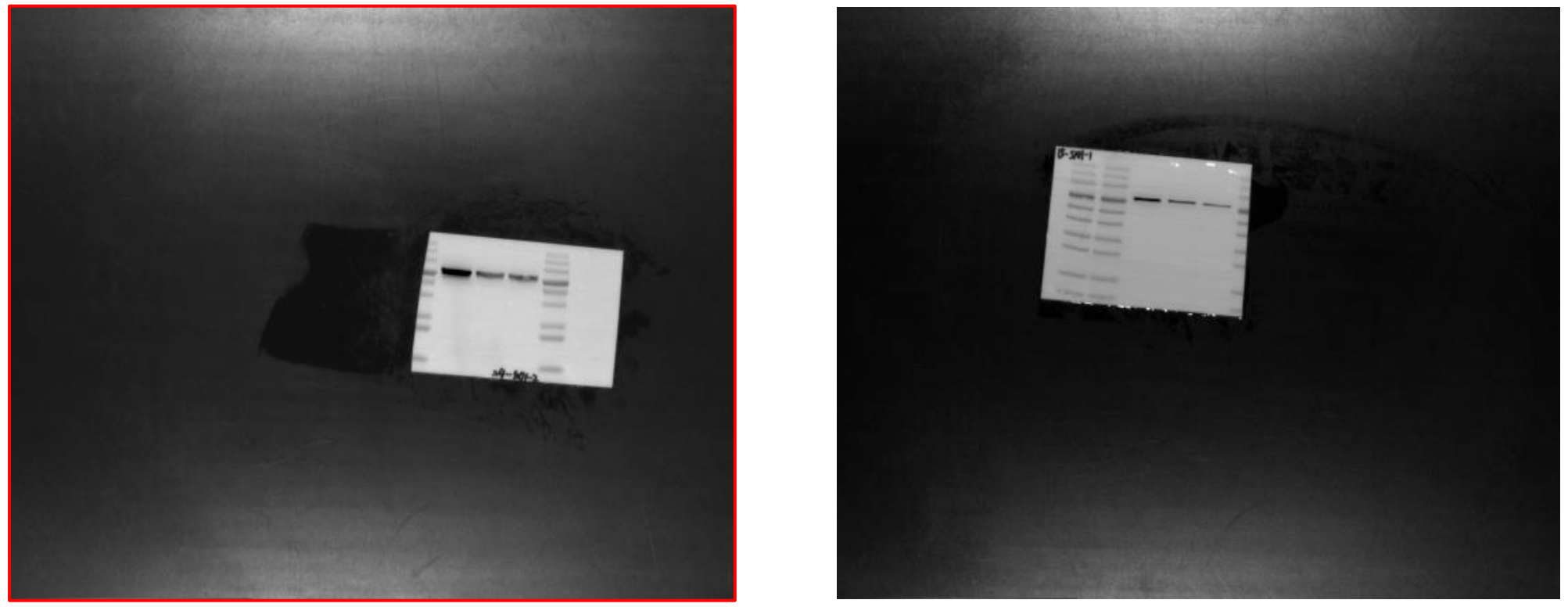

Figure S2E: ARNTL2 (three repeats)

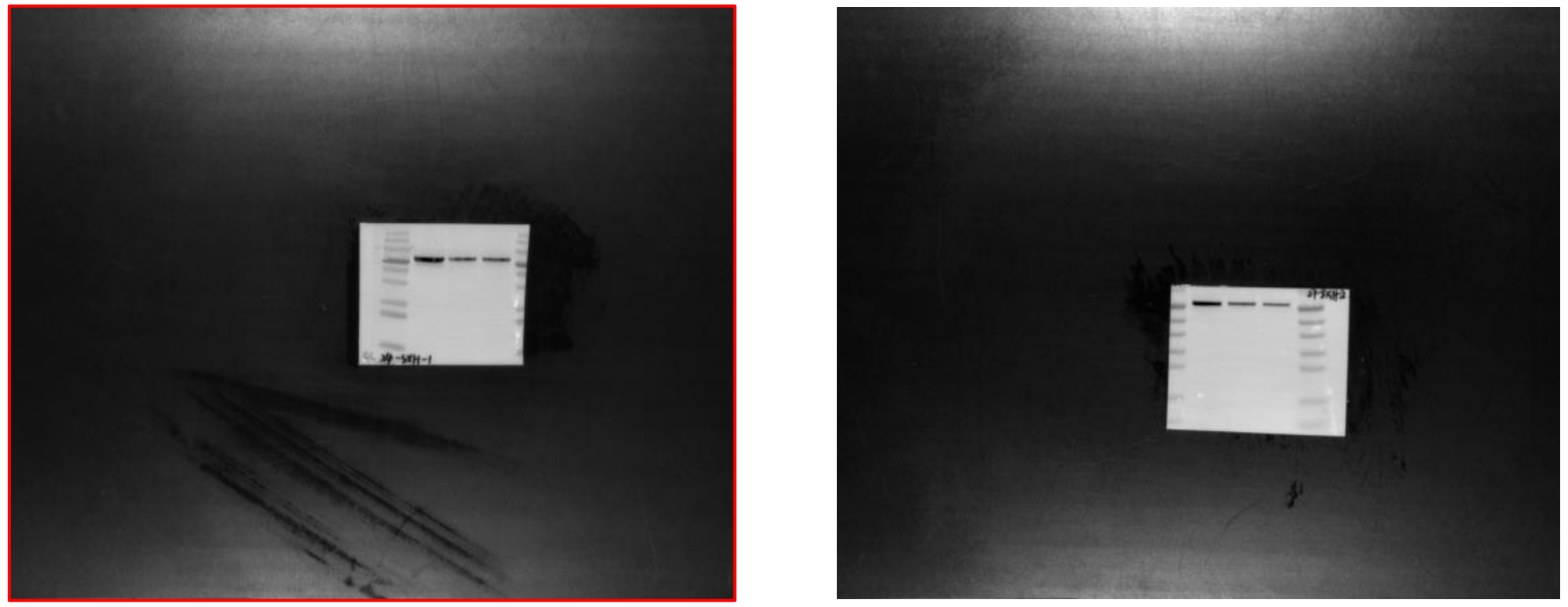

Figure S2D: GAPDH (three repeats)

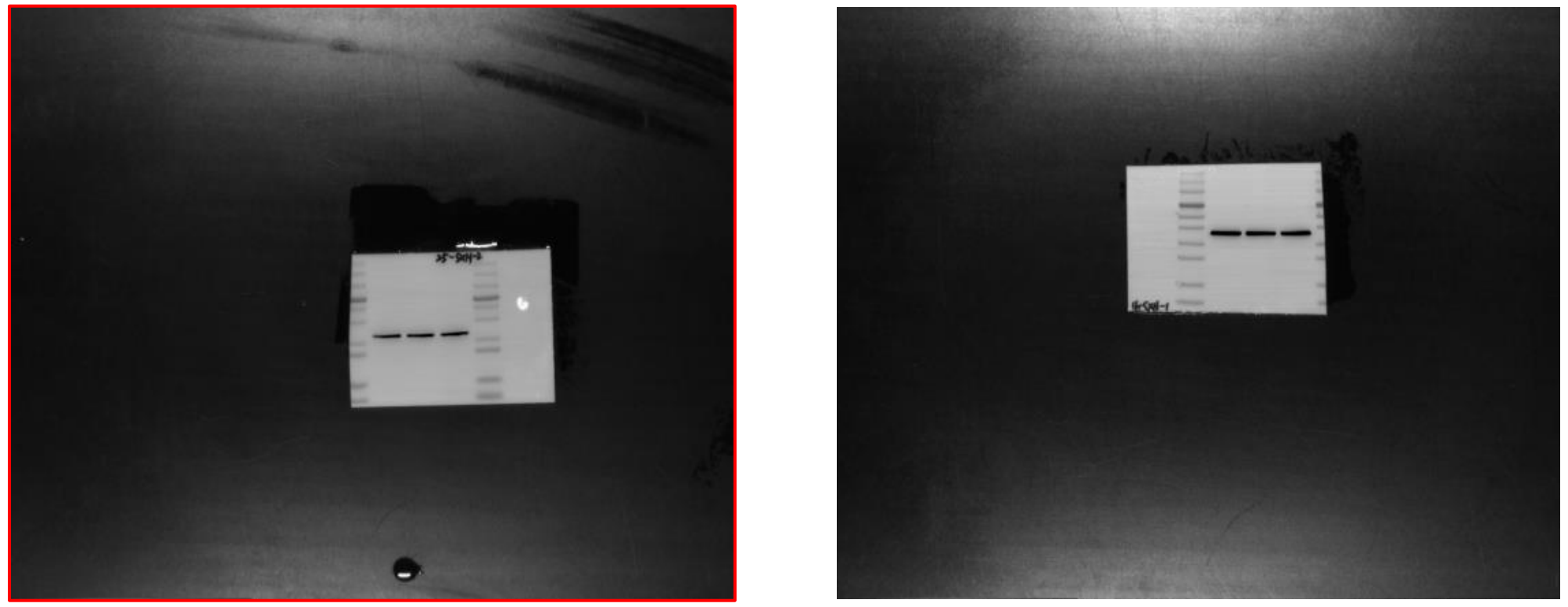

Figure S2E: GAPDH (three repeats)

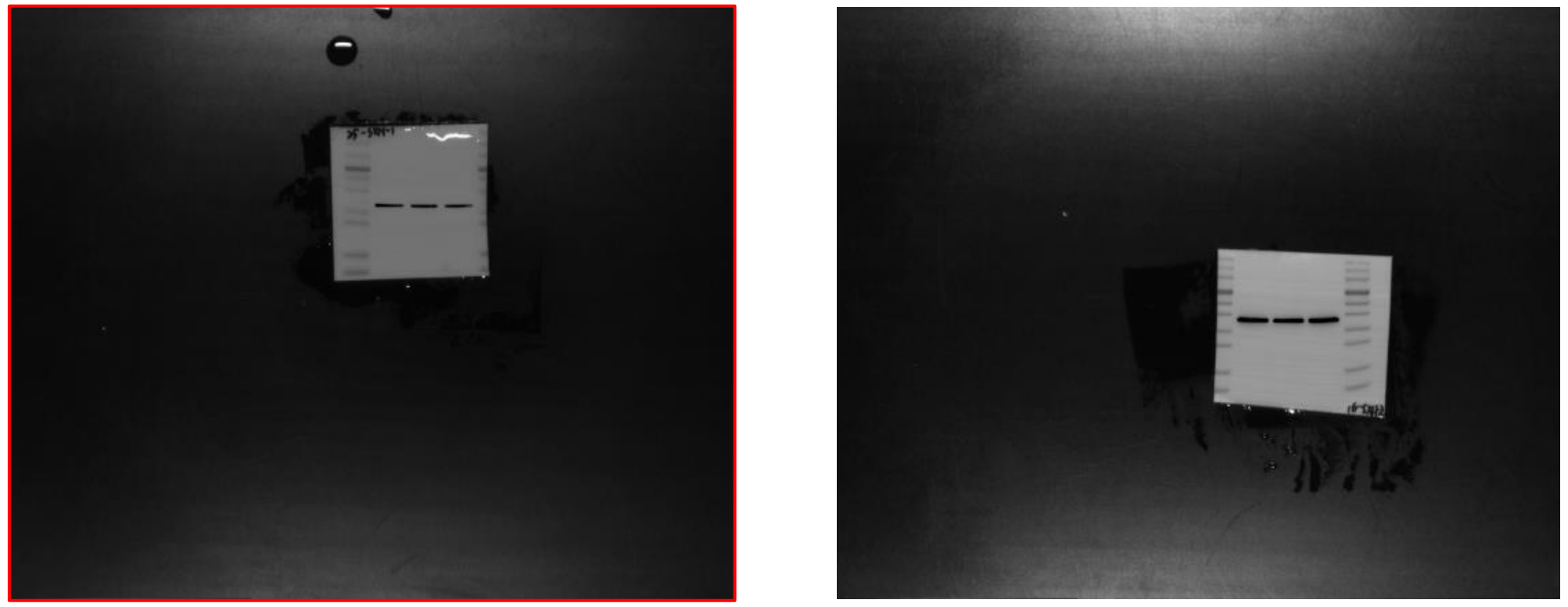

Figure S2F: ARNTL2 (three repeats)

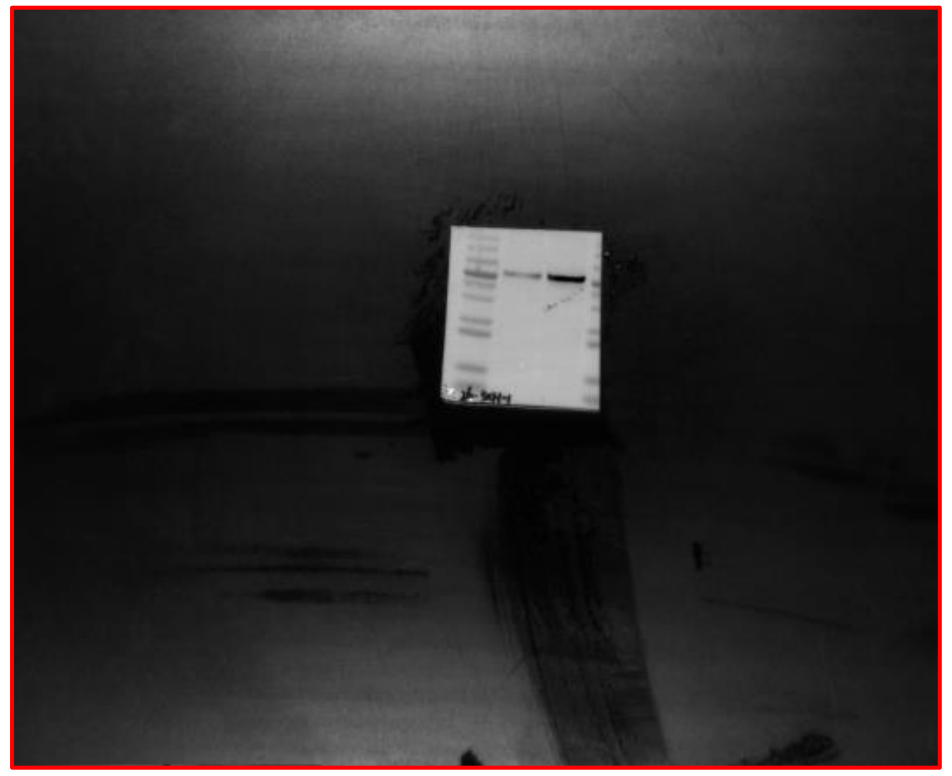

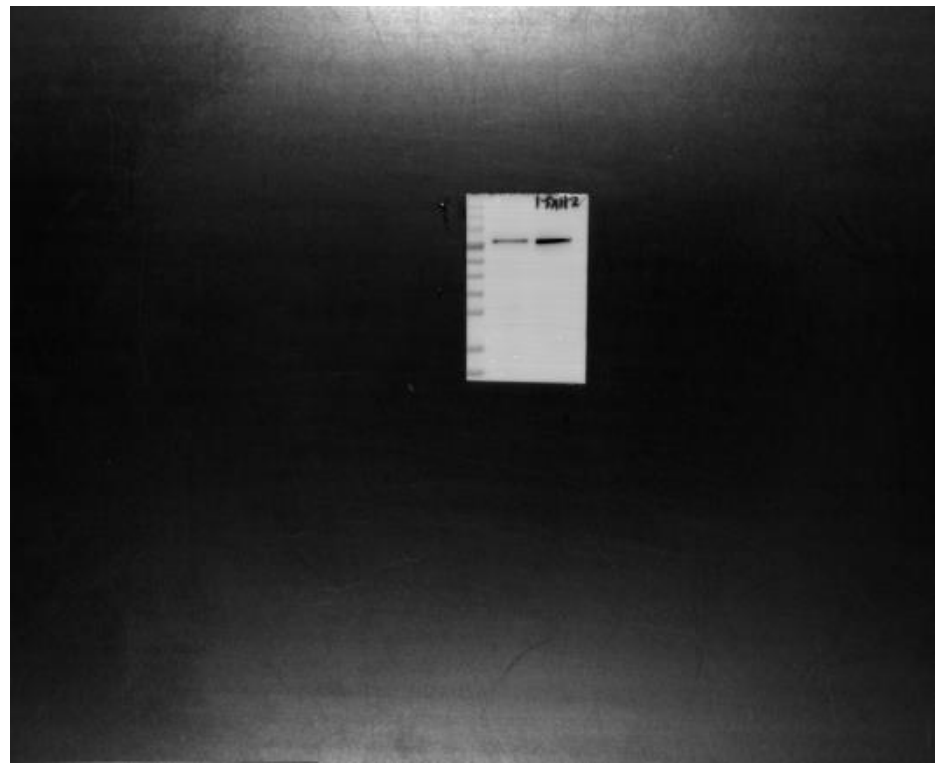

Figure S2F: GAPDH (three repeats)

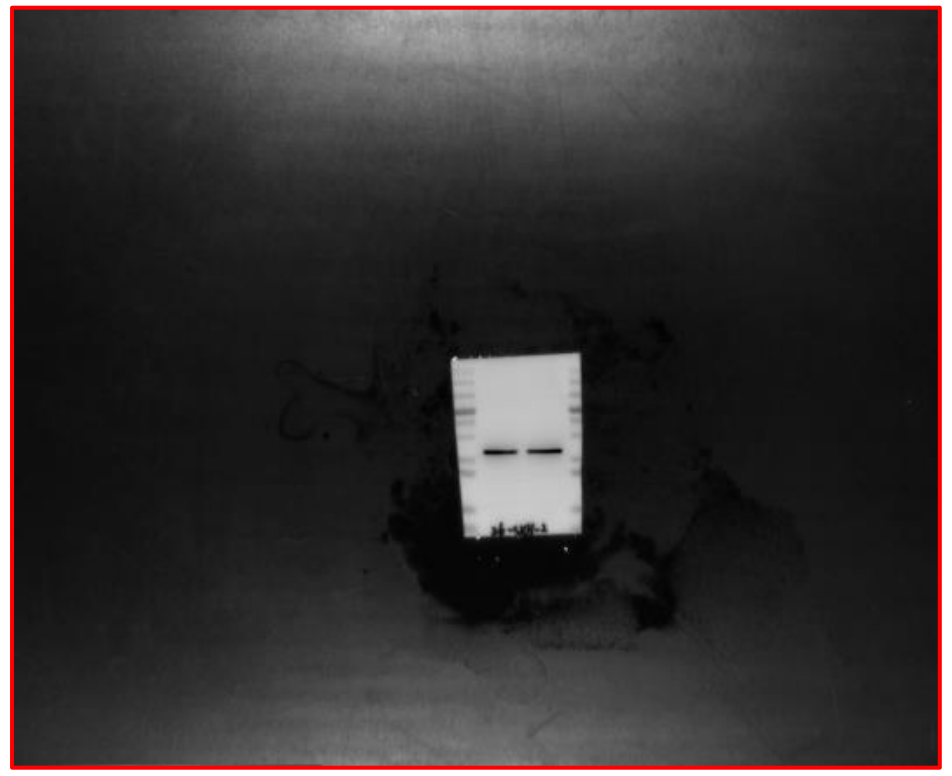

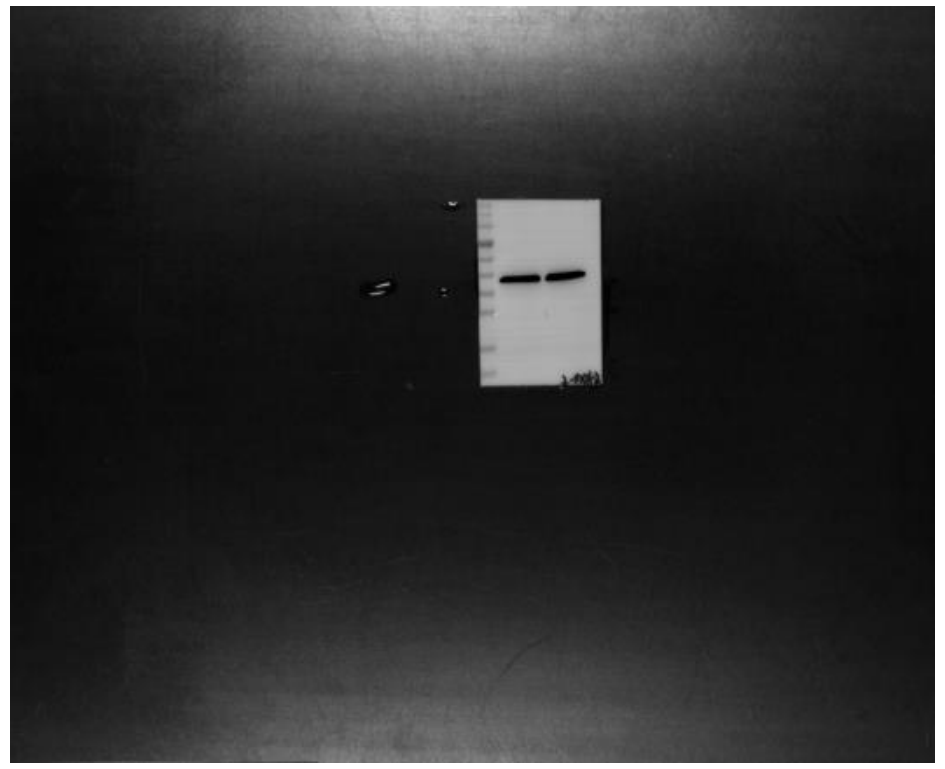

Figure S4G: SERPINE1 (three repeats)

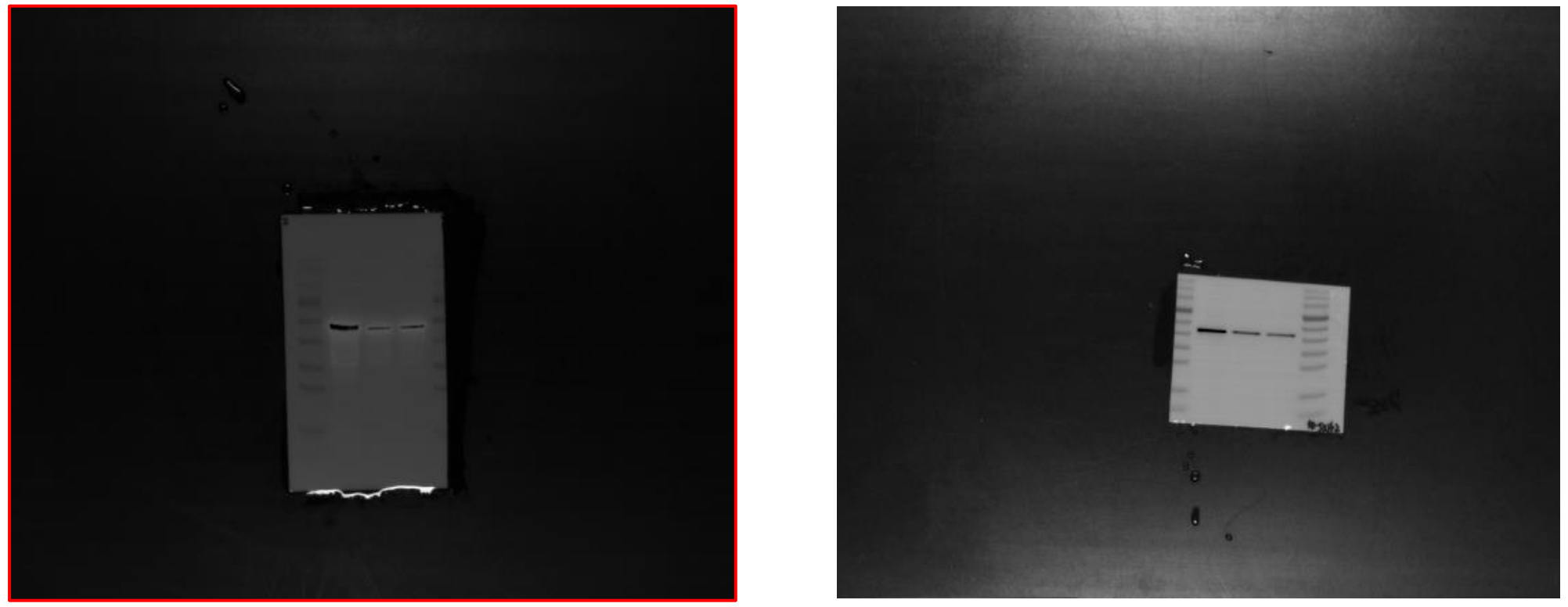

Figure S4G: GAPDH (three repeats)

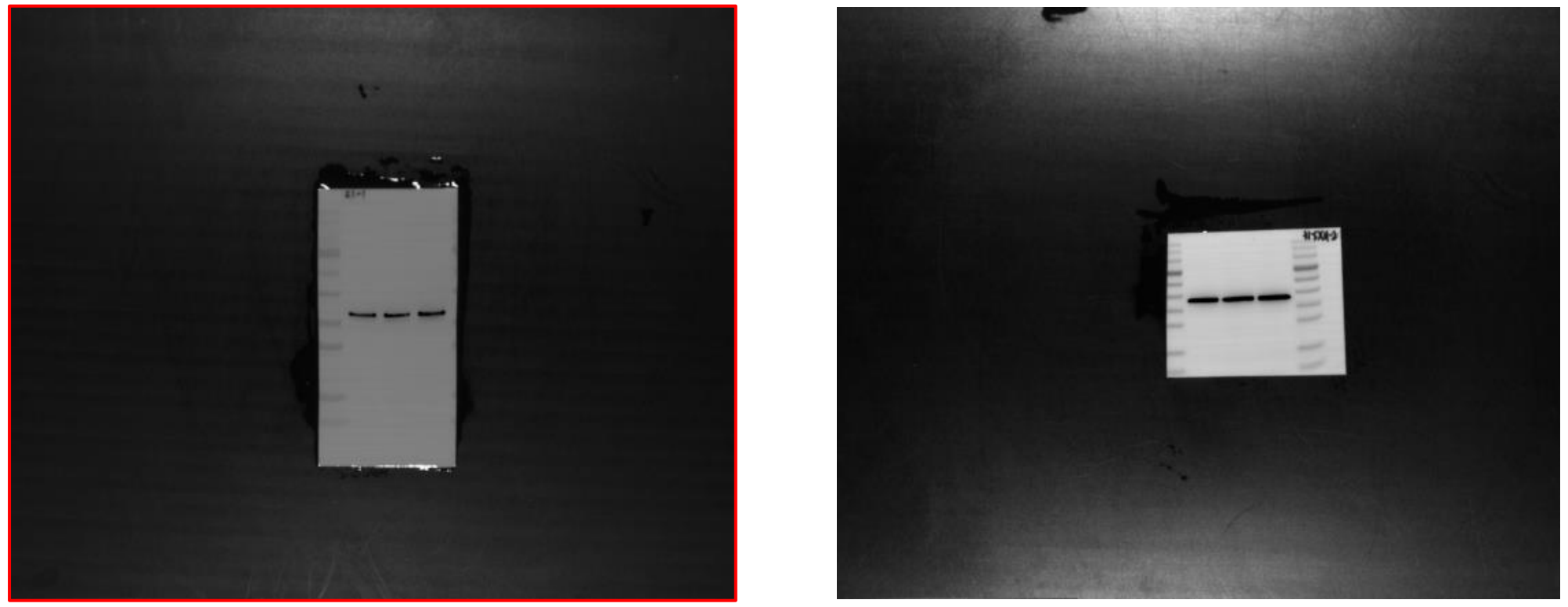

Figure S4H: SERPINE1 (three repeats)

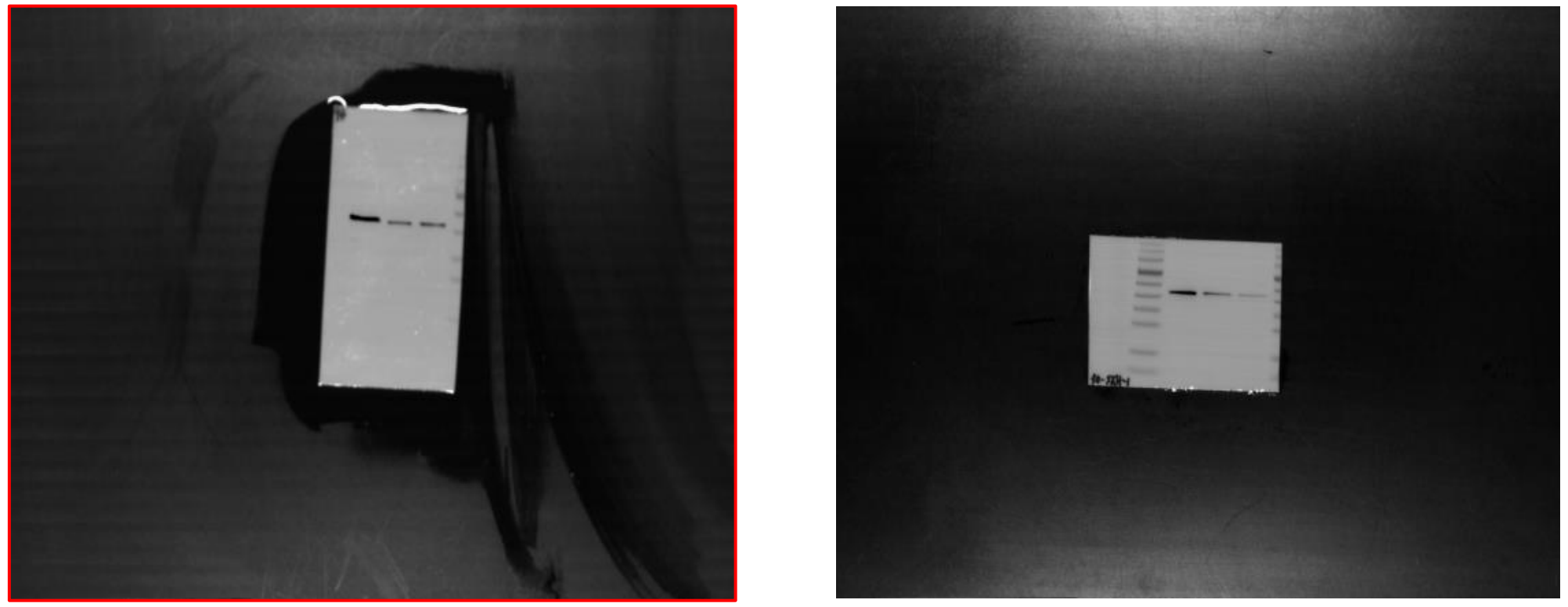

Figure S4H: GAPDH (three repeats)

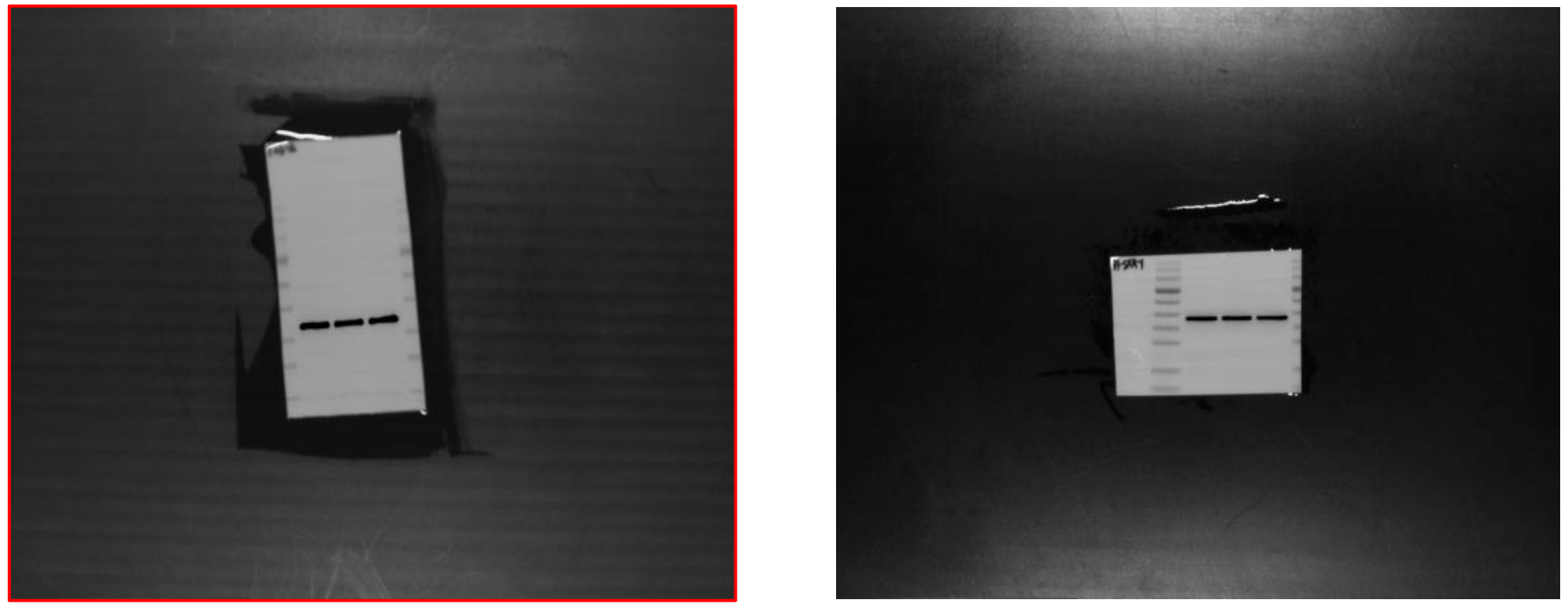

Figure S4I: SERPINE1 (three repeats)

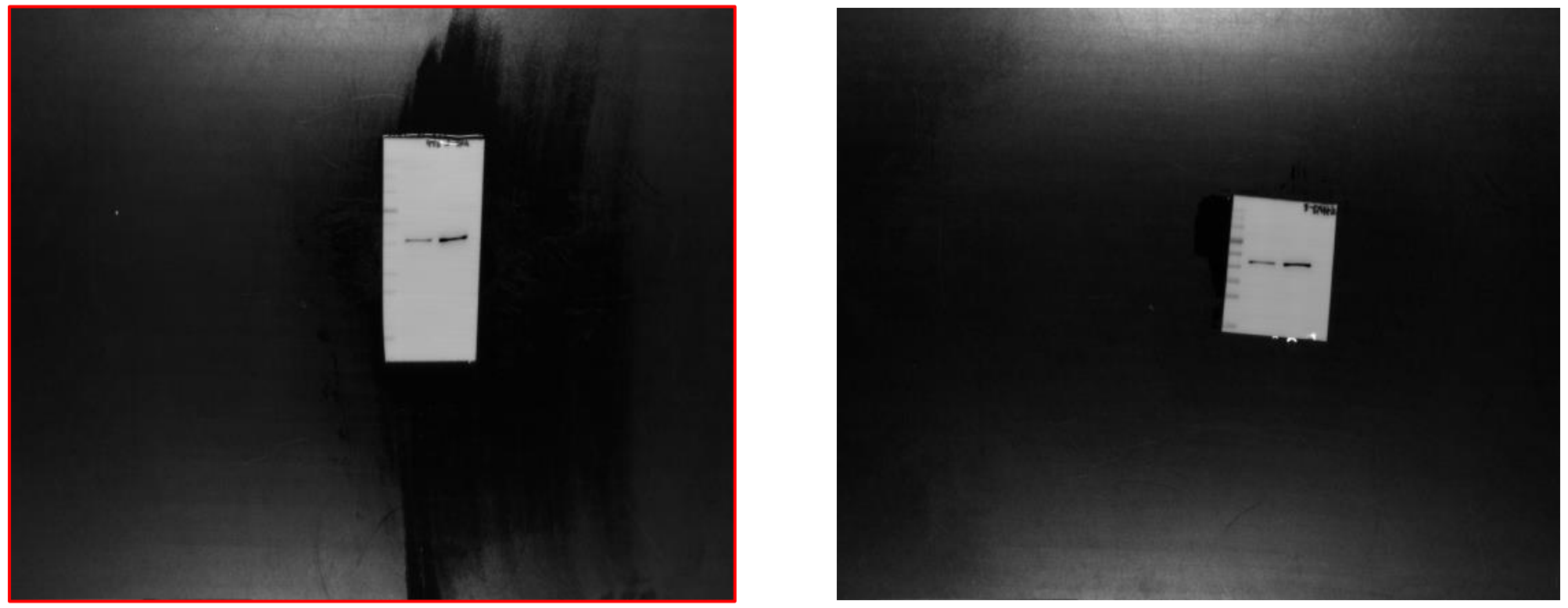

Figure S4I: GAPDH (three repeats)

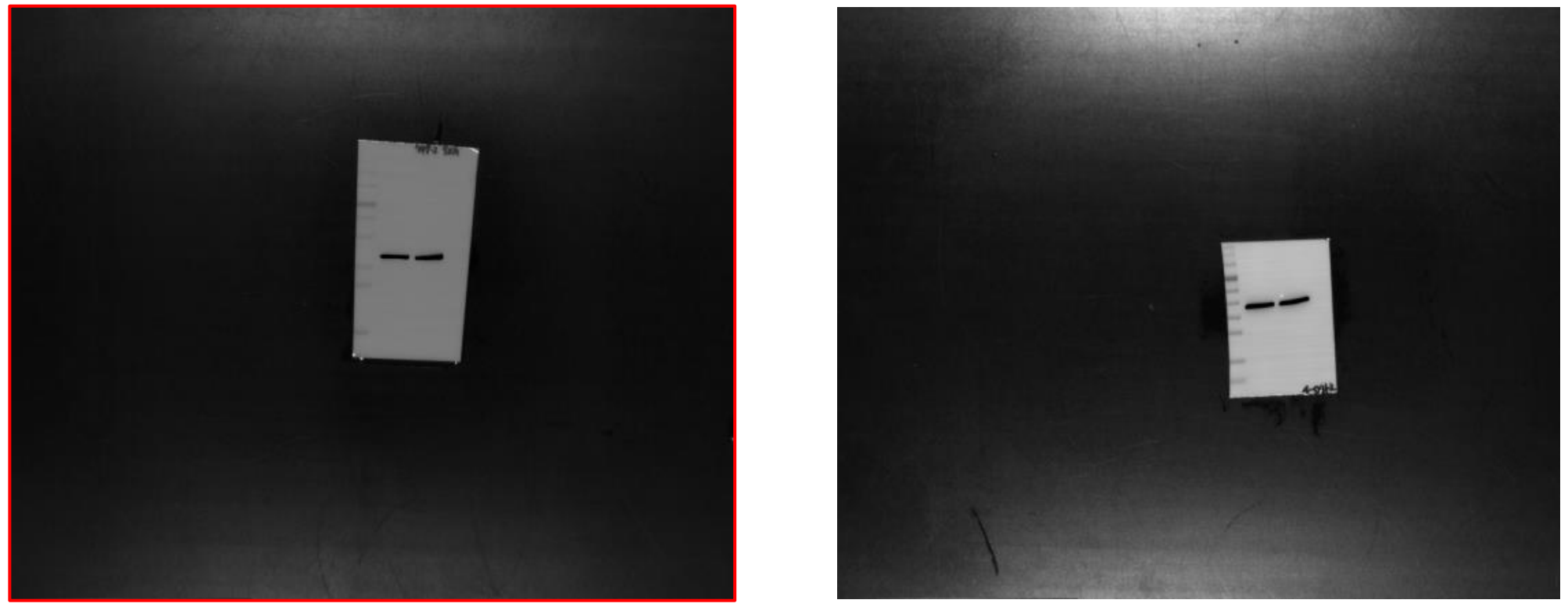

Figure S6A PANC-1: SERPINE1 (three repeats)

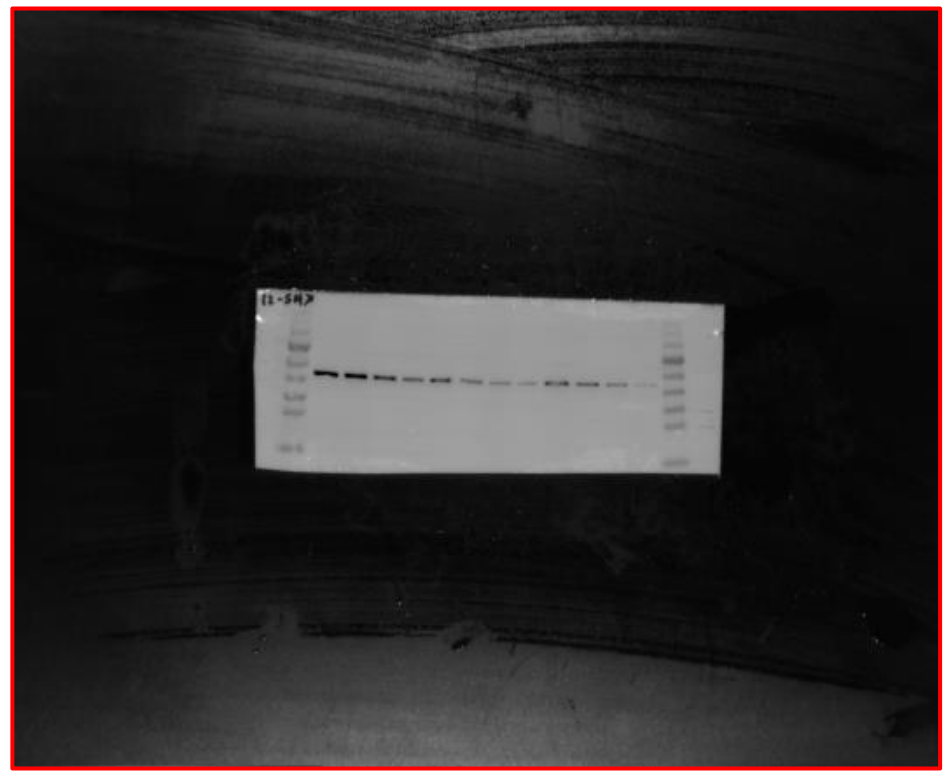

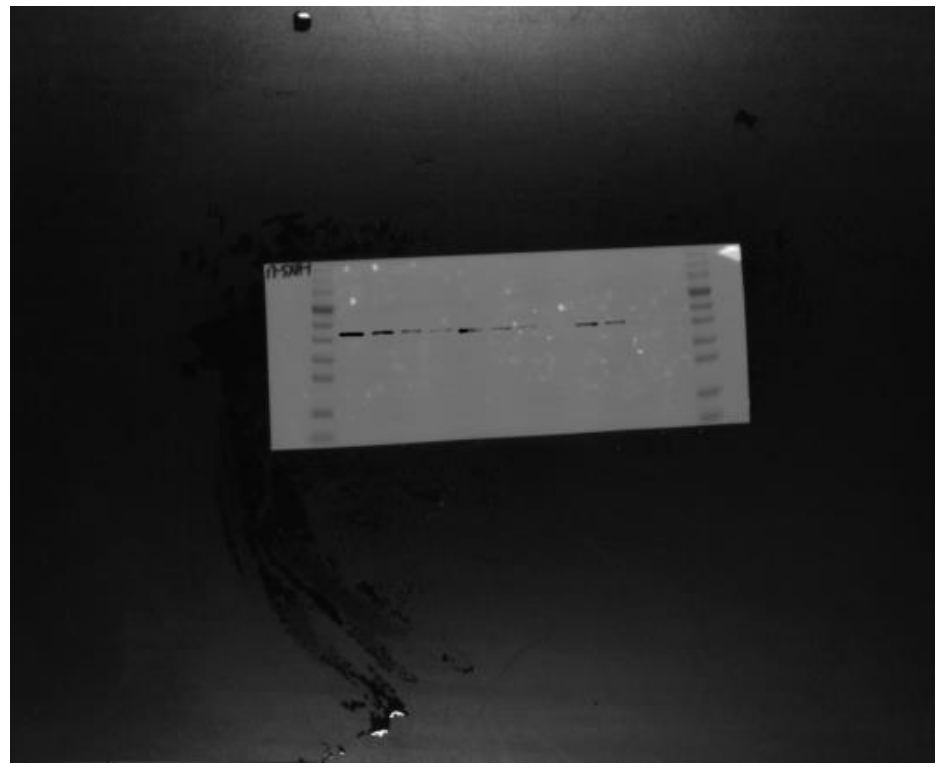

Figure S6A PANC-1: GAPDH (three repeats)

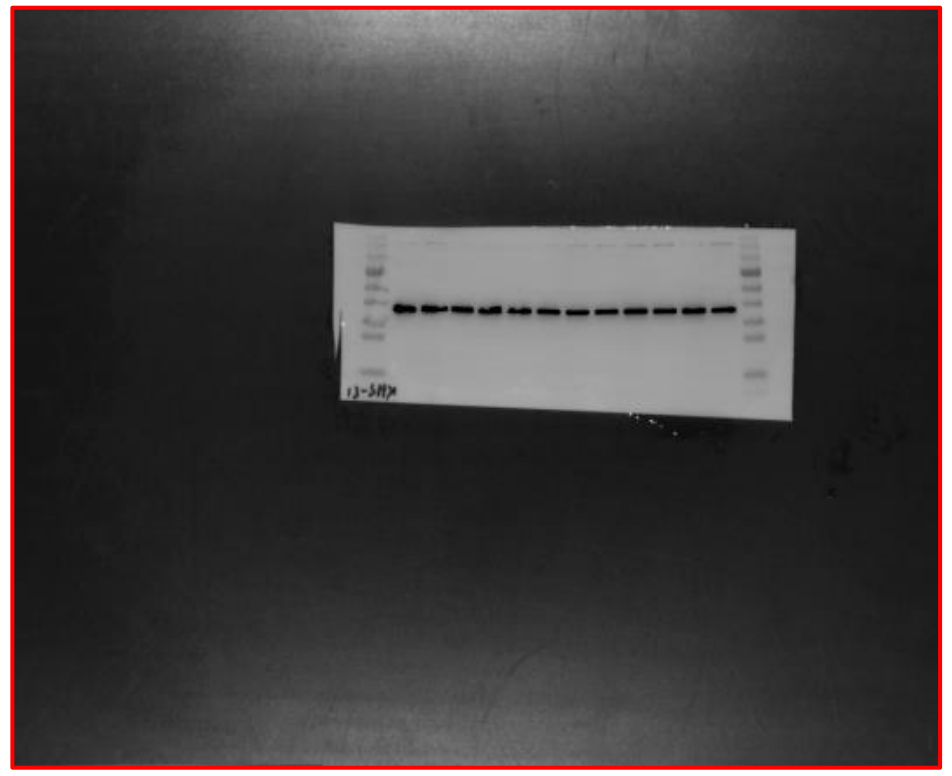

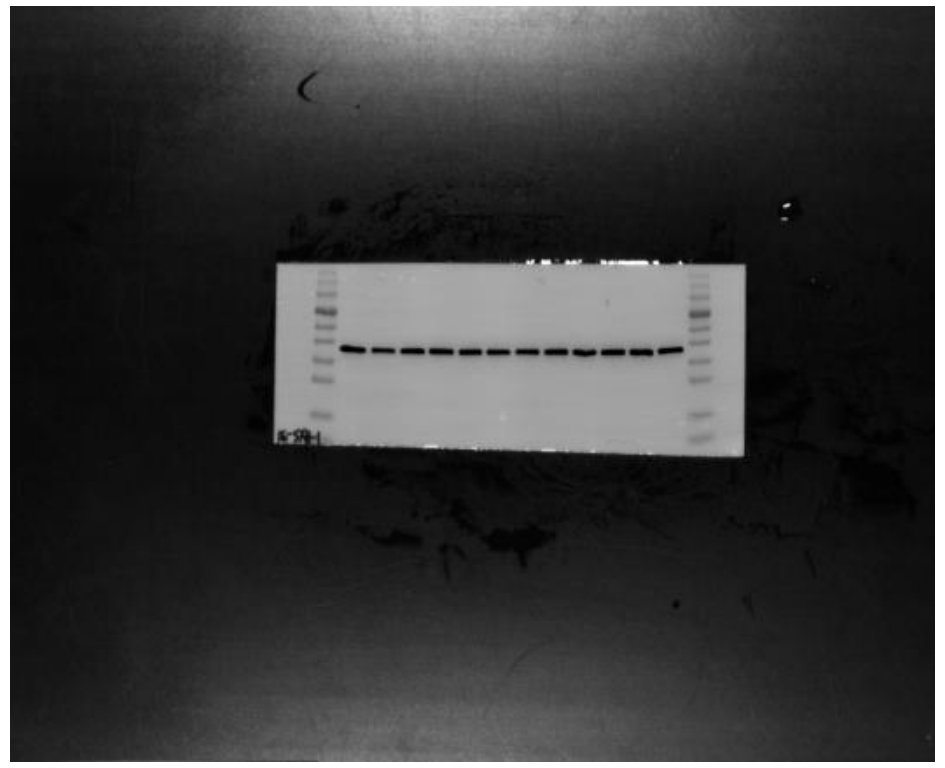

Figure S6A SW1990: SERPINE1 (three repeats)

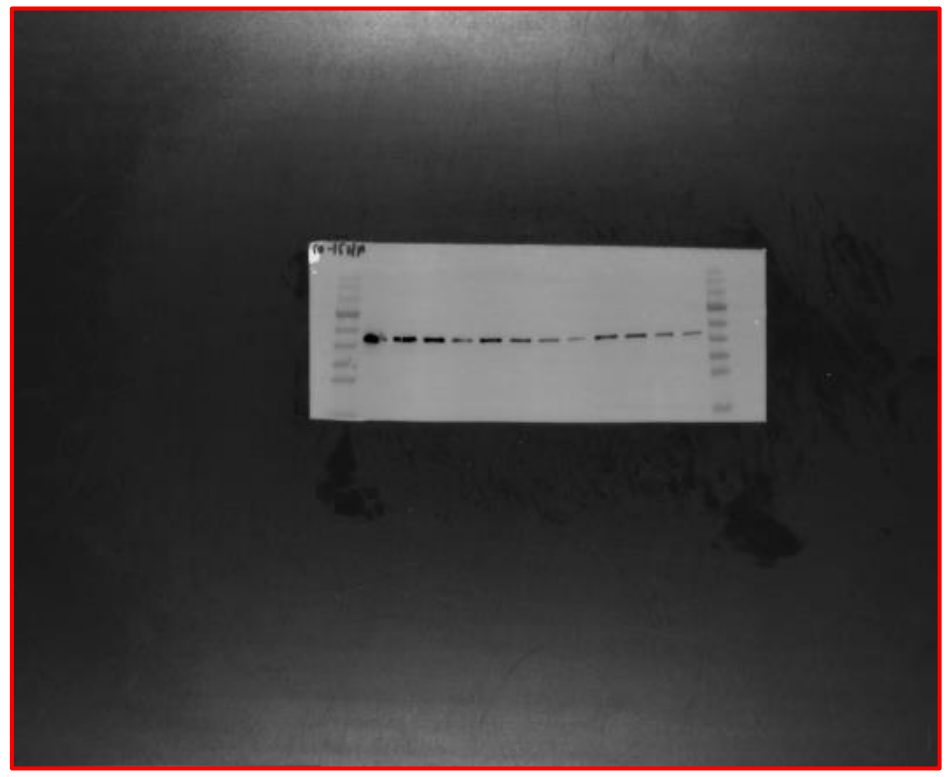

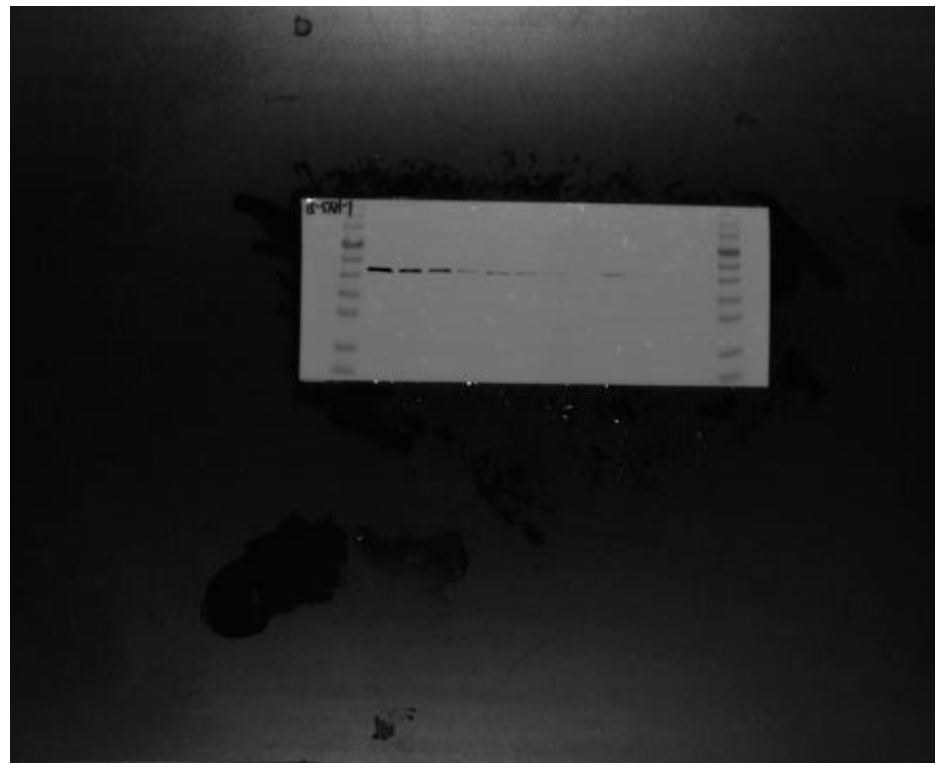

Figure S6A SW1990: GAPDH (three repeats)

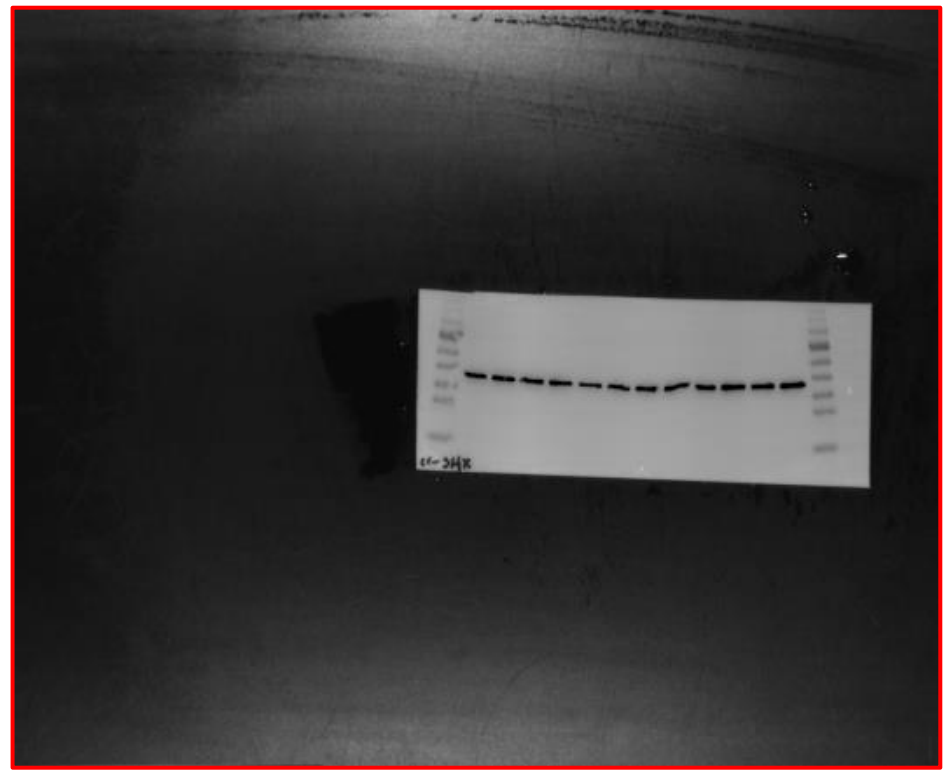

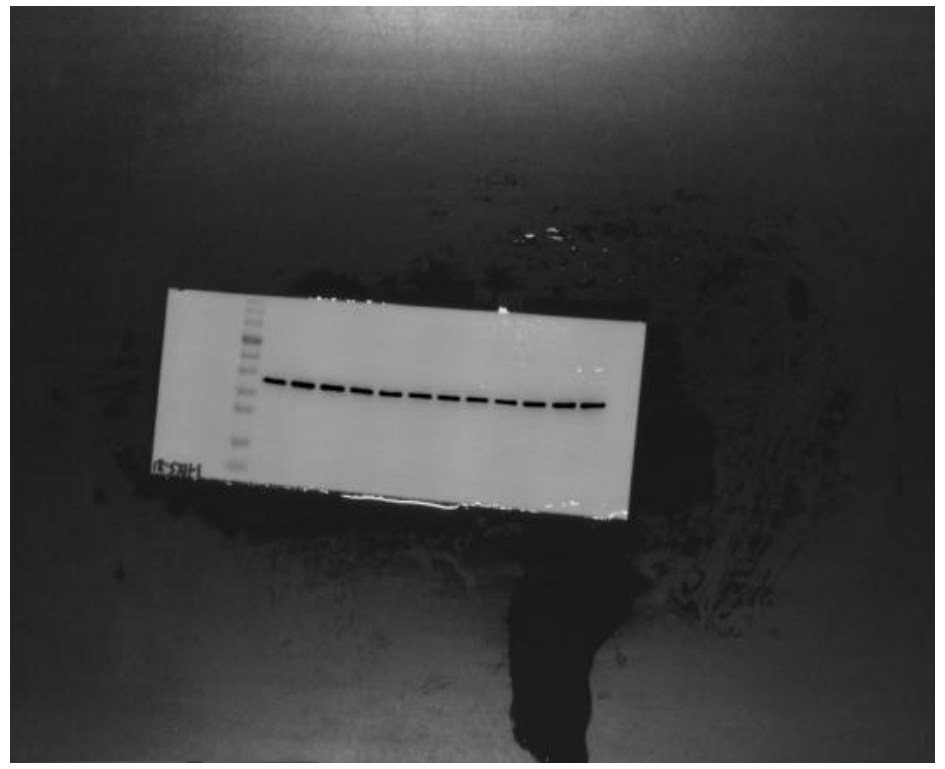

Figure S6C: SERPINE1 (three repeats)

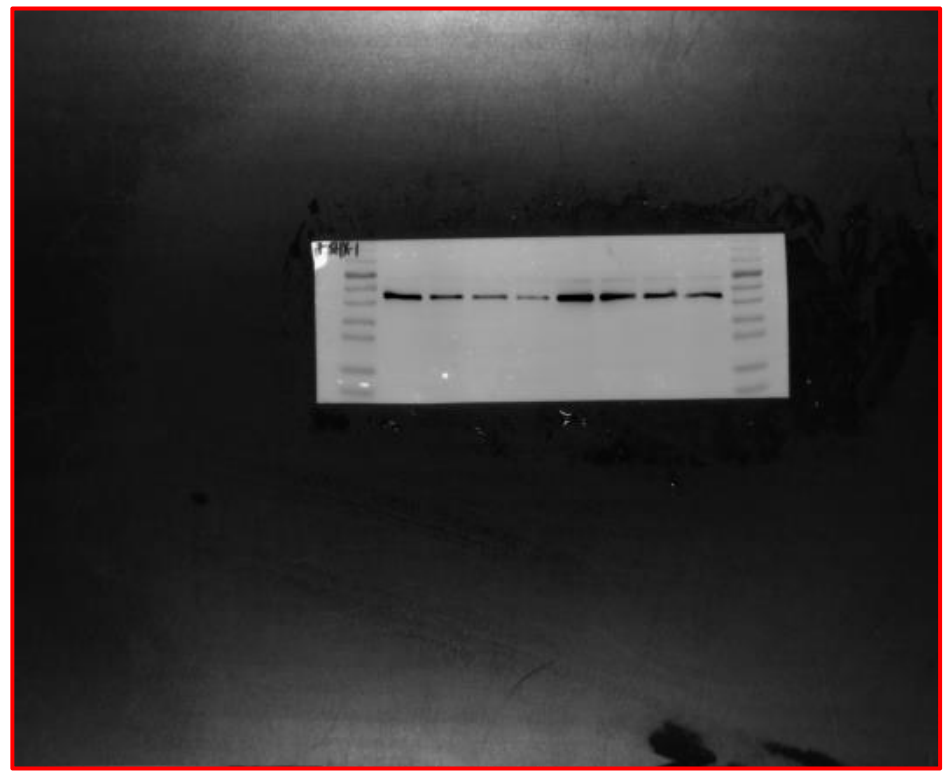

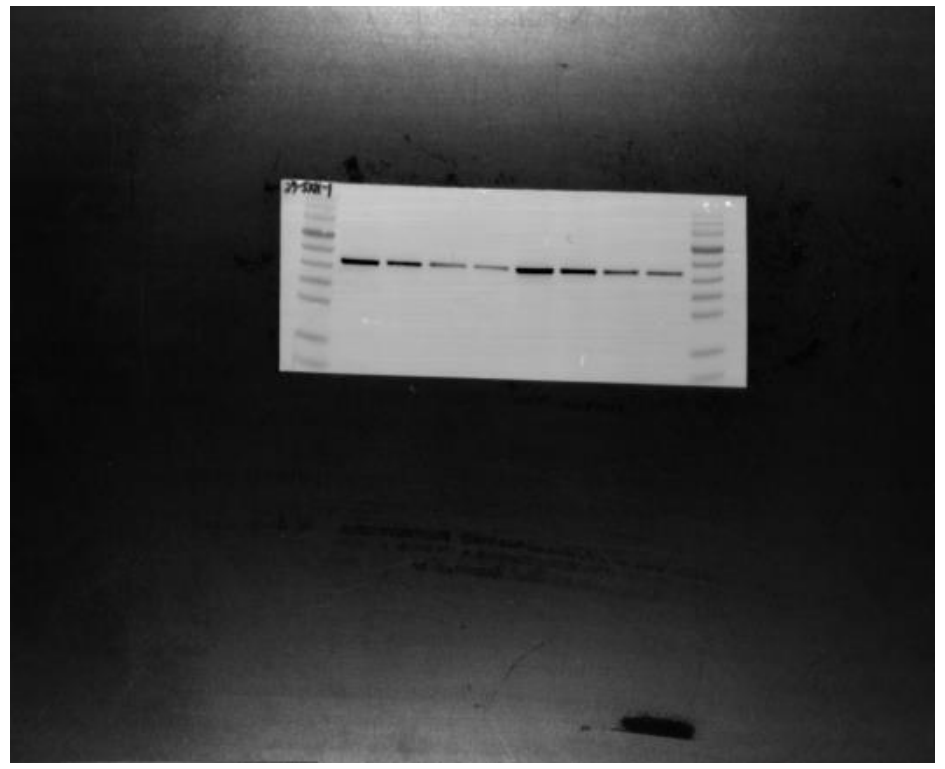

Figure S6C: GAPDH (three repeats)

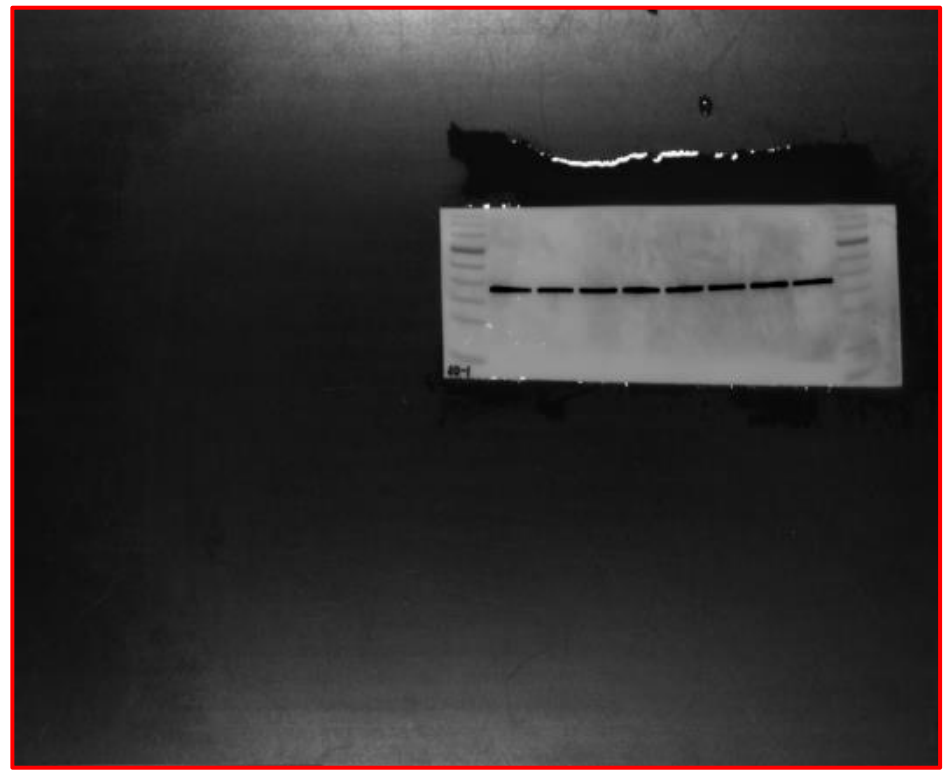

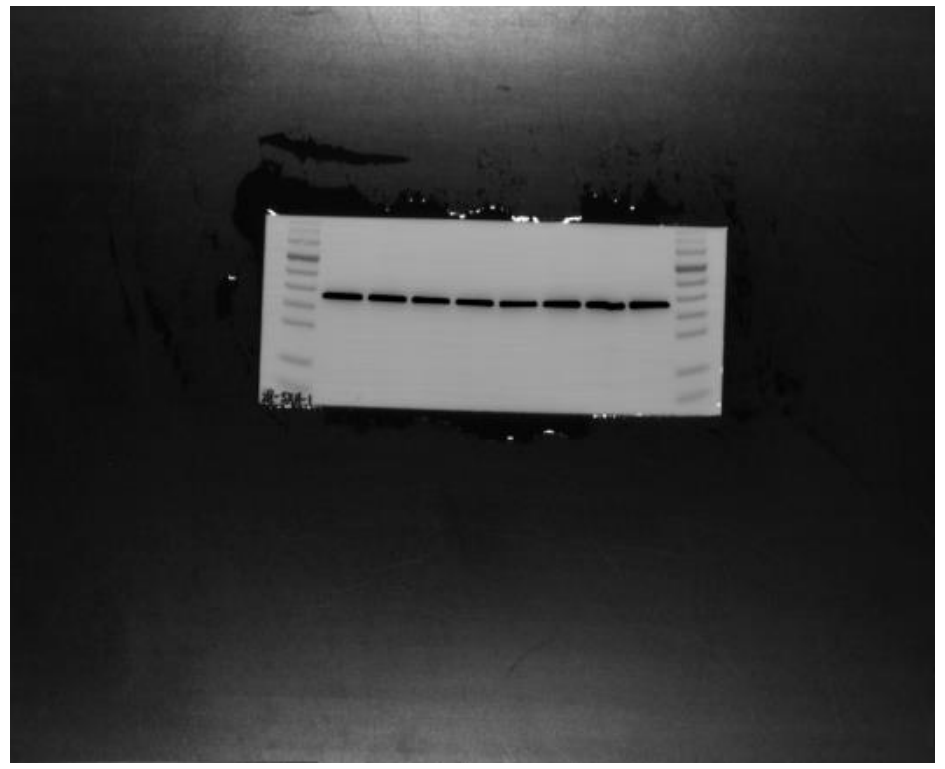

Supplement: Supplementary file 2 [file mmc2.pdf]
