# Supplementary material for: ARNTL2 promotes metastatic phenotypes through SERPINE1 transcription in KRAS G12D pancreatic cancer
Source: iScience. 2026 Sep 8;29(9):117479. doi: 10.1016/j.isci.2026.117479 (PMC13573702; doi:10.1016/j.isci.2026.117479)

Unedited microscopy images

- Unedited microscopy images from two independent experiments for each condition are provided.
- The images used in the published figures are indicated by red boxes.

Figure 2C: 0h shNC (three repeats)

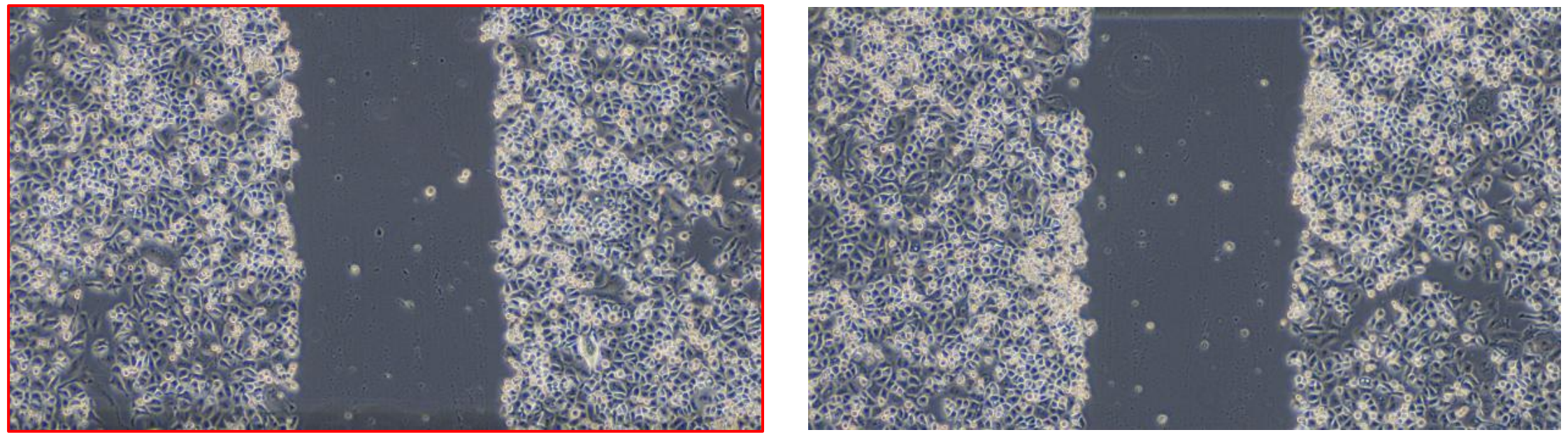

Figure 2C: 24h shNC (three repeats)

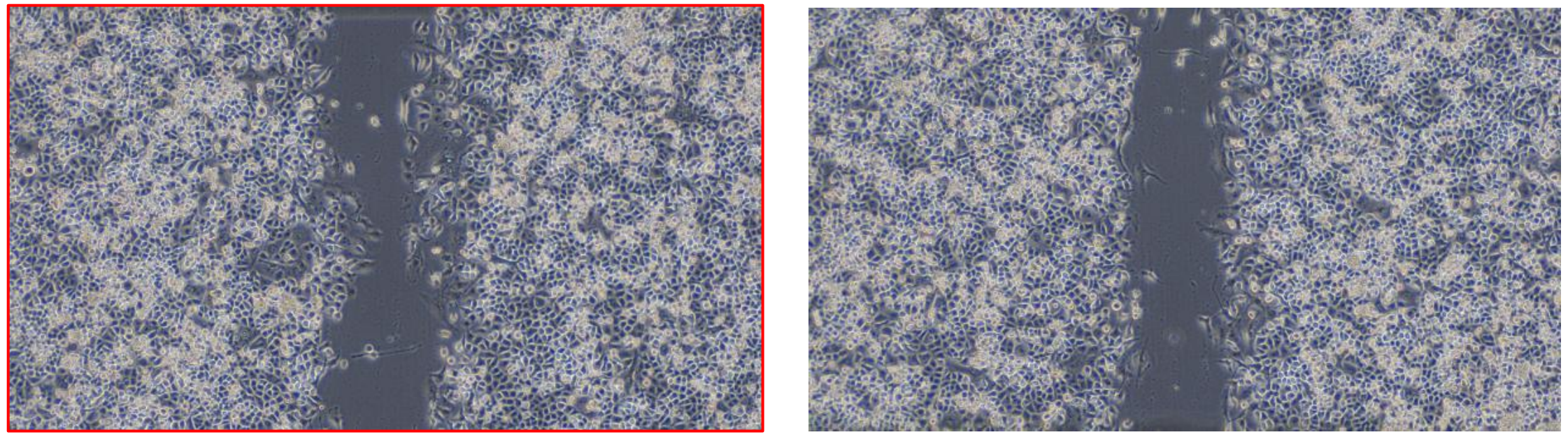

Figure 2C: 0h shARNTL2-1 (three repeats)

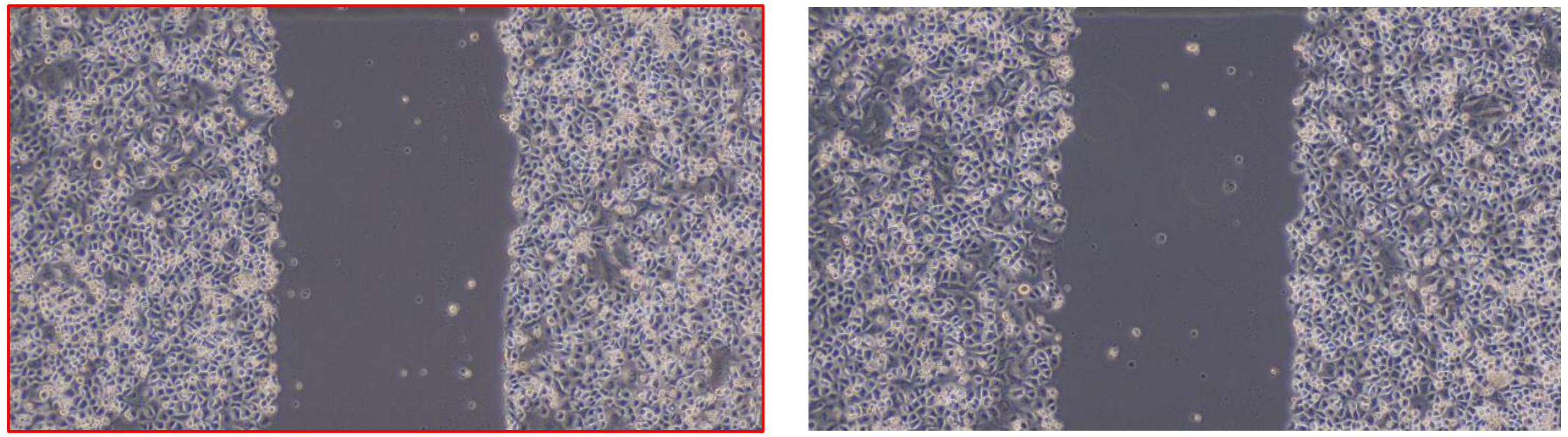

Figure 2C: 24h shARNTL2-1 (three repeats)

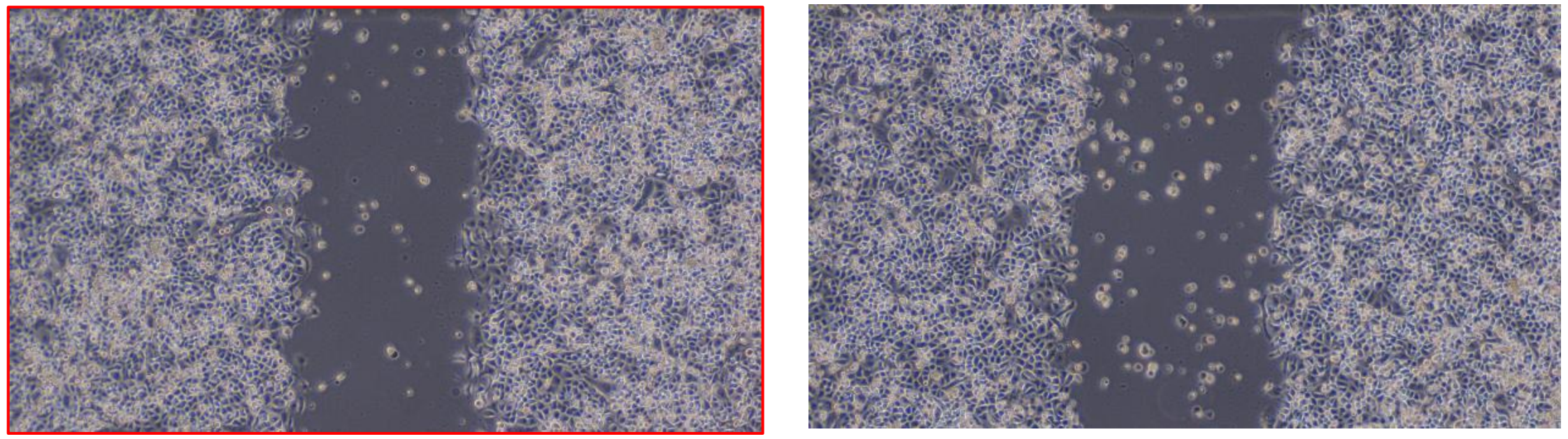

Figure 2C: 0h shARNTL2-2 (three repeats)

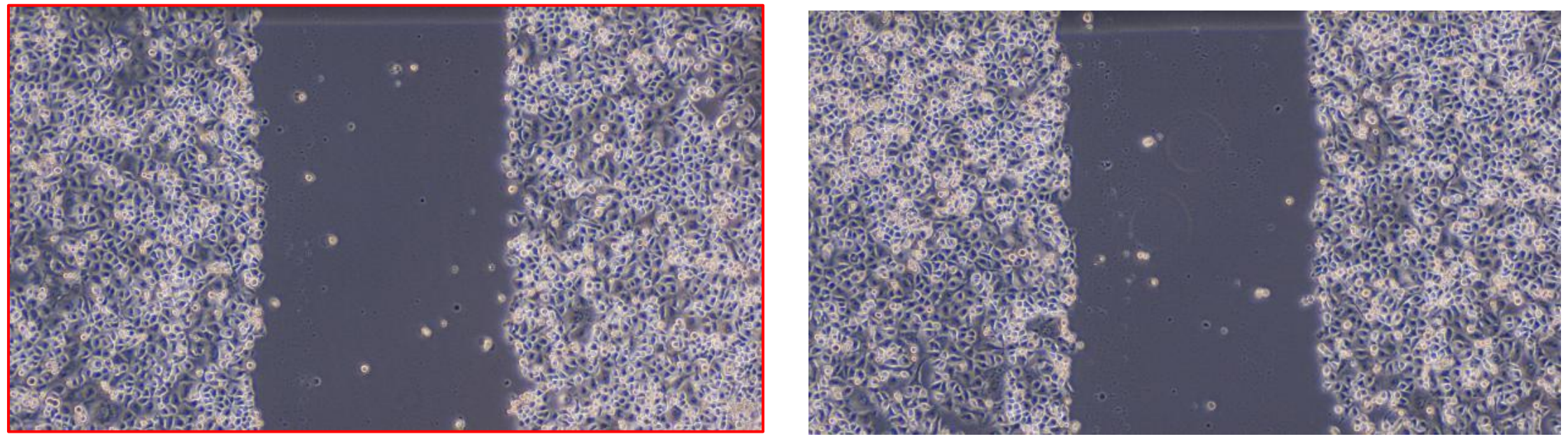

Figure 2C: 24h shARNTL2-2 (three repeats)

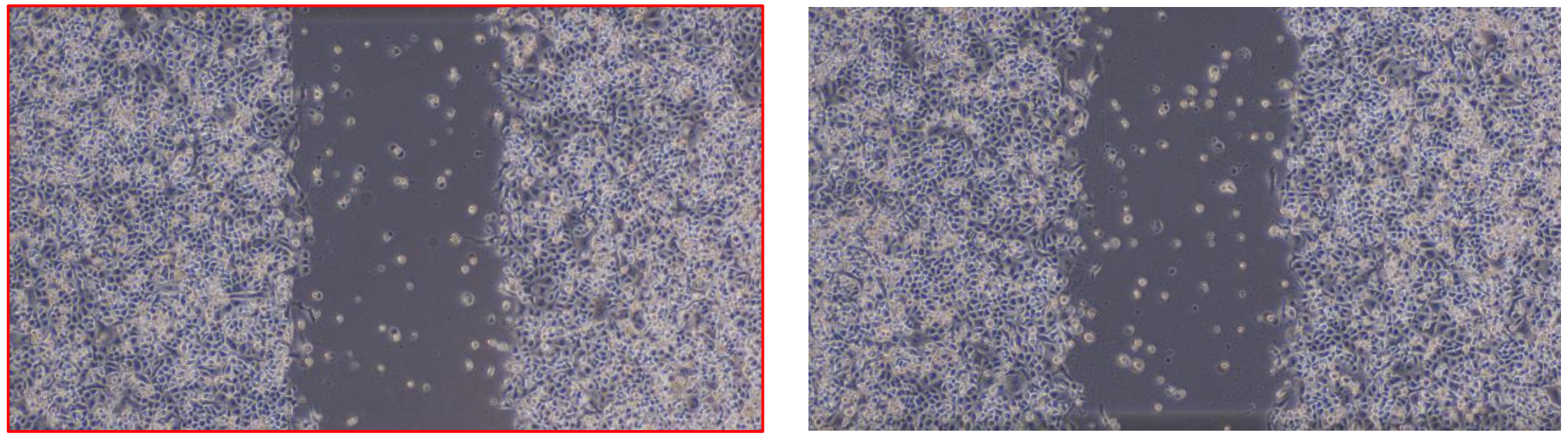

Figure 2D: 0h Vector (three repeats)

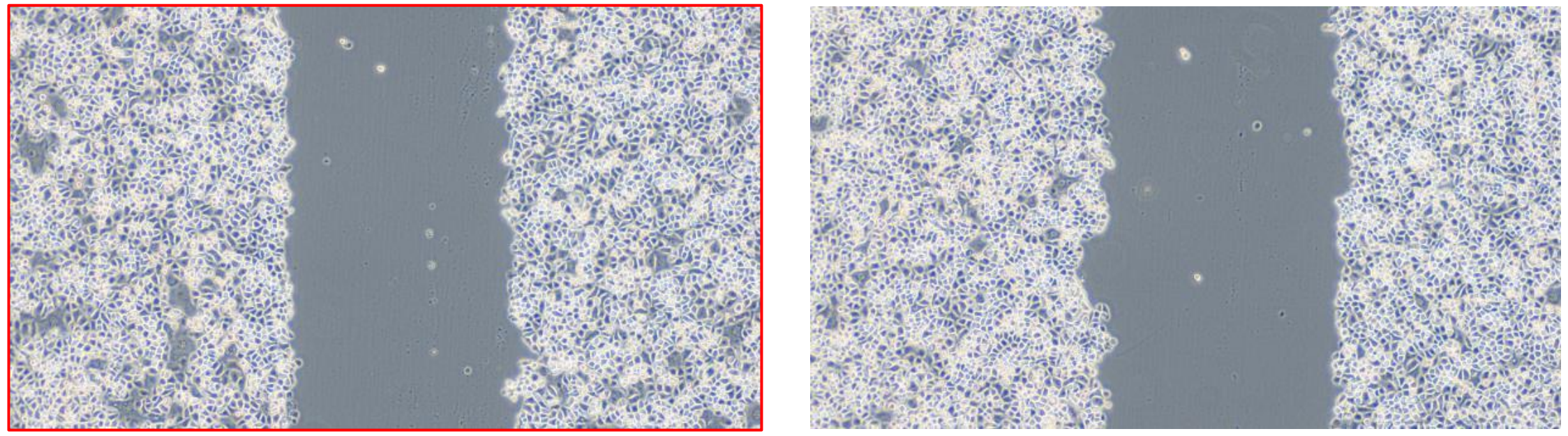

Figure 2D: 24h Vector (three repeats)

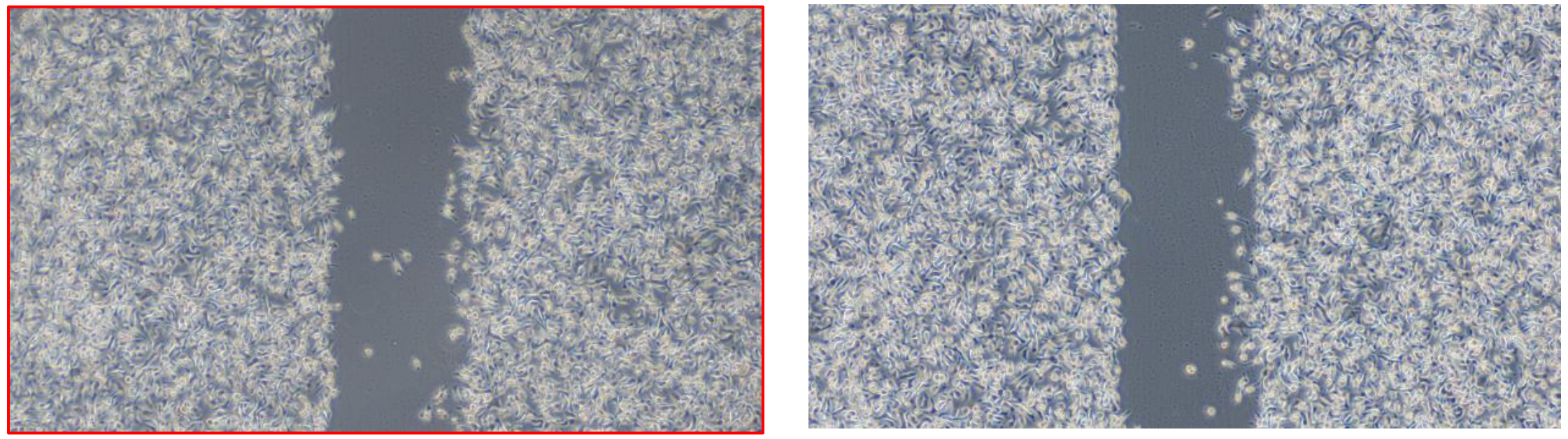

Figure 2D: 0h ARNTL2 (three repeats)

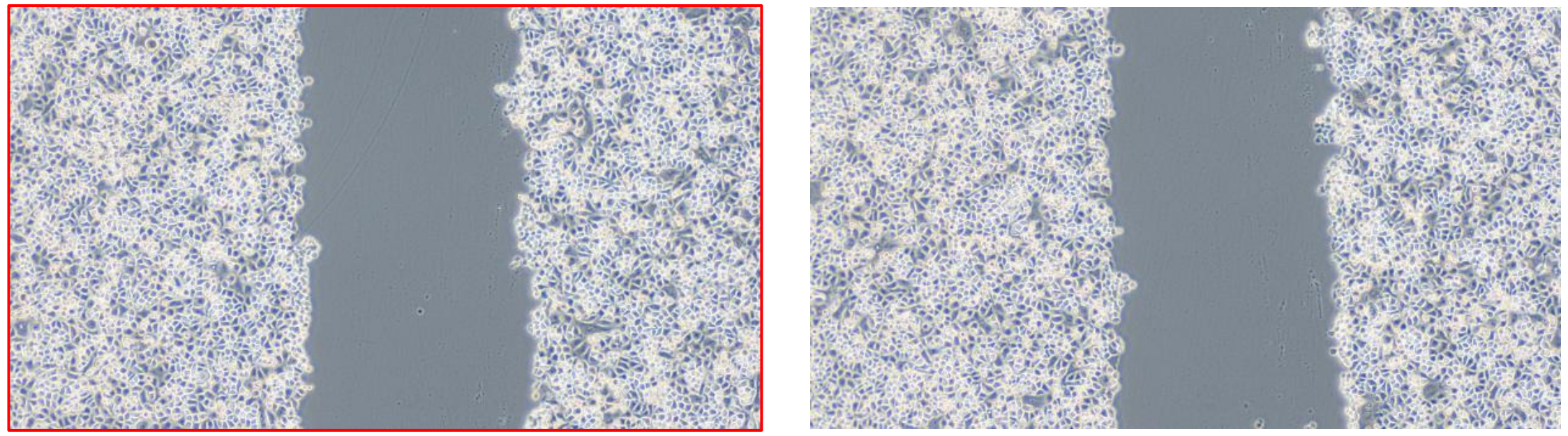

Figure 2D: 24h ARNTL2 (three repeats)

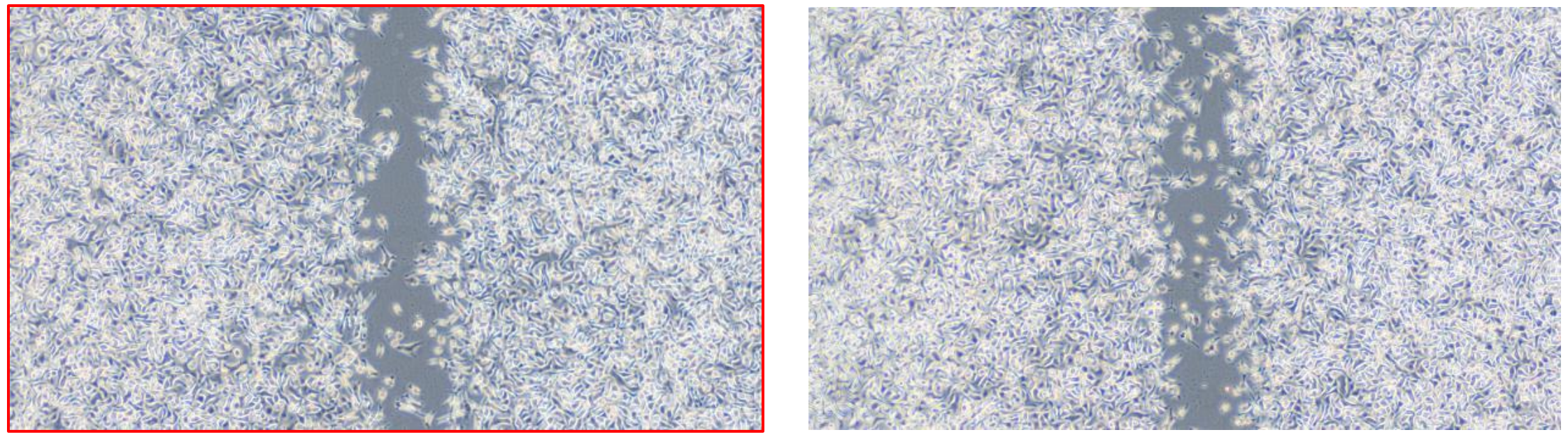

Figure 2E: shNC (three repeats)

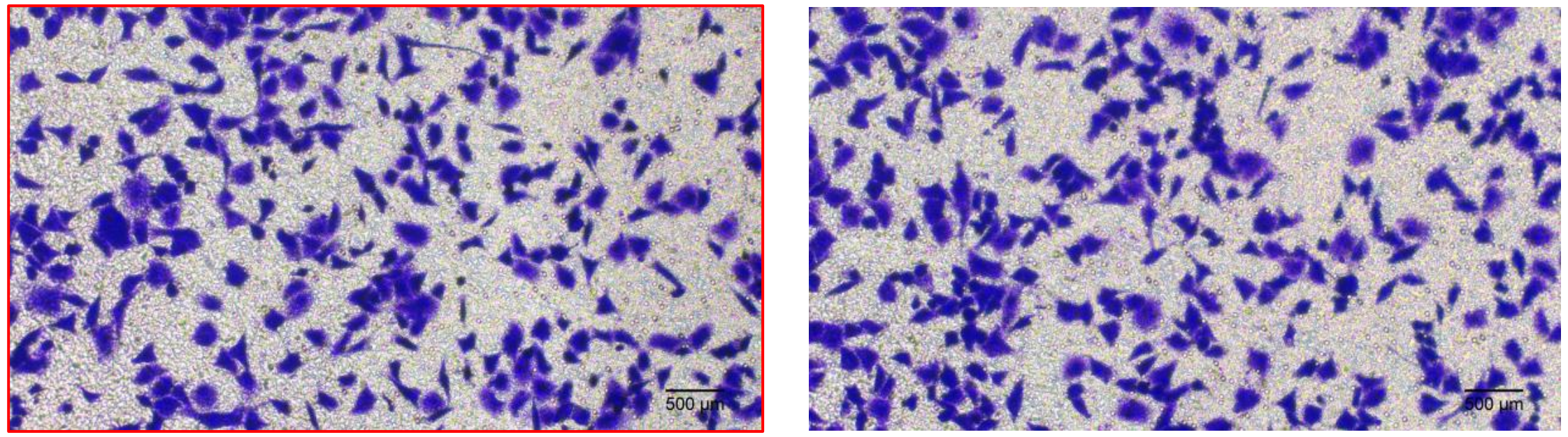

Figure 2E: shARNTL2-1 (three repeats)

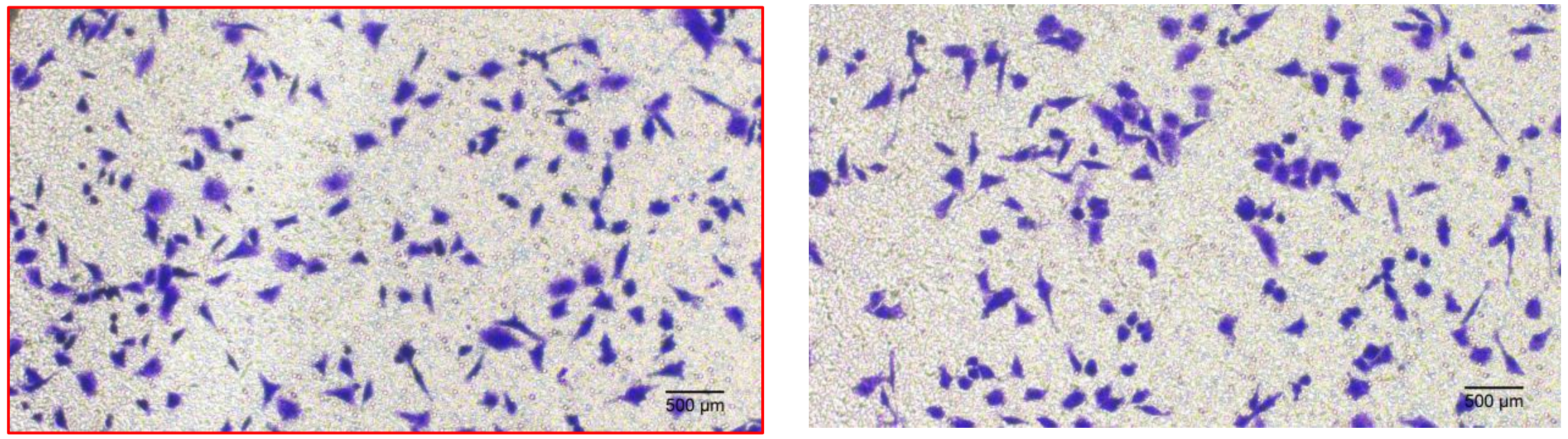

Figure 2E: shARNTL2-2 (three repeats)

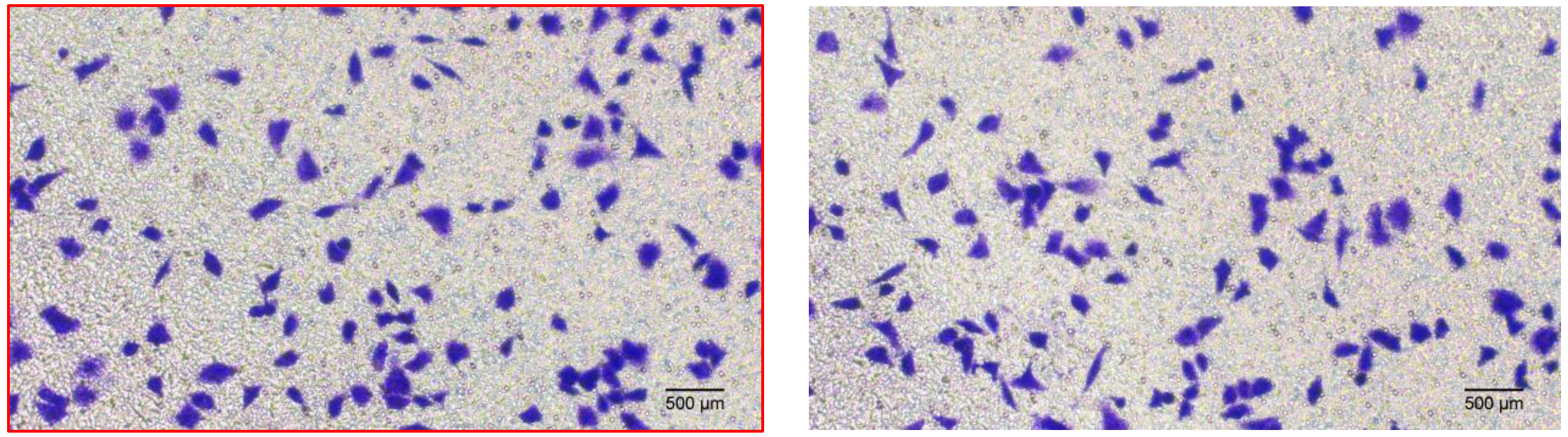

Figure 2F: Vector (three repeats)

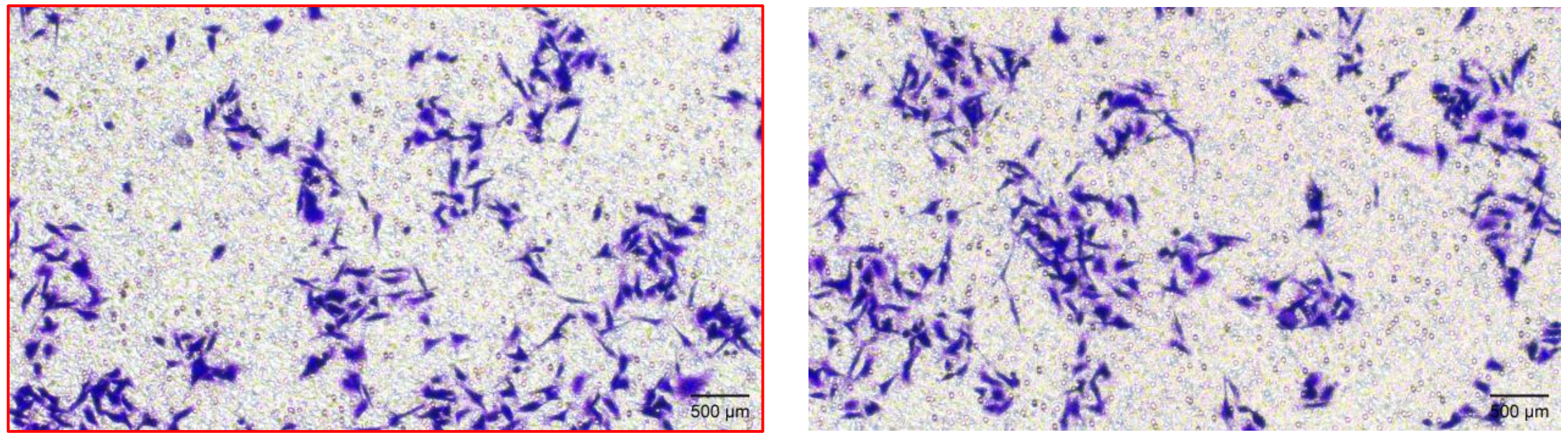

Figure 2F: ARNTL2 (three repeats)

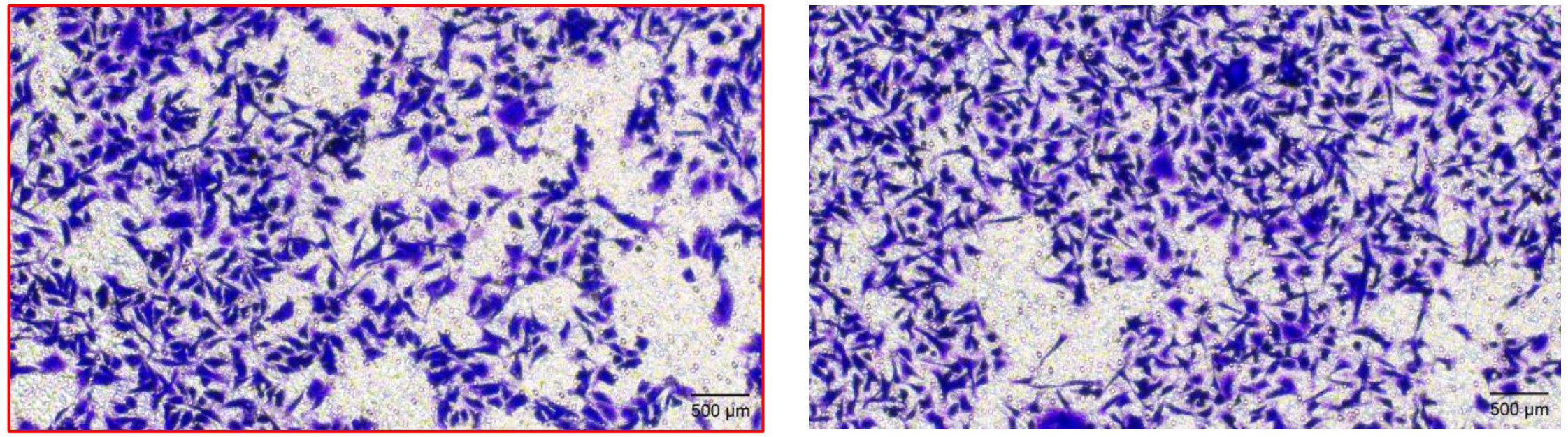

Figure 2G: shNC (three repeats)

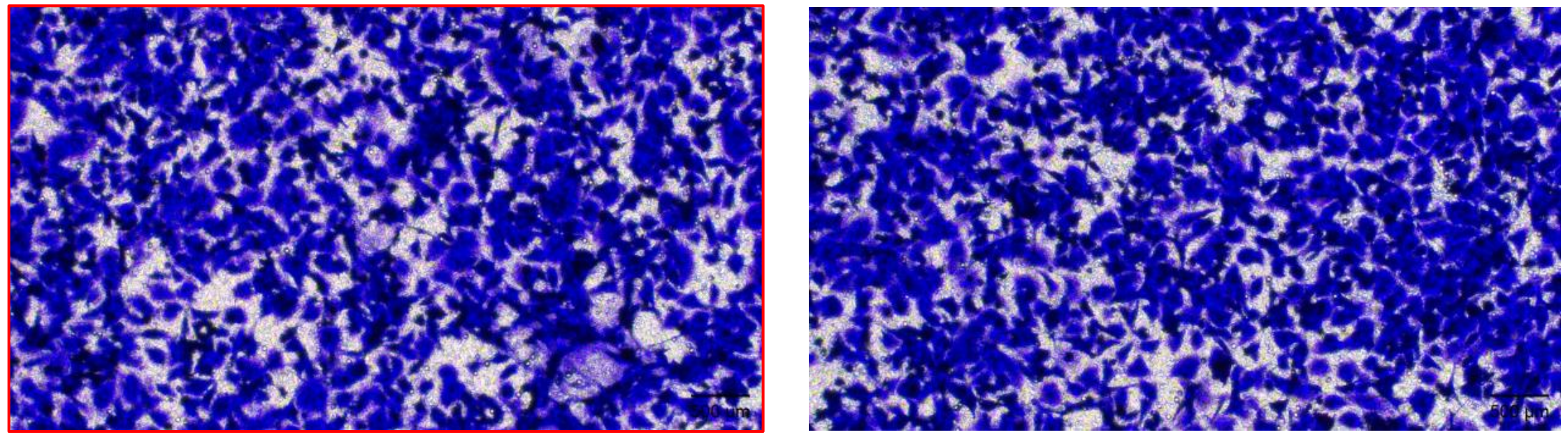

Figure 2G: shARNTL2-1 (three repeats)

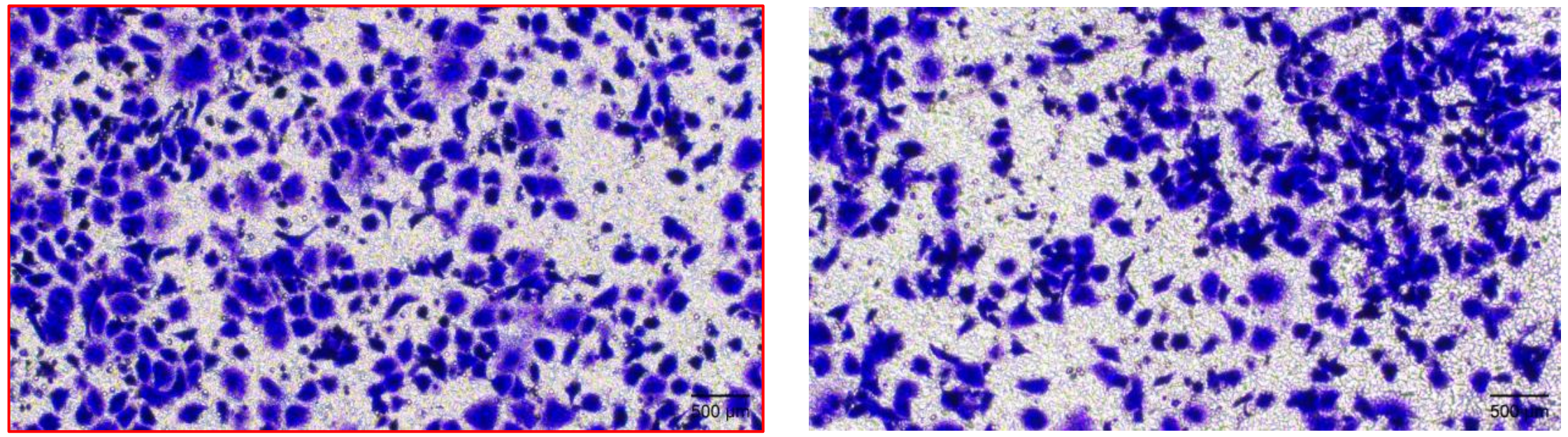

Figure 2G: shARNTL2-2 (three repeats)

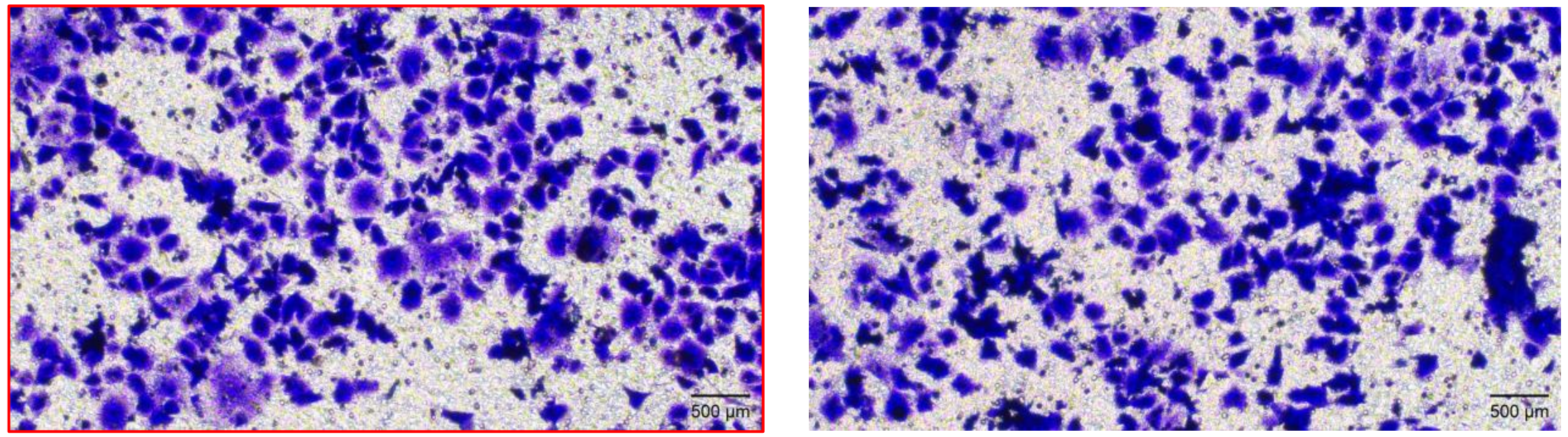

Figure 2H: Vector (three repeats)

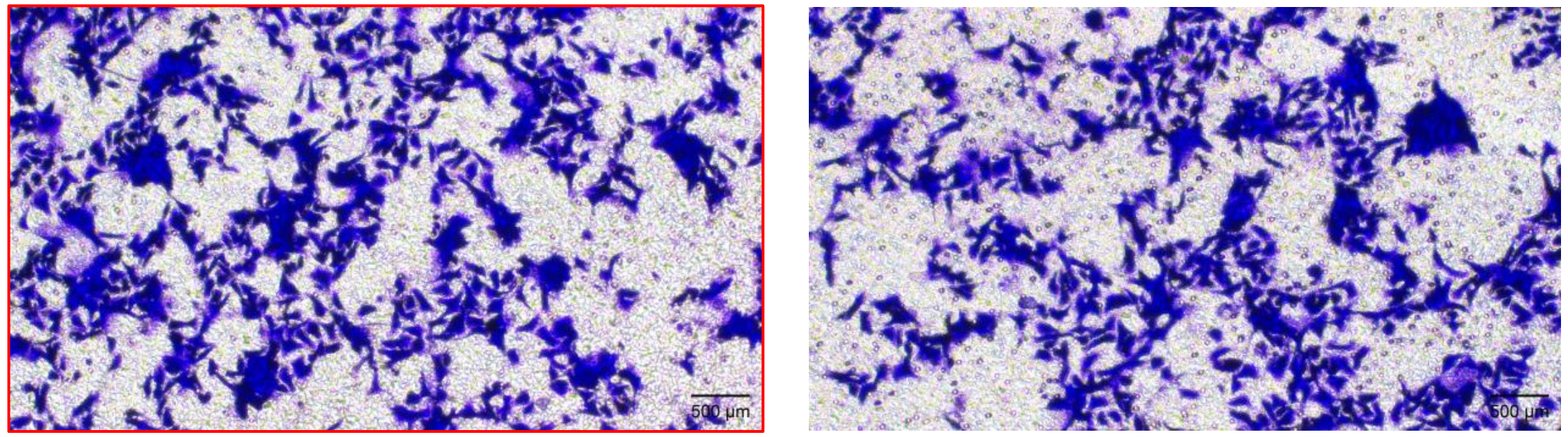

Figure 2H: ARNTL2 (three repeats)

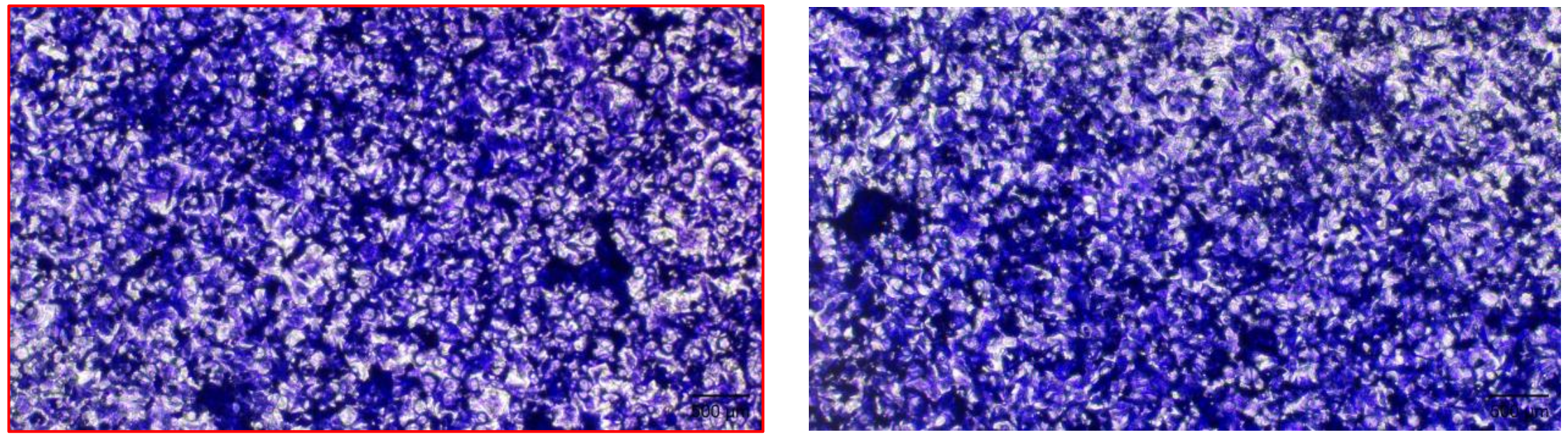

Figure 4D: 0h shNC (three repeats)

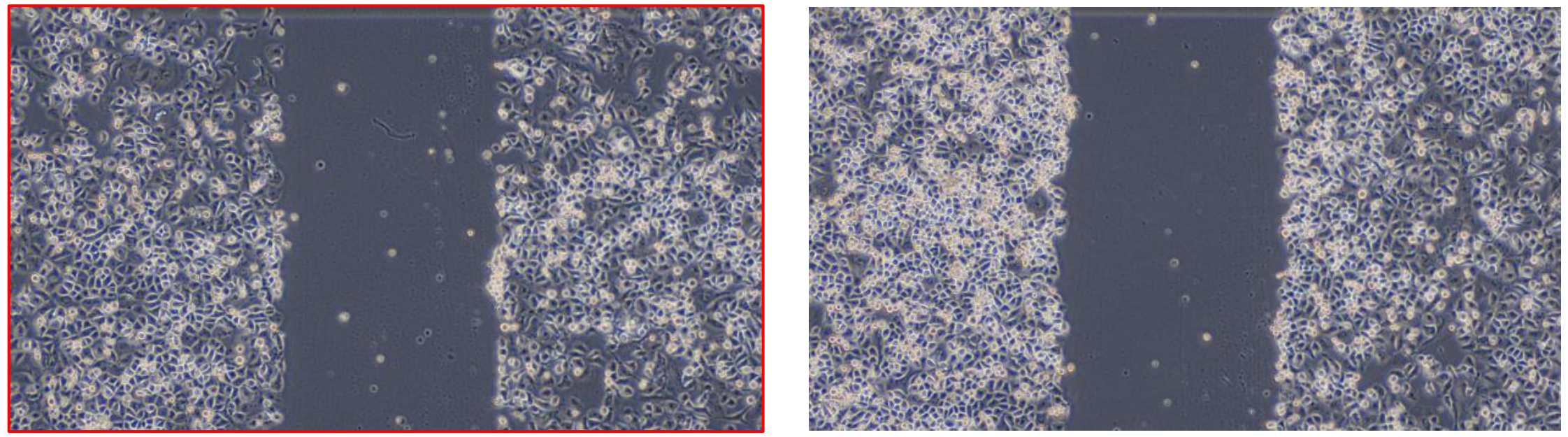

Figure 4D: 24h shNC (three repeats)

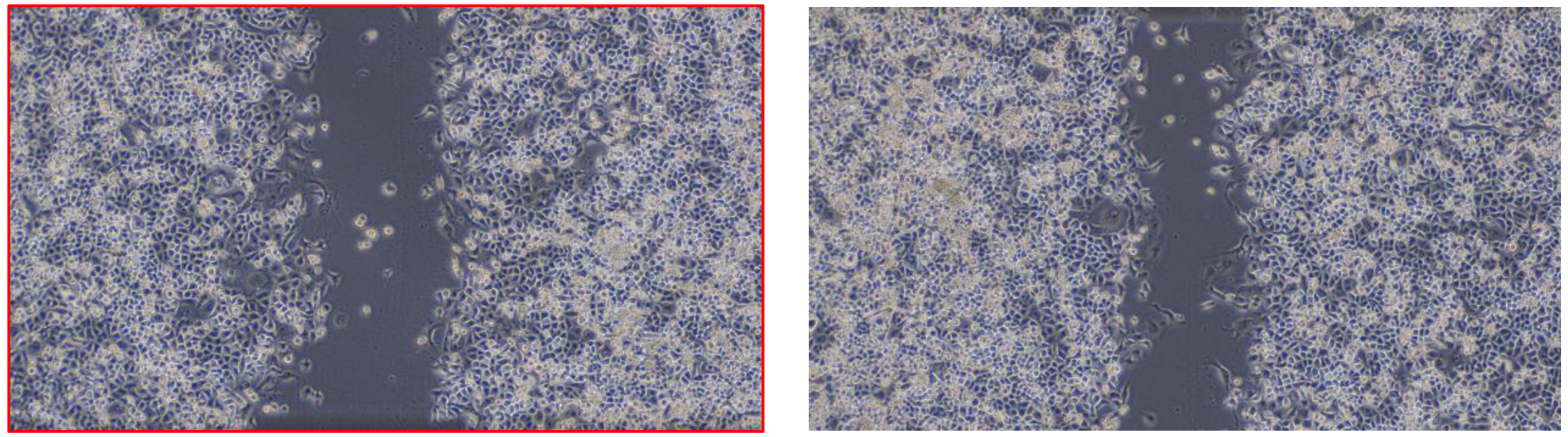

Figure 4D: 0h shSERPINE1-1 (three repeats)

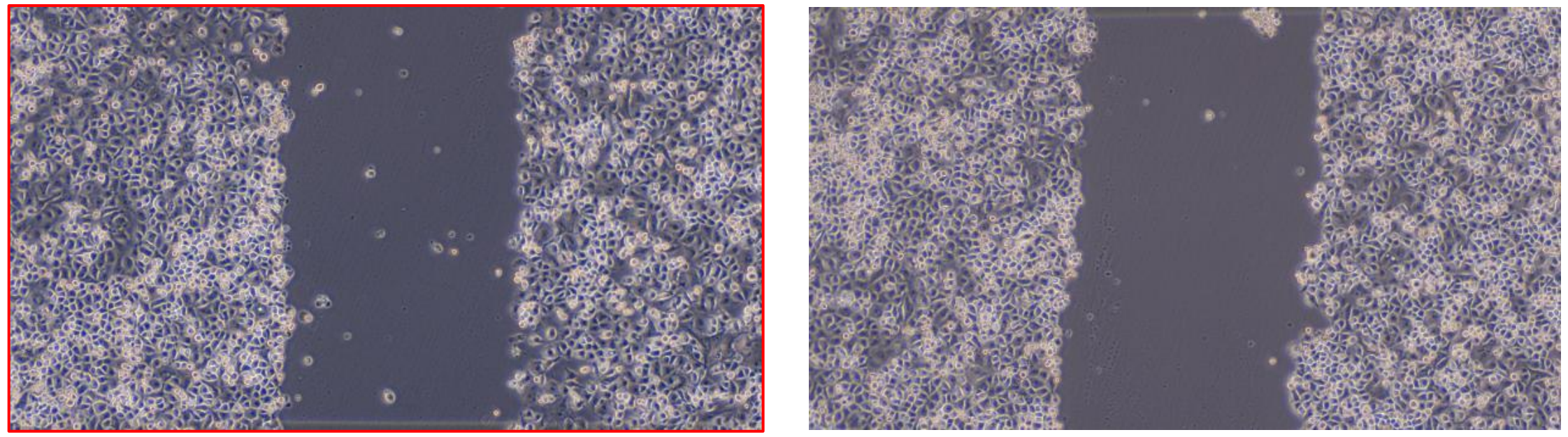

Figure 4D: 24h shSERPINE1-1 (three repeats)

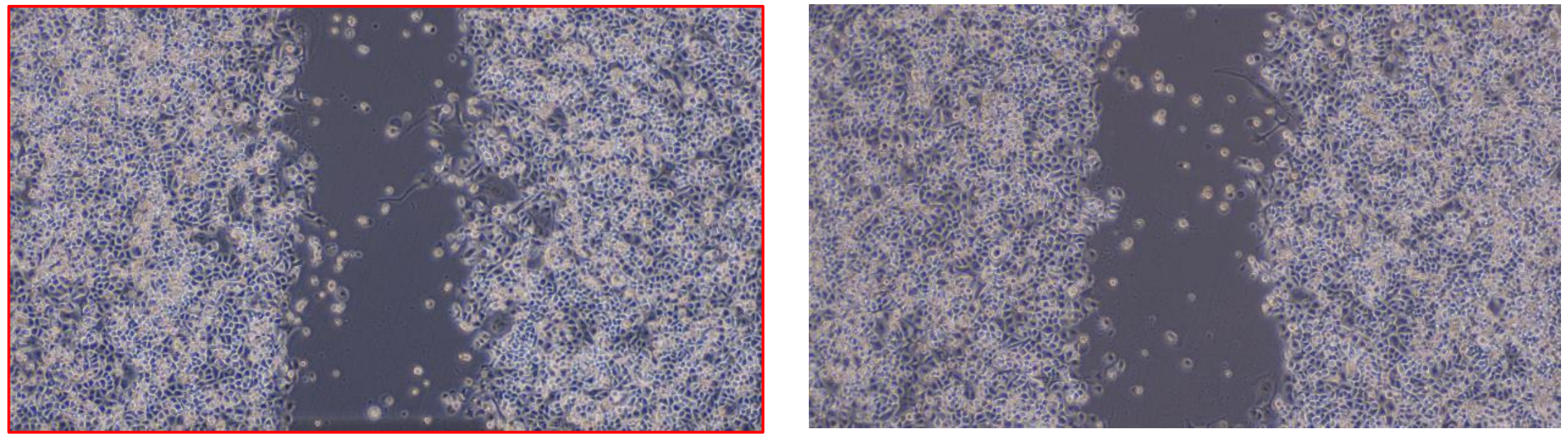

Figure 4D: 0h shSERPINE1-2 (three repeats)

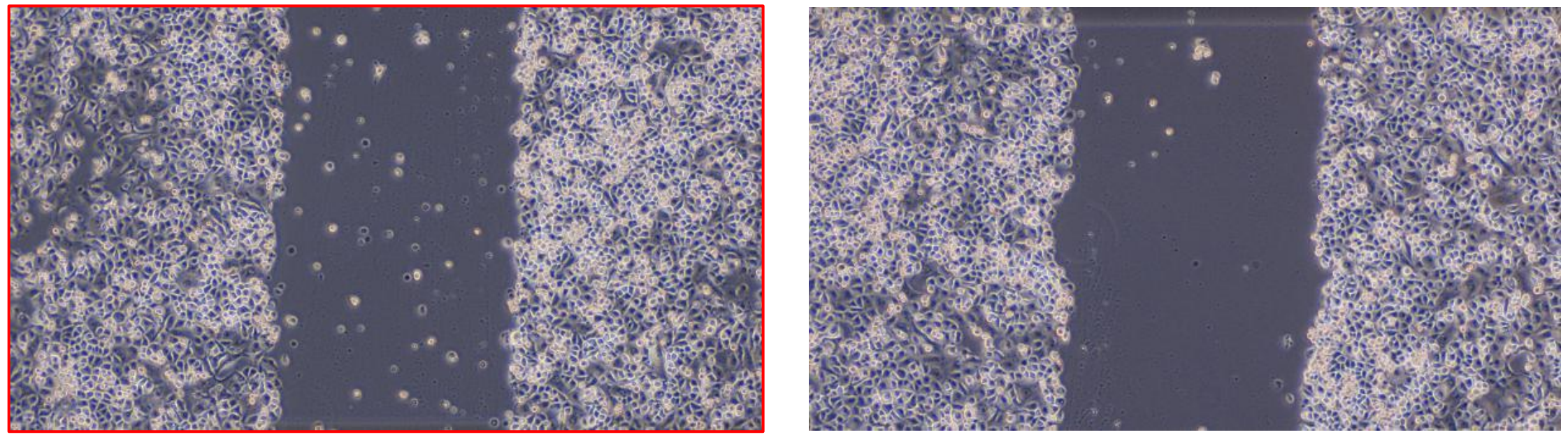

Figure 4D: 24h shSERPINE1-2 (three repeats)

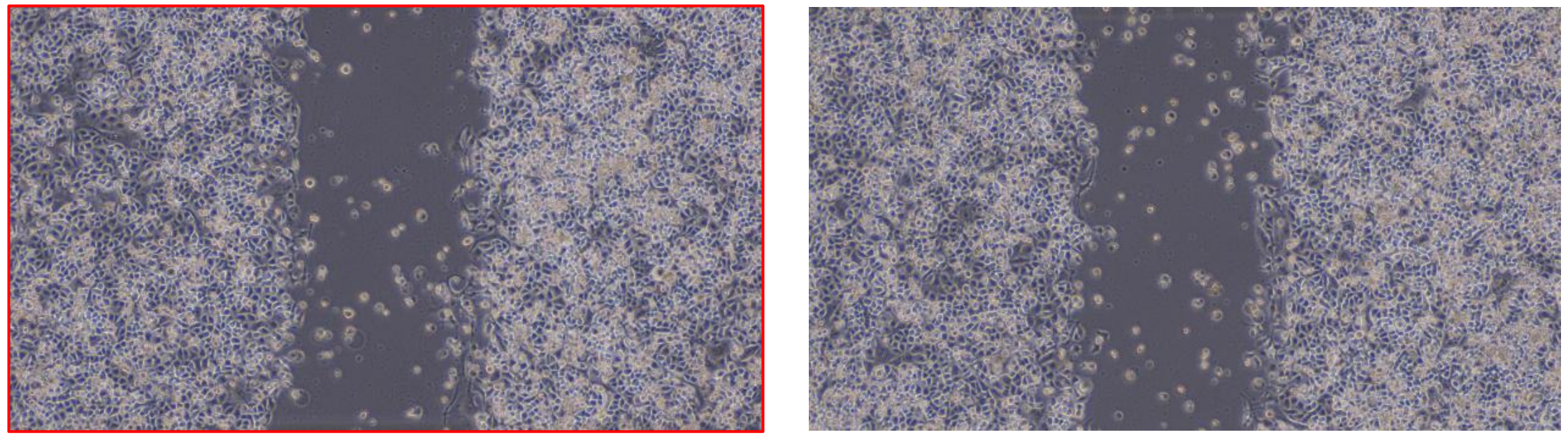

Figure 4E: 0h Vector (three repeats)

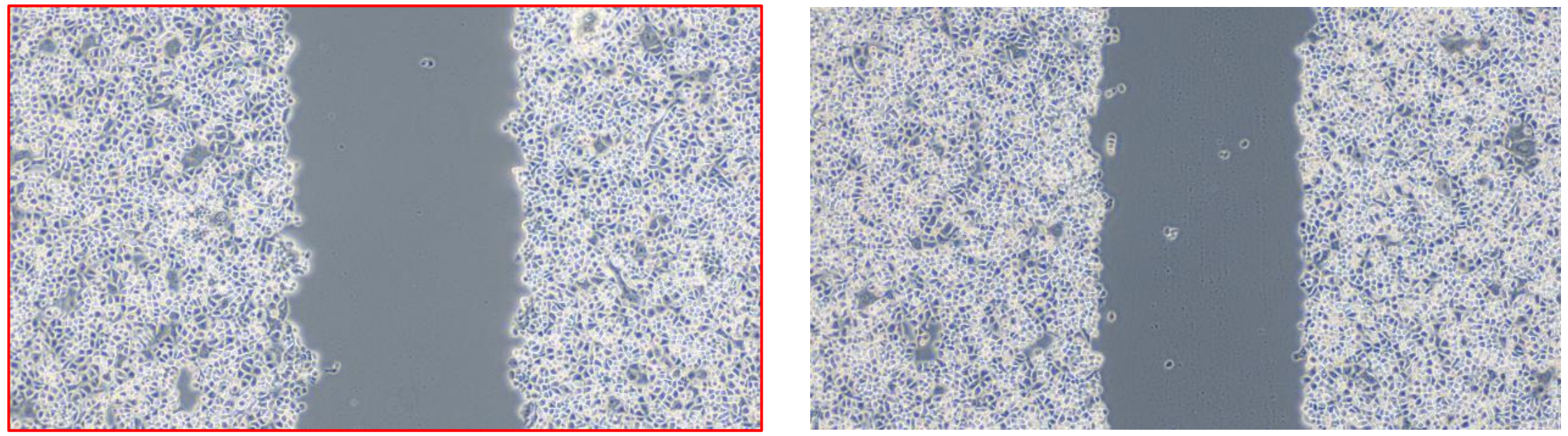

Figure 4E: 24h Vector (three repeats)

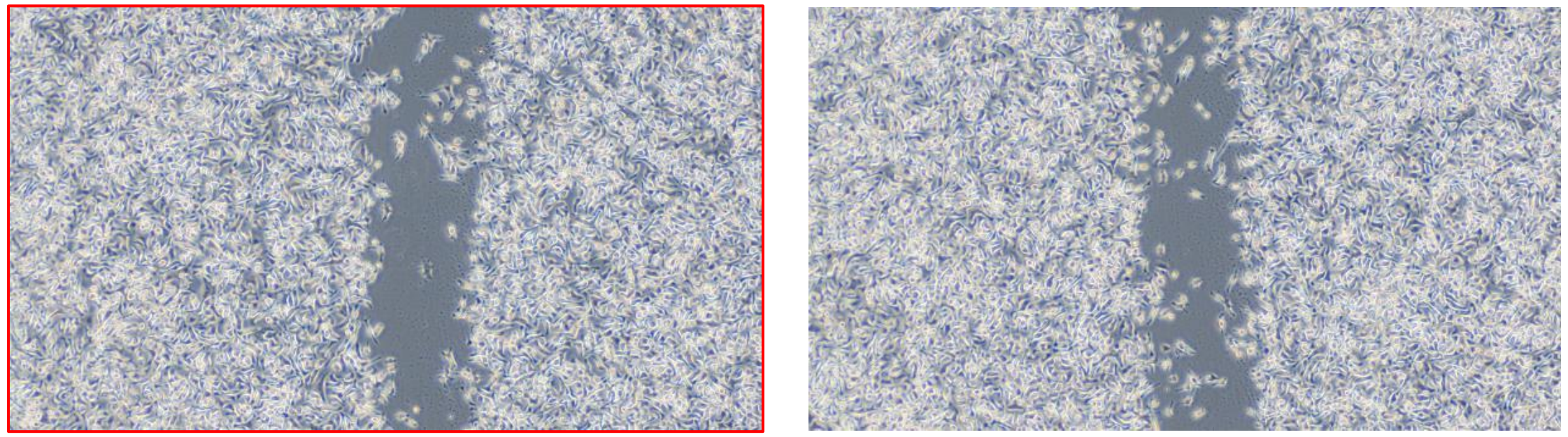

Figure 4E: 0h SERPINE1 (three repeats)

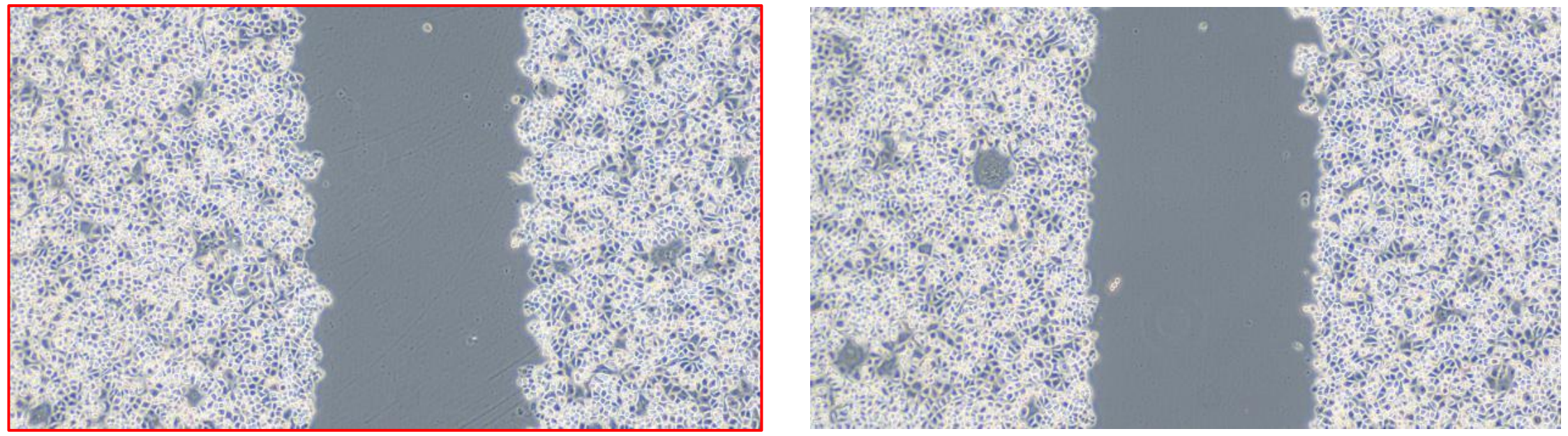

Figure 4E: 24h SERPINE1 (three repeats)

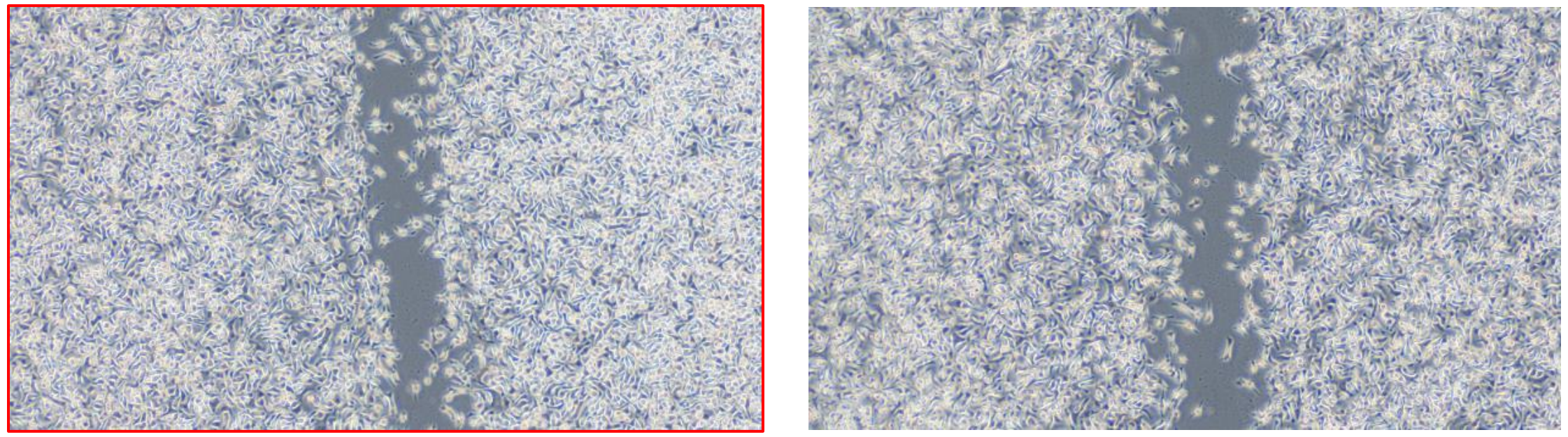

Figure 4F: shNC (three repeats)

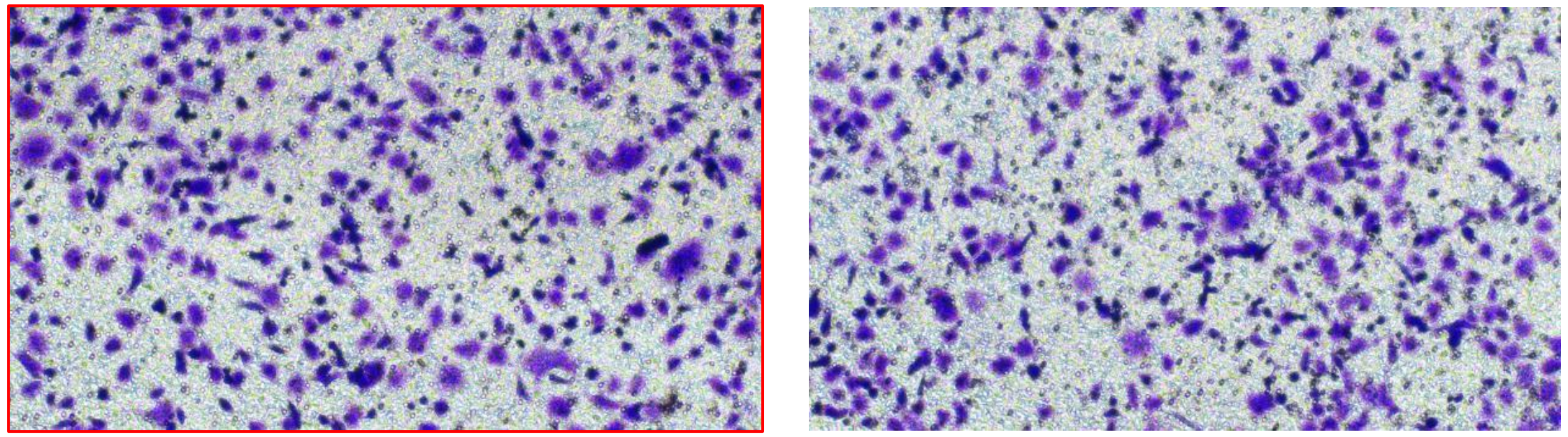

Figure 4F: shSERPINE1-1 (three repeats)

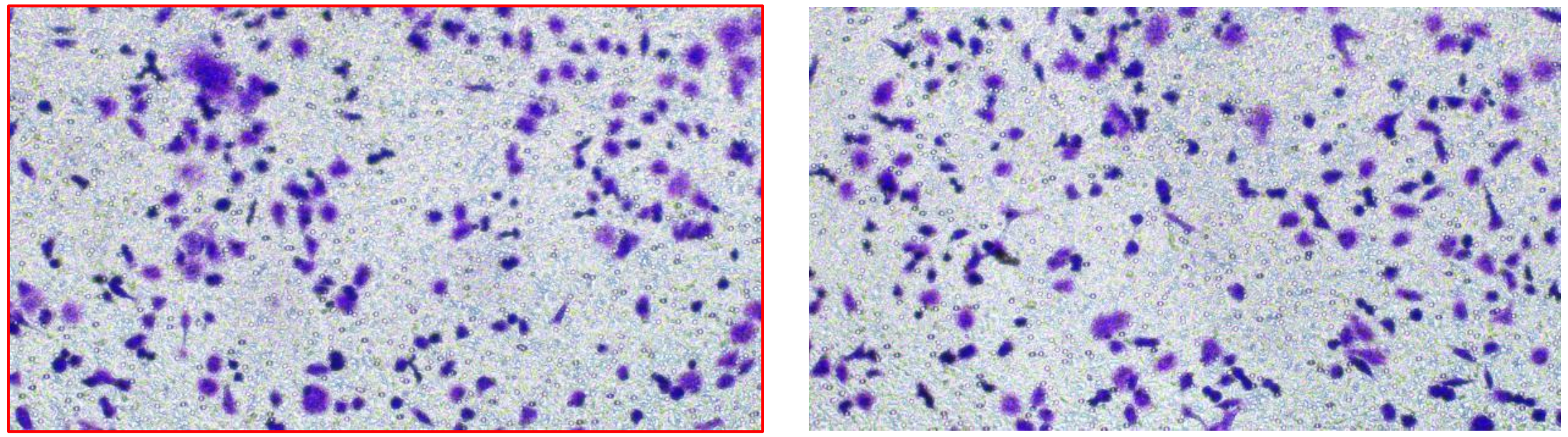

Figure 4F: shSERPINE1-2 (three repeats)

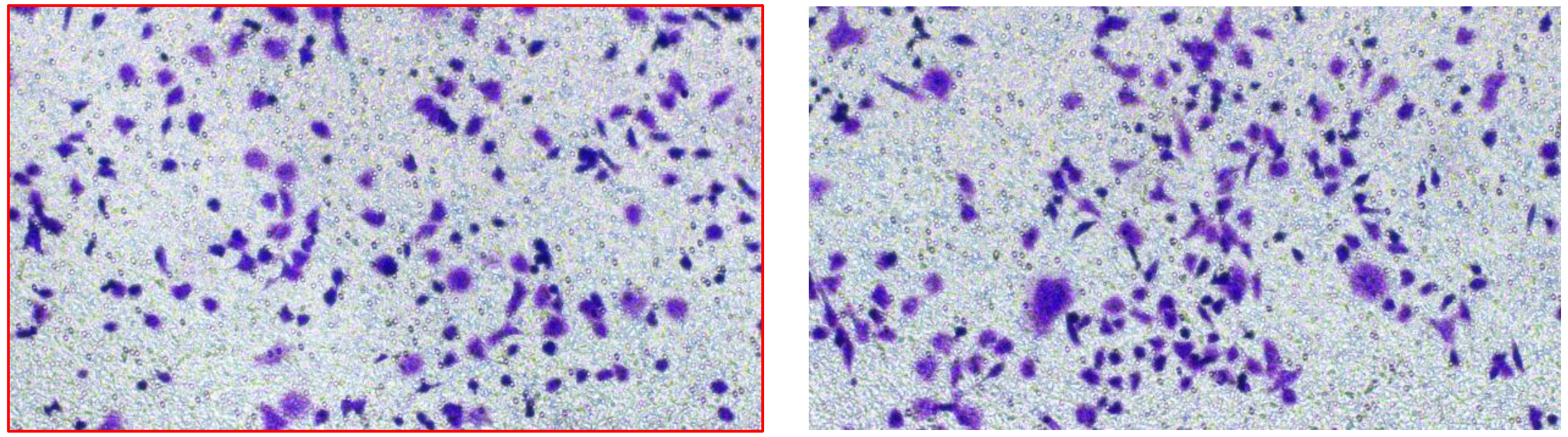

Figure 4G: Vector (three repeats)

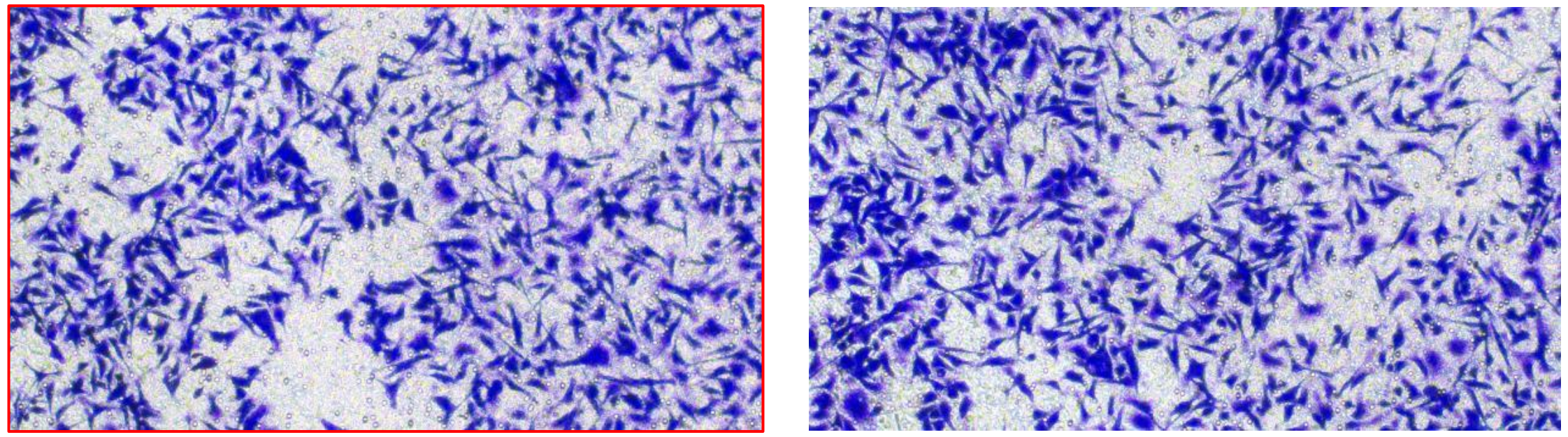

Figure 4G: SERPINE1 (three repeats)

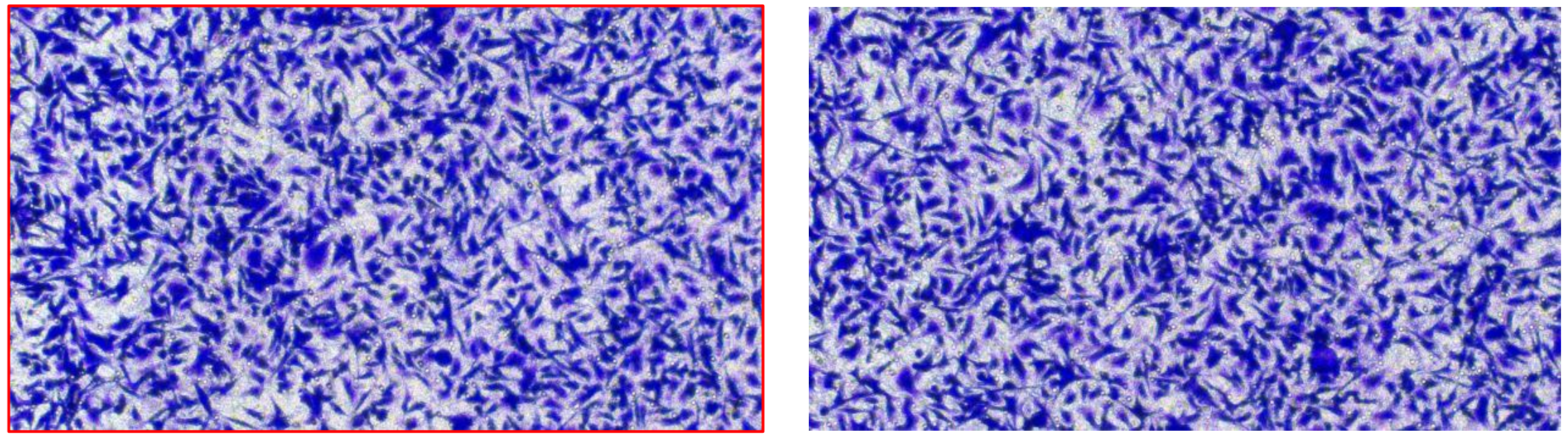

Figure 4H: shNC (three repeats)

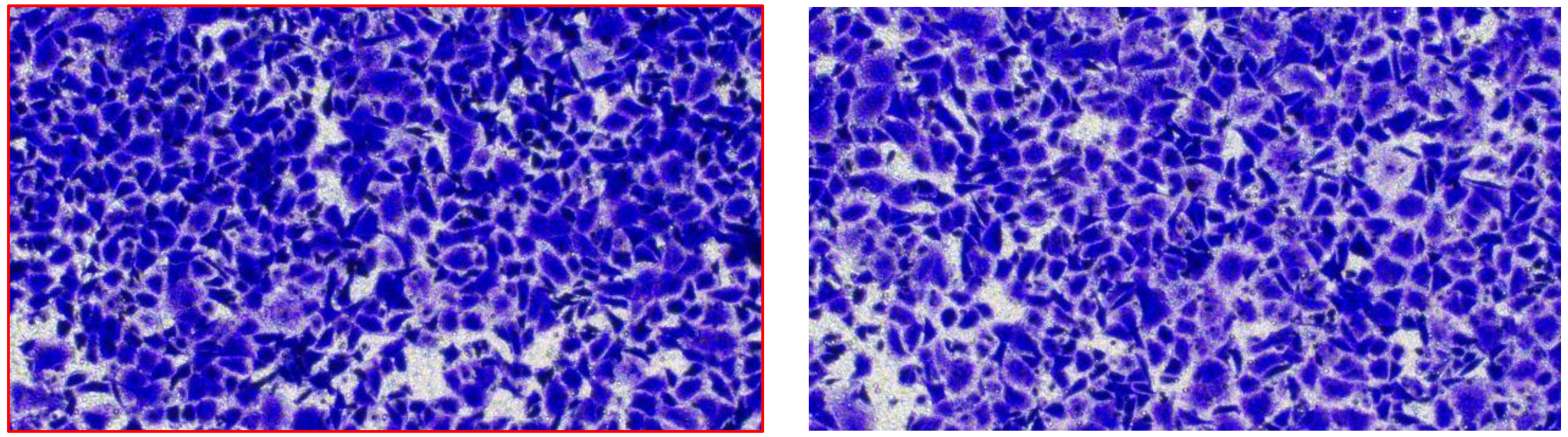

Figure 4H: shSERPINE1-1 (three repeats)

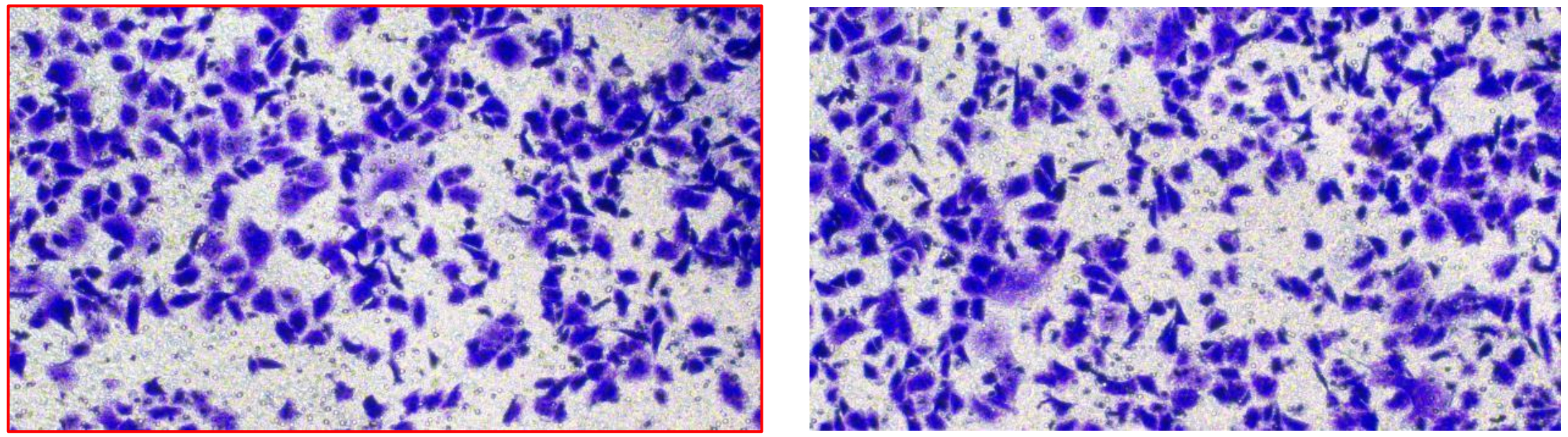

Figure 4H: shSERPINE1-2 (three repeats)

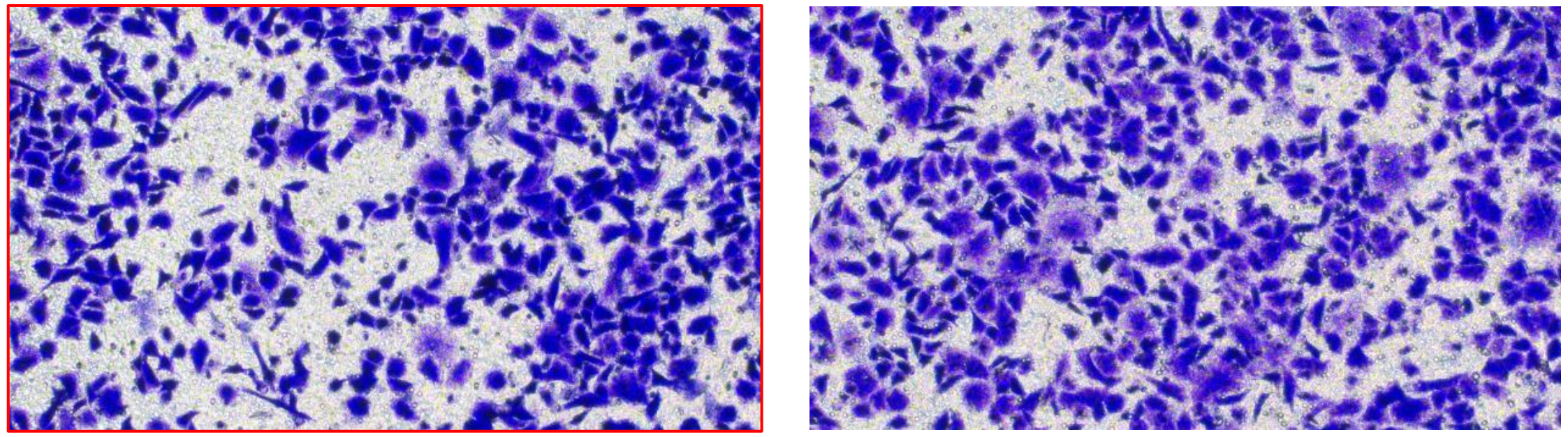

Figure 4I: Vector (three repeats)

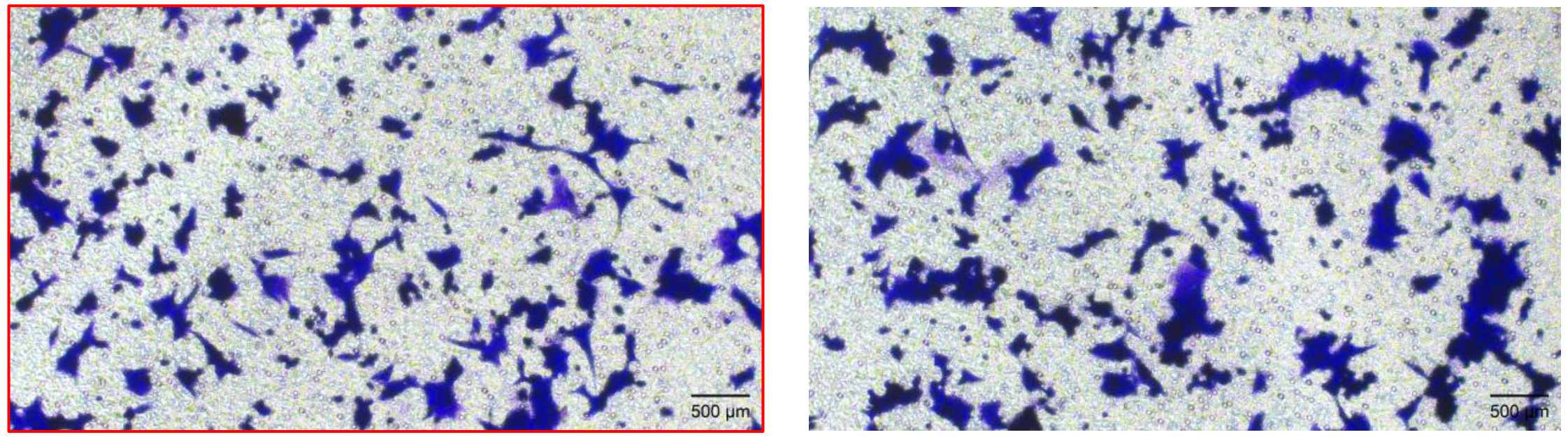

Figure 4I: SERPINE1 (three repeats)

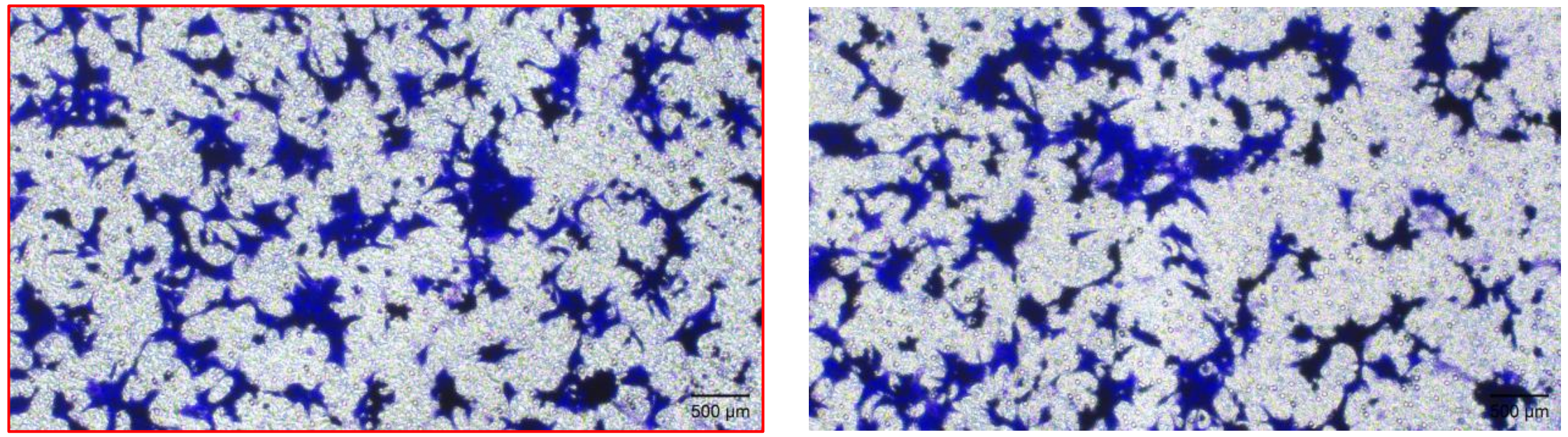

Figure 5A: 0h Control (three repeats)

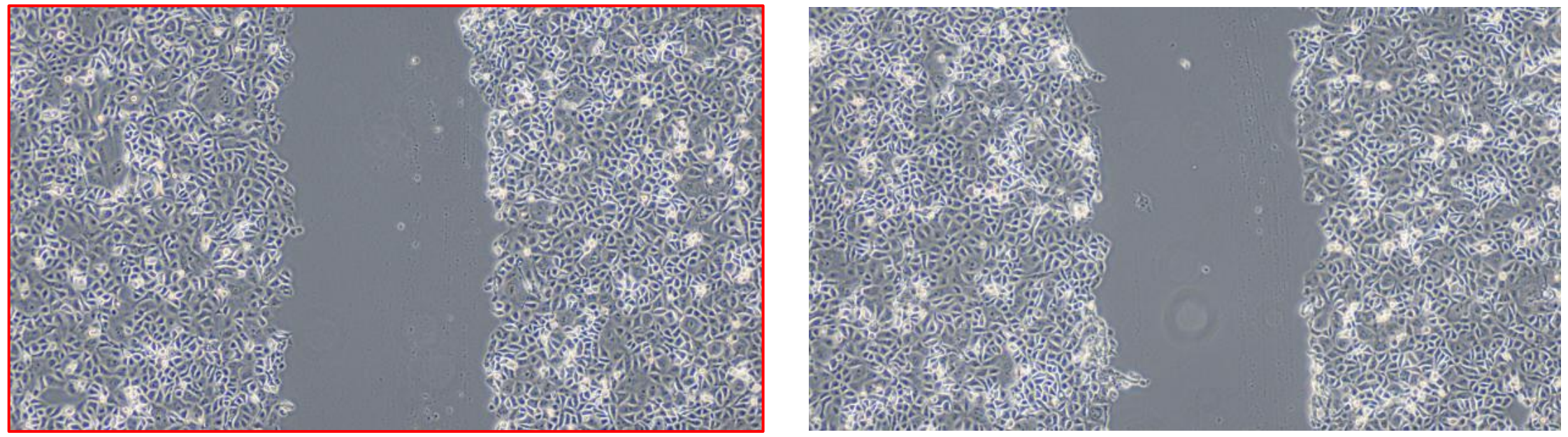

Figure 5A: 24h Control (three repeats)

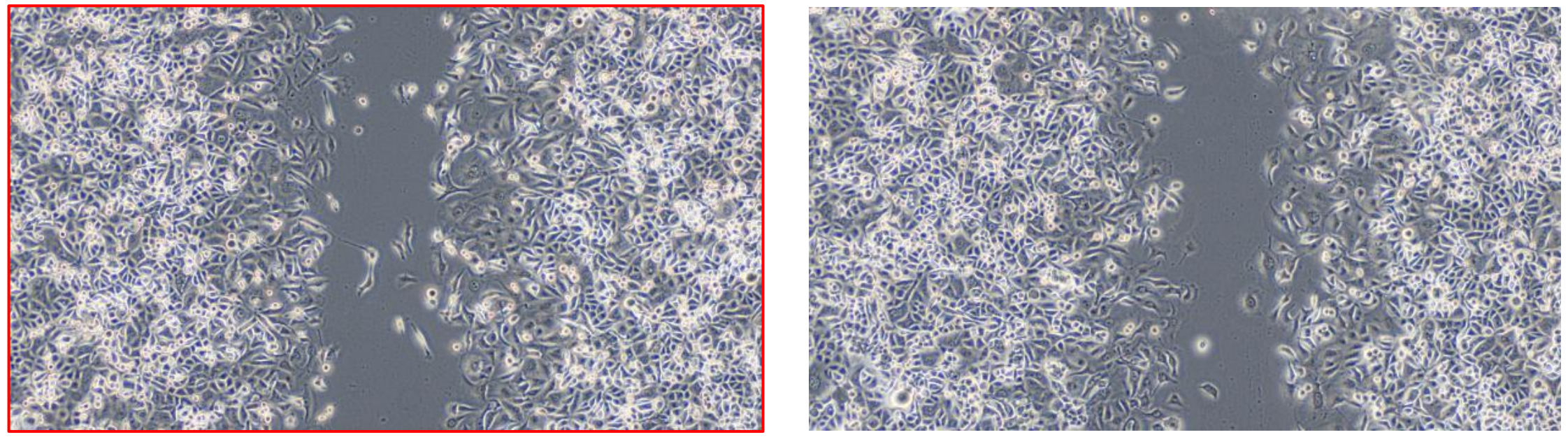

Figure 5A: 0h shARNTL2 (three repeats)

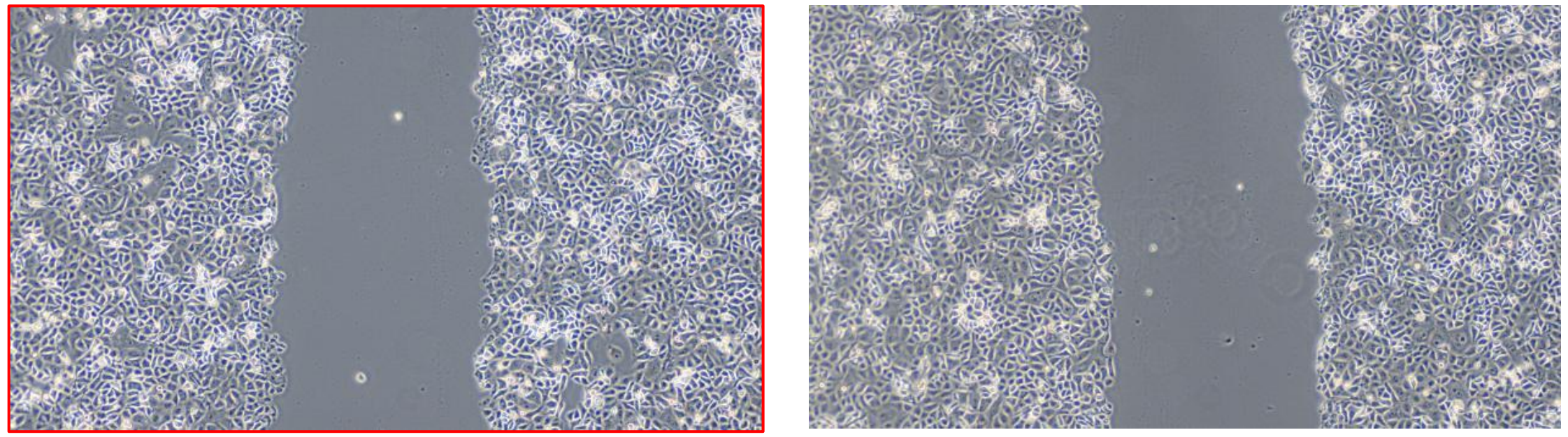

Figure 5A: 24h shARNTL2 (three repeats)

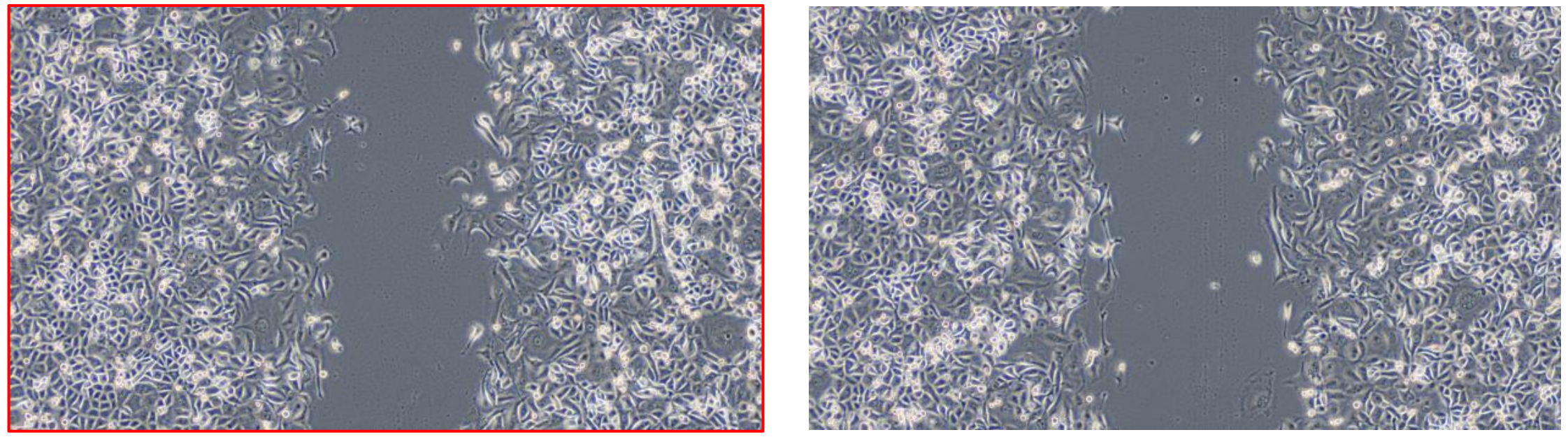

Figure 5A: 0h shARNTL2+SERPINE1 (three repeats)

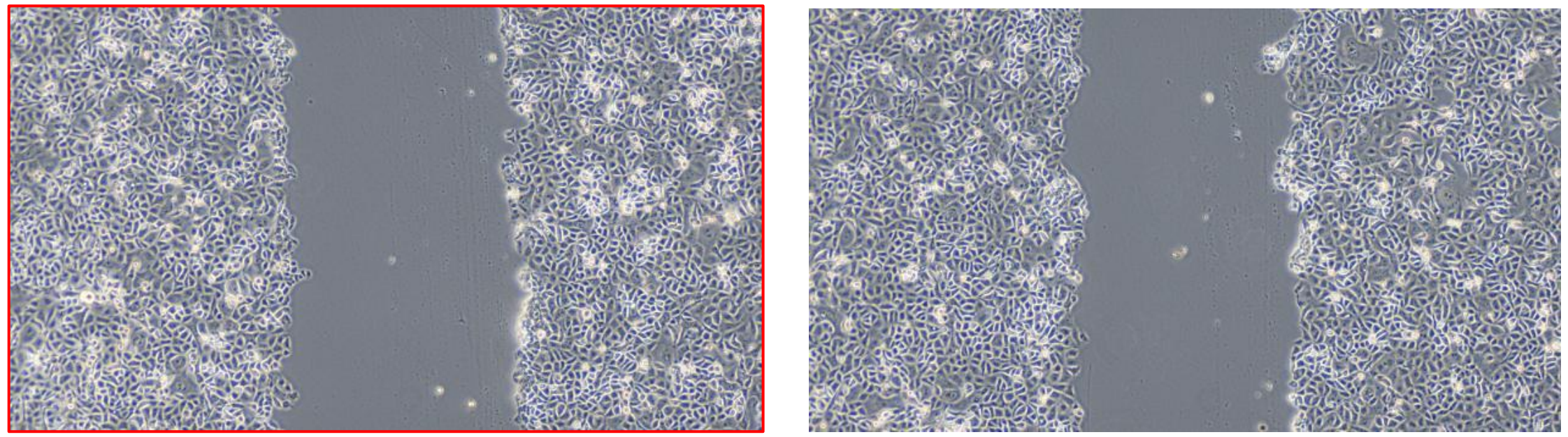

Figure 5A: 24h shARNTL2+SERPINE1 (three repeats)

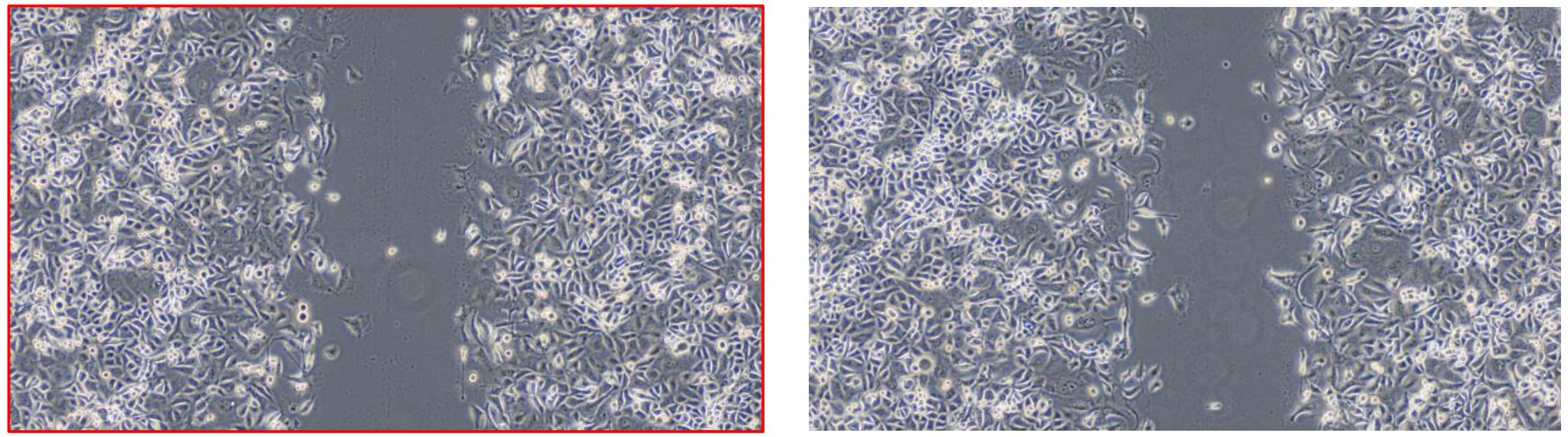

Figure 5B: 0h Control (three repeats)

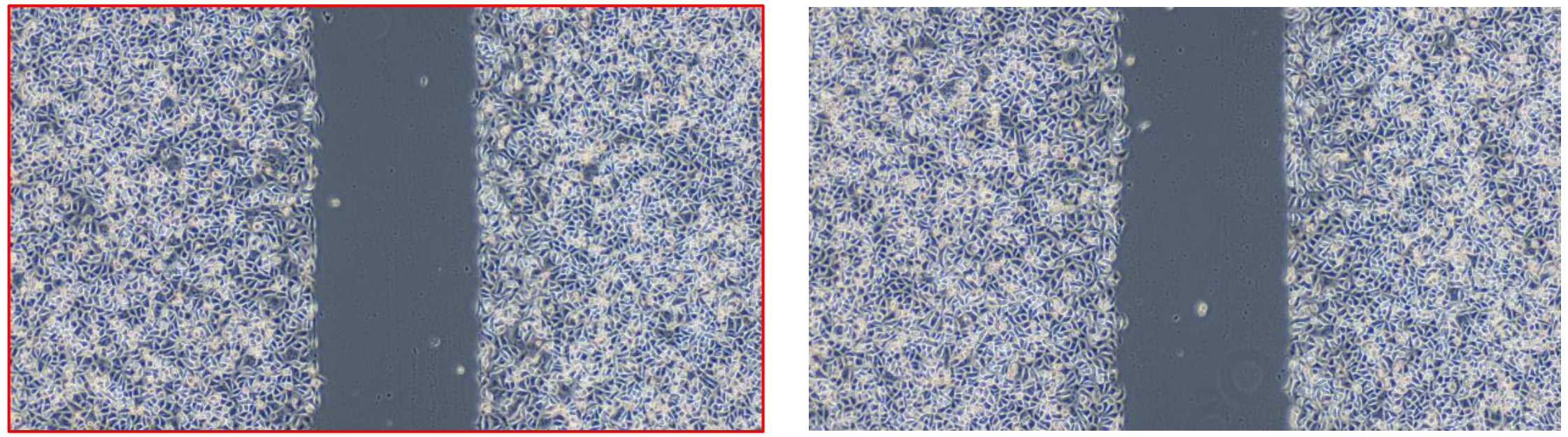

Figure 5B: 24h Control (three repeats)

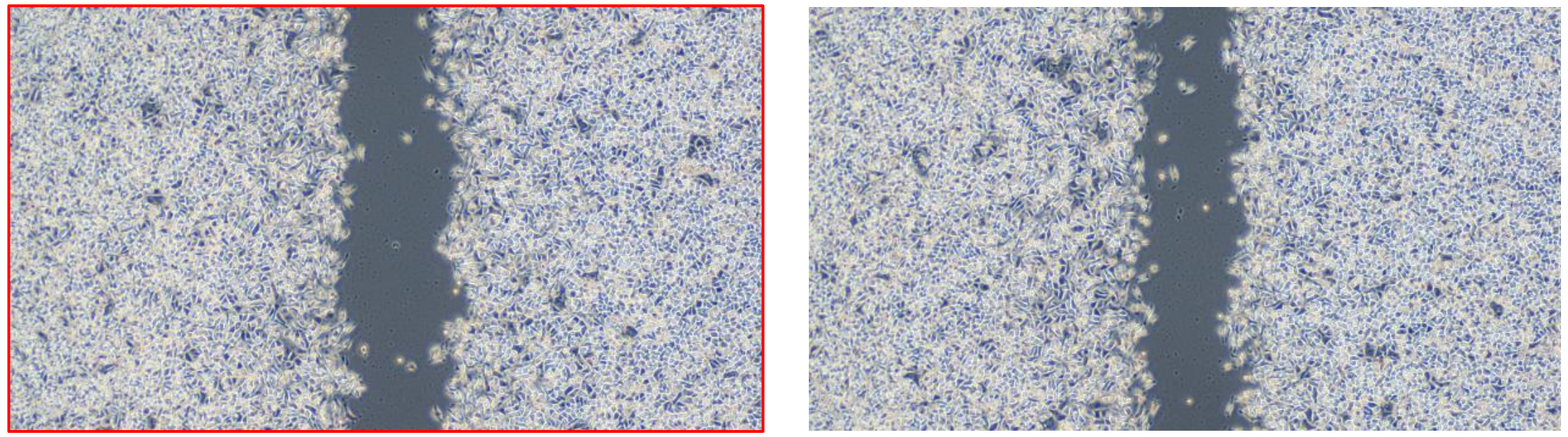

Figure 5B: 0h ARNTL2 (three repeats)

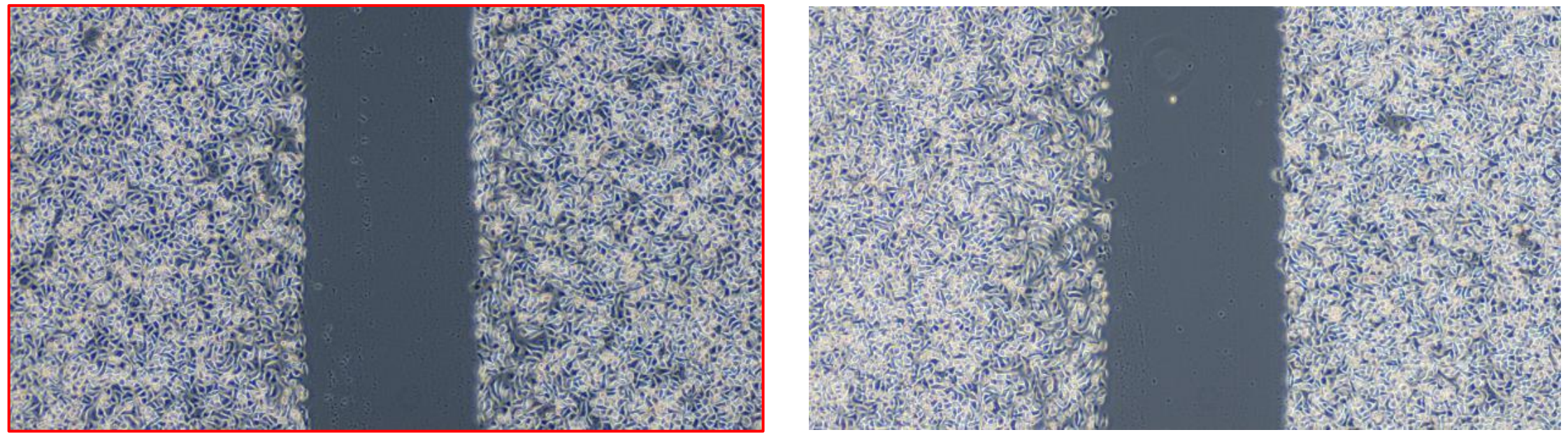

Figure 5B: 24h ARNTL2 (three repeats)

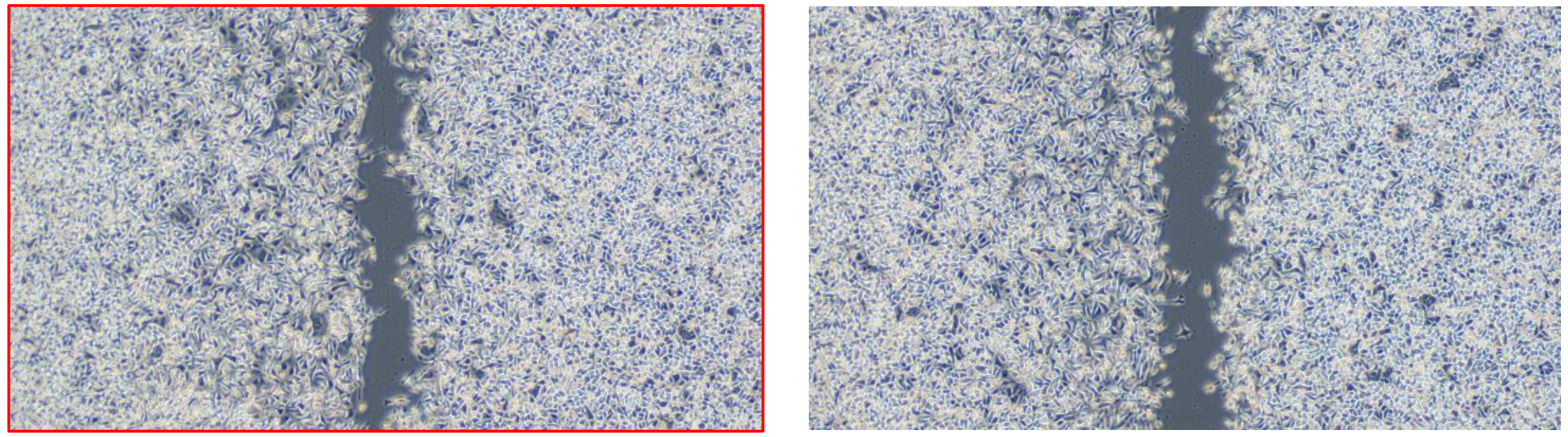

Figure 5B: 0h ARNTL2+shSERPINE1 (three repeats)

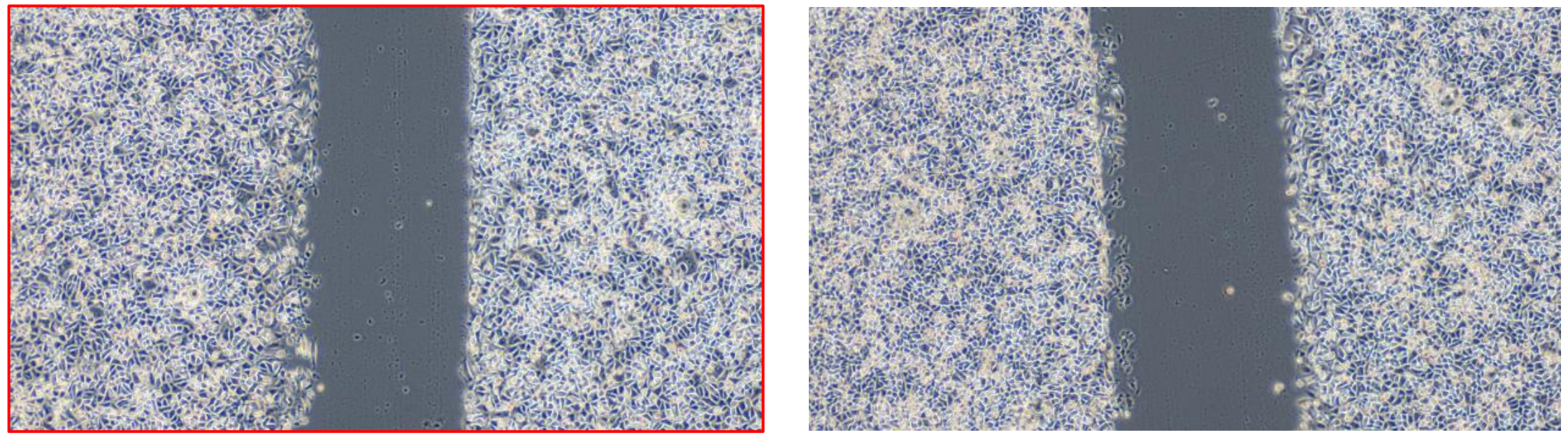

Figure 5B: 24h ARNTL2+shSERPINE1 (three repeats)

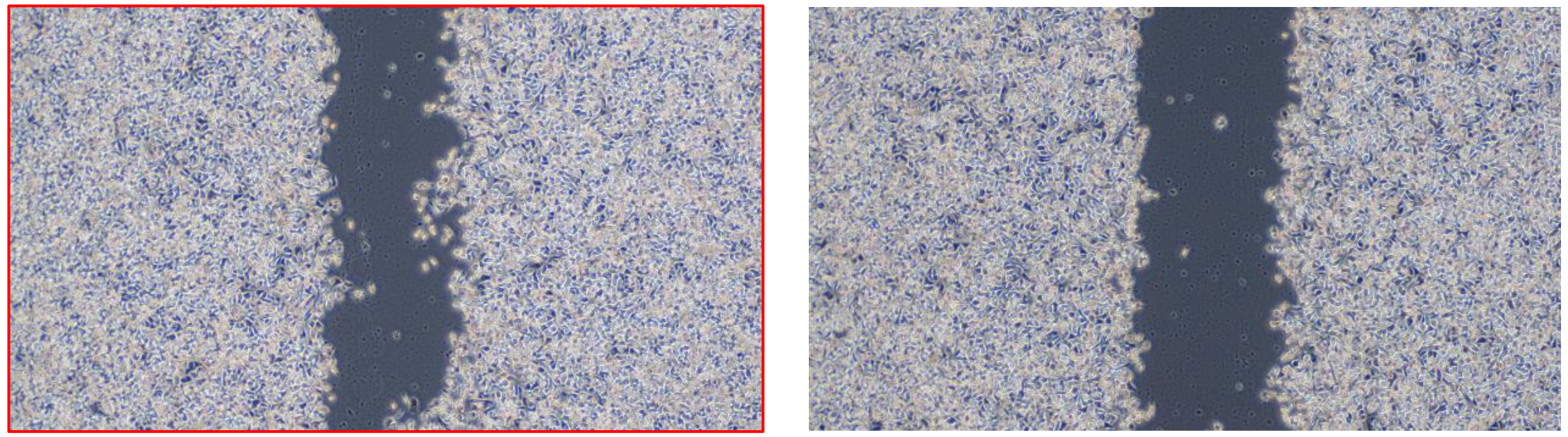

Figure 5C: Control (three repeats)

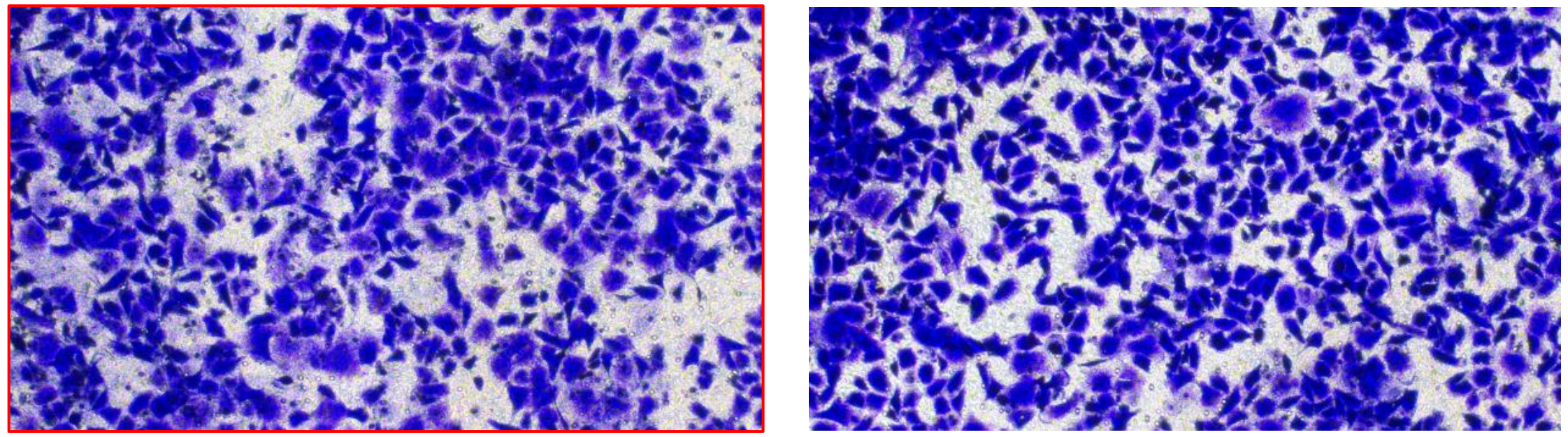

Figure 5C: shARNTL2 (three repeats)

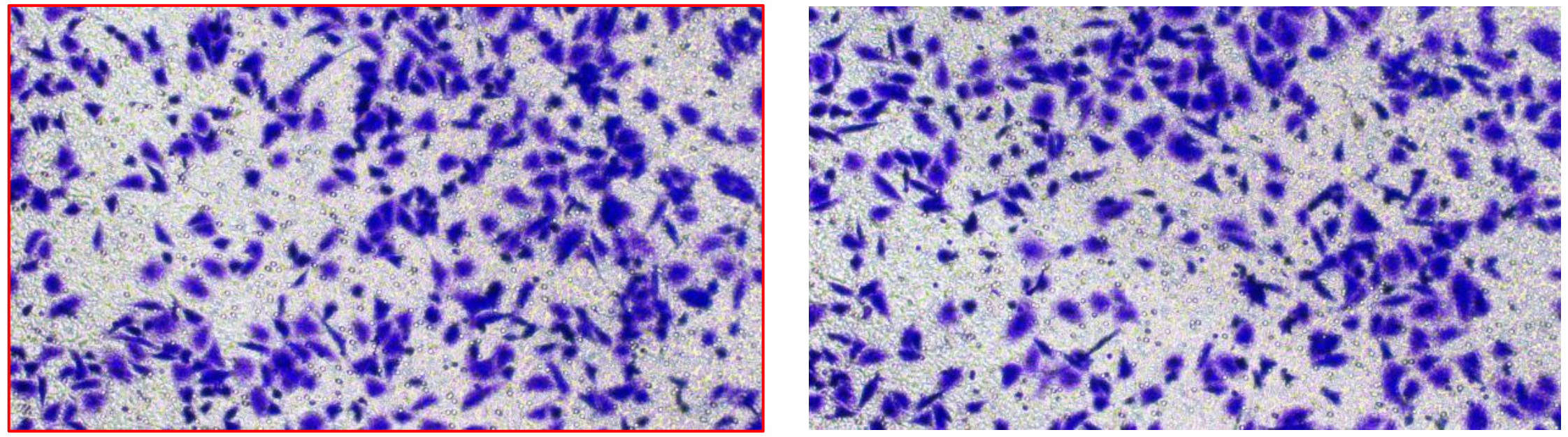

Figure 5C: shARNTL2+SERPINE1 (three repeats)

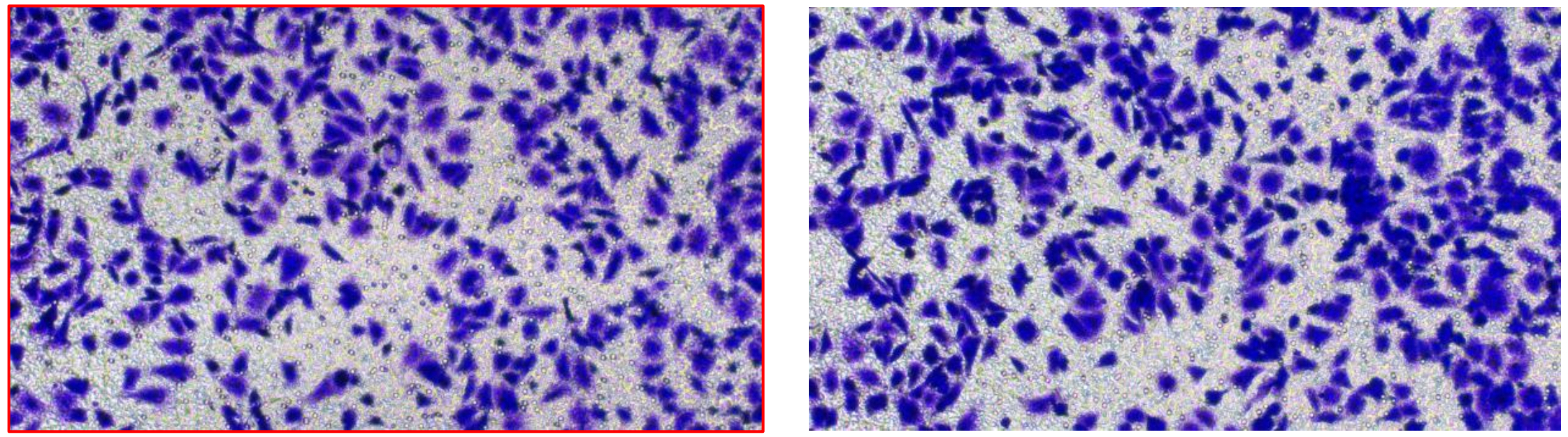

Figure 5D: Control (three repeats)

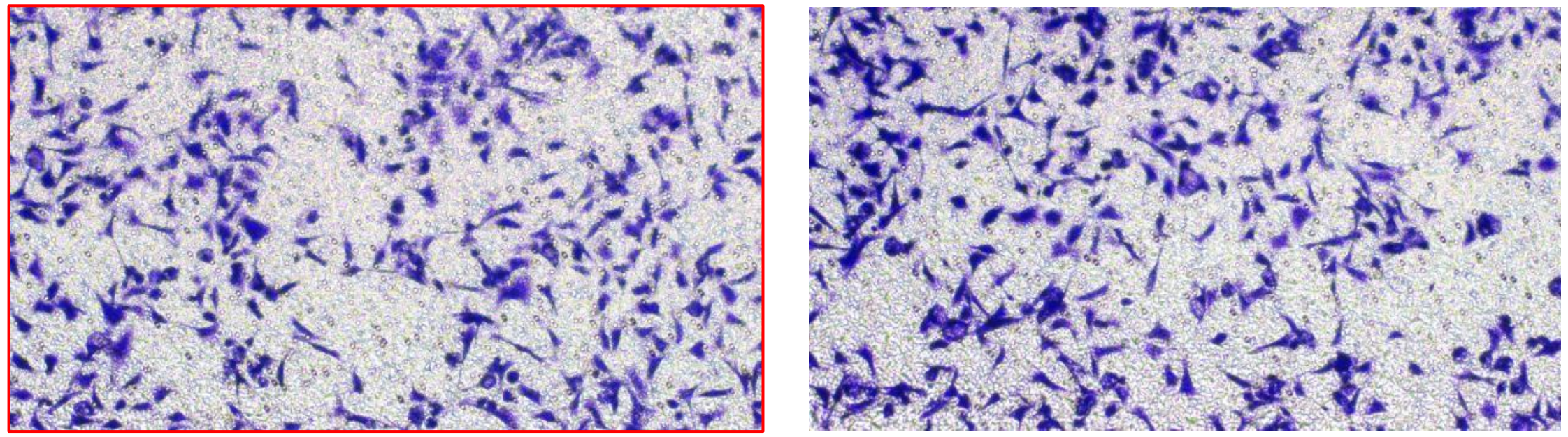

Figure 5D: ARNTL2 (three repeats)

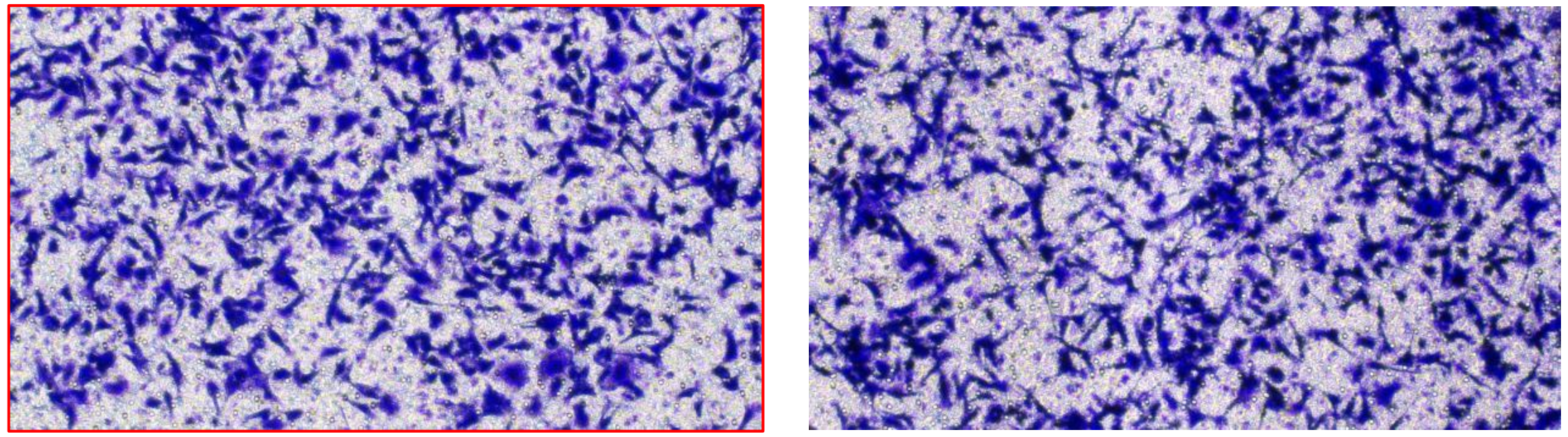

Figure 5D: ARNTL2+shSERPINE1 (three repeats)

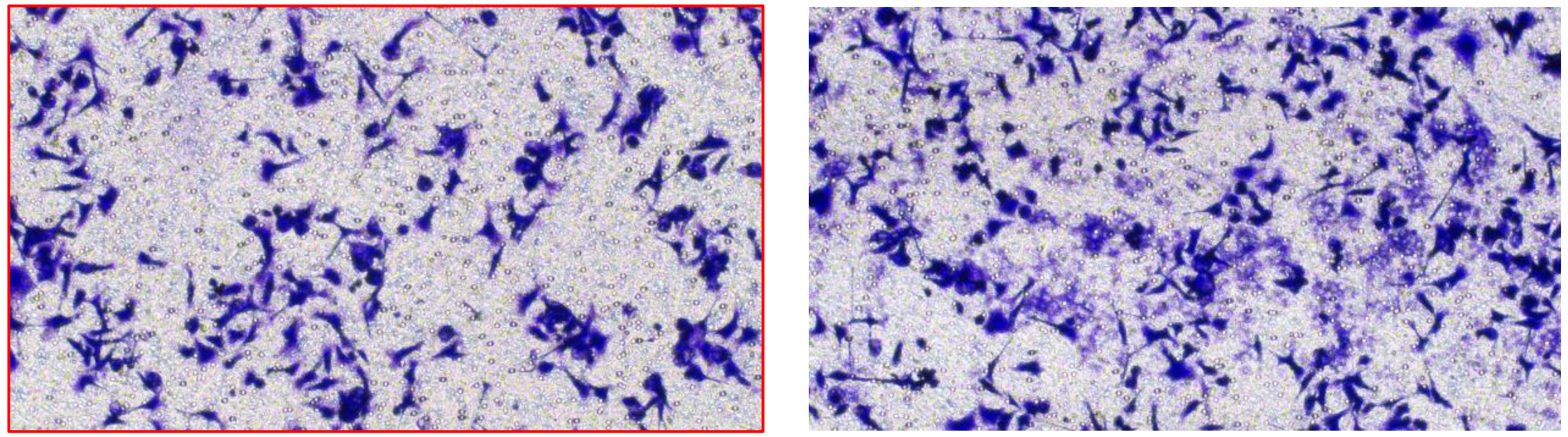

Figure 5E: Control (three repeats)

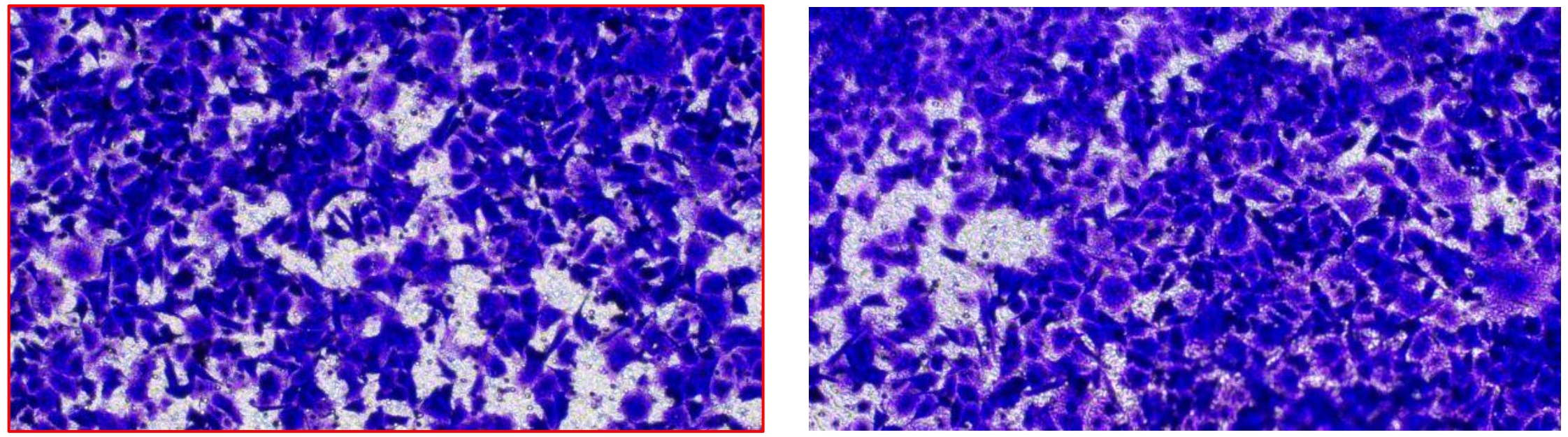

Figure 5E: shARNTL2 (three repeats)

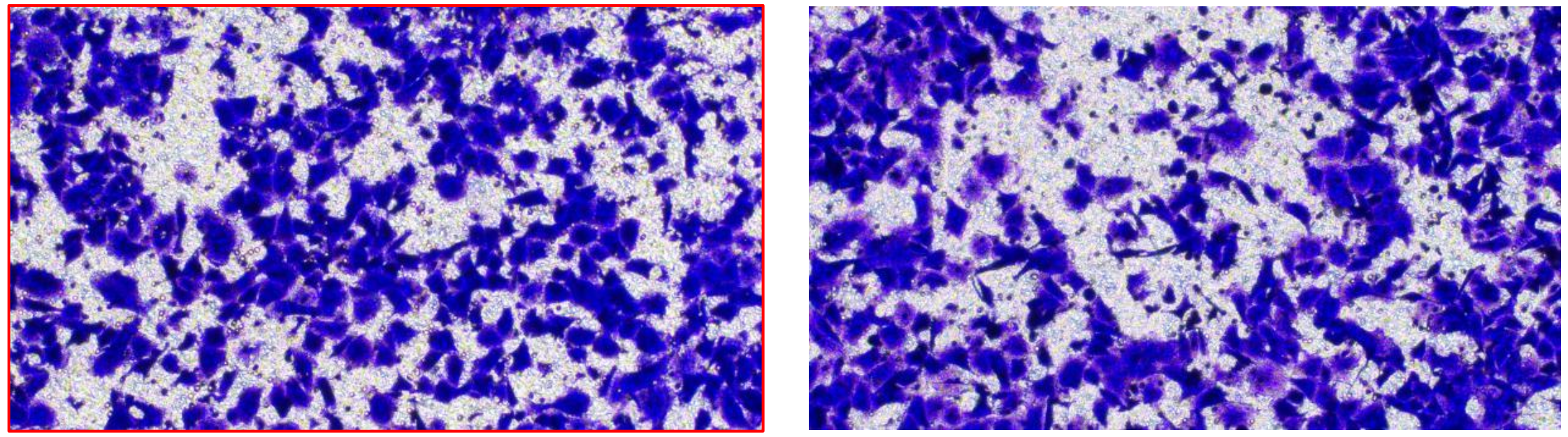

Figure 5E: shARNTL2+SERPINE1 (three repeats)

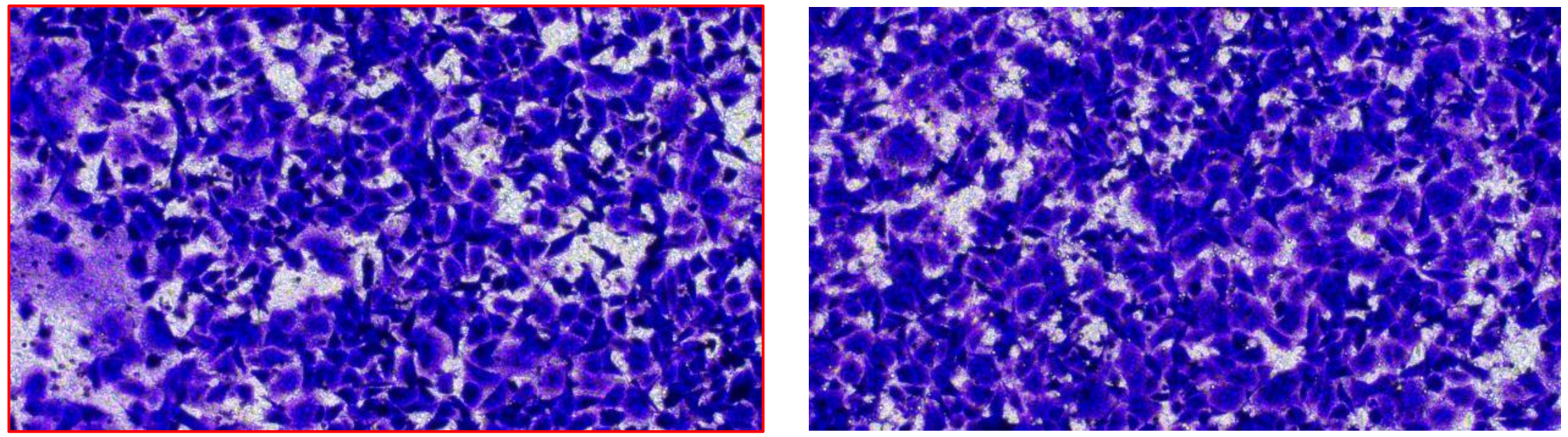

Figure 5F: Control (three repeats)

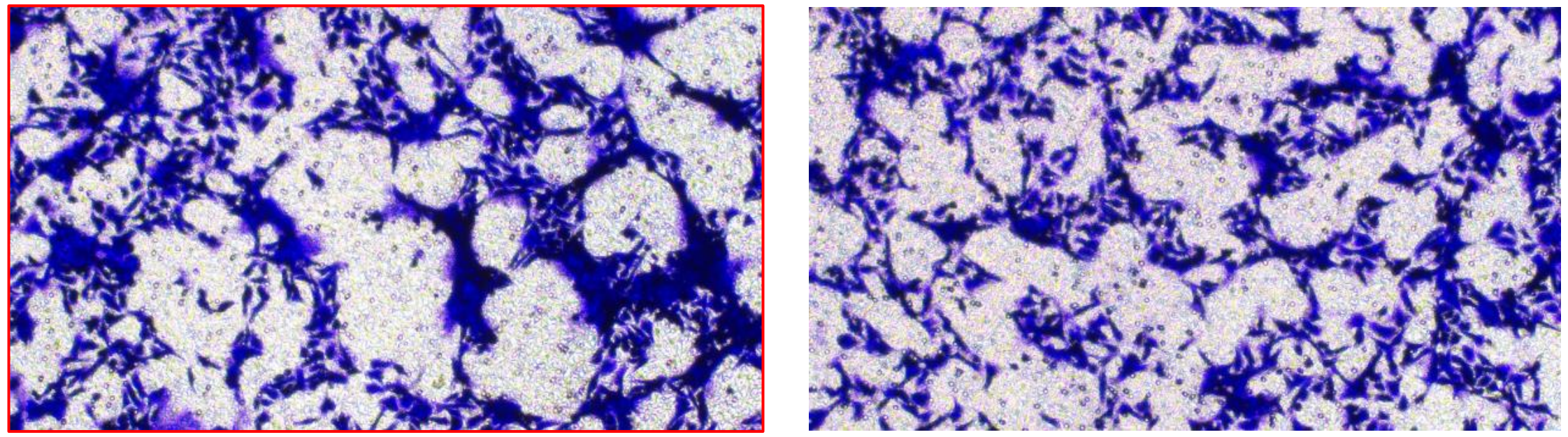

Figure 5F: ARNTL2 (three repeats)

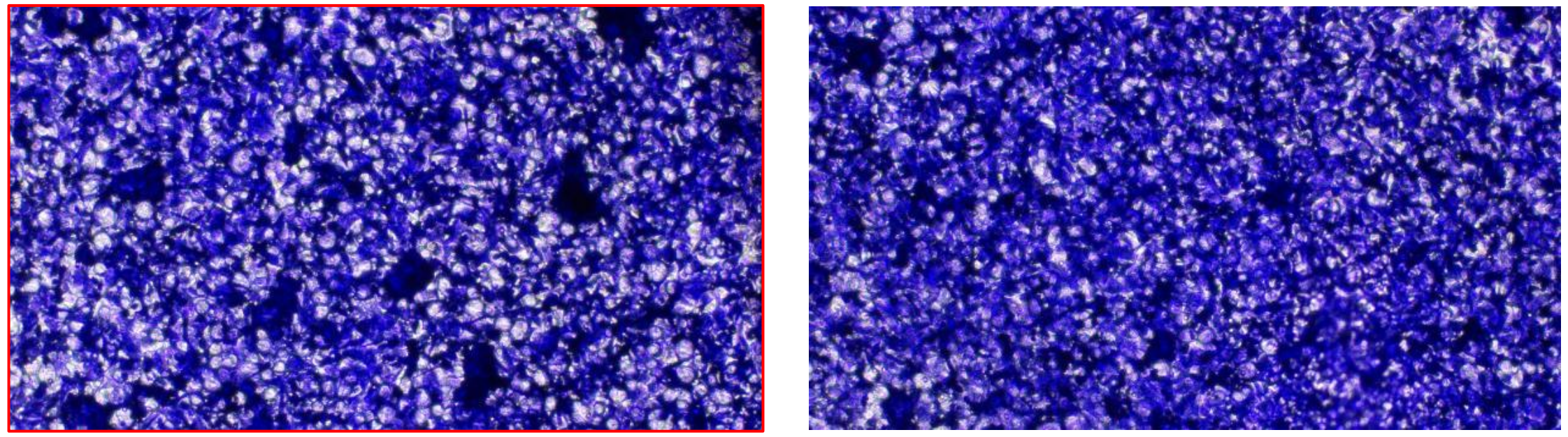

Figure 5F: ARNTL2+shSERPINE1 (three repeats)

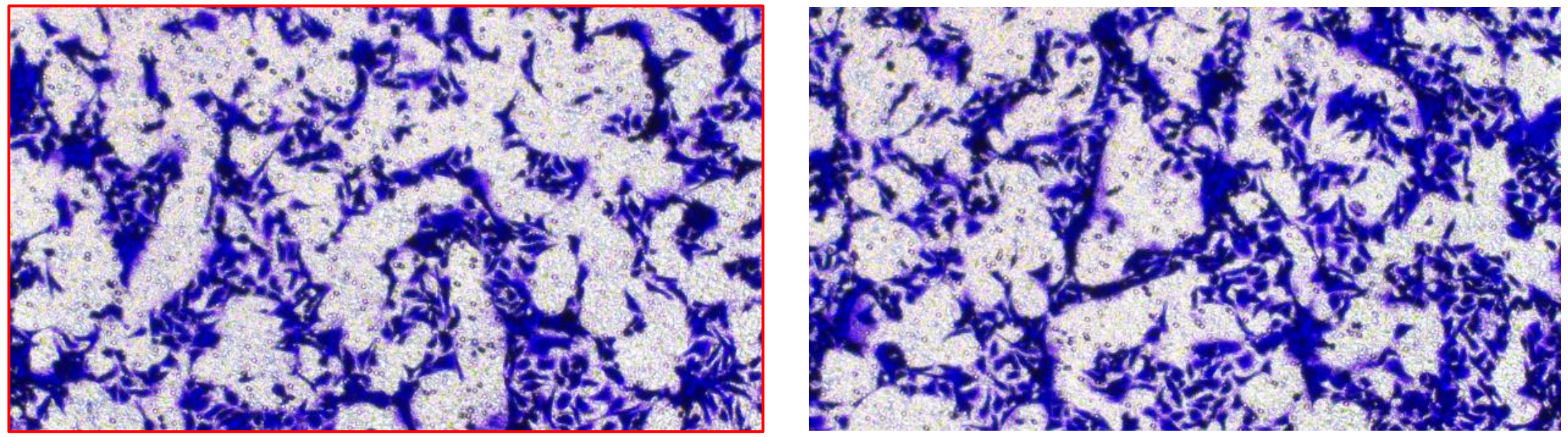

Figure 6G 200X: Left (three repeats)

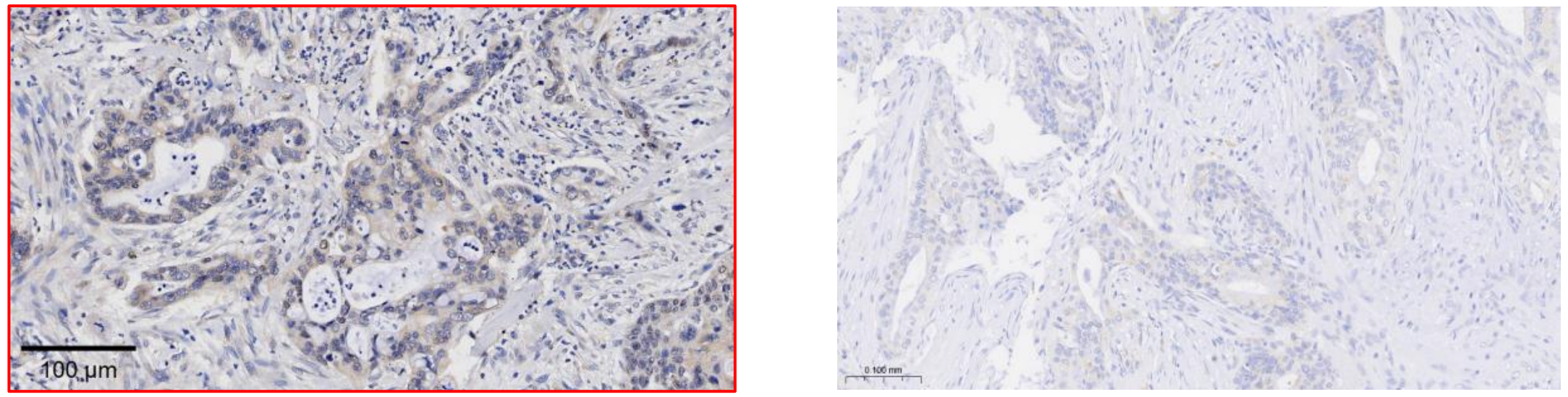

Figure 6G 400X: Left (three repeats)

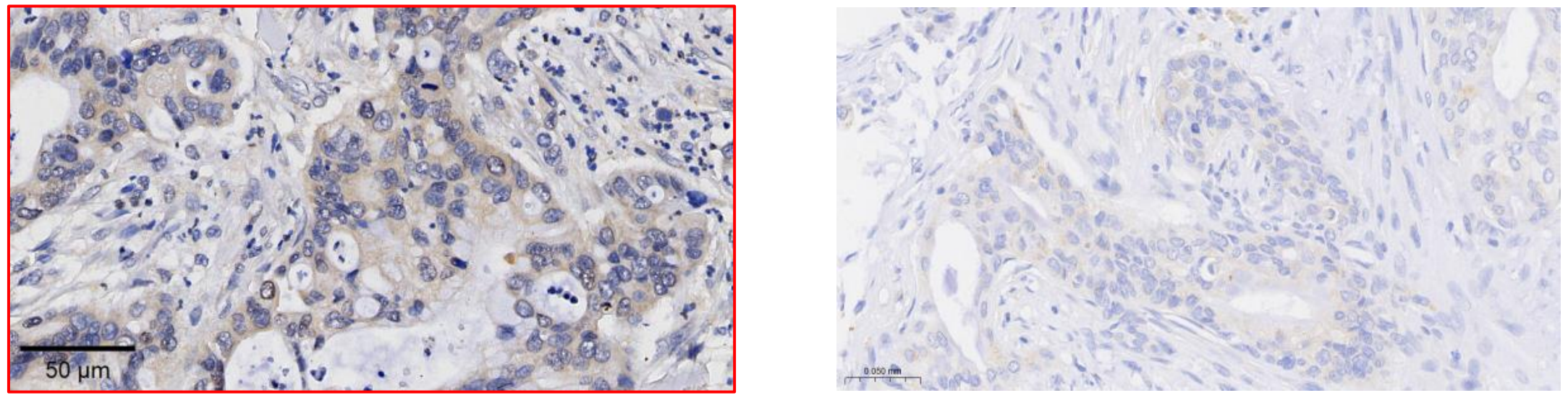

Figure 6G 200X: Right (three repeats)

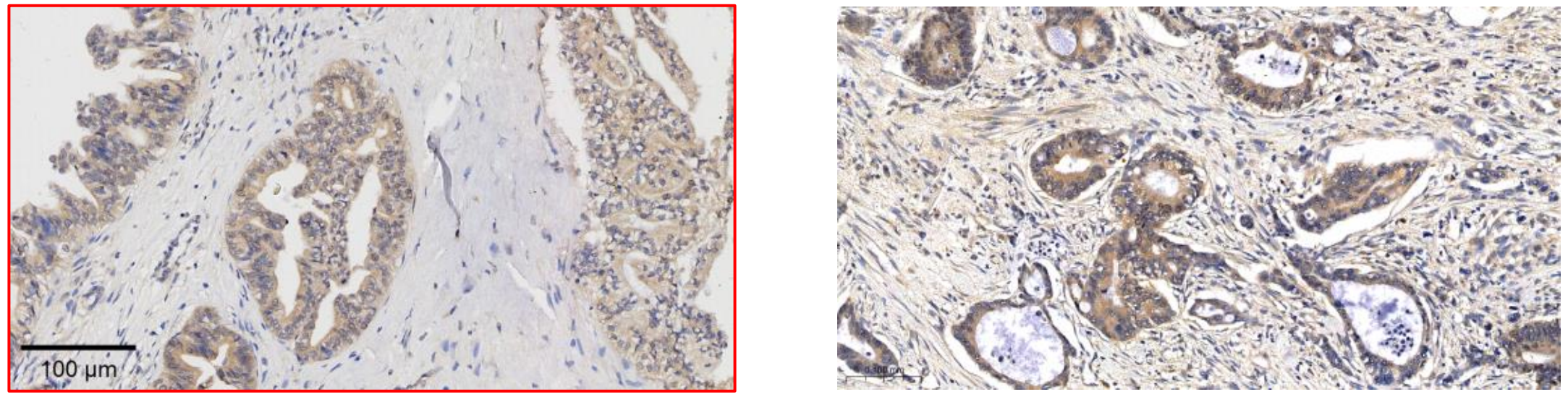

Figure 6G 400X: Right (three repeats)

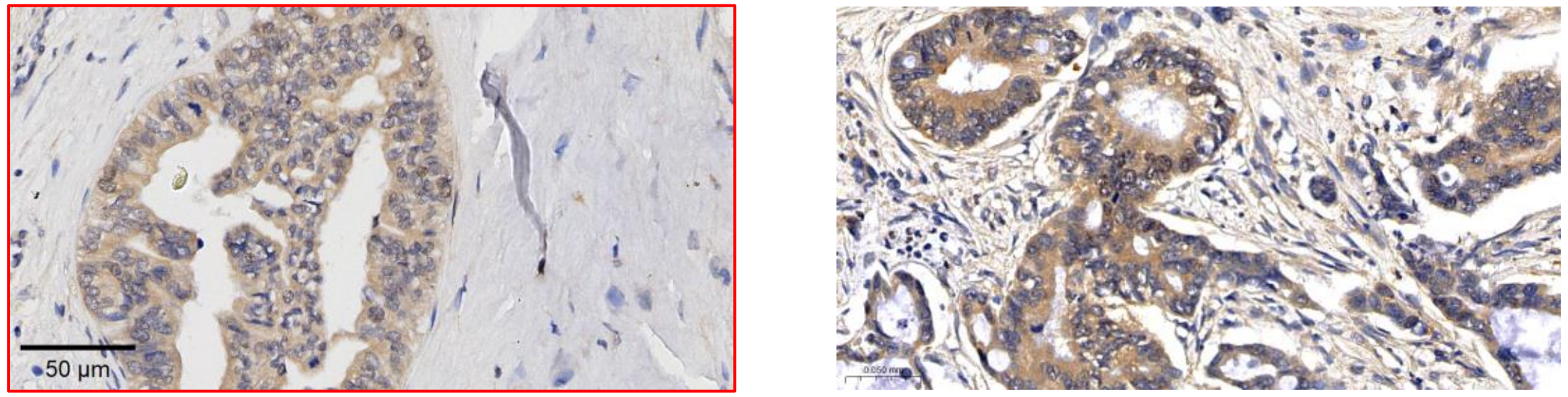

Figure 6I 200X: Left (three repeats)

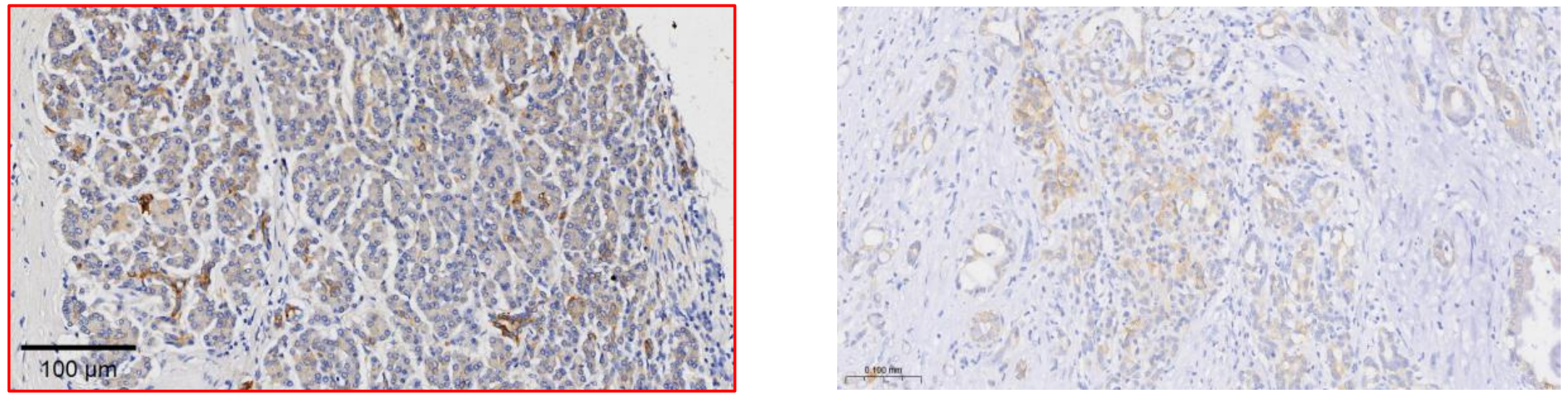

Figure 6I 400X: Left (three repeats)

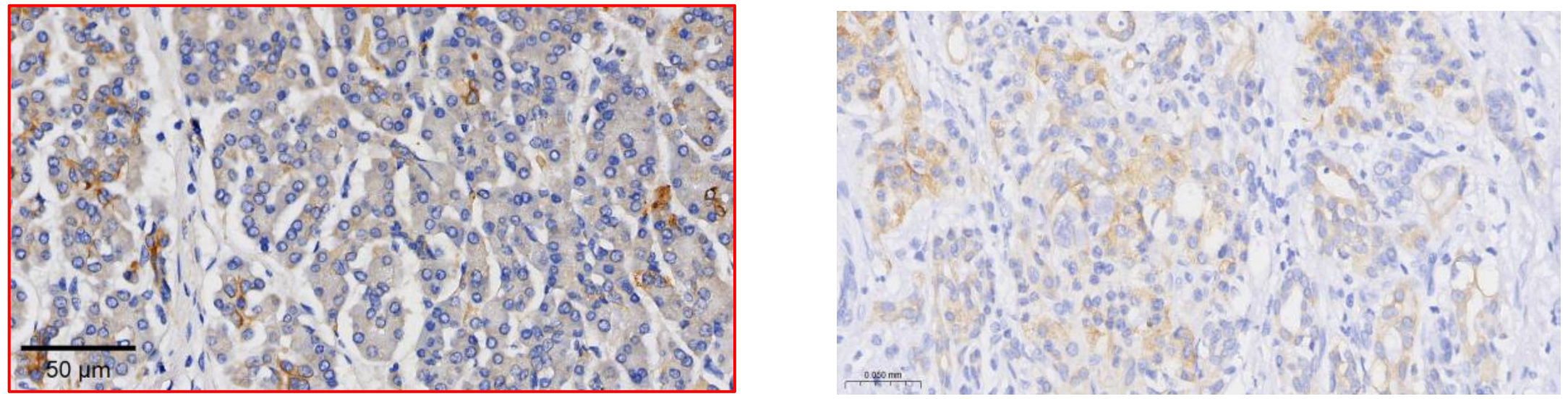

Figure 6I 200X: Right (three repeats)

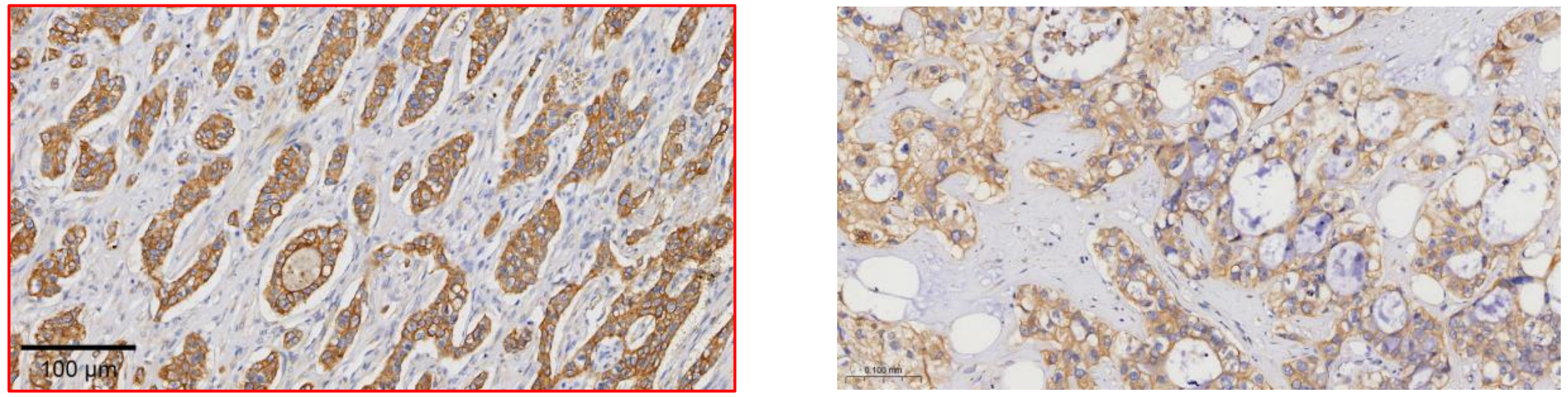

Figure 6I 400X: Right (three repeats)

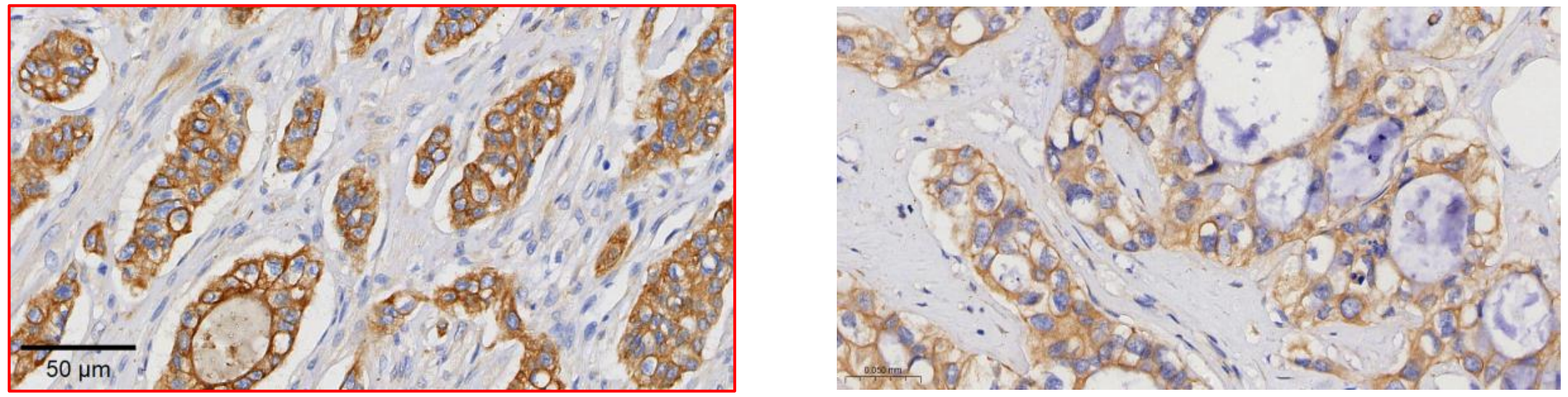

Figure S2K: 0h shNC (three repeats)

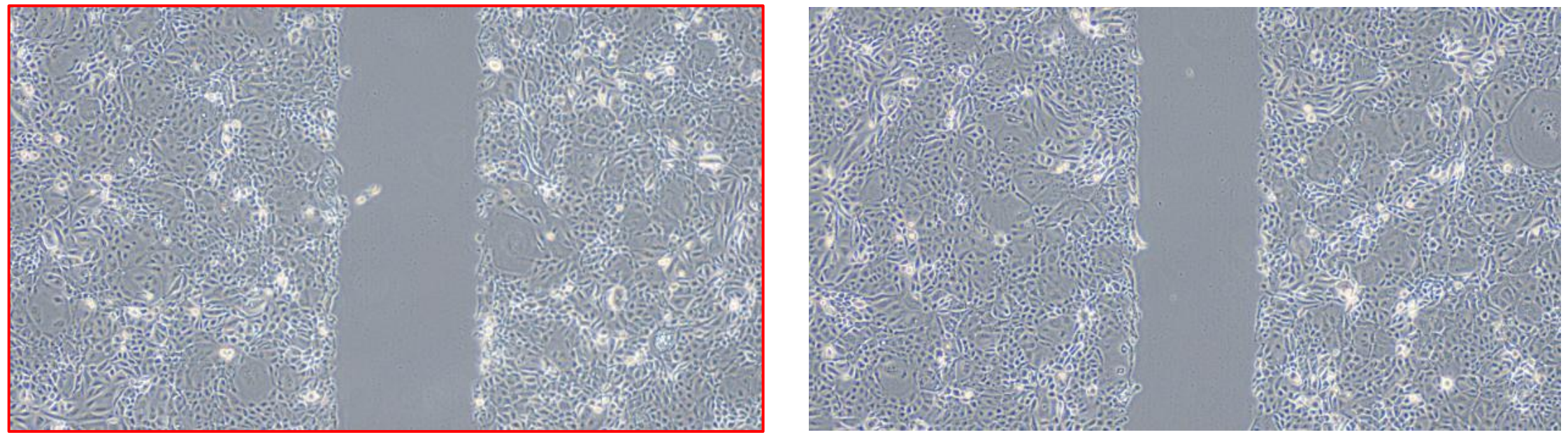

Figure S2K: 24h shNC (three repeats)

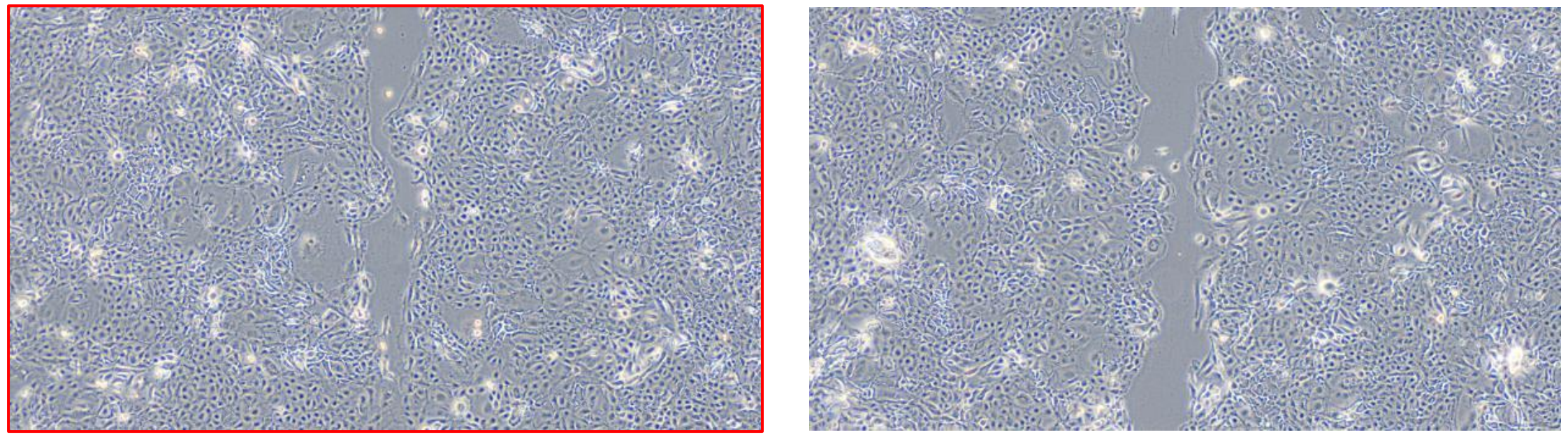

Figure S2K: 0h shARNTL2-1 (three repeats)

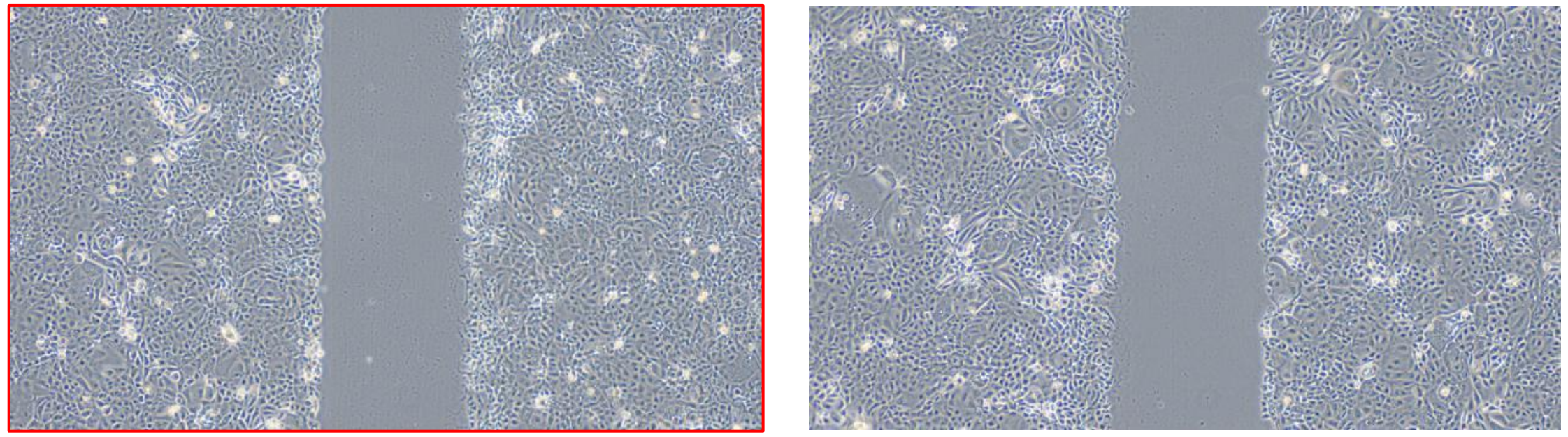

Figure S2K: 24h shARNTL2-1 (three repeats)

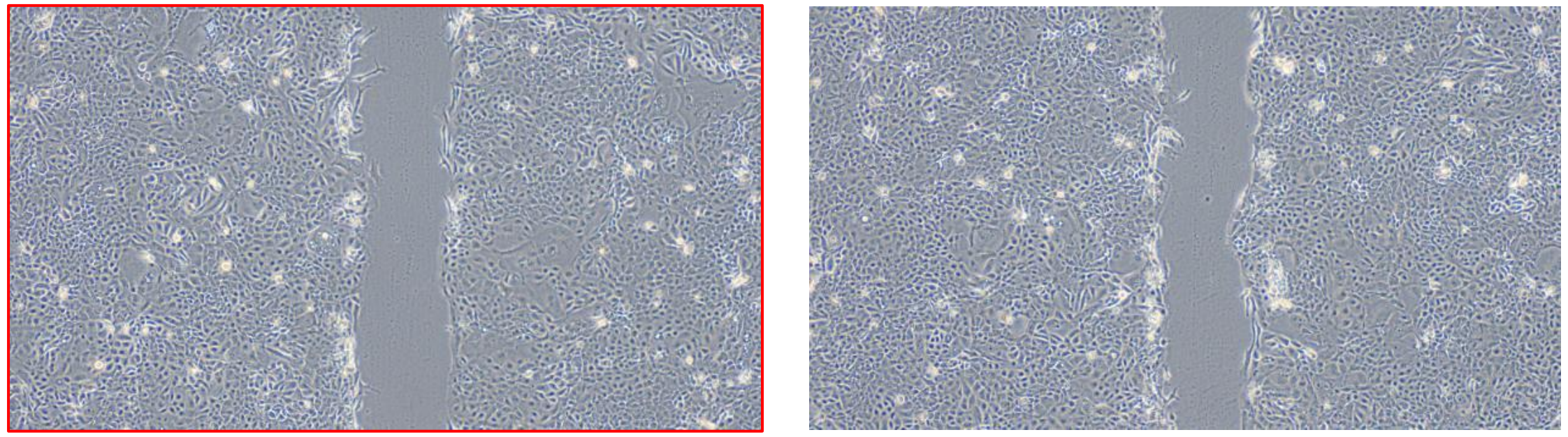

Figure S2K: 0h shARNTL2-2 (three repeats)

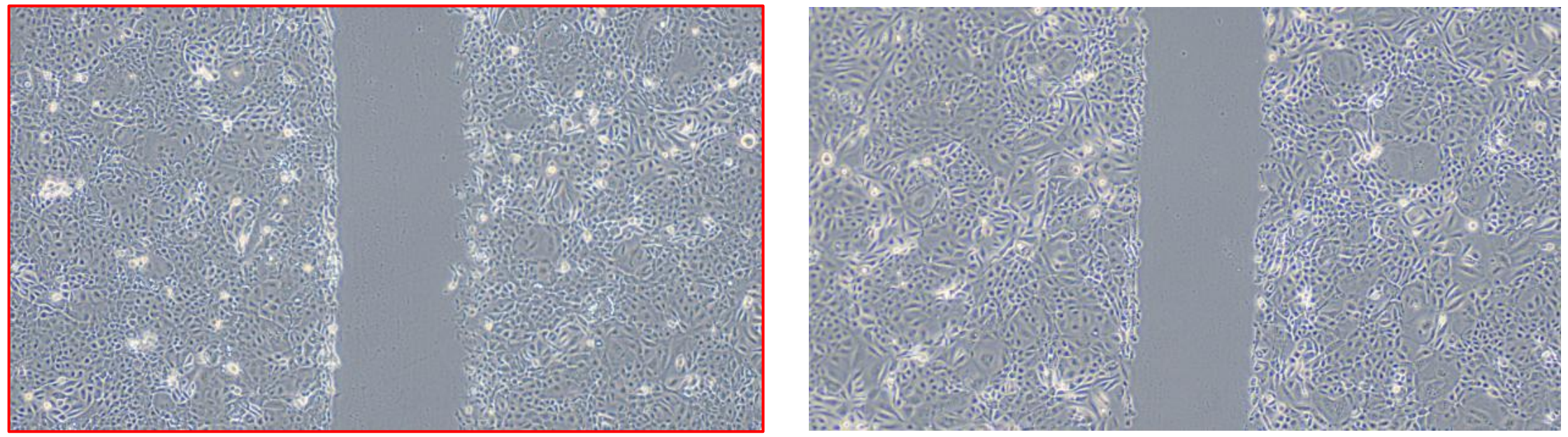

Figure S2K: 24h shARNTL2-2 (three repeats)

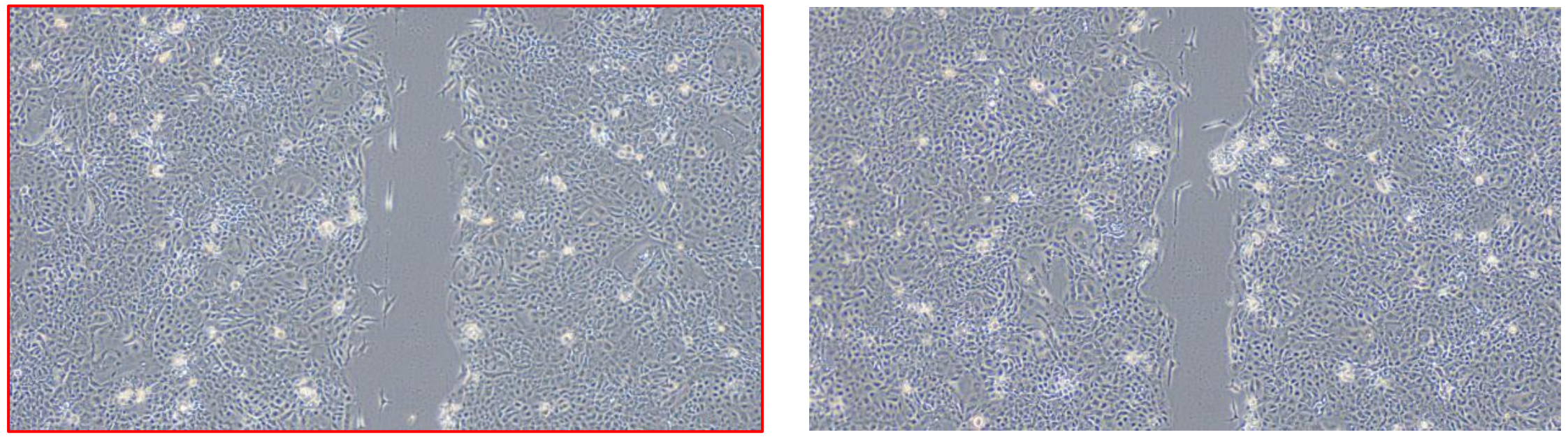

Figure S2L: shNC (three repeats)

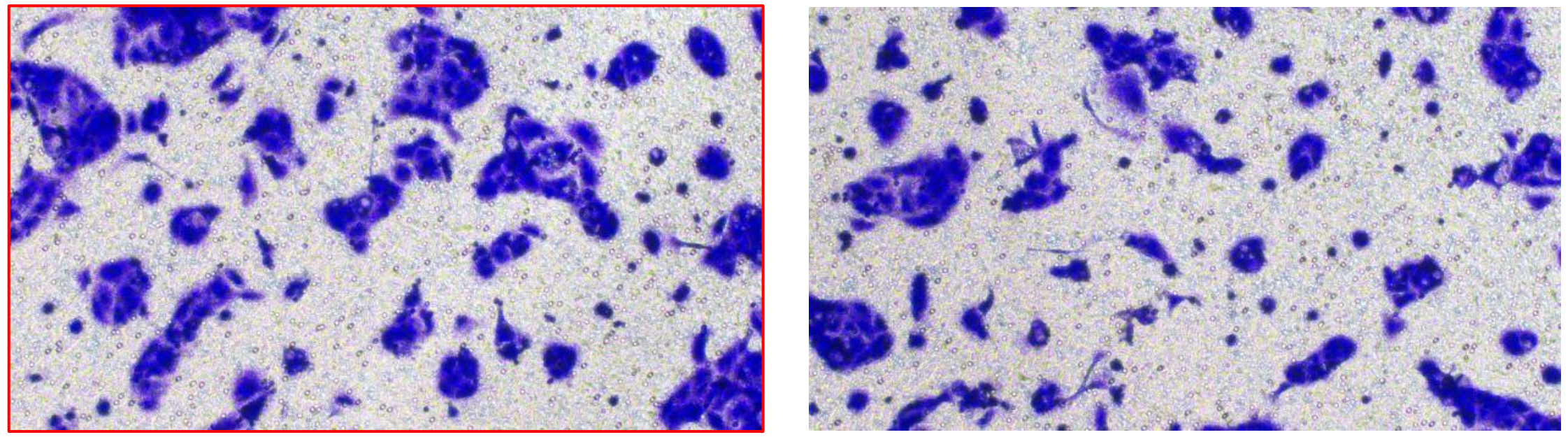

Figure S2L: shARNTL2-1 (three repeats)

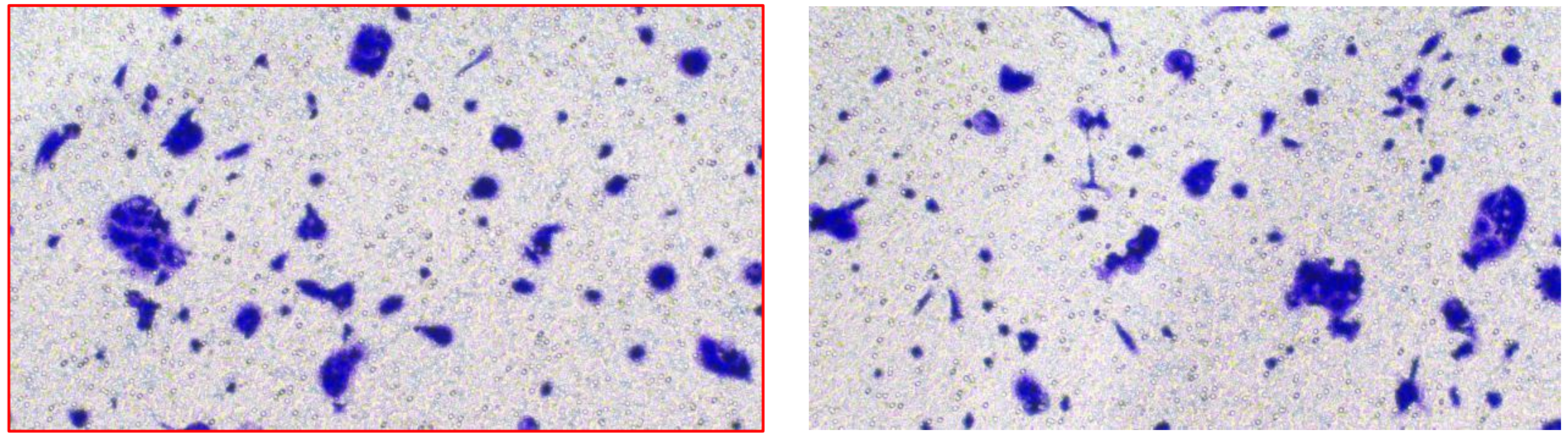

Figure S2L: shARNTL2-2 (three repeats)

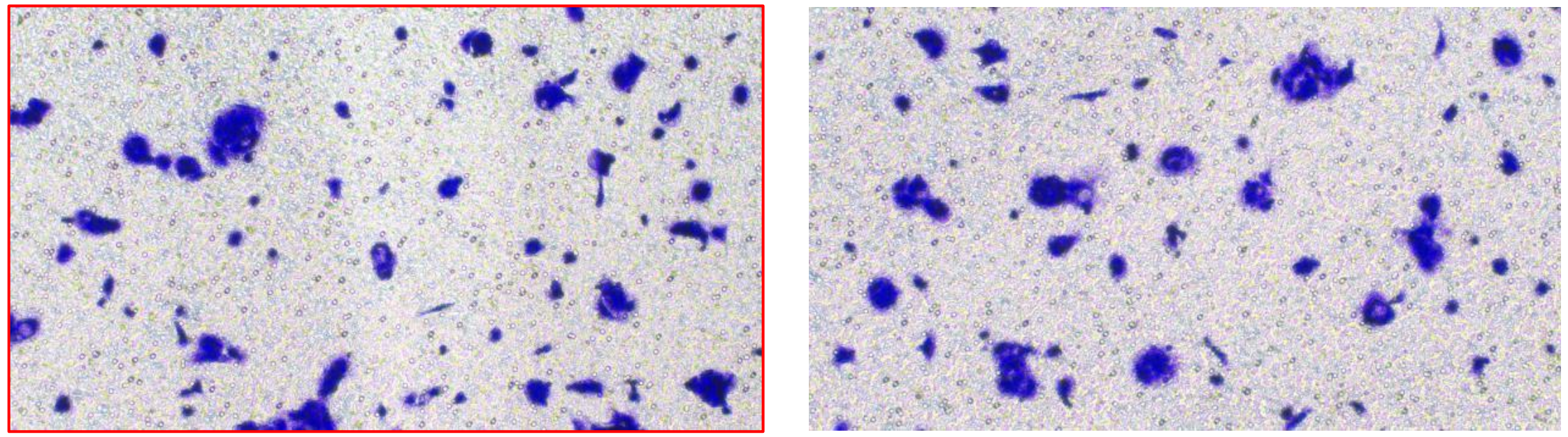

Figure S2M: shNC (three repeats)

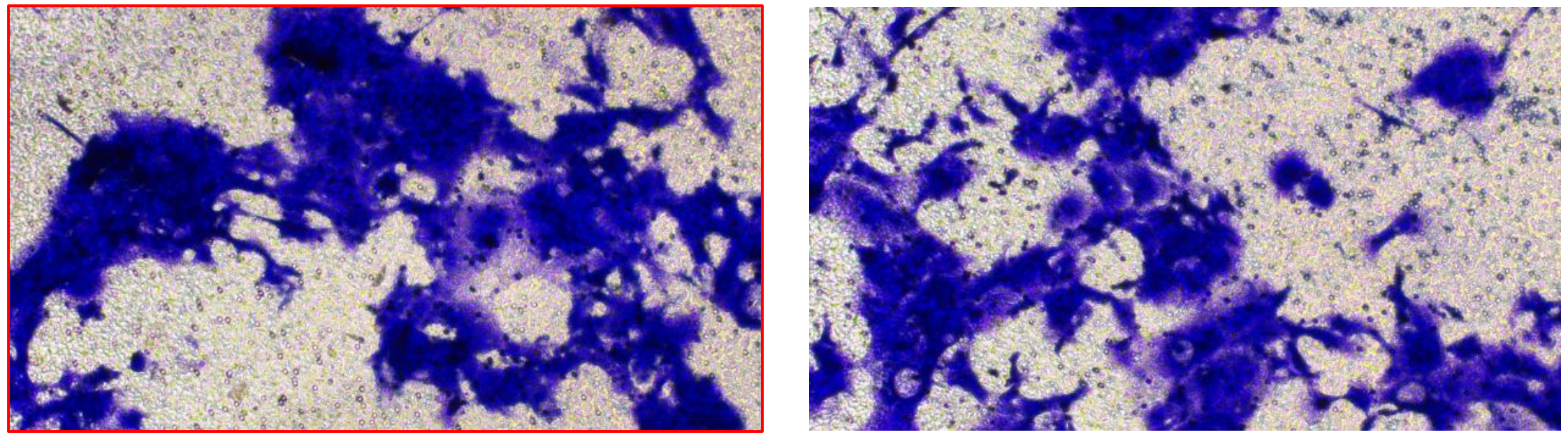

Figure S2M: shARNTL2-1 (three repeats)

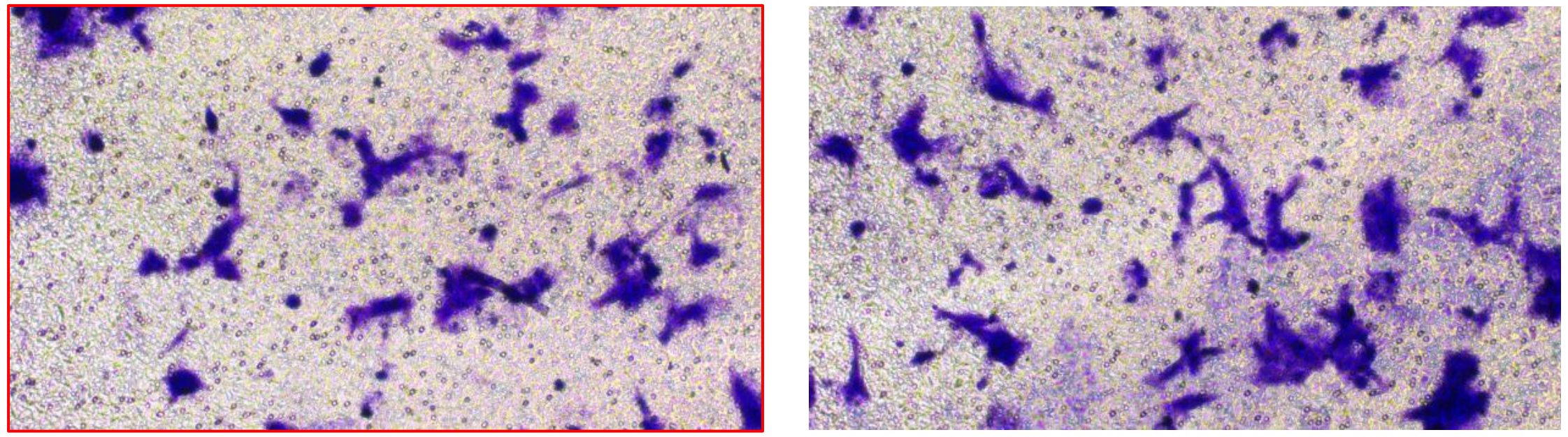

Figure S2M: shARNTL2-2 (three repeats)

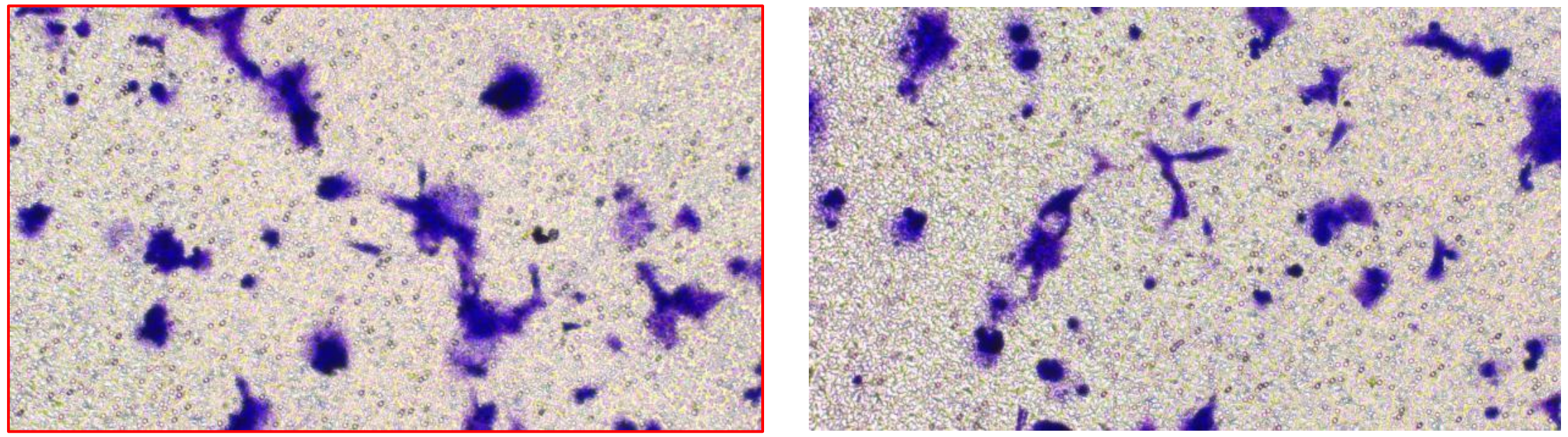

Figure S3A Vector: ARNTL2 (three repeats)

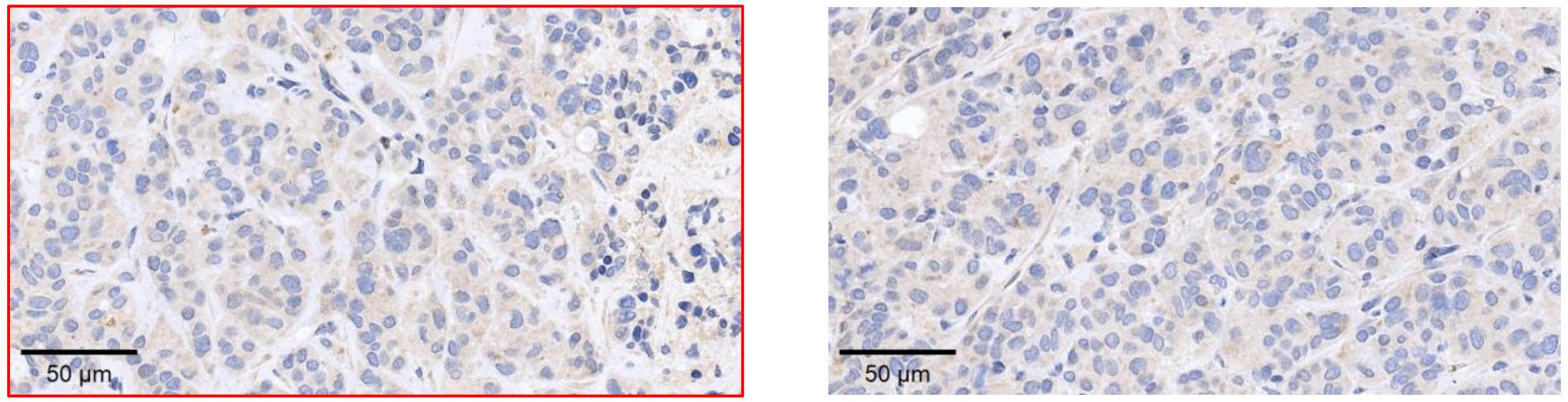

Figure S3A Vector: Vimentin (three repeats)

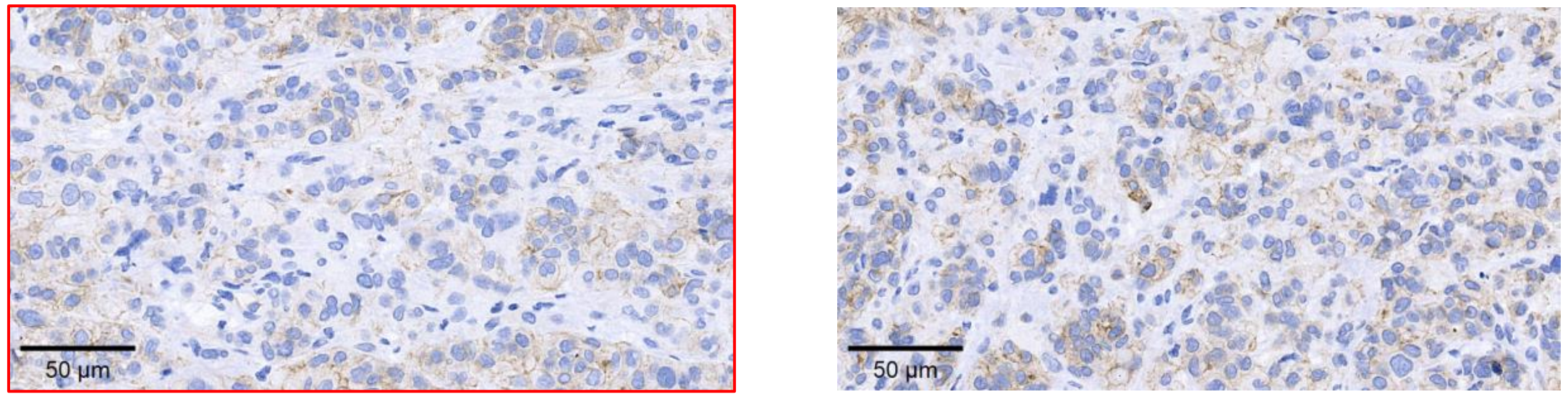

Figure S3A Vector: SERPINE1 (three repeats)

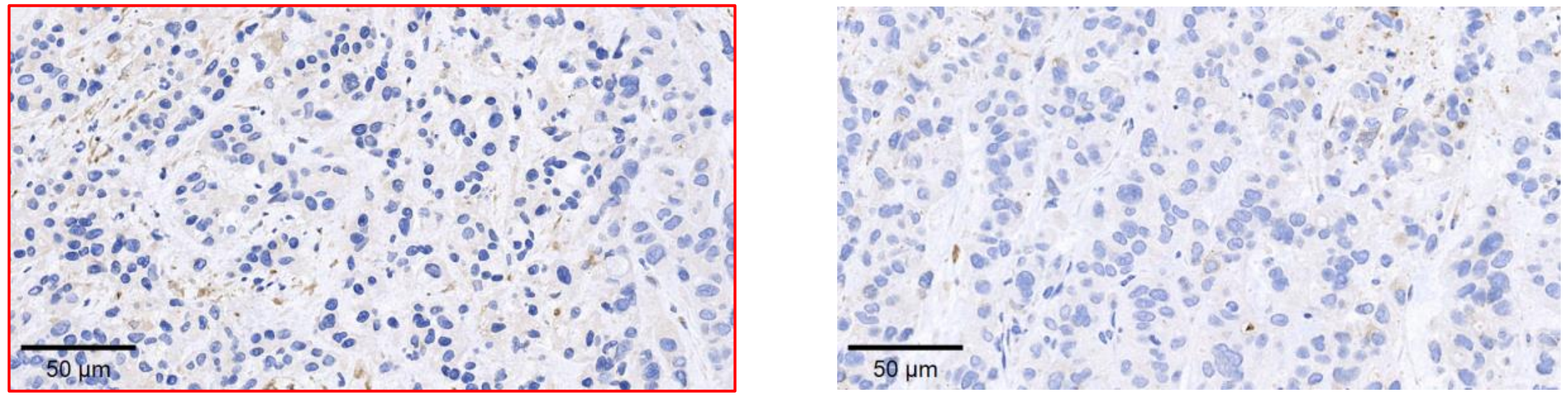

Figure S3A Vector: Snail (three repeats)

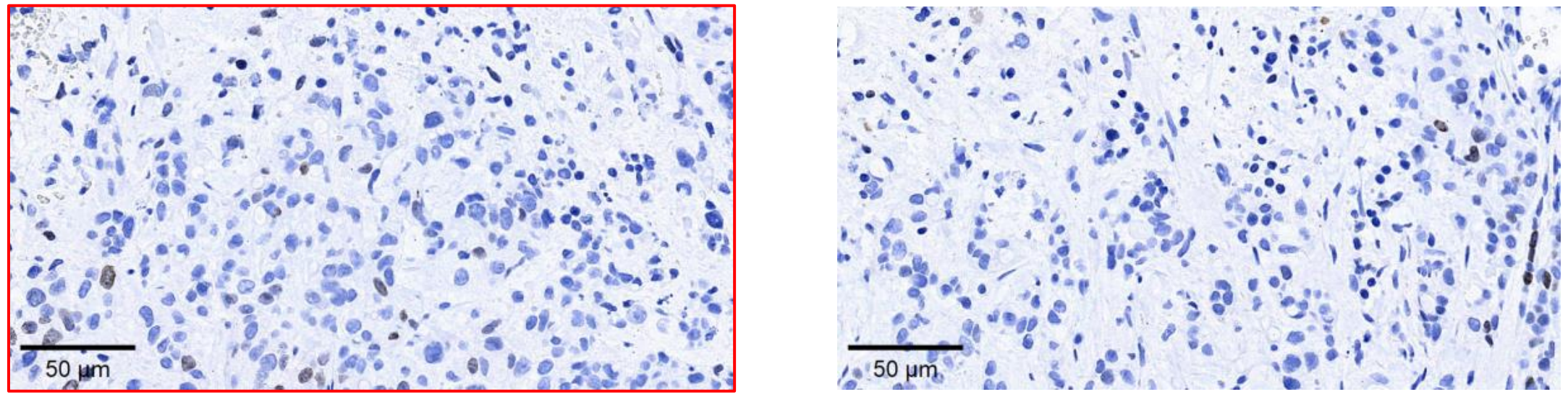

Figure S3A Vector: N-cadherin (three repeats)

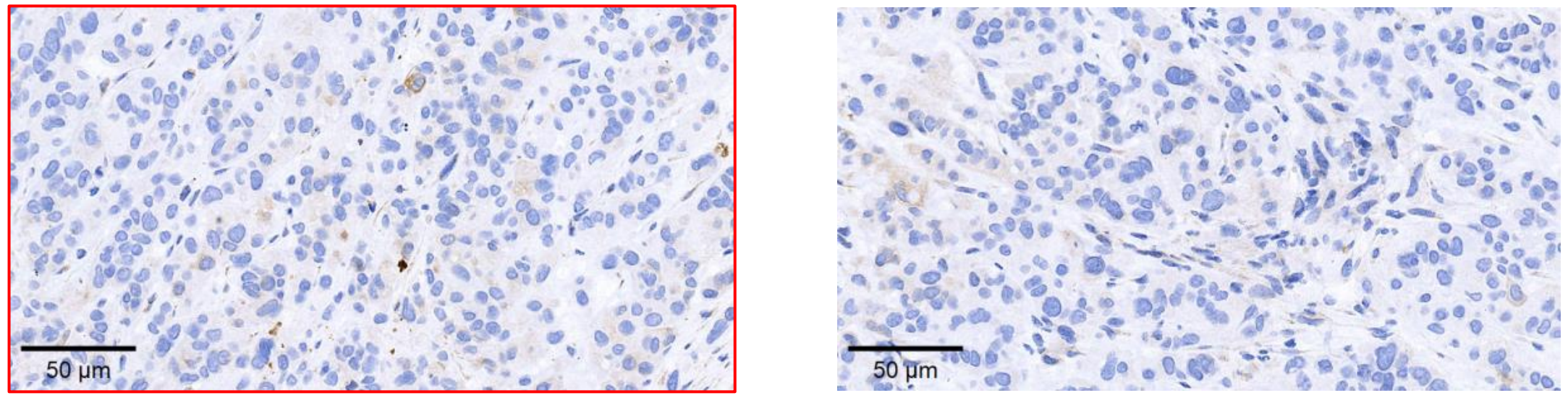

Figure S3A Vector: CD44 (three repeats)

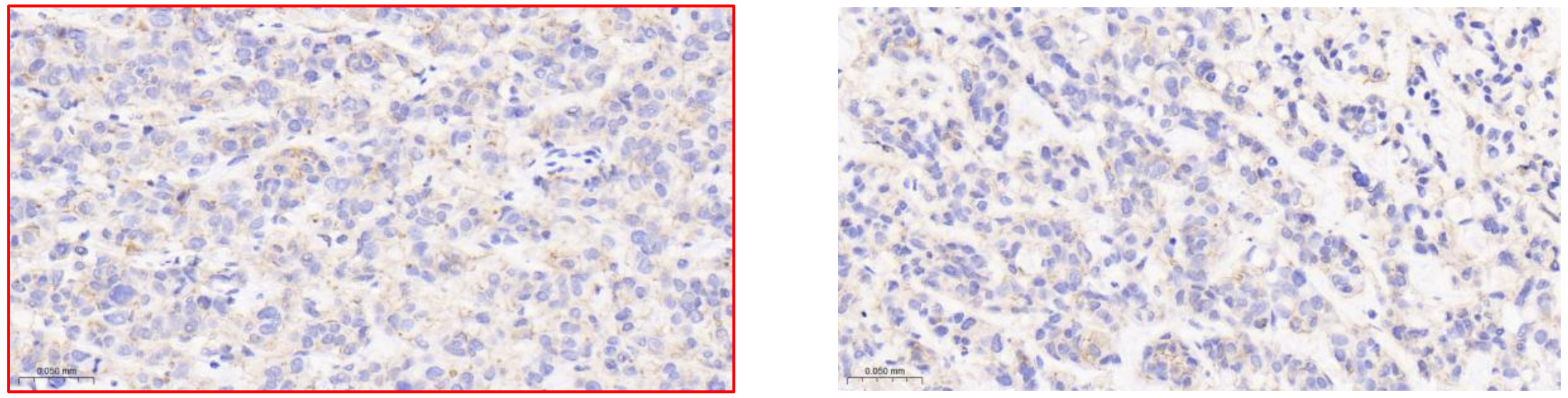

Figure S3A Vector: SOX2 (three repeats)

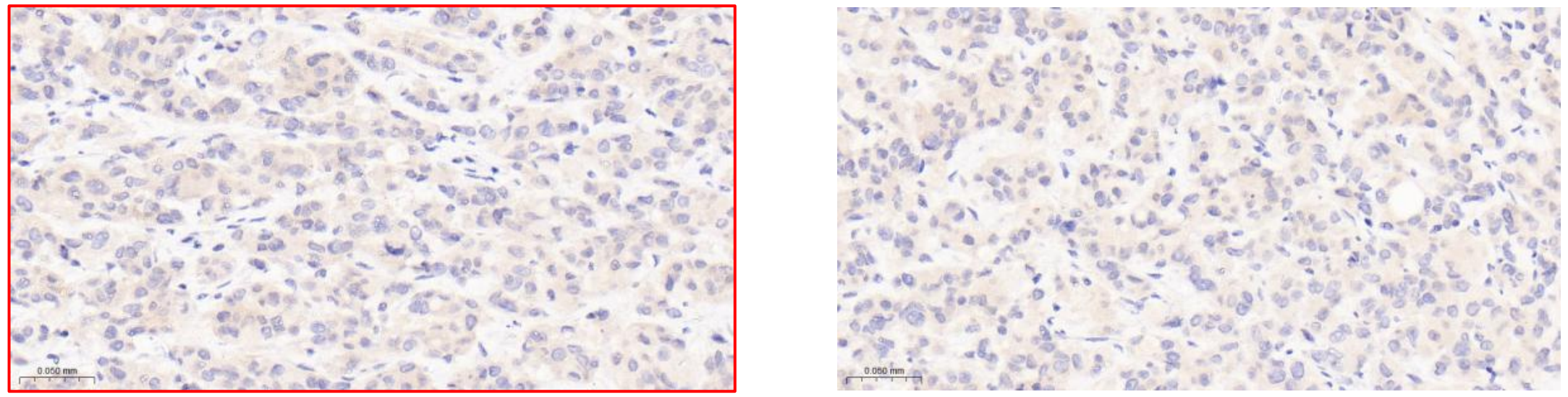

Figure S3A Vector: NANOG (three repeats)

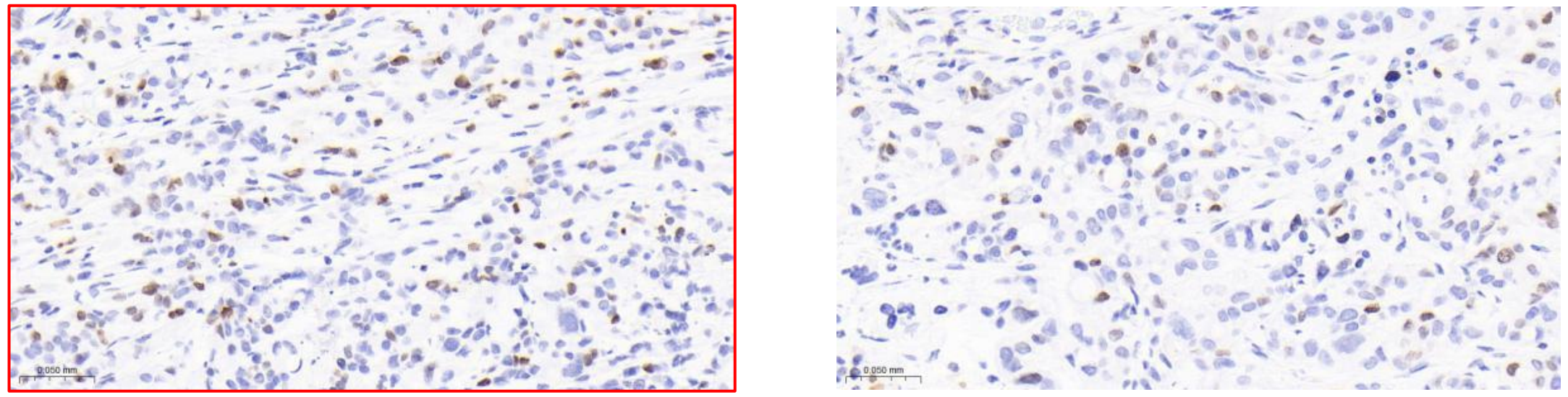

Figure S3A ARNTL2: ARNTL2 (three repeats)

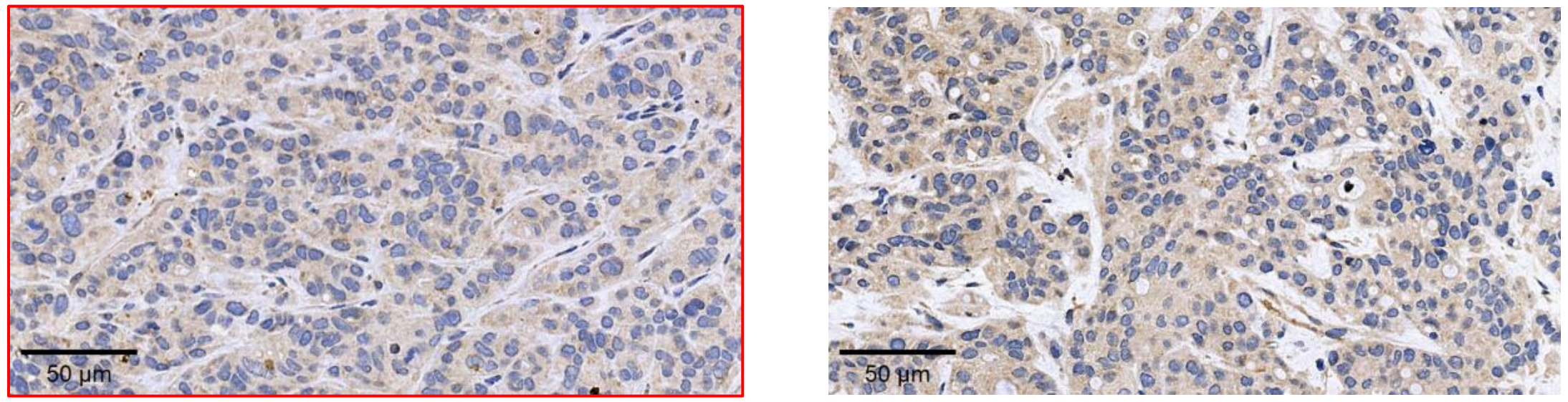

Figure S3A ARNTL2: Vimentin (three repeats)

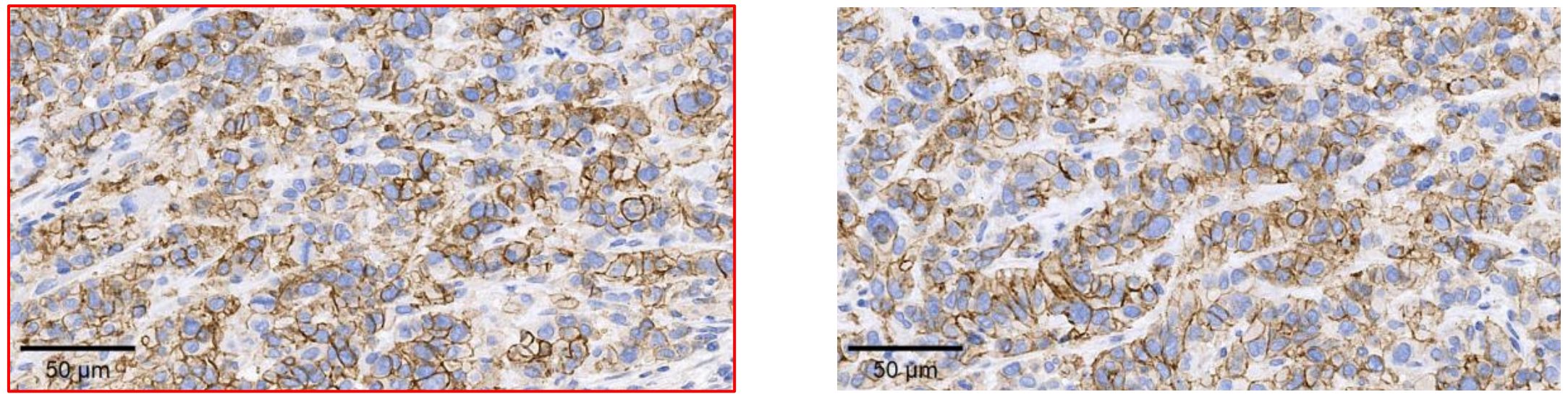

Figure S3A ARNTL2: SERPINE1 (three repeats)

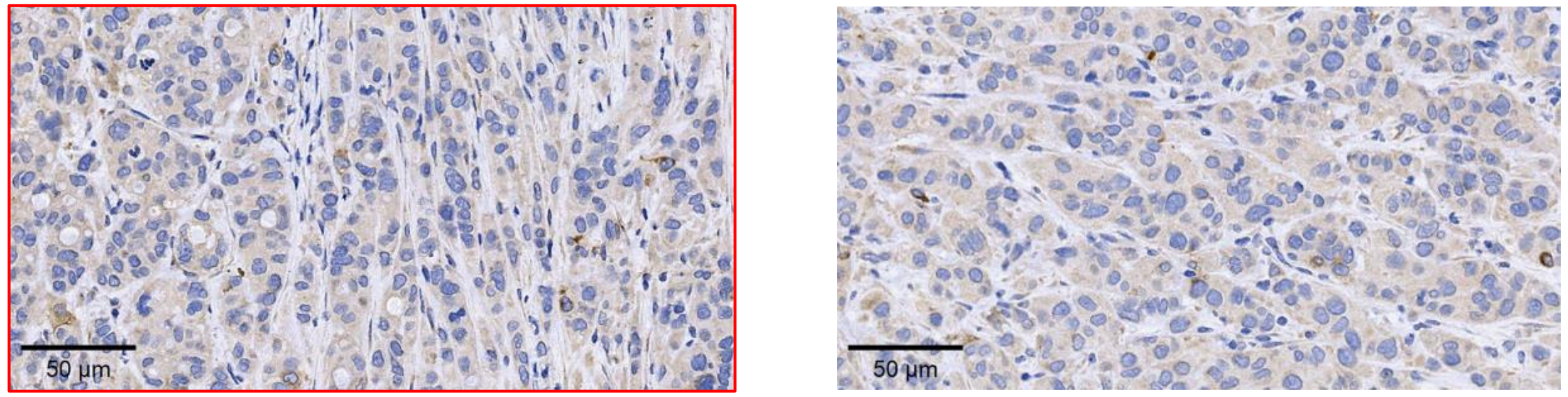

Figure S3A ARNTL2: Snail (three repeats)

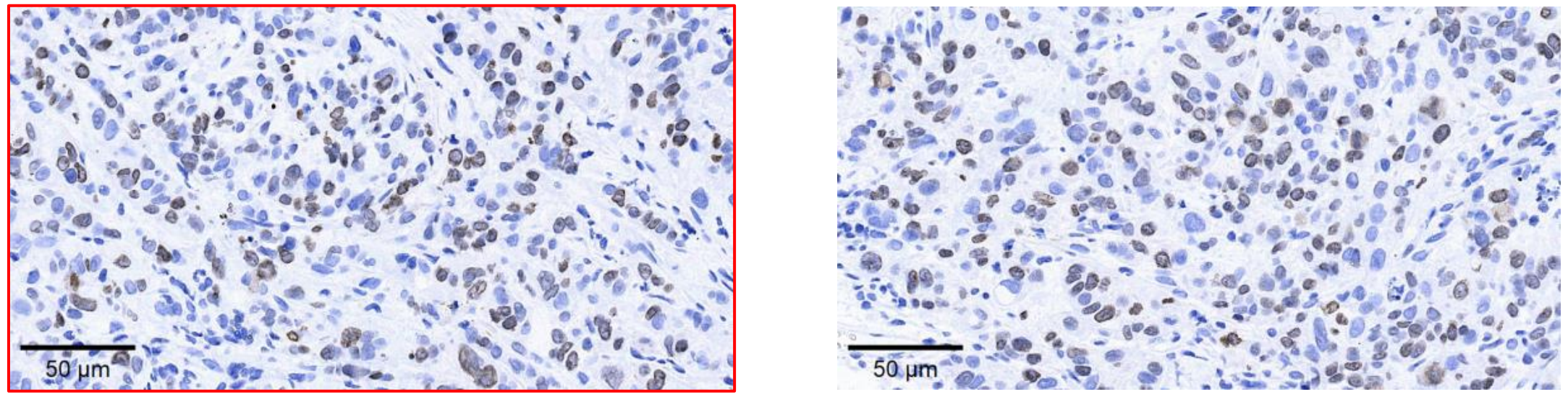

Figure S3A ARNTL2: N-cadherin (three repeats)

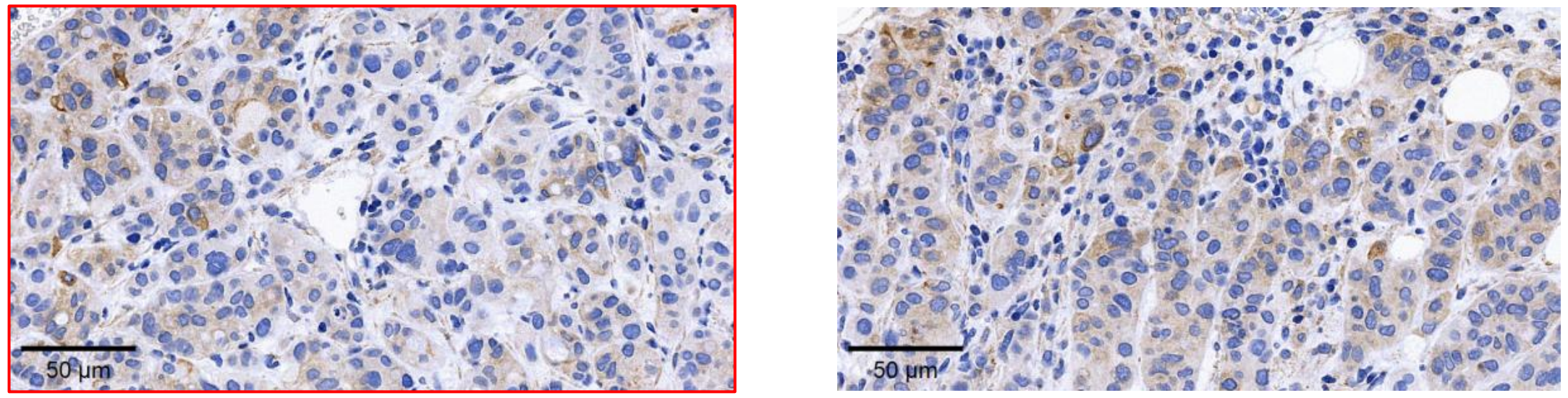

Figure S3A ARNTL2: CD44 (three repeats)

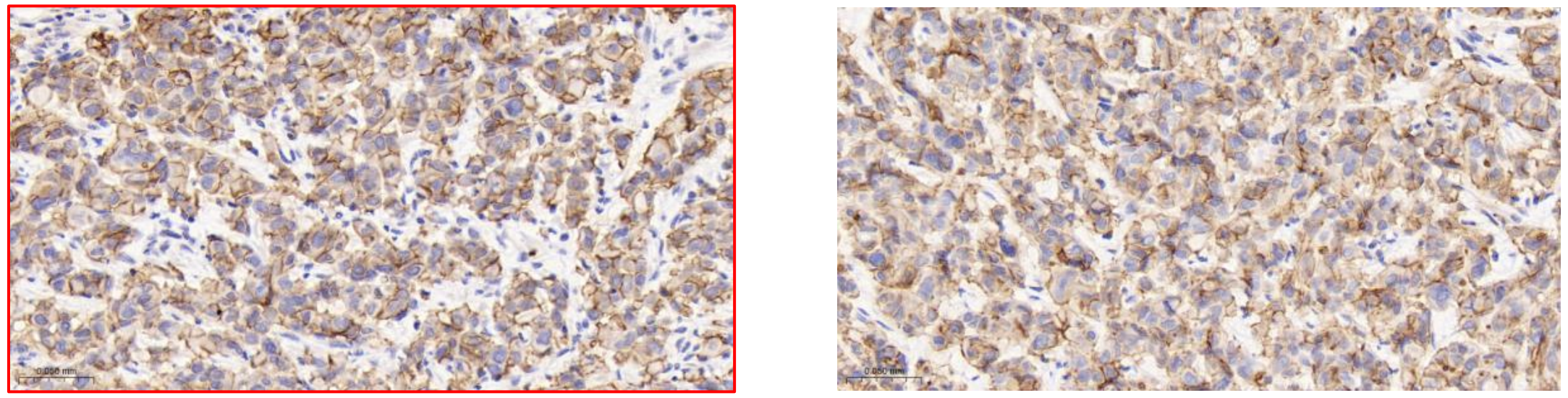

Figure S3A ARNTL2: SOX2 (three repeats)

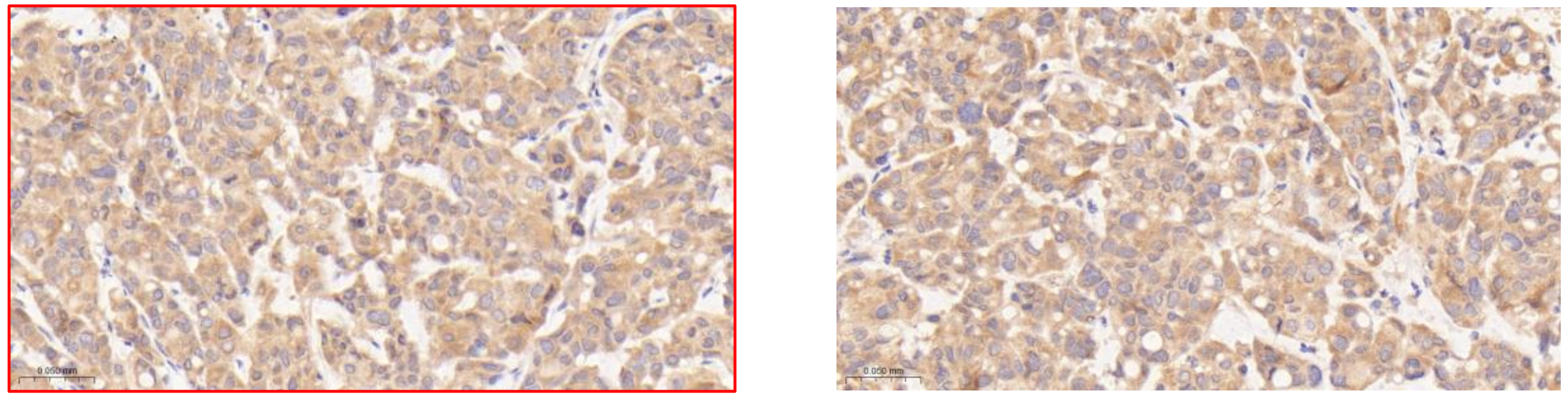

Figure S3A ARNTL2: NANOG (three repeats)

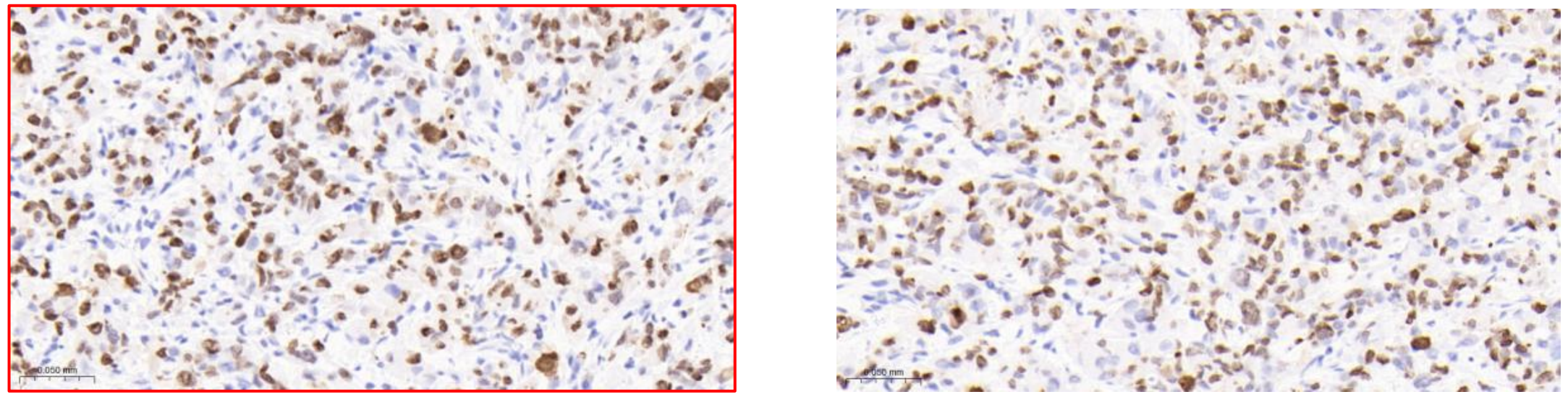

Figure S3B shNC: ARNTL2 (three repeats)

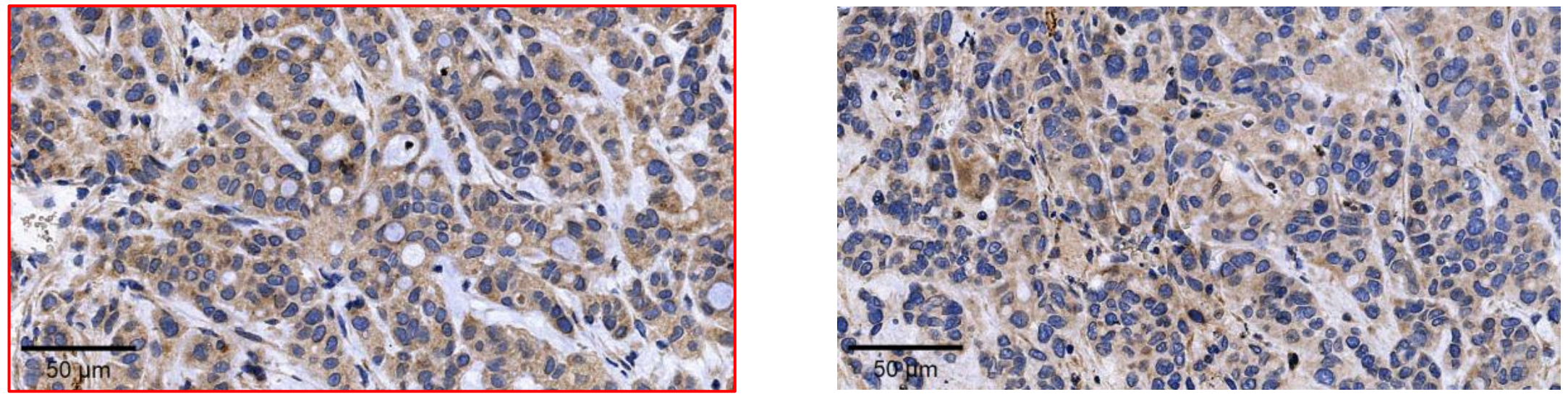

Figure S3B shNC: SERPINE1 (three repeats)

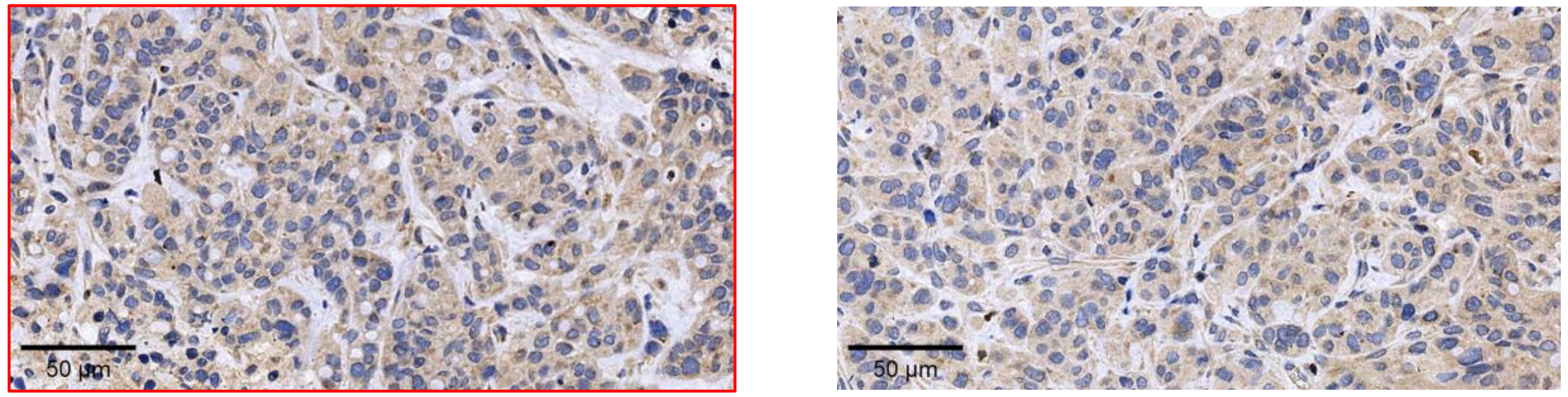

Figure S3B shNC: N-cadherin (three repeats)

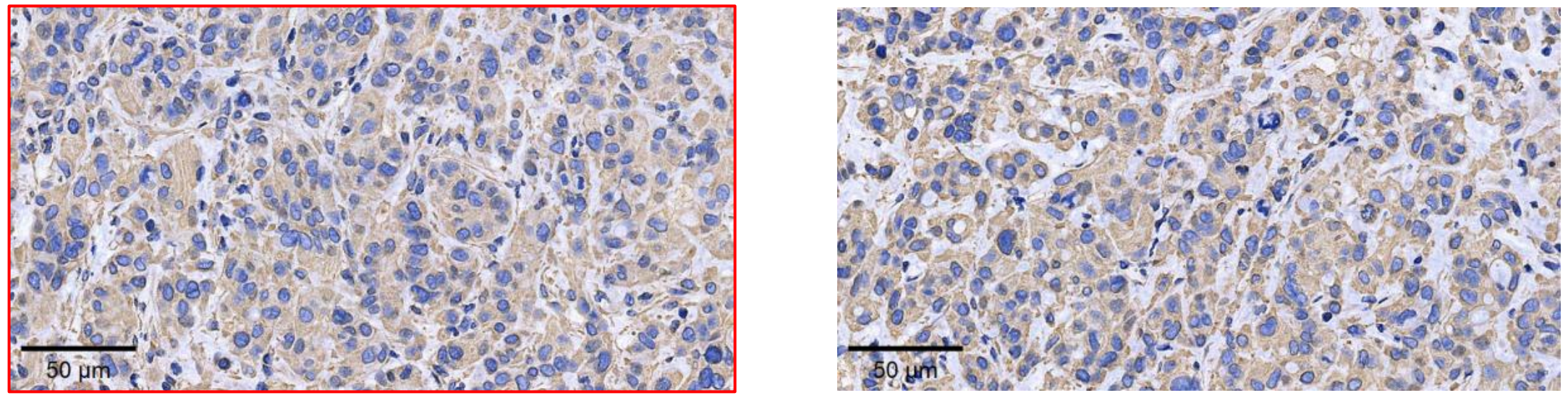

Figure S3B shNC: Vimentin (three repeats)

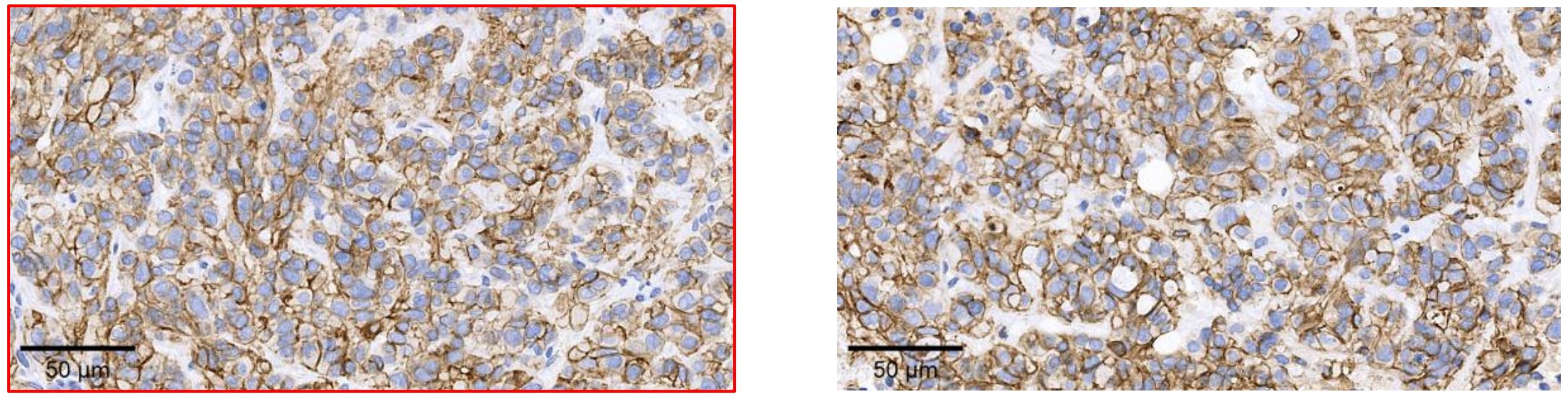

Figure S3B shNC: Snail (three repeats)

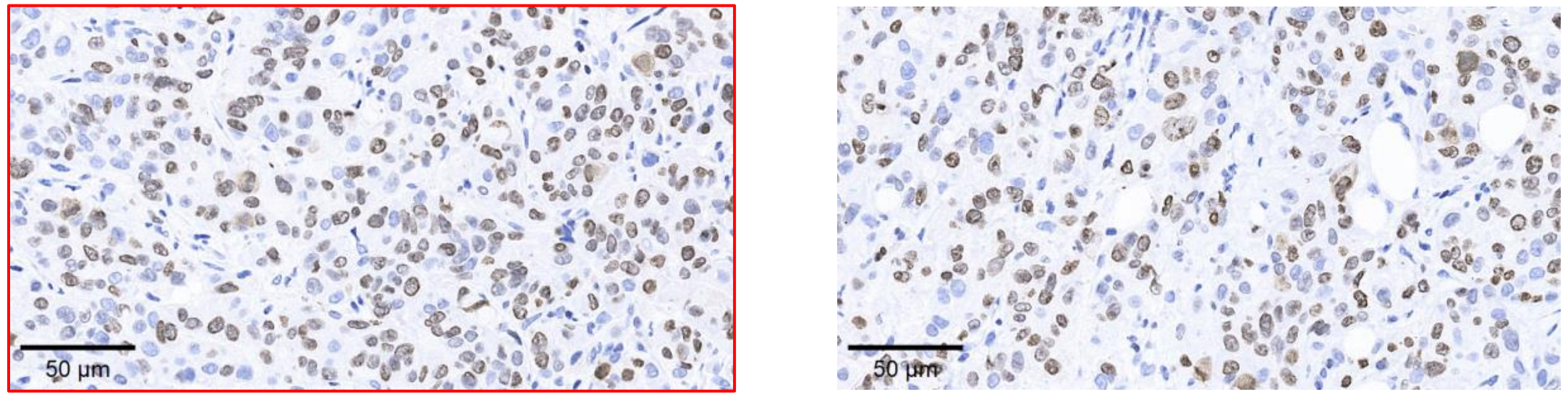

Figure S3B shNC: CD44 (three repeats)

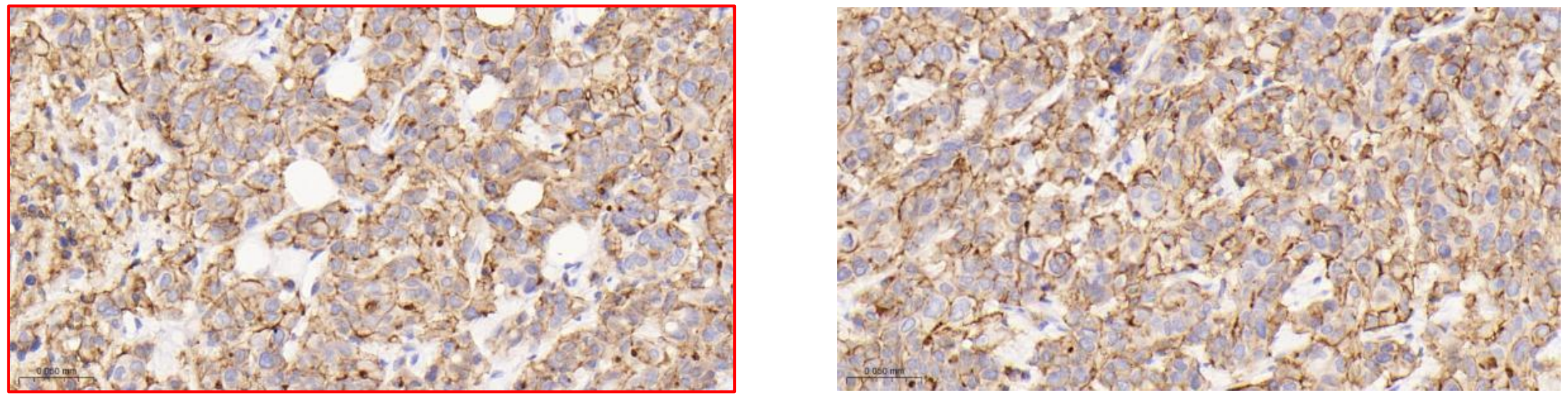

Figure S3B shNC: SOX2 (three repeats)

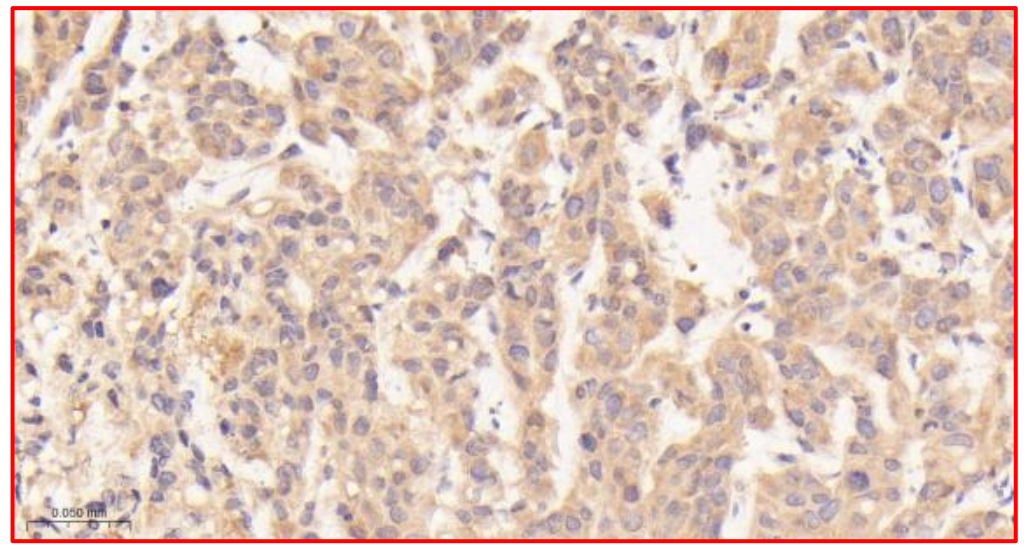

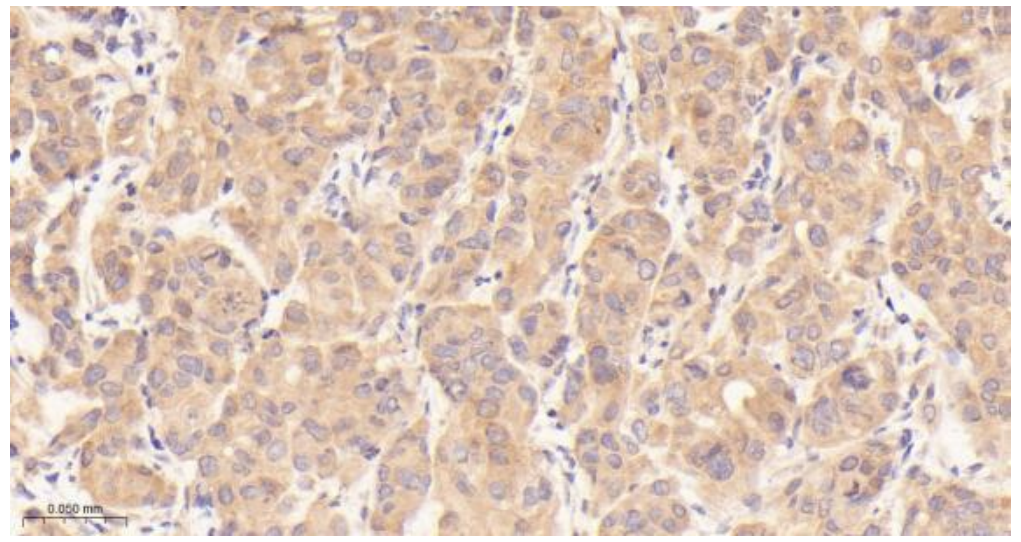

Figure S3B shNC: NANOG (three repeats)

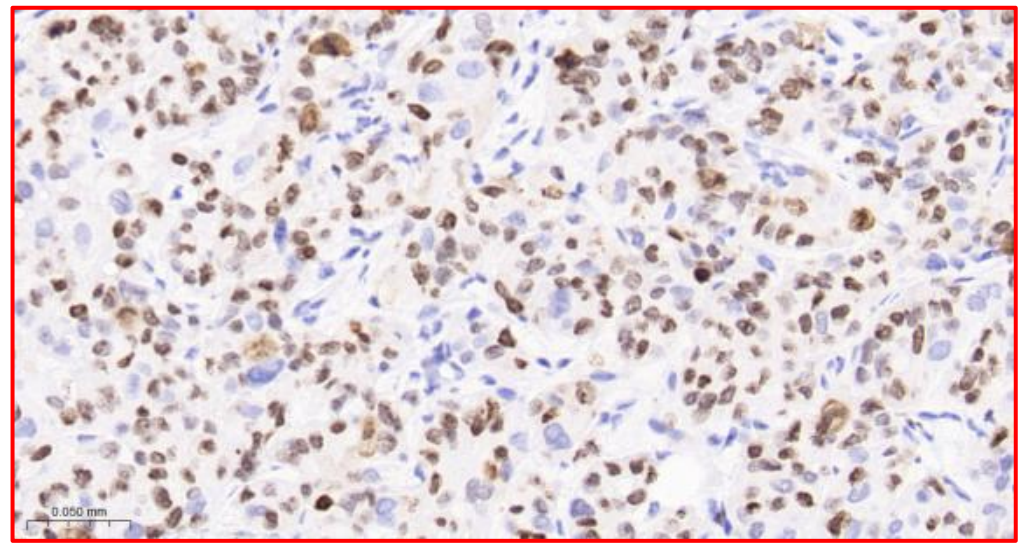

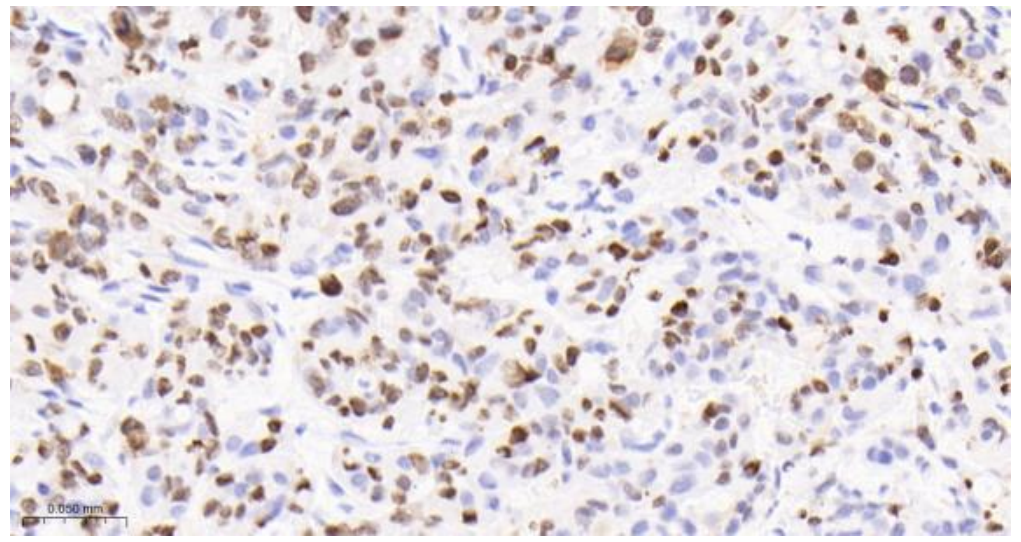

Figure S3B shARNTL2: ARNTL2 (three repeats)

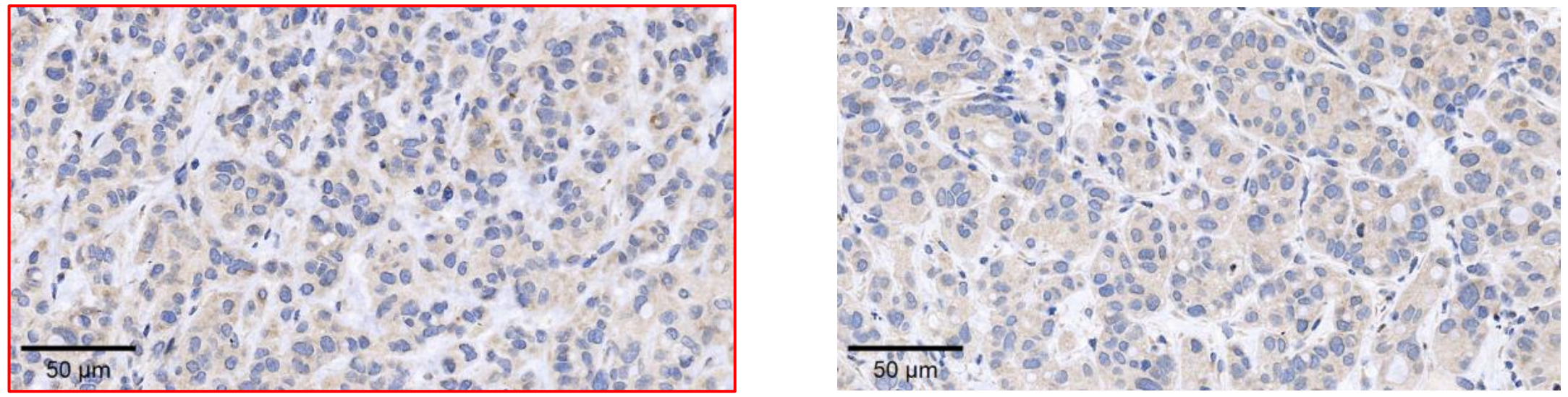

Figure S3B shARNTL2: Vimentin (three repeats)

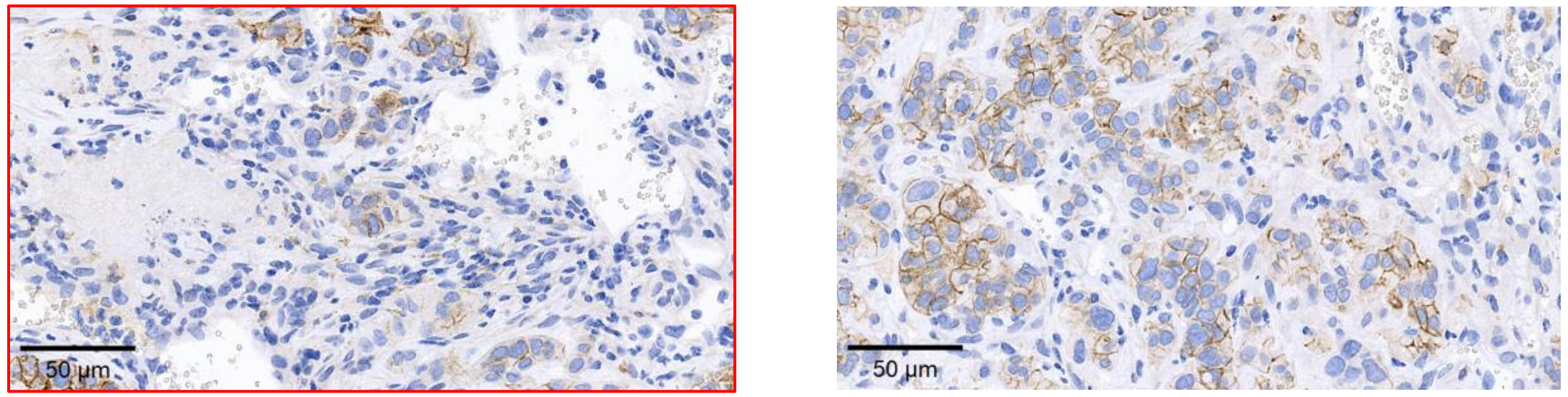

Figure S3B shARNTL2: SERPINE1 (three repeats)

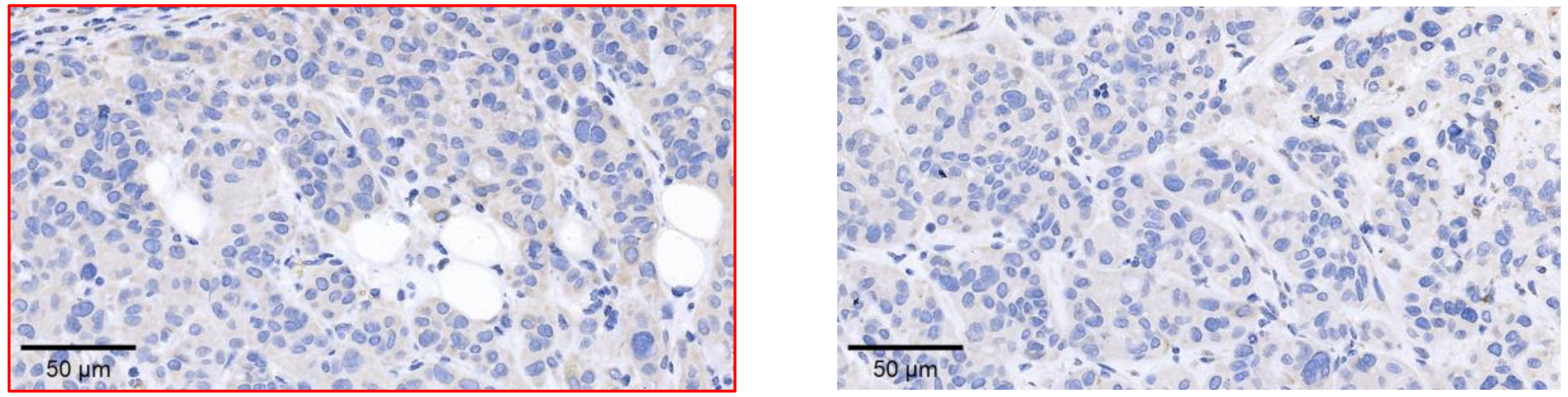

Figure S3B shARNTL2: Snail (three repeats)

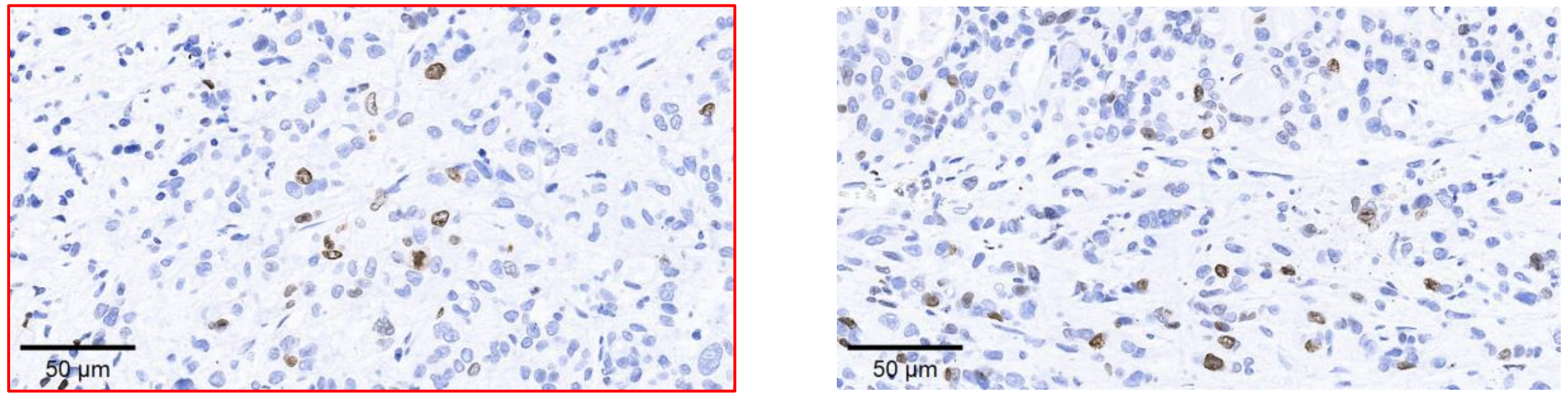

Figure S3B shARNTL2: N-cadherin (three repeats)

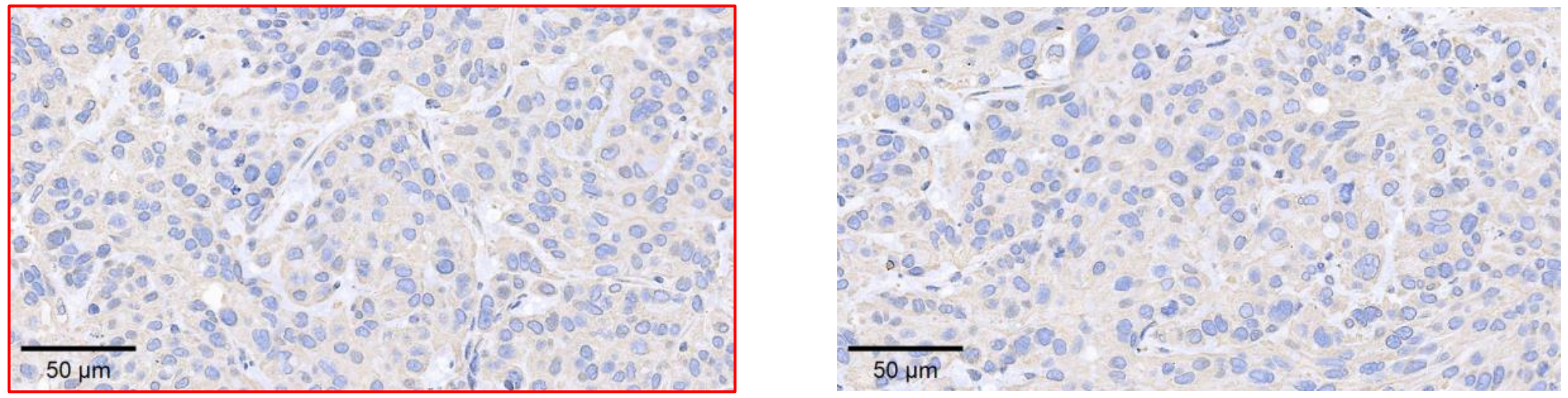

Figure S3B shARNTL2: CD44 (three repeats)

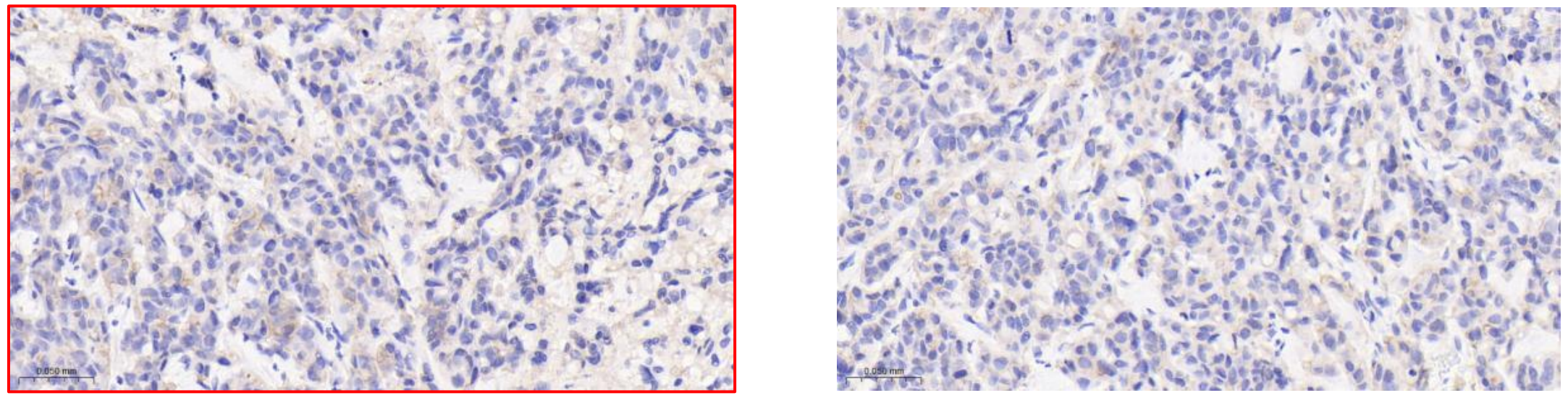

Figure S3B shARNTL2: SOX2 (three repeats)

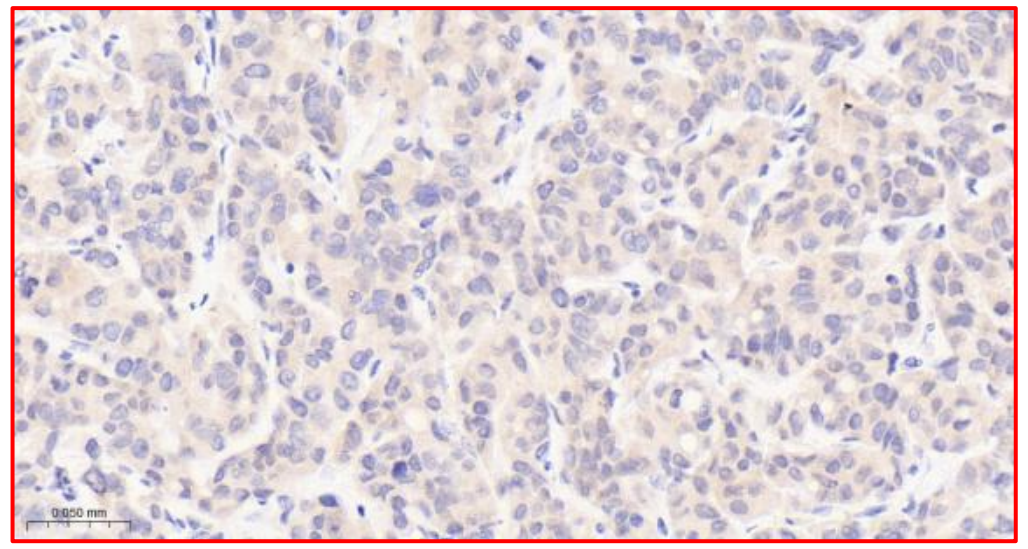

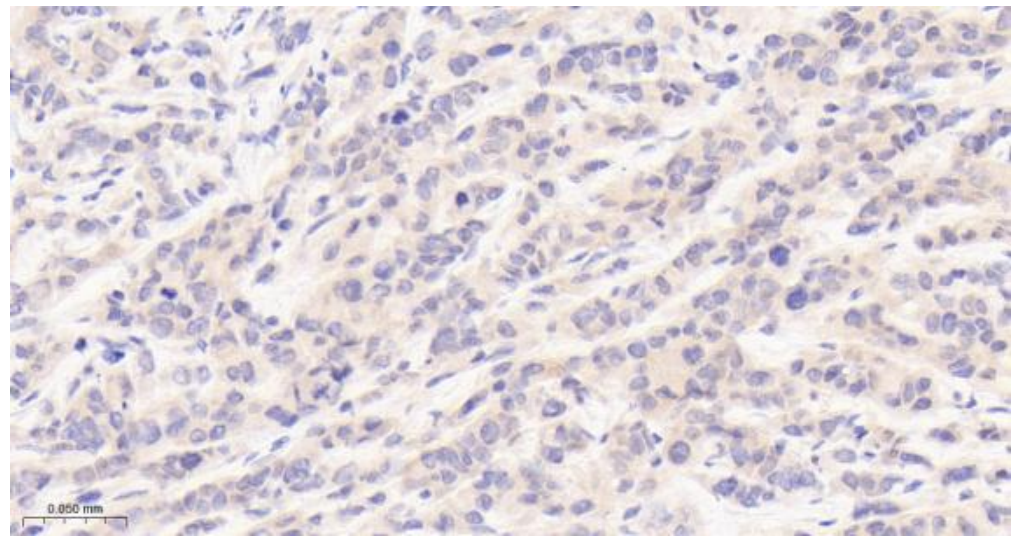

Figure S3B shARNTL2: NANOG (three repeats)

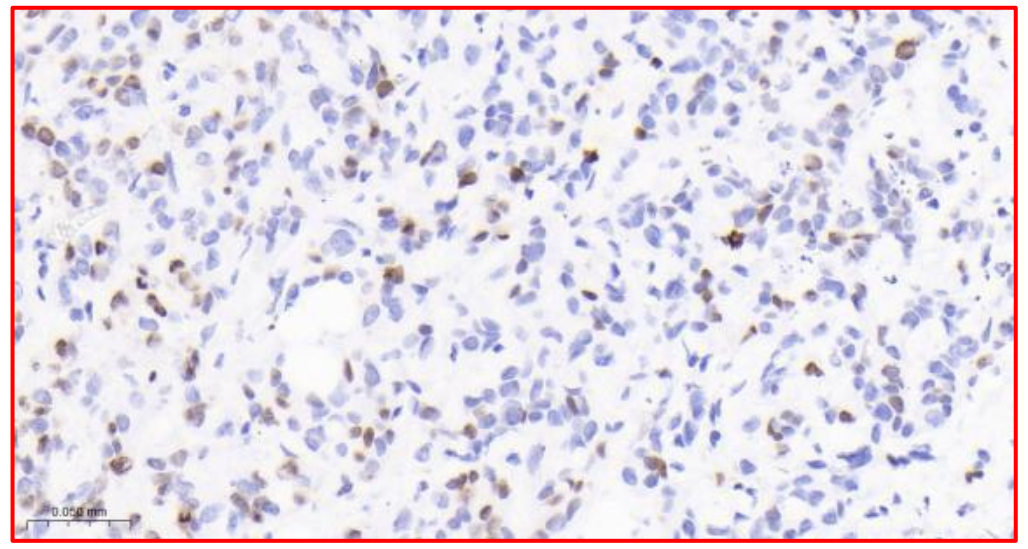

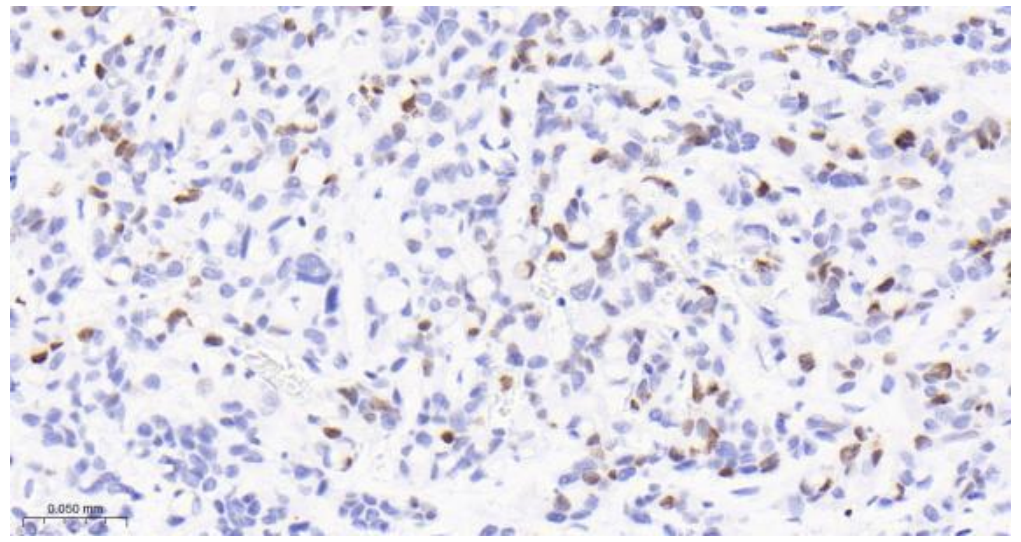

Figure S4L: 0h shNC (three repeats)

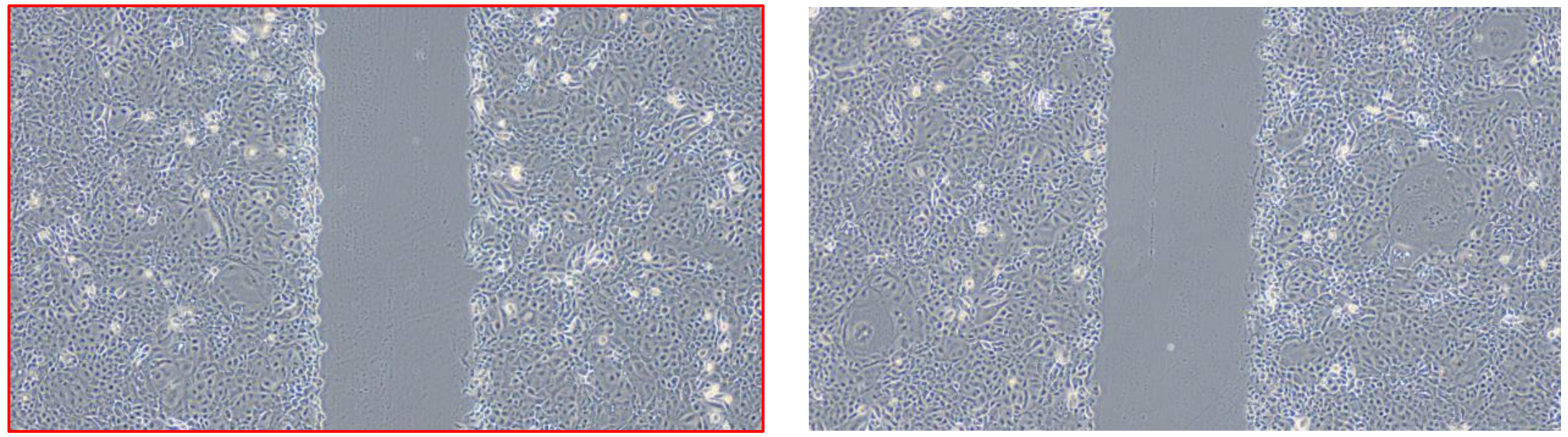

Figure S4L: 24h shNC (three repeats)

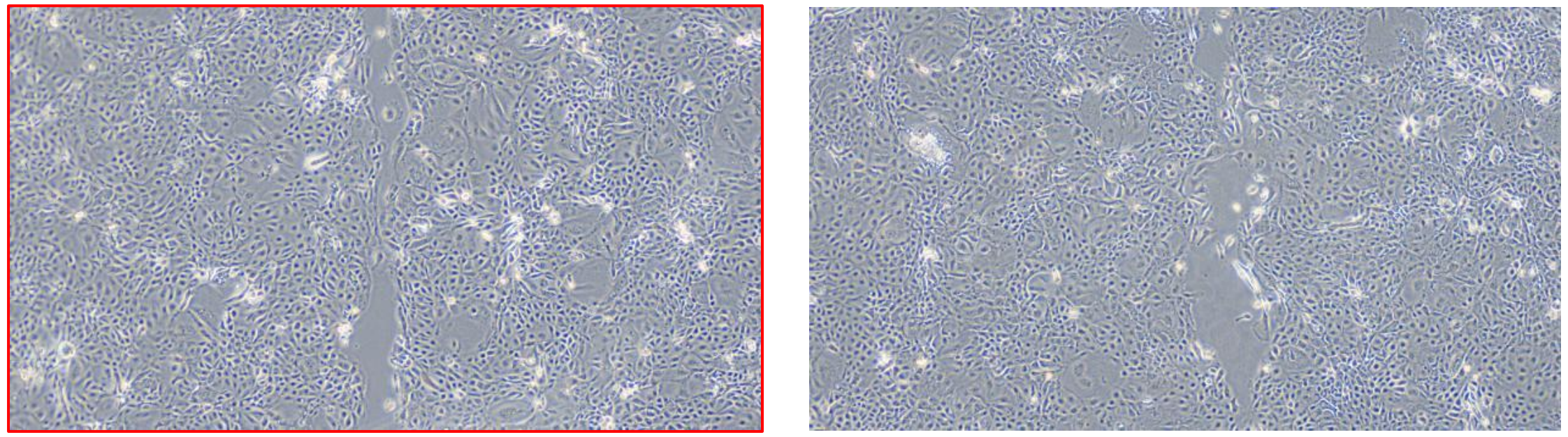

Figure S4L: 0h shSERPINE1-1 (three repeats)

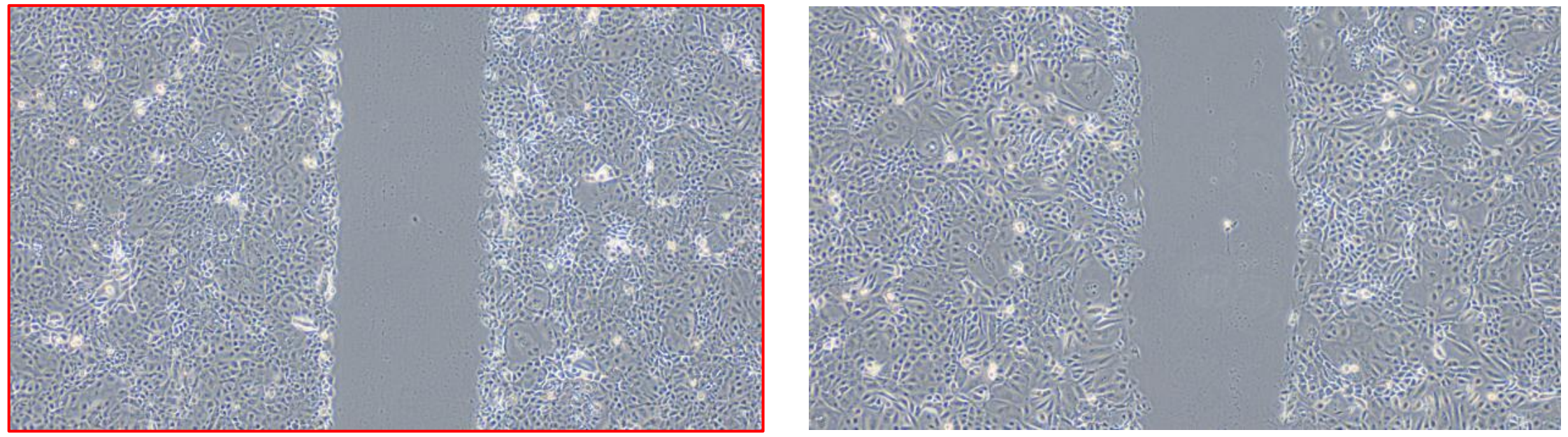

Figure S4L: 24h shSERPINE1-1 (three repeats)

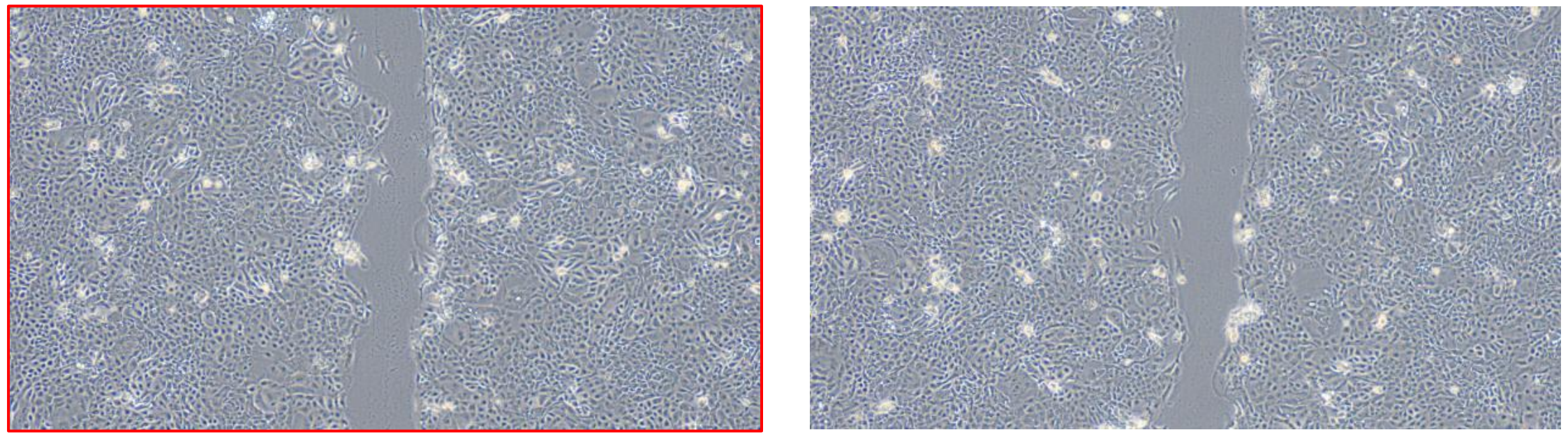

Figure S4L: 0h shSERPINE1-2 (three repeats)

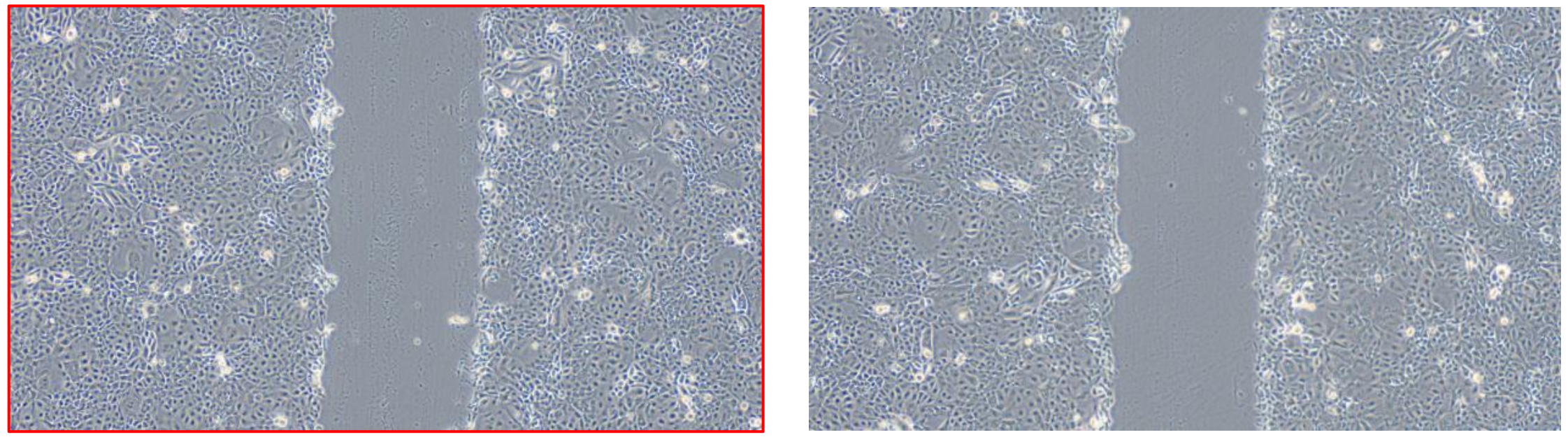

Figure S4L: 24h shSERPINE1-2 (three repeats)

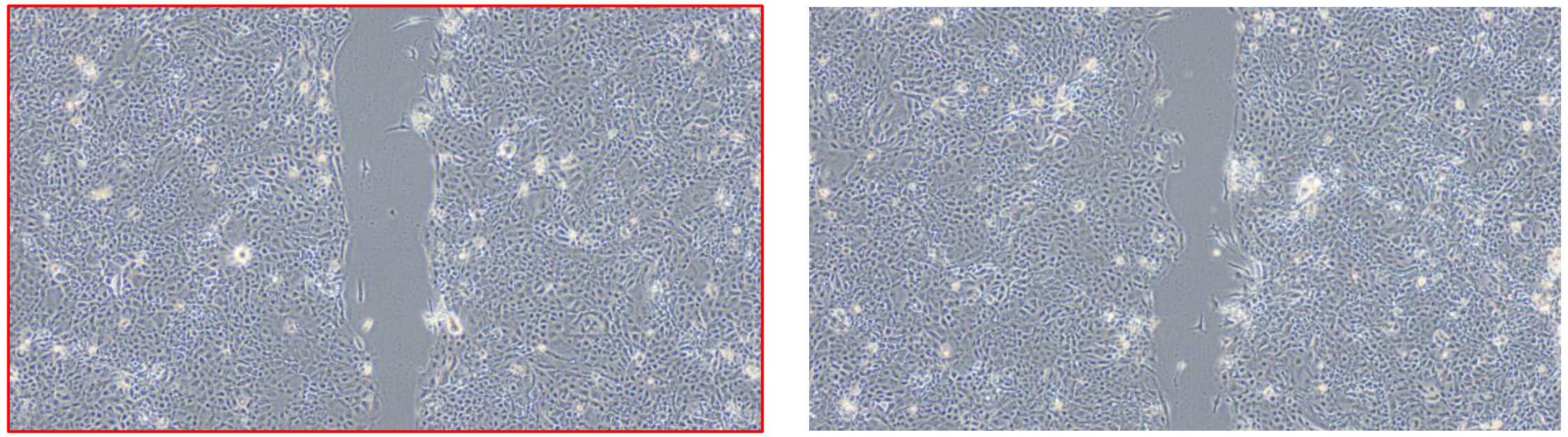

Figure S4M: shNC (three repeats)

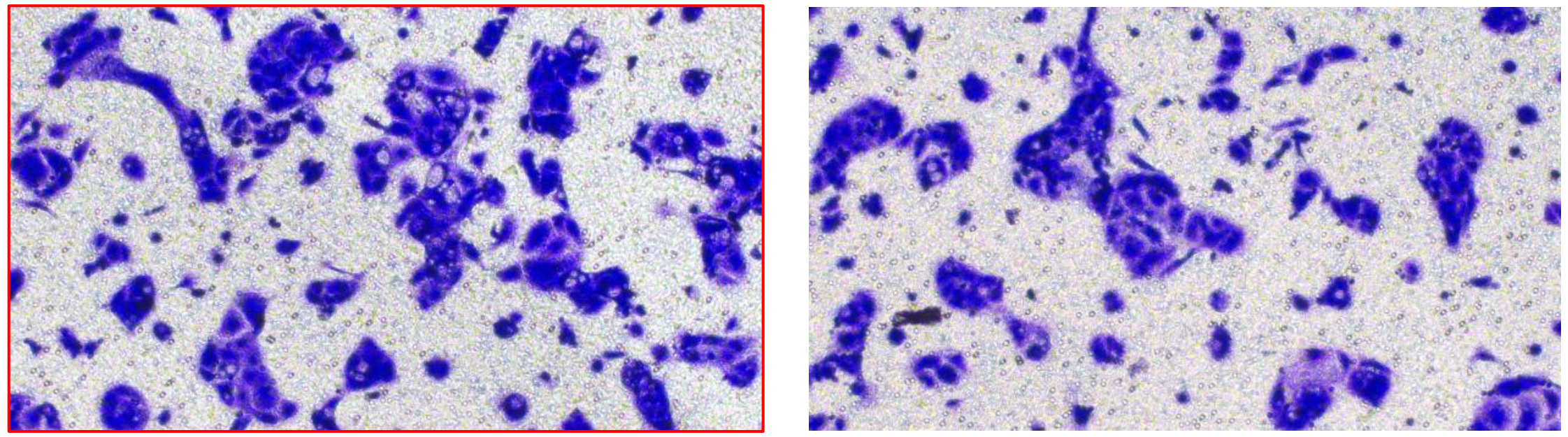

Figure S4M: shSERPINE1-1 (three repeats)

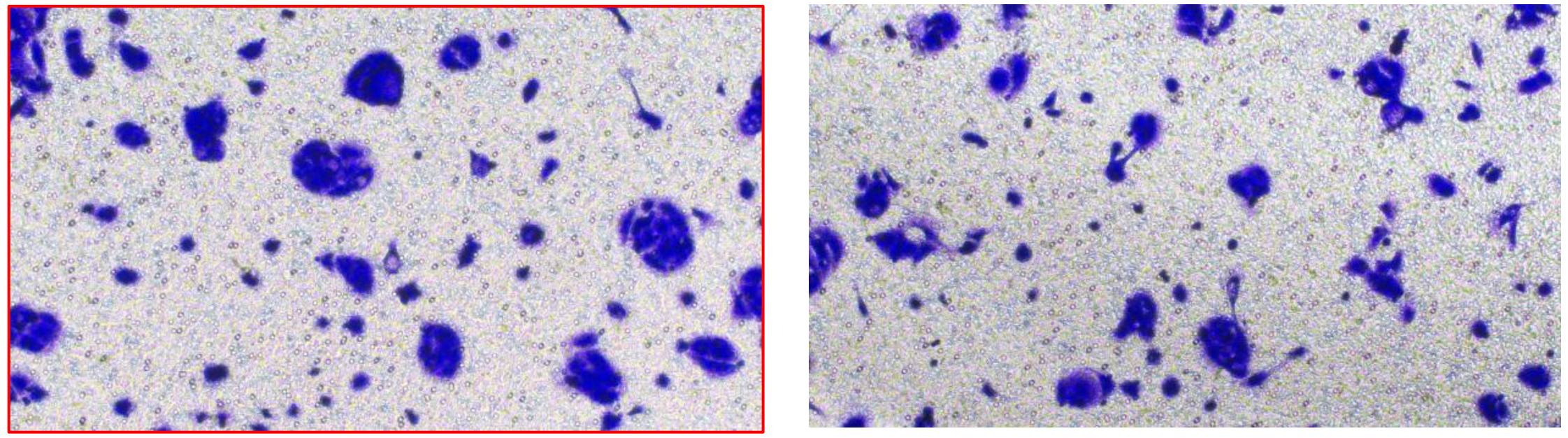

Figure S4M: shSERPINE1-2

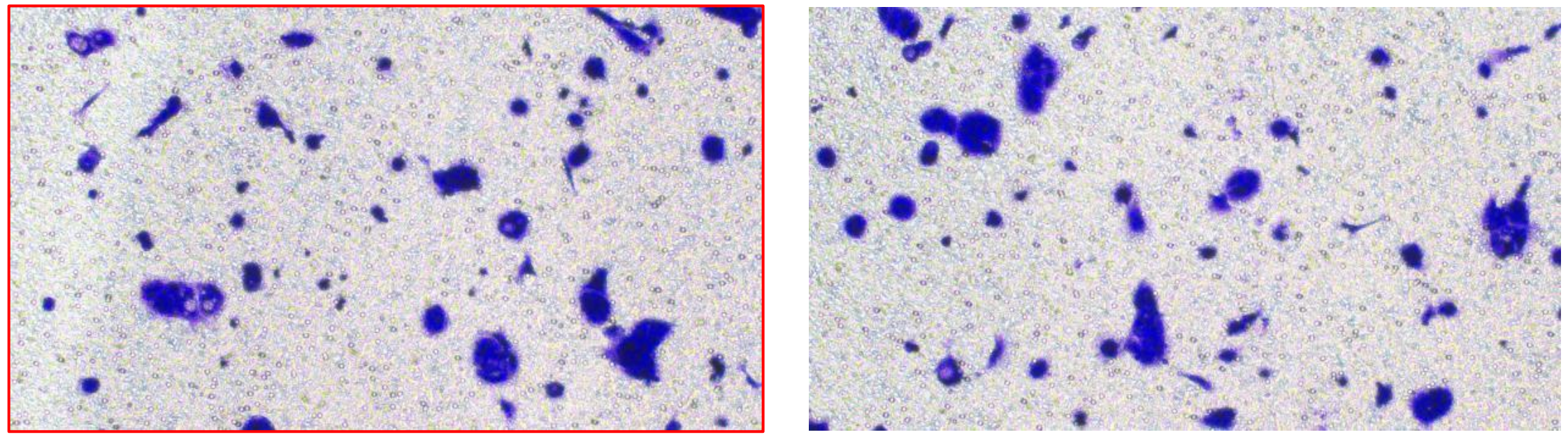

Figure S4N: shNC (three repeats)

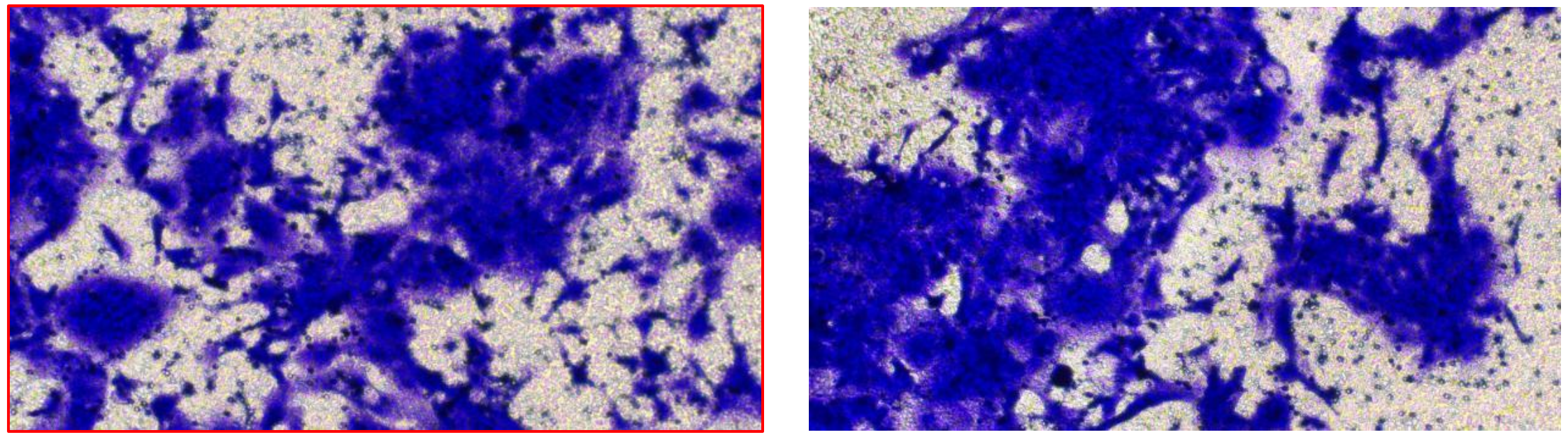

Figure S4N: shSERPINE1-1 (three repeats)

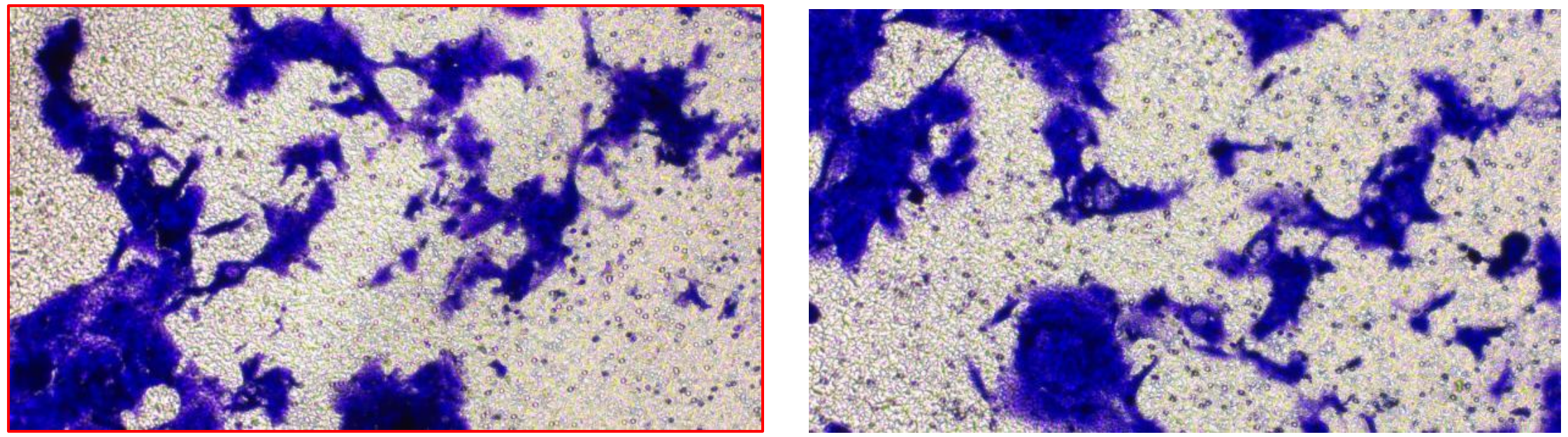

Figure S4N: shSERPINE1-2 (three repeats)

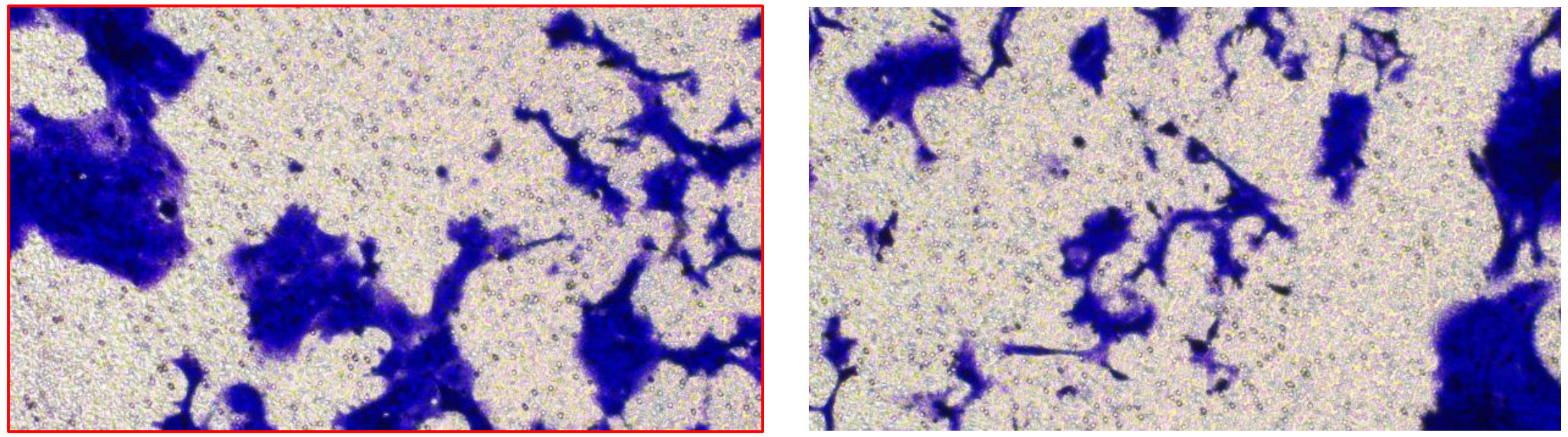

Figure S8A BxPC-3: Vector (three repeats)

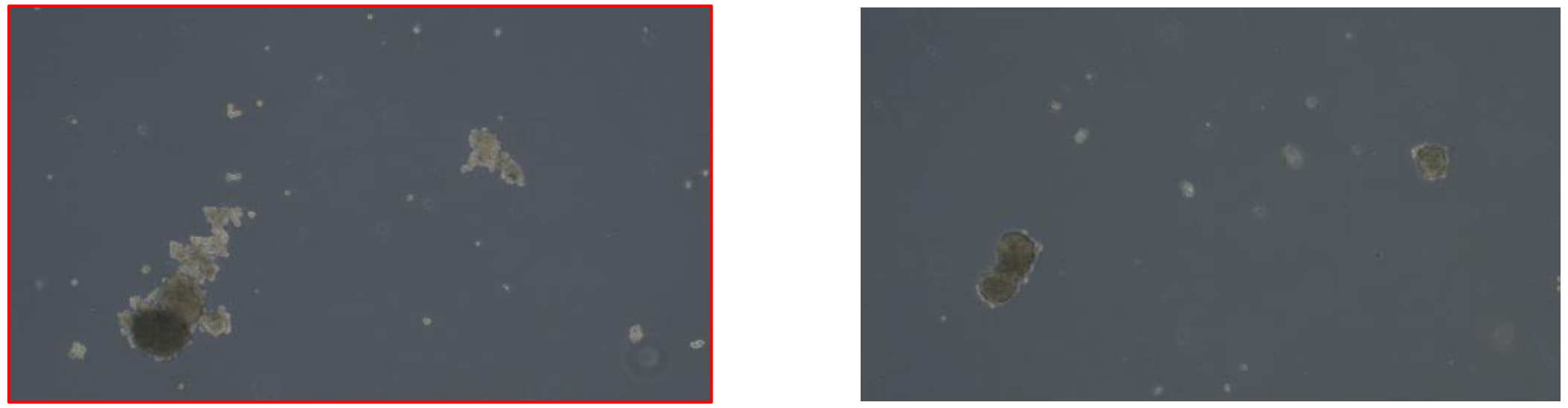

Figure S8A BxPC-3: SERPINE1 (three repeats)

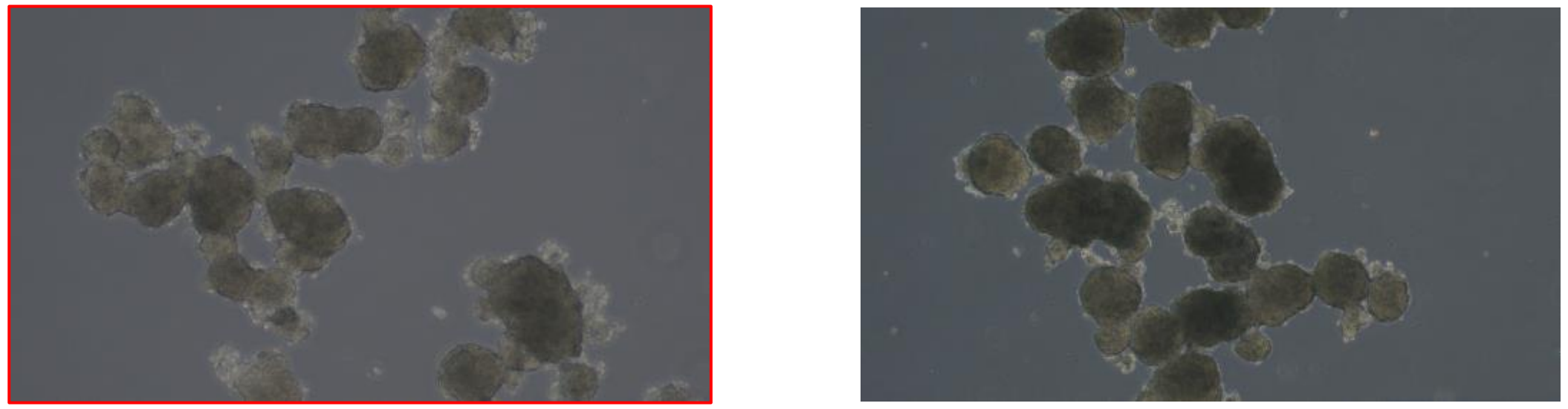

Figure S8A BxPC-3: ARNTL2 (three repeats)

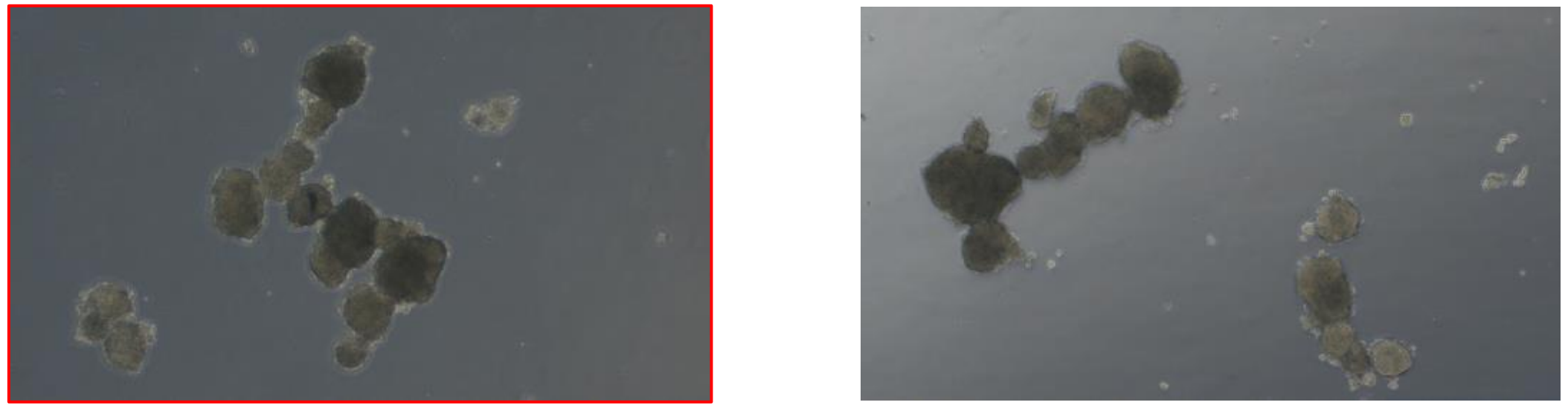

Figure S8A BxPC-3: ARNTL2+shSERPINE1 (three repeats)

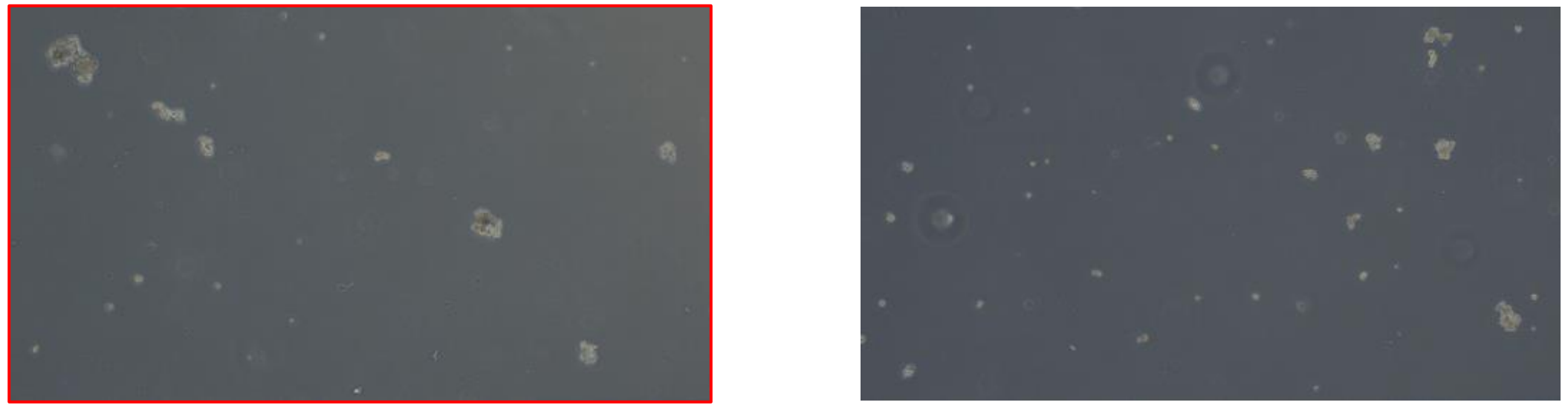

Figure S8A PANC-1: shNC (three repeats)

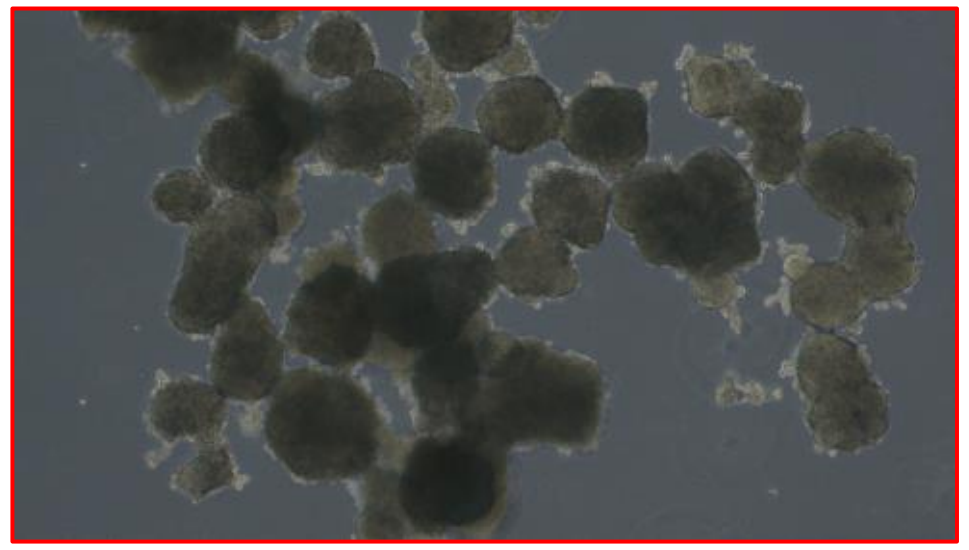
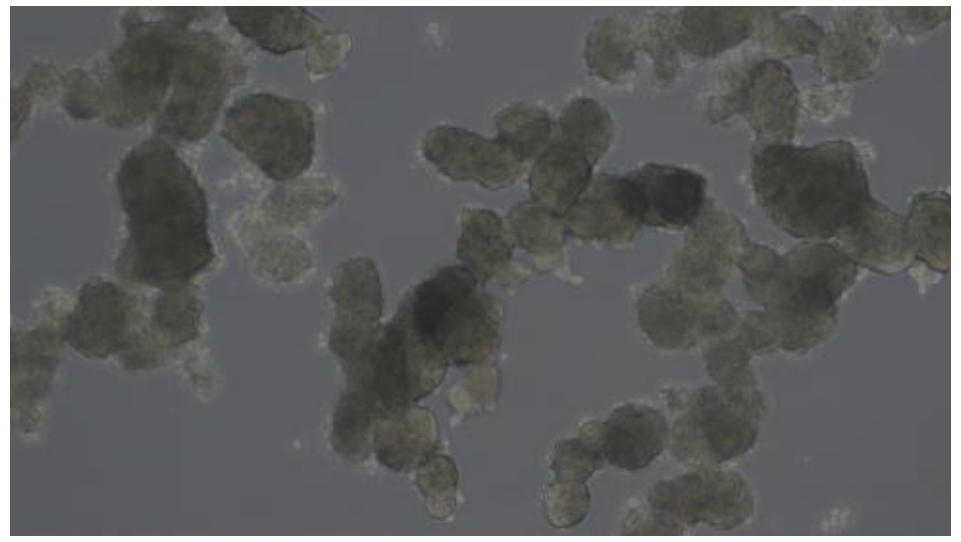

Figure S8A PANC-1: shSERPINE1 (three repeats)

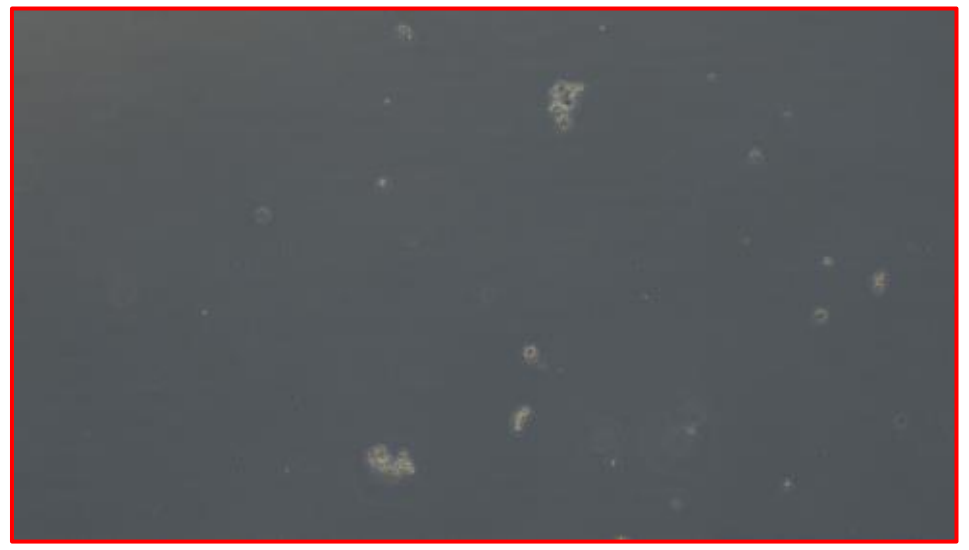
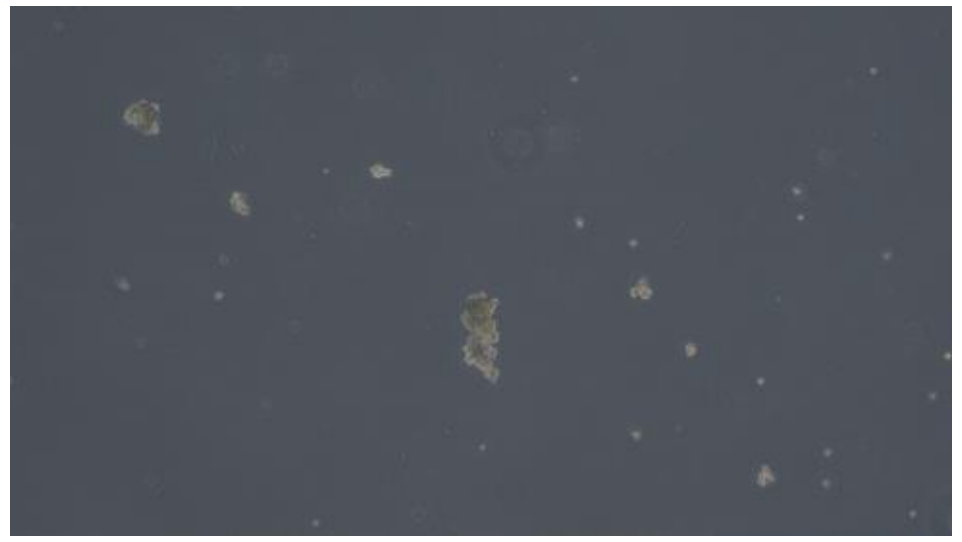

Figure S8A PANC-1: shARNTL2 (three repeats)

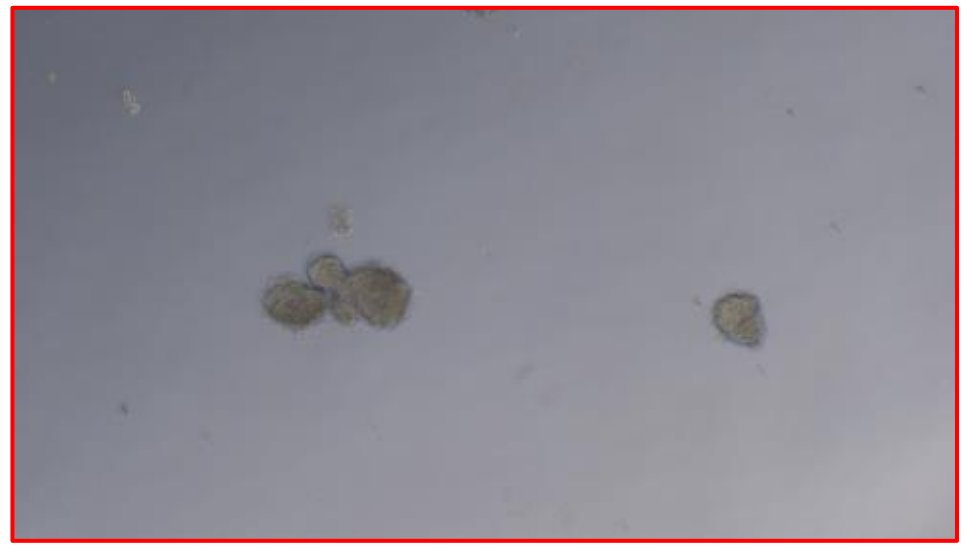
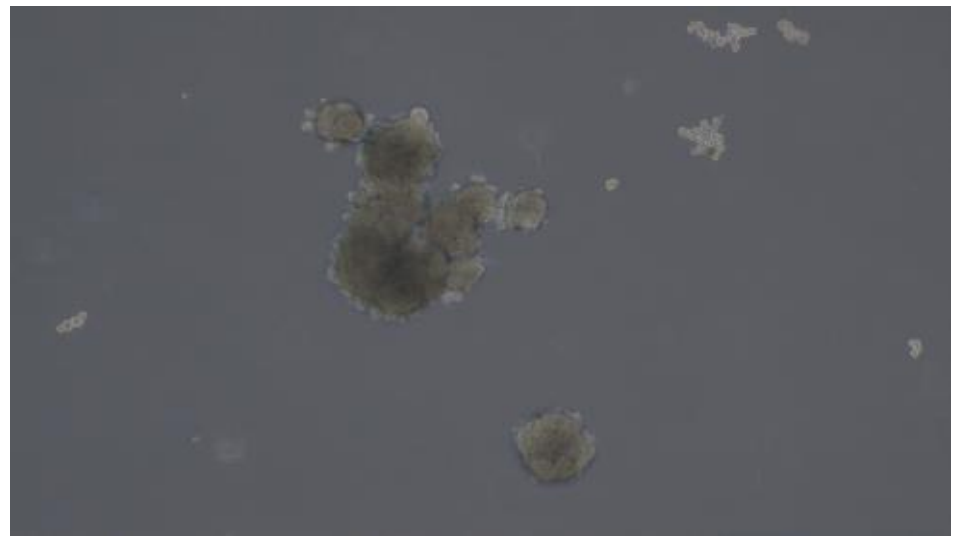

Figure S8A PANC-1: shARNTL2+SERPINE1 (three repeats)

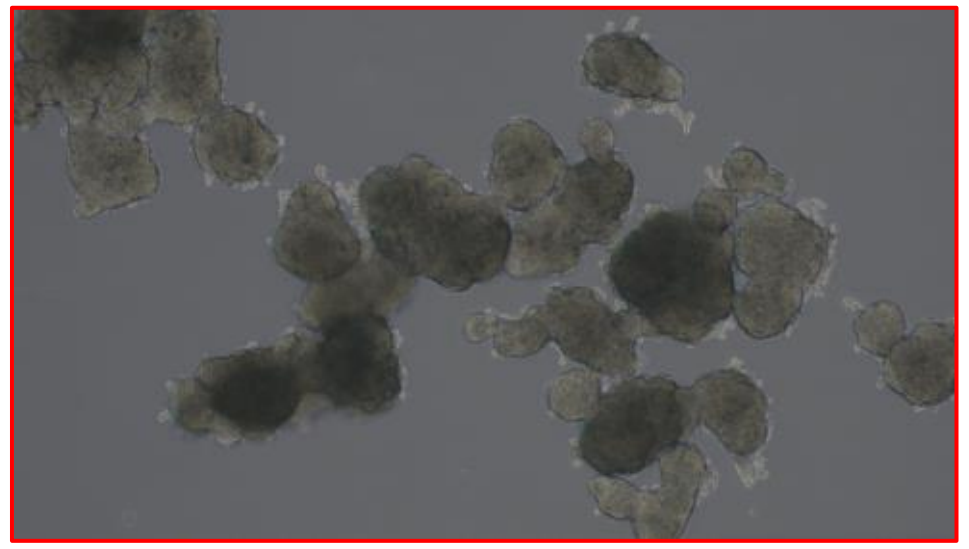
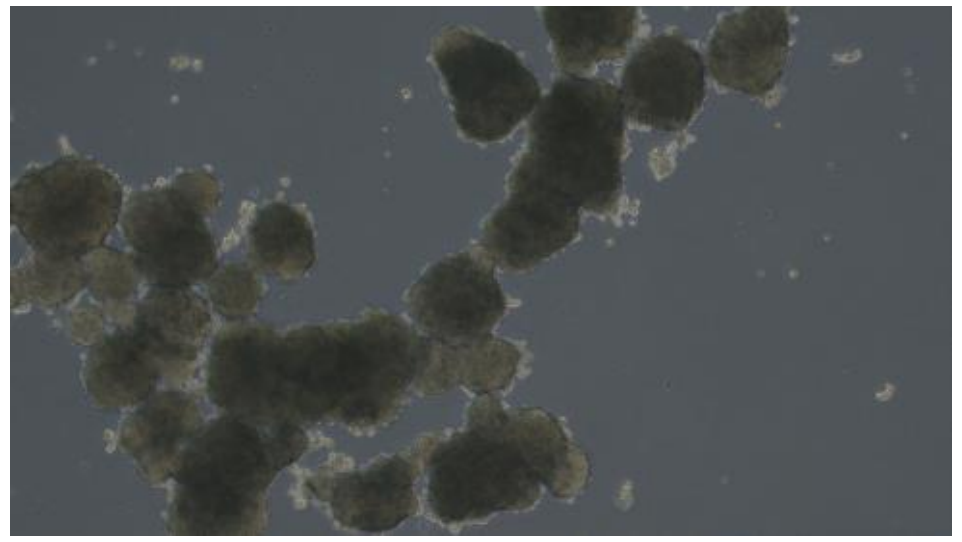

Figure S8A SW1990: shNC (three repeats)

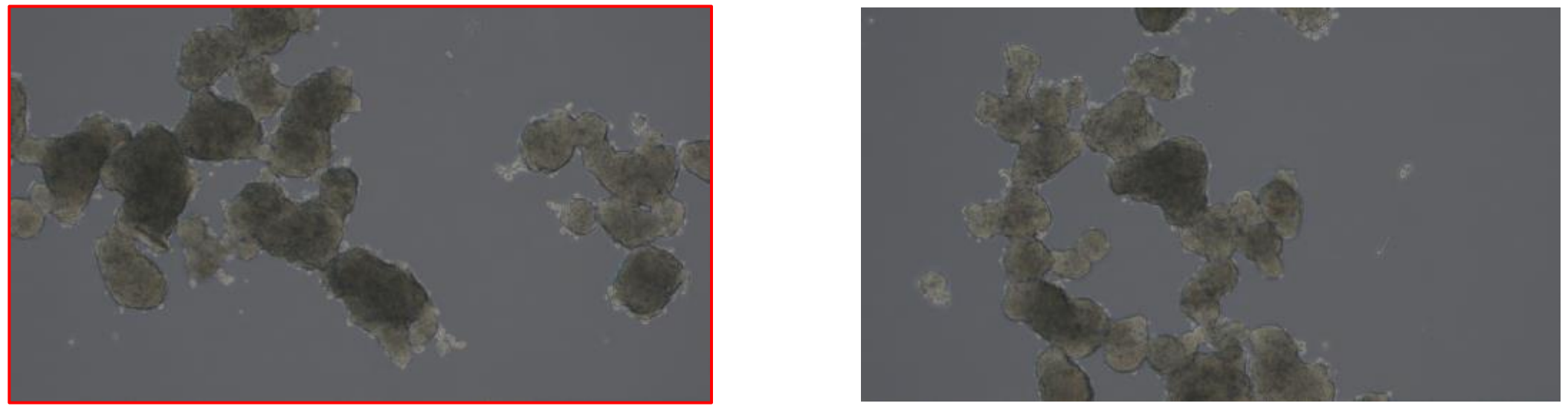

Figure S8A SW1990: shSERPINE1 (three repeats)

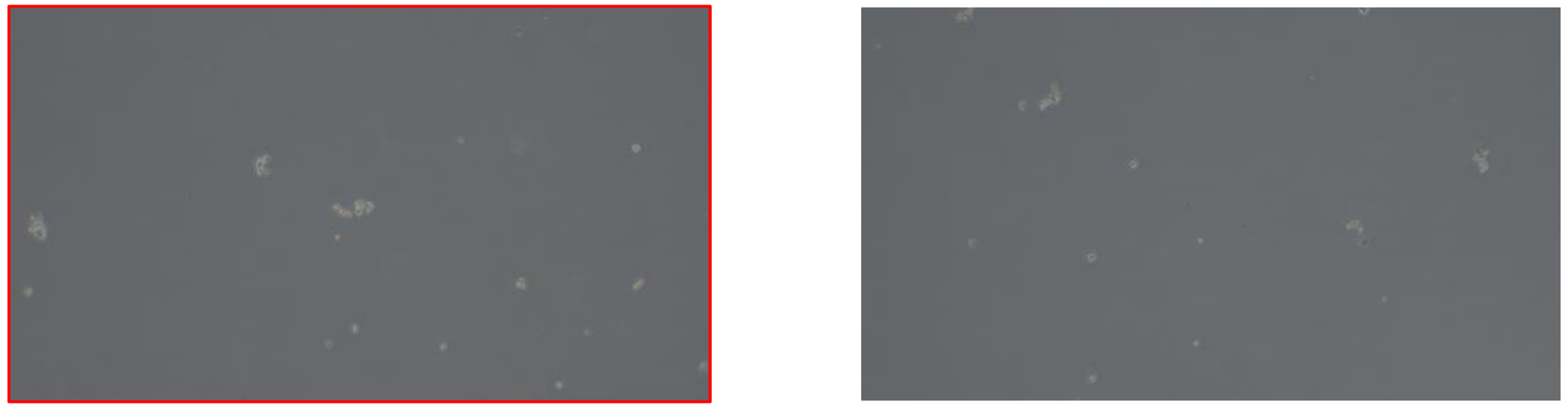

Figure S8A SW1990: shARNTL2 (three repeats)

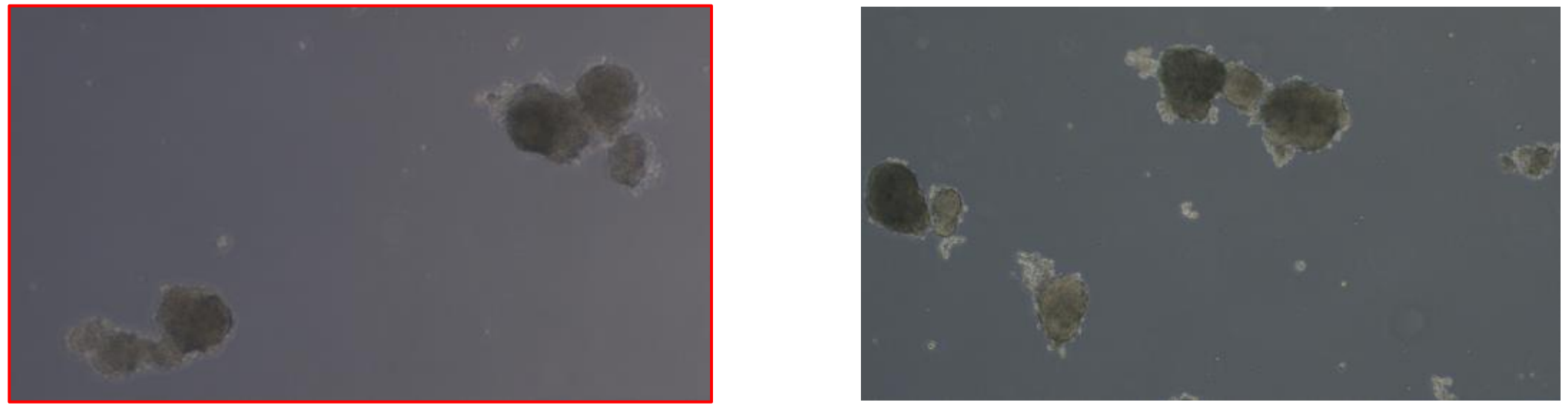

Figure S8A SW1990: shARNTL2+SERPINE1 (three repeats)

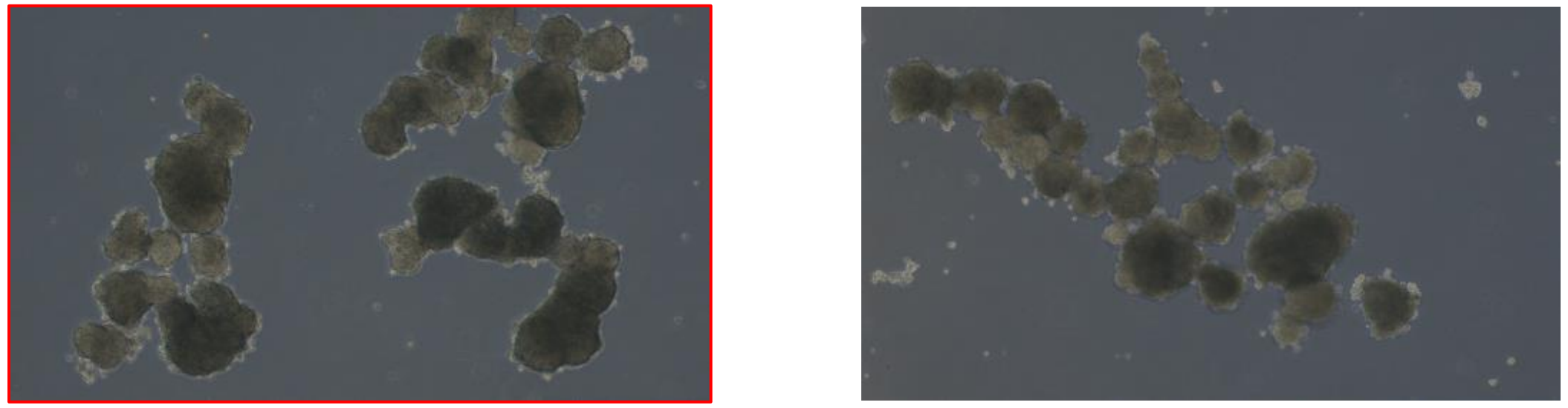

Figure S8B BxPC-3: Vector (three repeats)

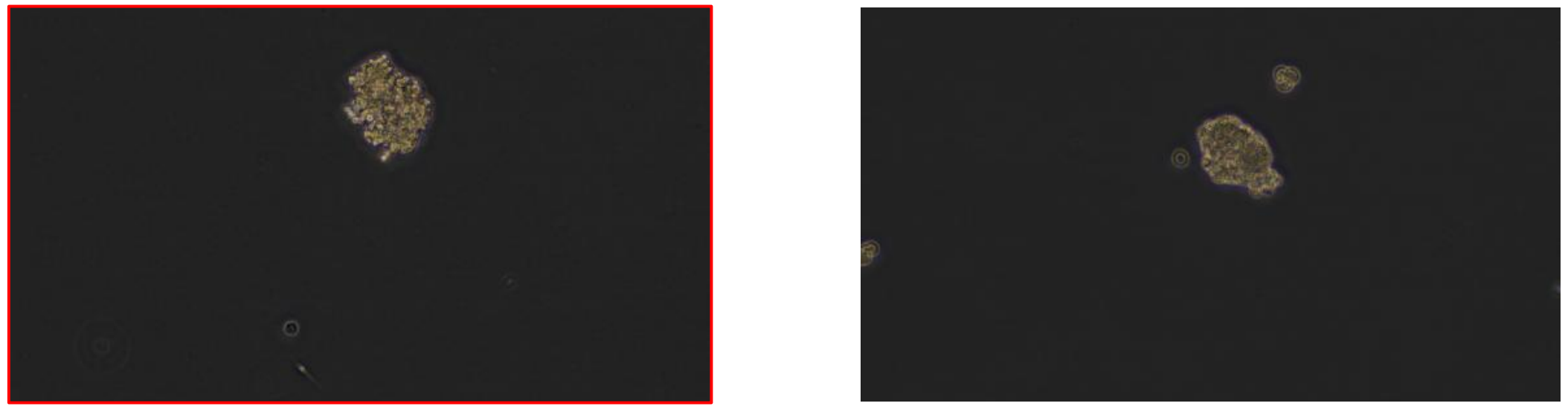

Figure S8B BxPC-3: SERPINE1 (three repeats)

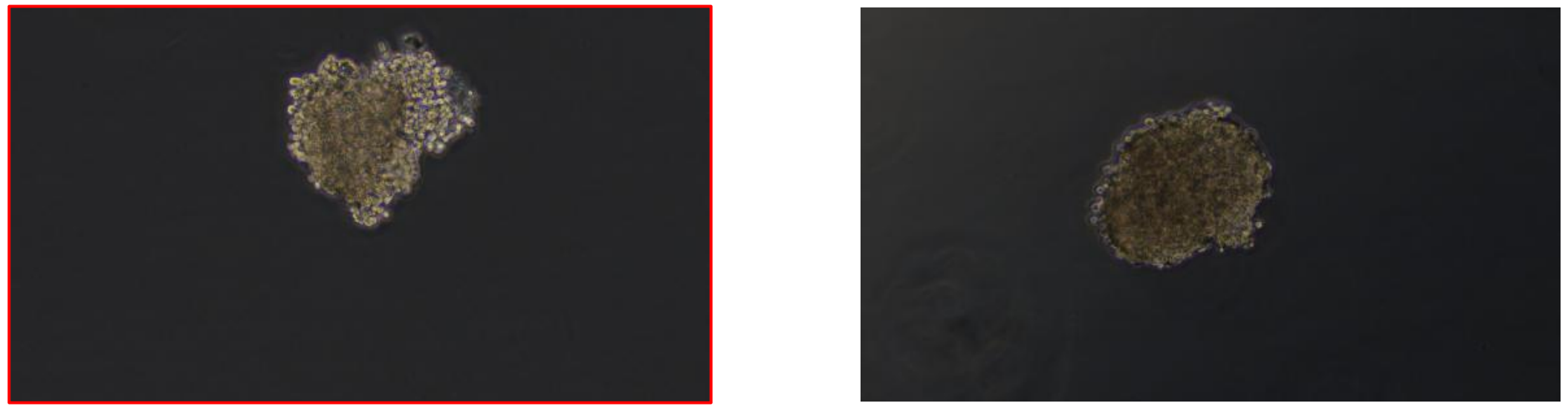

Figure S8B BxPC-3: ARNTL2 (three repeats)

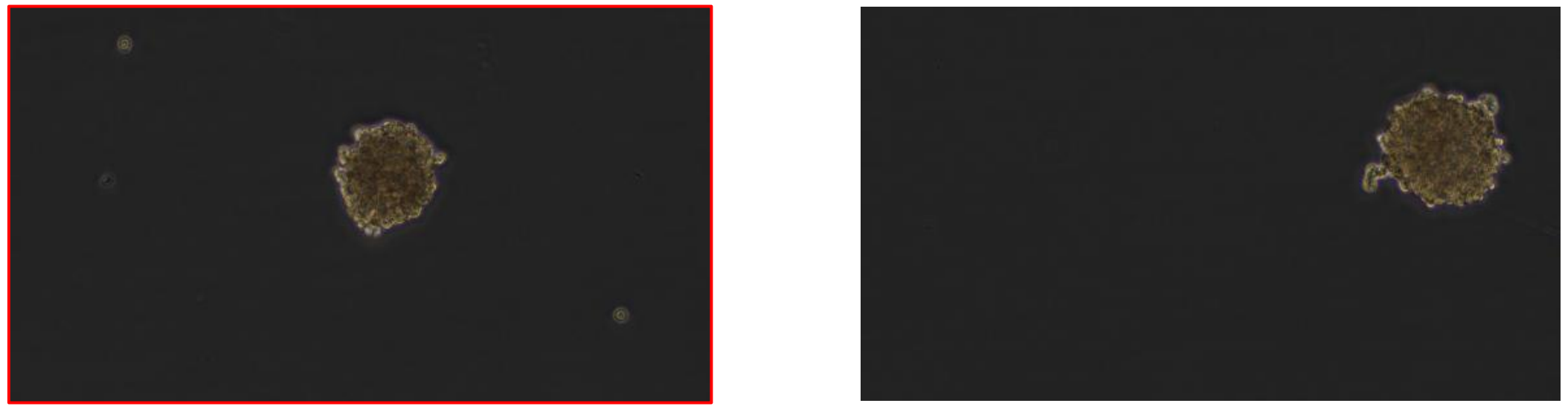

Figure S8B BxPC-3: ARNTL2+shSERPINE1 (three repeats)

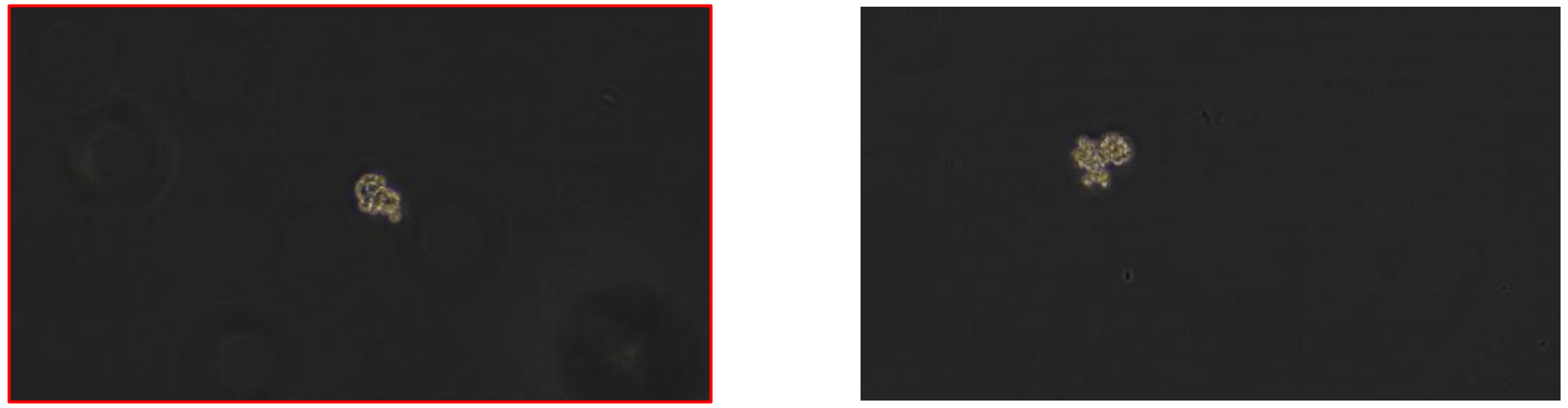

Figure S8B PANC-1: shNC (three repeats)

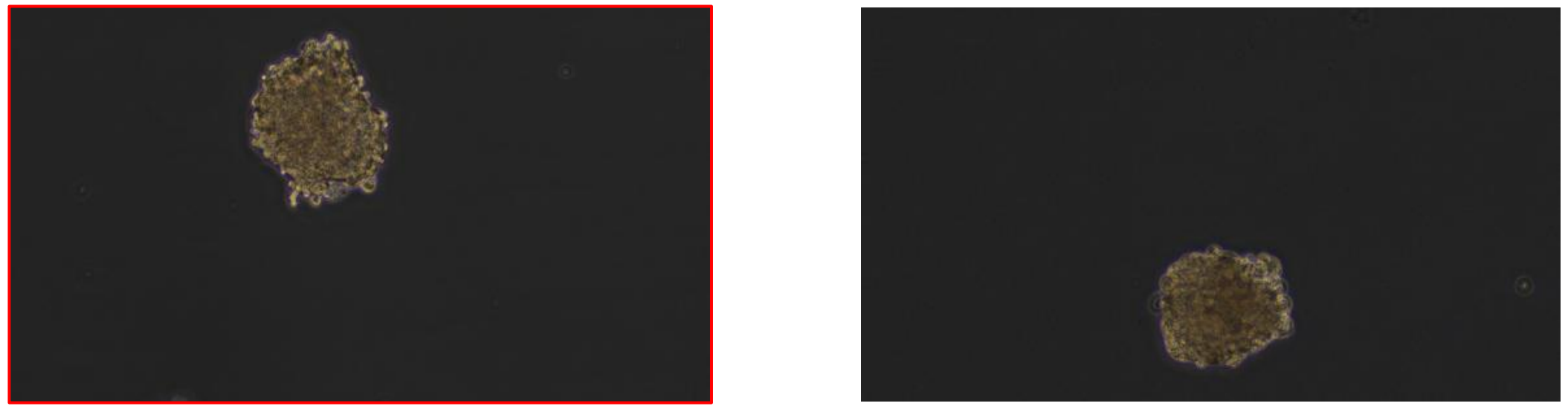

Figure S8B PANC-1: shSERPINE1 (three repeats)

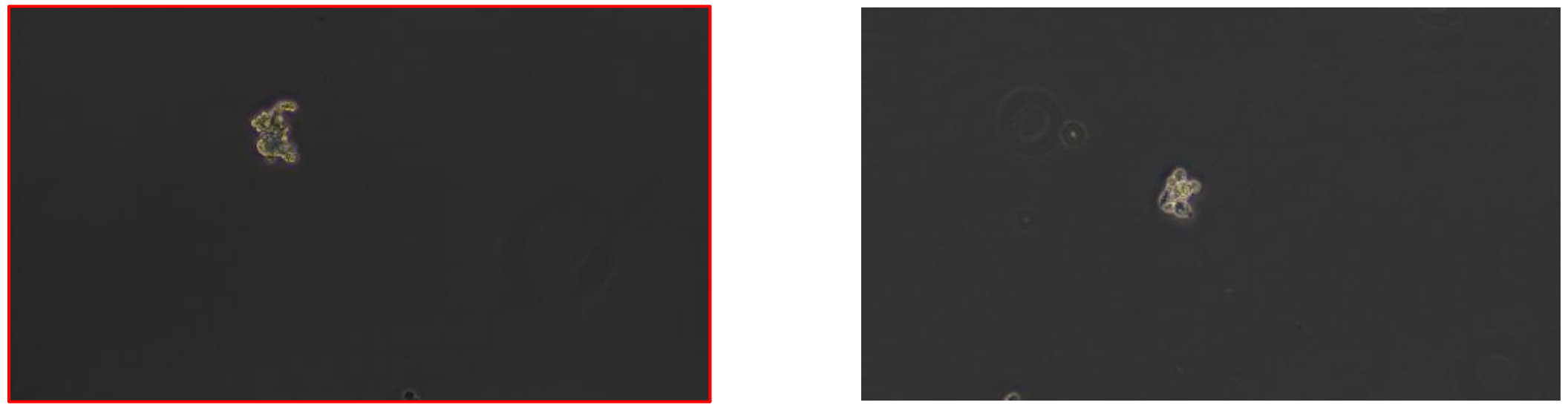

Figure S8B PANC-1: shARNTL2 (three repeats)

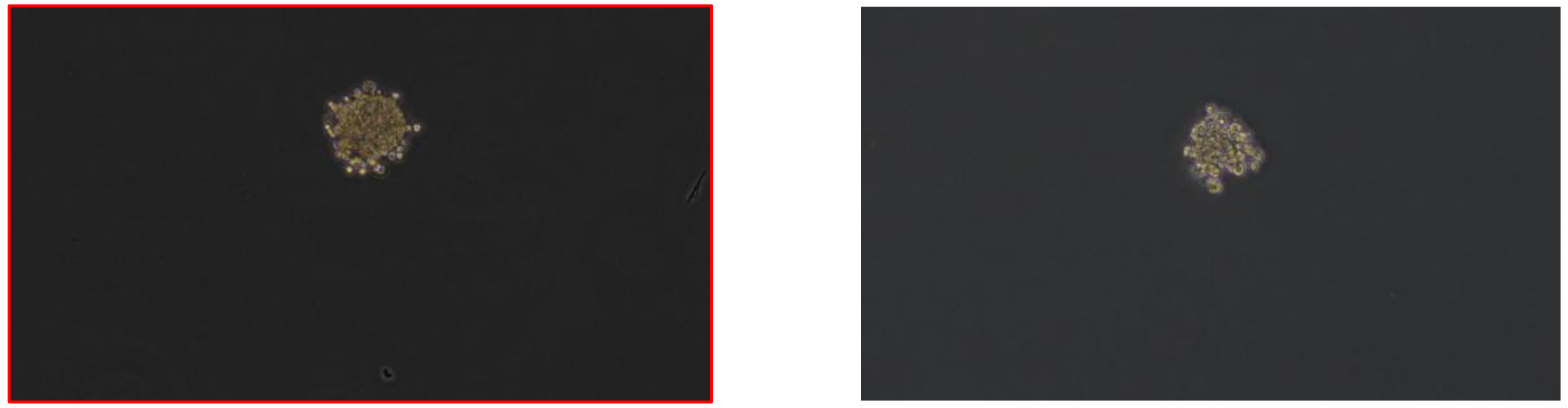

Figure S8B PANC-1: shARNTL2+SERPINE1 (three repeats)

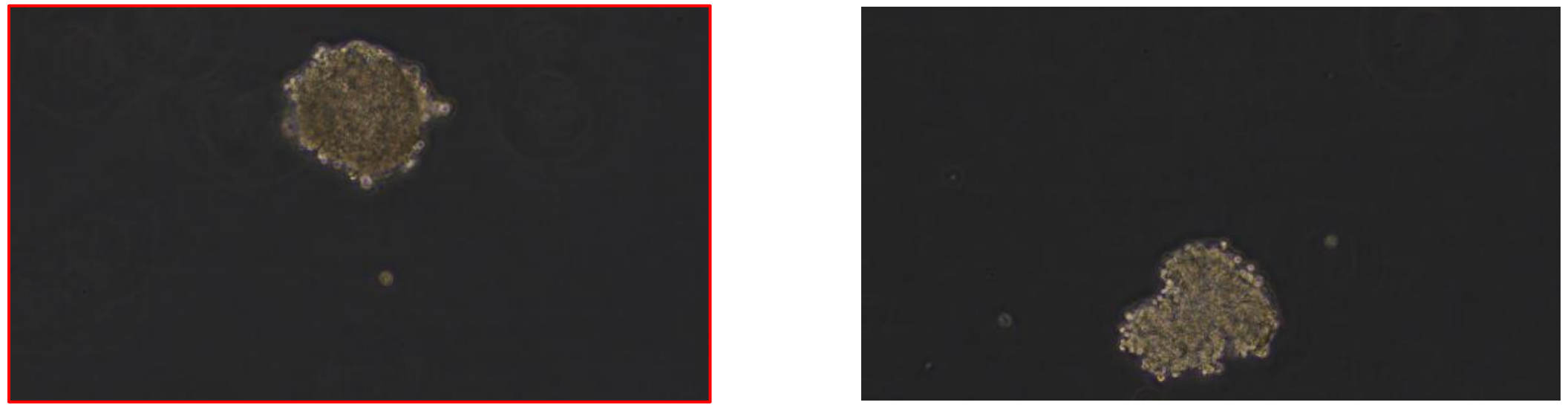

Figure S8B SW1990: shNC (three repeats)

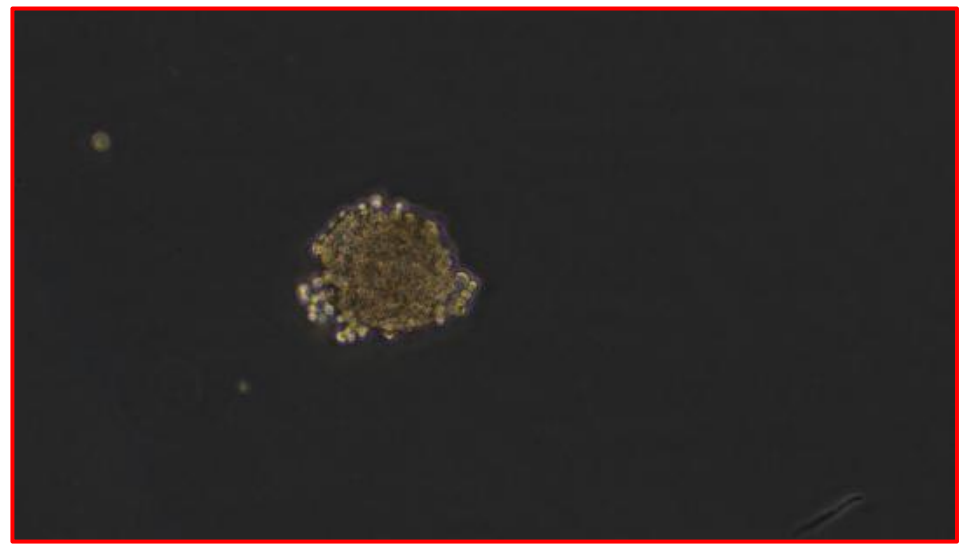

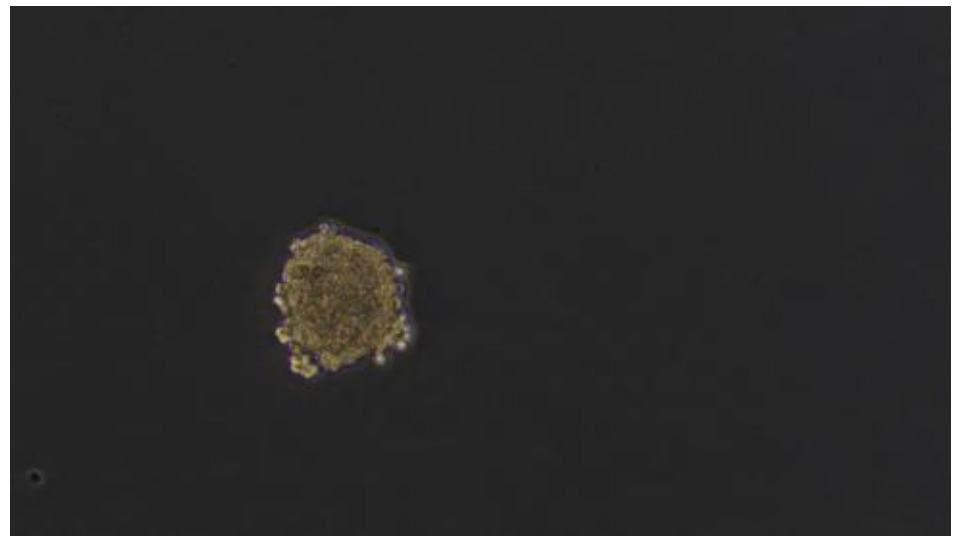

Figure S8B SW1990: shSERPINE1 (three repeats)

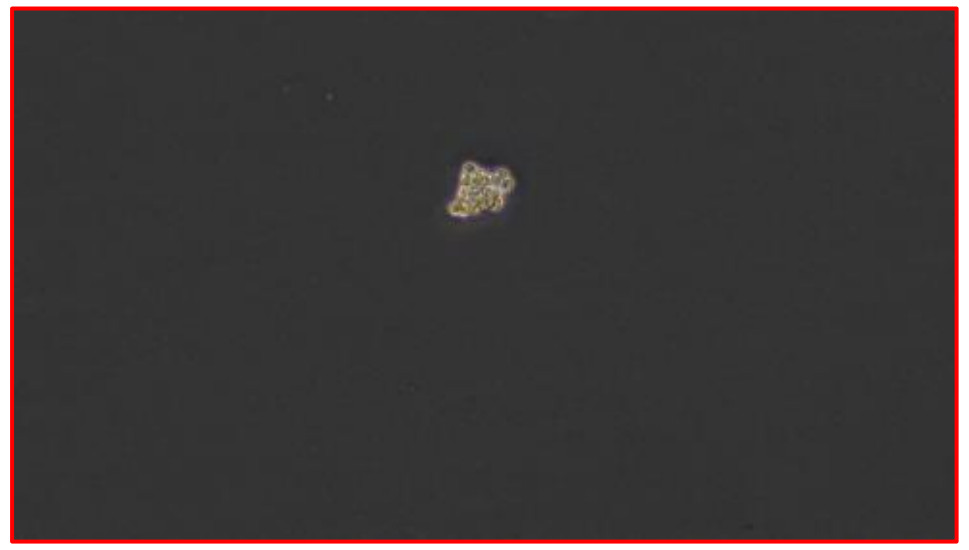

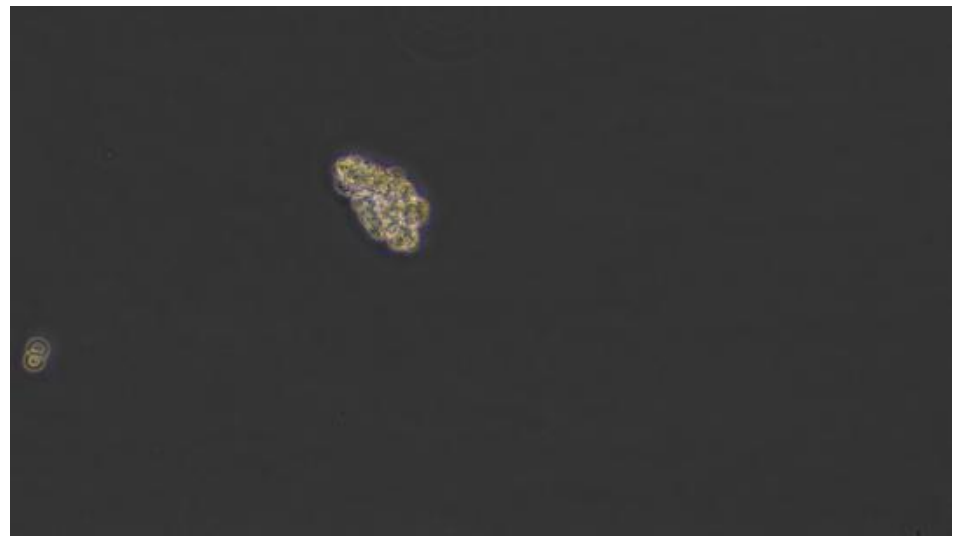

Figure S8B SW1990: shARNTL2 (three repeats)

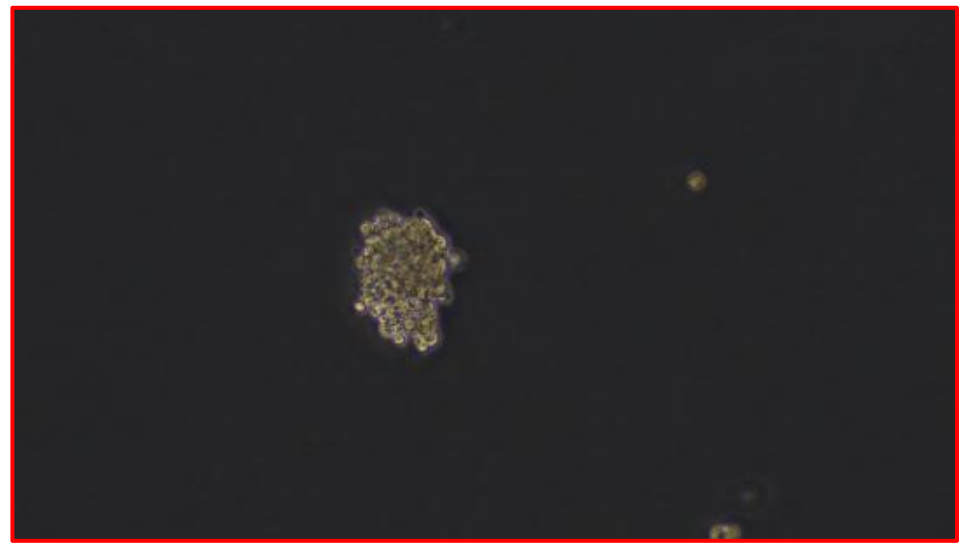

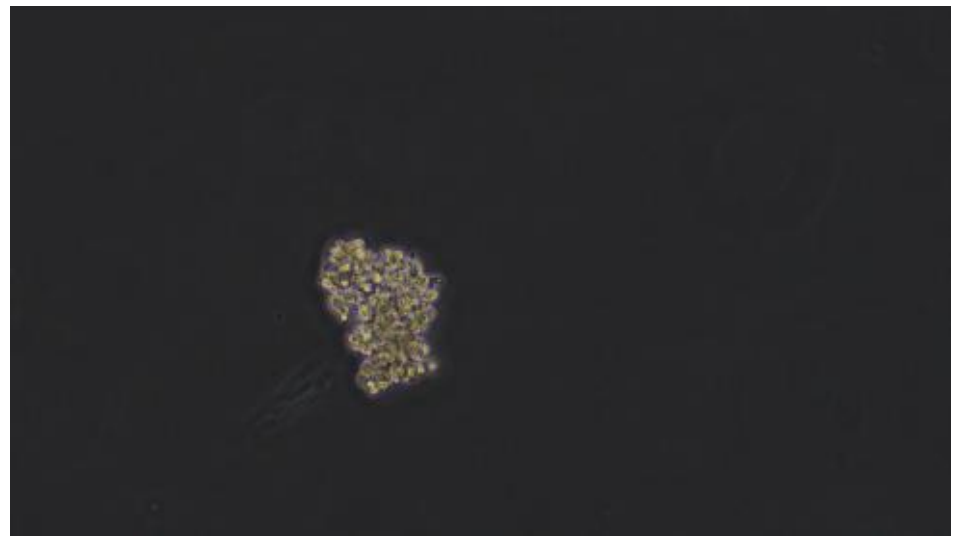

Figure S8B SW1990: shARNTL2+SERPINE1 (three repeats)

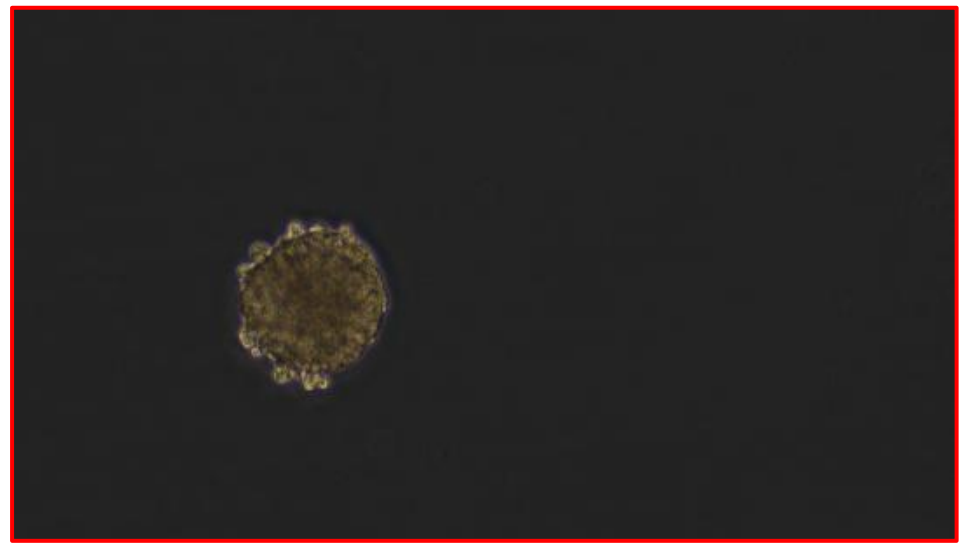

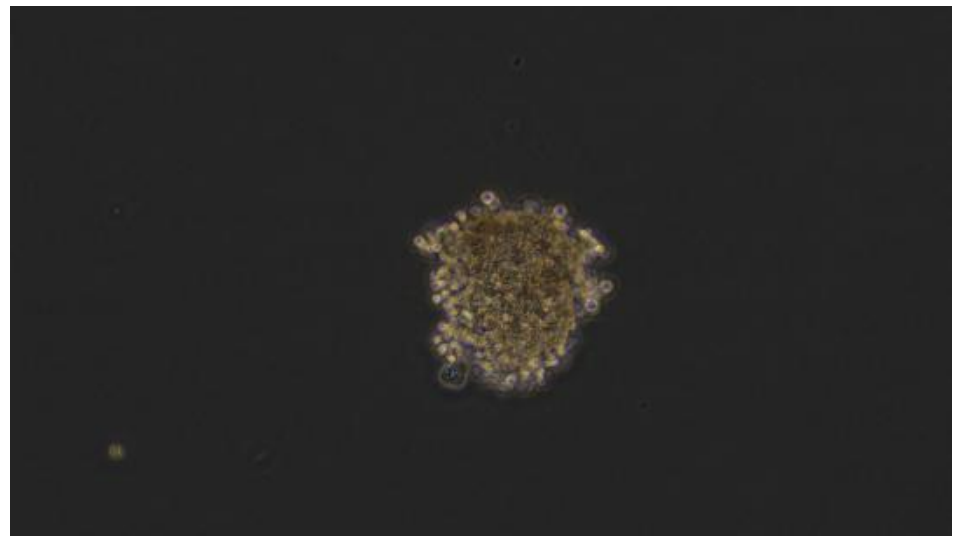

Supplement: Supplementary file 3 [file mmc3.pdf]
