# Supplementary material for: ARNTL2 promotes metastatic phenotypes through SERPINE1 transcription in KRAS G12D pancreatic cancer
Source: iScience. 2026 Sep 8;29(9):117479. doi: 10.1016/j.isci.2026.117479 (PMC13573702; doi:10.1016/j.isci.2026.117479)
Supplement: Supplementary file 4 [file mmc4.pdf]

Sequence (5'→3')

WT:acgcgtgcagcaccctggccacccttccatccacaacatccagaccacacggccaagggcacctgaccctgtcaaaacc
ccaaatccagctgggcgcggtggctcatgcctgtaatcccagcatttgggaggccgaggcagccggatcacgaagtcaggagtt
cgagaccagcctgaccaacatggtgaaaccccgtctctactaaaatacaaaaattagccgggcgtggtggtgcacacctgtaatc
ccagctactcgggaggctgaggcaggagaaccacttgaacctgggaggcggagattgcagtgagccaagatagtgctactgca
ctcagcctgggcaacaaaataagactccgtctcaaaacagaaaaacaaaaaaaacaaaaaacaacaacaacaacaaaatc
cactagtctagccctacatgctcacccactgctagccagtttccaccctctacagcagaggtacccaacctctgggccaggggccc
gtactggtccatagcctgttaggaaccaggctgcataacaggaggtgagtggcaggtgagtgaaatttcatctgtagttacagcca
ctcctcatcactcgcattaccaccagagctccactccctgtcagatcagcggcggcattagattctcataggagctcgaaccctattc
taaactgttcatgtgagggatctaggttgcaagctccctatgagaatctaatgcctgatgatctgtcacggtctcccatcacccctagat
gggaccatctagttgcaggaaaacaagctcagggctcccactgattctacacgatggtgaattgtggaattatttcattatatatatta
caatgtaataataatagaaataaagcacacaataaatgtaatgtgcttgaatcatcccgaaaccatcccaccctggtctgtgaaaa
aattgtcttccatgaaaccagtccctggtgccaaaaacgttgaggaccactgctccacagaatctatcggtcactcttcctcccctca
ccccccttgccctaaaagcacaccctgcaaacctgccatgaattgacactctgtttctatcccttttccccttgtgtctgtgtctggaggaa
gaggataaaggacaagctgccccaagtcctagcgggcagctcgaagaagtgaaacttacacgttggtctcctgtttccttaccaa
gcttttaccatggtaacccctggtcccgttcagccaccaccaccccacccagcacacctccaacctcagccagacaaggttgttga
cacaagagagccctcaggggcacagagagagtctggacacgtggggagtcagccgtgtatcatcggaggcggccgggcaca
tggcagggatgagggaaagaccaagagtcctctgttgggcccaagtcctagacagacaaaacctagacaatcacgtggctggc
tgcatgccctgtggctgttgggctgggcccaggaggagggaggggcgctctttcctggaggtggtccagagcaccgggtggaca
gccctgggggaaaacttccacgttttgatggaggttatctttgataactccacagtgacctggttcgccaaaggaaaagcaggcaa
cgtgagctgttttttttttctccaagctgaacactaggggtcctaggctttttgggtcacccggcatggcagacagtcaacctggcagg
acatccgggagagacagacacaggcagagggcagaaaggtcaagggaggttctcaggccaaggctattggggtttgctcaatt
gttcctgaatgctcttacacacgtacacacacagagcagcacacacacacacacacacatgcctcagcaagtcccagagaggg
aggtgtcgagggggacccgctggctgttcagacggactcccagagccagtgagtgggtggggctggaacatgagttcatctatttc
ctgcccacatctggtataaaaggaggcagtggcccacagaggagcagatct

Promoter1:acgcgtgcagcaccctggccacccttccatccacaacatccagaccacacggccaagggcacctgaccctgt
caaaaccccaaatccagctgggcgcggtggctcatgcctgtaatcccagcatttgggaggccgaggcagccggatcacgaagt
caggagttcgagaccagcctgaccaacatggtgaaaccccgtctctactaaaatacaaaaattagccgggcgtggtggtgcaca
cctgtaatcccagctactcgggaggctgaggcaggagaaccacttgaacctgggaggcggagattgcagtgagccaagatagt
gctactgcactcagcctgggcaacaaaataagactccgtctcaaaacagaaaaacaaaaaaaacaaaaaacaacaacaac
aacaaaatccactagtctagccctacatgctcacccactgctagccagtttccaccctctacagcagaggtacccaacctctgggc
caggggcccgtactggtcagatct

Promoter2:acgcgtcatagcctgttaggaaccaggctgcataacaggaggtgagtggcaggtgagtgaaatttcatctgtagt
tacagccactcctcatcactcgcattaccaccagagctccactccctgtcagatcagcggcggcattagattctcataggagctcga
accctattctaaactgttcatgtgagggatctaggttgcaagctccctatgagaatctaatgcctgatgatctgtcacggtctcccatca
cccctagatgggaccatctagttgcaggaaaacaagctcagggctcccactgattctacacgatggtgaattgtggaattatttcatt

atatatattacaatgtaataataatagaaataaagcacacaataaatgtaatgtgcttgaatcatcccgaaaccatcccaccctggtc tgtgaaaaaattgtcttccatgaaaccagtccctggtgccaaaaacgttgaggaccactgctccacagaatctatcggagatct

Promoter3:acgcgttcactcttcctcccctcacccccttgccctaaaagcacaccctgcaaacctgccatgaattgacactctgt ttctatcccttttccccttgtgtctgtgtctggaggaagaggataaaggacaagctgccccaagtcctagcgggcagctcgaagaag tgaaacttacacgttggtctcctgtttccttaccaagcttttaccatggtaacccctggtcccgttcagccaccaccaccccacccagc acacctccaacctcagccagacaaggttgttgacacaagagagccctcaggggcacagagagagtctggacacgtggggagt cagccgtgtatcatcggaggcggccgggcacatggcagggatgagggaaagaccaagagtcctctgttgggcccaagtcctag acagacaaaacctagacaatcacgtggctggctgcatgccctgtggctgttgggctgggcccaggaggagggaggggcgctctt tcctagatct

Promoter4:acgcgtggaggtggtccagagcaccgggtggacagccctgggggaaaacttccacgttttgatggaggttatctt tgataactccacagtgacctggttcgccaaaggaaaagcaggcaacgtgagctgtttttttttctccaagctgaacactaggggtcc taggctttttgggtcacccggcatggcagacagtcaacctggcaggacatccgggagagacagacacaggcagagggcagaa aggtcaagggaggttctcaggccaaggctattggggtttgctcaattgttcctgaatgctcttacacacgtacacacacagagcagc acacacacacacacacacatgcctcagcaagtcccagagagggaggtgtcgagggggacccgctggctgttcagacggactc ccagagccagtgagtgggtggggctggaacatgagttcatctatttcctgcccacatctggtataaaggaggcagtggcccaca gaggagcagatct
